# Supplementary material for: Design, Synthesis and Evaluation of Novel Derivatives of Curcuminoids with Cytotoxicity
Source: Int J Mol Sci. 2021 Nov 10;22(22):12171. doi: 10.3390/ijms222212171 (PMC8624111; doi:10.3390/ijms222212171)
Supplement: Supplementary file 1 [file ijms-22-12171-s001.zip › ijms-1394058-supplementary.pdf]

Design, Synthesis and Evaluation of Novel Derivatives of Curcuminoids with Cytotoxicity

Chen-Yin Chen[1#], Jin-Cherng Lien[1#], Chien-Yu Chen[1], Chin-Chuan Hung*[12] and Hui-Chang Lin*[1]

[1] School of Pharmacy, China Medical University, No. 100, Sec. 1, Jingmao Rd., Beitun Dist., Taichung City, 406040, Taiwan, ROC
[2] Department of Pharmacy, China Medical University Hospital, 2 Yude Road, Taichung 40447, Taiwan, R.O.C.
* the correspondence author; email: cchung@mail.cmu.edu.tw.
# the authors contributed to the work equally

(Total 1 pages include this cover page)

Supplementary Materials

^{1}H, ^{13}C NMR and mass for compound 1-24, 1a-7a, 12a, 14a, MD1-13......................................S2-19

^{1}H, ^{13}C NMR spectra for compound 1-24, 1a-7a, 12a, 14a, MD1-13.......................................S20-100

Supplementary Materials

Mass and ¹H and ¹³ C NMR data

(E)-5-hydroxy-1,7-diphenylhept-1-en-3-one (1)

Reference ompound [22]

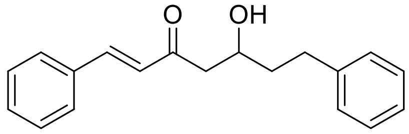

Known compound; white solid; yield 77.7%. ^{1}H NMR (500 MHz, $CDCl_3$) δ 7.49-7.46 (m, 3H), 7.34-7.32 (m, 3H), 7.21-7.11 (m, 5H), 6.63 (d, *J* = 15 Hz, 1H), 4.11-4.07 (m, 1H), 2.83-2.63 (m, 4H), 1.85-1.80 (m, 1H), 1.74-1.68 (m, 1H); ^{13}C NMR (125 MHz,$CDCl_3$) δ 201.0, 143.8, 142.1, 134.3, 131.0, 129.2, 128.6, 128.6, 126.4, 126.0, 67.3, 47.0, 38.3, 32.0; HRMS [ESI]$^+$ calculated for $C_{19}H_{21}O_2$: 281.1536 [M + H]$^+$; found: 281.1545.

(1E,4E)-1,7-diphenylhepta-1,4-dien-3-one (1a)

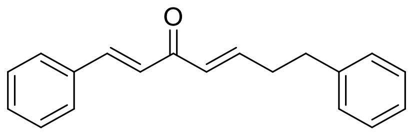

Known compound; yellow viscous oil; yield 90.2%. ^{1}H NMR (500 MHz, CD_3OD) δ 7.58-7.55 (m, 3H), 7.34-7.33 (m, 3H), 7.22-7.09 (m, 5H), 7.03-6.99 (m, 2H), 6.43 (d, *J* = 15 Hz, 1H), 2.76 (t, *J* = 7.5 Hz, 2H), 2.57-2.53 (m, 2H); ^{13}C NMR (125 MHz, CD_3OD) δ 191.8, 149.4, 145.2, 142.5, 136.3, 131.8, 130.8, 130.2, 129.7, 129.6, 129.6, 127.3, 125.9, 35.7, 35.6. HRMS [ESI]$^+$ calculated for $C_{19}H_{19}O$: 263.1430 [M + H]$^+$; found: 263.1429.

(E)-1-(3,4-dimethoxyphenyl)-5-hydroxy-7-phenylhept-1-en-3-one (2)

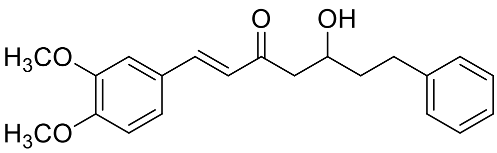

Yellowish powder; yield 80.3%; m.p.: 66.6-67.5 °C. ^{1}H NMR (500 MHz, CD_3OD) δ 7.47 (d, *J* = 15 Hz, 1H), 7.17-7.04 (m, 7H), 6.84 (d, *J* = 5 Hz, 1H), 6.63 (d, *J* = 15 Hz, 1H), 4.08-4.03 (m, 1H), 3.75 (s, 6H), 2.82-2.78 (m, 1H), 2.71-2.59 (m, 2H), 2.58-2.56 (m, 1H), 1.74-1.71 (m, 2H); ^{13}C NMR (125 MHz, CD_3OD) δ 201.8, 153.2, 150.8, 145.3, 143.5, 129.3, 129.0, 126.9, 125.7, 124.7, 112.7, 111.8, 68.9, 56.6, 56.5, 49.1, 40.4, 33.0. HRMS [ESI]$^+$ calculated for $C_{21}H_{25}O_4$: 341.1747 [M + H]$^+$; found: 341.1757.

(E)-5-hydroxy-7-phenyl-1-(3,4,5-trimethoxyphenyl)hept-1-en-3-one (3)

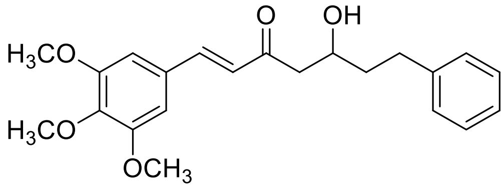

Yellow viscous oil; yield 74.3%. ^{1}H NMR (500 MHz, CD_3OD) δ 7.47 (d , *J* = 15 Hz, 1H), 7.17-7.11 (m, 4H), 7.08-7.04 (m, 1H), 6.82 (s, 1H), 6.69 (d, *J* = 15 Hz, 1H), 4.09-4.04 (m, 1H), 3.75 (s, 6H), 3.69 (s, 3H), 2.82-2.78 (m, 1H), 2.72-2.67 (m, 2H), 2.61-2.55 (m, 1H), 1.73-1.69 (m, 2H); ^{13}C NMR (125 MHz, CD_3OD) δ 201.7, 154.9, 145.1, 143.5, 141.6, 131.8, 129.6, 129.5, 127.2, 126.9, 107.1, 68.8, 61.3, 56.8, 49.1, 40.5, 33.0. HRMS [ESI]$^+$ calculated for $C_{22}H_{27}O_5$: 371.1853 [M + H]$^+$; found: 371.1862.

(1E,4E)-7-phenyl-1-(3,4,5-trimethoxyphenyl)hepta-1,4-dien-3-one (3a)

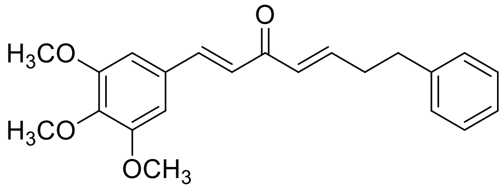

Yellow oil; yield 86.5%. ^{1}H NMR (500 MHz, CD_3OD) δ 7.52 (d, *J* = 15 Hz, 1H), 7.22-6.91 (m, 9H), 6.45 (d, *J* =15 Hz, 1H), 3.82 (s, 6H), 3.73 (s, 3H), 2.79-2.76 (m, 2H), 2.61-2.54 (m, 2H); ^{13}C NMR (125 MHz, CD_3OD) δ191.8, 155.0, 149.3, 145.4, 132.1, 130.9, 129.7, 129.6, 127.3, 125.3, 107.3, 61.4, 56.9, 35.8, 35.7. HRMS [ESI]$^+$ calculated for $C_{22}H_{25}O_4$: 353.1747 [M + H]$^+$; found: 353.1746.

(E)-5-hydroxy-1-(4-methoxyphenyl)-7-phenylhept-1-en-3-one (4)

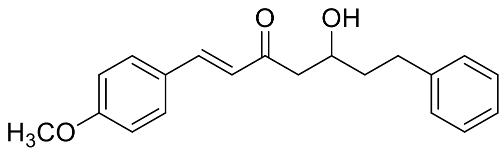

Yellow oil; yield 83.2%. ^{1}H NMR (500 MHz, CD_3OD) δ 7.51-7.46 (m, 3H), 7.17-7.10 (m, 4H), 7.06-7.03 (m, 1H), 6.85 (d, *J* = 5 Hz, 2H), 6.62 (d, *J*=15 Hz, 1H), 4.78-4.01 (m, 1H), 3.72 (s, 3H), 2.81-2.77 (m, 1H), 2.74-2.67 (m, 2H), 2.61-2.55 (m, 1H) 1.76-1.73 (m, 2H); ^{13}C NMR (125 MHz, CD_3OD) δ 201.9, 163.5, 145.1, 143.5, 131.5, 129.6, 129.5, 128.6, 126.9, 125.4, 115.6, 68.9, 56.0, 48.9, 40.4, 33.0. HRMS [ESI]$^+$ calculated for $C_{20}H_{23}O_3$: 311.1642 [M + H]$^+$; found: 311.1651.

(1E,4E)-1-(4-methoxyphenyl)-7-phenylhepta-1,4-dien-3-one (4a)

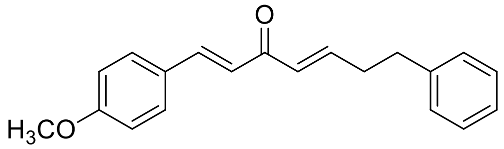

Yellow solid; yield 88.7%; m.p.: 55-54 °C. ^1H NMR (500 MHz, CD_3OD) δ 7.55-7.51 (m, 3H), 7.21-7.08 (m, 5H), 6.99-6.85 (m, 4H), 6.42 (d, *J* = 15 Hz, 1H), 3.75 (s, 3H), 2.76-2.73 (m, 2H), 2.55-2.50 (m, 2H); ^{13}C NMR (125 MHz, CD_3OD) δ 191.9, 163.6, 148.8, 145.4, 142.5, 131.6, 130.8, 129.6, 129.6, 128.9, 127.3, 123.6, 115.7, 56.1, 35.7, 35.7. HRMS [ESI]$^+$ calculated for $C_{20}H_{21}O_2$: 293.1536 [M + H]$^+$; found: 293.1533.

(E)-5-hydroxy-1-(4-hydroxyphenyl)-7-phenylhept-1-en-3-one (5)

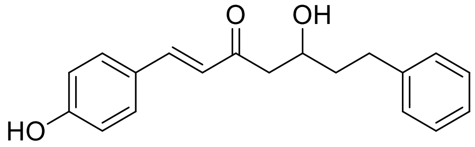

Yellow solid; yield 78.6%; m.p. 111-112 °C. NMR (500 MHz, CD_3OD) δ 7.45 (d, *J* = 15 Hz, 1H), 7.36-7.34 (m, 2H), 7.14-7.07 (m, 4H), 7.03-7.01 (m, 1H), 6.72 (d, *J* = 10 Hz, 2H), 6.54 (d, *J* = 15 Hz, 1H), 4.77 (brs, 2H), 4.04-3.99 (m, 1H), 2.78-2.72 (m, 1H), 2.70-2.62 (m, 2H), 2.59-2.53 (m, 1H), 1.71-1.66 (m, 2H); ^{13}C NMR (125 MHz, CD_3OD) δ 202.0, 161.7, 145.7, 143.5, 131.8, 129.6, 129.6, 127.4, 127.0, 124.7, 117.1, 69.0, 48.8, 40.4, 33.1. HRMS [ESI]$^+$ calculated for $C_{19}H_{19}O_3$: 295.1329 [M + H]$^+$; found: 295.1339.

(1E,4E)-1-(4-hydroxyphenyl)-7-phenylhepta-1,4-dien-3-one (5a)

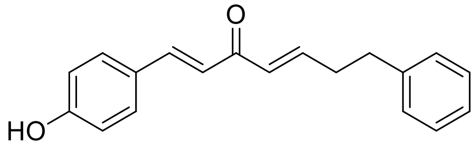

Yellow solid; yield 81.5%; m.p.: 130.1-131.3 °C. NMR (500 MHz, CD_3OD) δ 7.51 (d, *J* = 15 Hz, 1H), 7.43 (d, *J* = 5 Hz, 2H), 7.20-7.07 (m, 5H), 6.97-6.93 (m, 1H), 6.81 (d, *J* = 15 Hz, 1H), 6.72 (d, *J* = 5 Hz, 2H), 6.41 (d, *J* = 15 Hz, 1H), 2.76-2.73 (m, 2H), 2.54-2.50 (m, 2H); ^{13}C NMR (125 MHz, CD_3OD) δ 192.0, 161.8, 148.6, 145.9, 142.5, 131.8, 129.6, 129.6, 127.7, 127.3, 122.8, 117.1, 35.7, 35.7. HRMS [ESI]$^+$ calculated for $C_{19}H_{17}O_2$: 277.1223 [M + H]$^+$; found: 277.1223.

(E)-1-(4-fluorophenyl)-5-hydroxy-7-phenylhept-1-en-3-one (6)

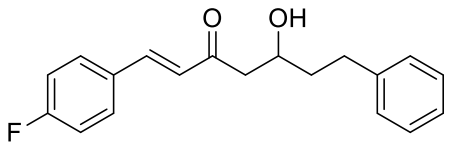

Yellow solid; yield 73.5%; m.p.: 100.9-101.8 °C. NMR (500 MHz, CD_3OD) δ 7.59-7.57 (m, 2H), 7.52 (d, *J* = 15 Hz, 1H), 7.17-7.11 (m, 4H), 7.07-7.04 (m, 3H), 6.71 (d, *J* = 15 Hz, 1H), 4.07-4.02 (m, 1H), 2.84-2.79 (m, 1H), 2.75-2.69 (m, 2H), 2.63-2.57 (m, 1H), 1.75-1.68 (m, 2H); ^{13}C NMR (125

MHz, CD_3OD) δ 201.7, 166.7, 164.7, 143.6, 143.5, 132.6, 132.6, 131.9, 131.8, 129.6, 129.5, 127.7, 127.0, 117.2, 117.0, 68.8, 49.1, 40.5, 33.0. HRMS [ESI]$^+$ calculated for $C_{19}H_{20}O_2F$: 299.1442 [M + H]$^+$; found: 299.1452.

(1E,4E)-1-(4-fluorophenyl)-7-phenylhepta-1,4-dien-3-one (6a)

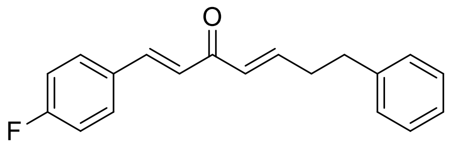

Yellow oil; yield 77.8%. NMR (500 MHz, CD_3OD) δ 7.62-7.59 (m, 2H), 7.53 (d, *J* = 10 Hz, 1H), 7.21-6.94 (m, 9H), 6.41 (d, *J* = 15 Hz, 1H), 2.76-2.73 (m, 2H), 2.55-2.51 (m, 2H); ^{13}C NMR (125 MHz, CD_3OD) δ 191.6, 166.6, 164.7, 149.5, 143.8, 142.5, 132.7, 131.9, 131.9, 130.8, 129.6, 127.3, 125.7, 117.2, 117.0, 35.7, 35.6. HRMS [ESI]$^+$ calculated for $C_{19}H_{18}OF$: 281.1336 [M + H]$^+$; found: 281.1346.

(E)-1-(4-chlorophenyl)-5-hydroxy-7-phenylhept-1-en-3-one (7).

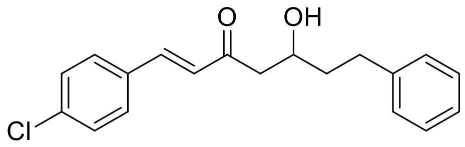

Yellow solid; yield 75.8%; m.p.: 85-85.9 °C. NMR (500 MHz, CD_3OD) δ 7.50-7.46 (m, 3H), 7.3 (d, *J* = 15 Hz, 2H), 7.20-7.10 (m, 4H), 7.06-7.03 (m, 1H), 6.73 (d, *J* = 15 Hz, 1H), 4.07-4.02 (m, 1H), 2.83-2.79 (m, 1H), 2.73-2.68 (m, 2H), 2.62-2.57 (m, 1H), 1.74-1.69 (m, 2H); ^{13}C NMR (125 MHz, CD_3OD) δ 201.5, 143.5, 143.3, 137.5, 134.8, 131.1, 130.3, 129.6, 129.5, 128.4, 126.9, 68.7, 49.2, 40.4, 33.0. HRMS [ESI]$^+$ calculated for $C_{19}H_{20}O_2Cl$: 315.1146 [M + H]$^+$; found: 315.1154.

(1E,4E)-1-(4-chlorophenyl)-7-phenylhepta-1,4-dien-3-one (7a)

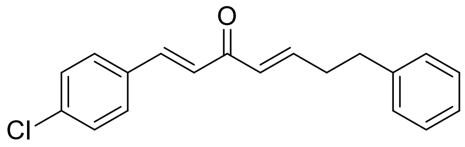

Yellow oil; yield 80.2 %. NMR (500 MHz, CD_3OD) δ 7.54-7.48 (m, 3H), 7.32-7.30 (d, *J* = 5 Hz, 2H), 7.19-7.16 (m, 2H), 7.13-7.06 (m, 3H), 7.02-6.96 (m, 2H), 6.39 (d, *J* = 15 Hz, 1H), 2.75-2.72 (m, 2H), 2.54-2.49 (m, 2H); ^{13}C NMR (125 MHz, CD_3OD) δ191.5, 149.7, 143.5, 142.4, 137.5, 135.0, 131.1, 130.8, 130.4, 129.6, 127.3, 126.5, 35.7, 35.6. HRMS [ESI]$^+$ calculated for $C_{19}H_{18}OCl$: 297.1041 [M + H]$^+$; found: 297.1048.

(E)-5-hydroxy-7-phenyl-1-(pyridin-2-yl)hept-1-en-3-one (8)

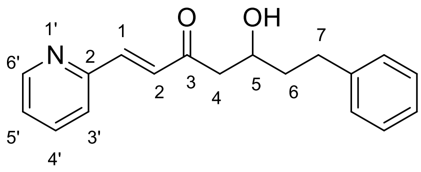

Colorless oil; yield 57%. ^{1}H NMR (500 MHz, CD_3OD) δ 8.49-8.48 (m, 1H), 7.76-7.73 (m, 1H), 7.57 (d, *J* = 5 Hz, 1H), 7.49 (d, *J* = 15 Hz, 1H), 7.30-7.27 (m, 1H), 7.15-7.08 (m, 1H), 7.05-7.01 (m, 7H), 4.09-4.02 (m, 1H), 2.86-2.81 (m, 1H), 2.76-2.67 (m, 2H), 2.61-2.56 (m, 1H), 1.73-1.68 (m, 2H); ^{13}C NMR (125 MHz, CD_3OD) δ 201.5, 154.5, 151.1, 143.5, 142.68, 139.0, 131.5, 129.6, 129.5, 126.9, 126.1, 126.0, 68.6, 49.5, 40.5, 33.1. HRMS [ESI]$^+$ calculated for $C_{18}H_{20}NO_2$: 282.1489 $[M + H]^+$; found: 282.1496.

1,5-dihydroxy-7-phenyl-1-(pyridin-3-yl)heptan-3-one (9)

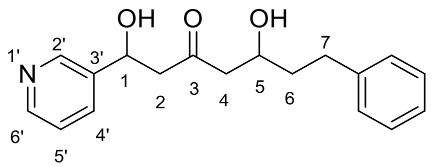

Yellow oil; yield 62.8%. ^{1}H NMR (500 MHz, $CDCl_3$) δ 8.64-8.57 (m, 2H), 7.79 (d, *J* = 10.0 Hz, 1H), 7.43-7.40 (m, 1H), 7.23-7.18 (m, 2H), 7.13-7.10 (m, 3H), 4.67-4.64 (m, 1H), 3.72-3.69 (m, 1H), 2.97-2.80 (brs, 2H), 2.79-2.77 (m, 1H), 2.75-2.69 (m, 1H), 2.63-2.60 (m, 1H), 2.47-2.33 (m, 3H), 2.06-2.02 (m, 1H), 1.88-1.85 (m, 1H); ^{13}C NMR (125 MHz, $CDCl_3$) δ 205.0, 147.1, 145.3, 141.0, 135.3, 128.5, 128.3, 126.1, 124.3, 76.7, 76.5, 48.8, 47.4, 37.6, 31.4. HRMS [ESI]$^+$ calculated for $C_{18}H_{22}NO_3$: 300.1594 $[M + H]^+$; found: 300.1600.

(E)-5-hydroxy-7-phenyl-1-(pyridin-3-yl)hept-1-en-3-one (10)

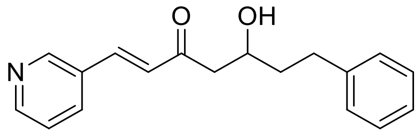

Yellow oil; yield 62.8%. ^{1}H NMR (500 MHz, CD_3OD) δ 8.62 (d, *J* = 5.0 Hz, 1H), 8.42 (d, *J* = 5.0 Hz, 1H), 7.96 (d, *J*= 5 Hz, 1H), 7.52-7.48 (m, 1H), 7.34-7.31 (m, 1H), 7.14-7.08 (m, 4H), 7.04-7.01 (m, 1H) 6.84 (d, *J* = 15.0 Hz, 1H,), 4.78-4.03 (m, 1H), 2.85-2.81 (m, 1H), 2.74-2.67 (m, 2H), 2.60-2.54 (m, 1H), 1.75-1.68 (m, 2H); ^{13}C NMR (125 MHz, CD_3OD) δ 201.0, 151.5, 150.6, 143.4, 140.3, 136.6, 132.6, 129.6, 129.5, 126.9, 125.6, 68.6, 49.4, 40.4, 33.04. HRMS [ESI]$^+$ calculated for $C_{18}H_{20}NO_2$: 282.1489 $[M + H]^+$; found: 282.1493.

(E)-5-hydroxy-7-phenyl-1-(pyridin-4-yl)hept-1-en-3-one (11)

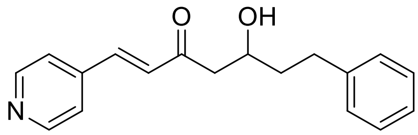

Yellow oil; yield 66.6%. ^{1}H NMR (500 MHz, CD_3OD) δ 8.45 (d, *J* = 5 Hz, 2H), 7.54-7.42 (m, 3H), 7.45-7.42 (m, 2H), 7.15-7.08 (m, 3H), 7.04-7.01 (m, 1H), 6.93 (d, *J* = 15 Hz, 1H), 4.78-4.02 (m, 1H), 2.85-2.81 (m, 1H), 2.75-2.67 (m, 2H), 2.60-2.54 (m, 1H), 1.73-1.68 (m, 2H); ^{13}C NMR (125 MHz, CD_3OD) δ 200.9, 151.0, 144.7, 143.5, 140.7, 133.9, 133.2, 133.1, 132.3, 130.1, 130.1, 129.6, 129.5, 127.0, 123.9, 68.5, 49.5, 40.5, 33.0. HRMS $[ESI]^+$ calculated for $C_{18}H_{20}O_2N$: 282.1483 $[M + H]^+$; found: 282.1489.

(E)-1-(furan-2-yl)-5-hydroxy-7-phenylhept-1-en-3-one (12)

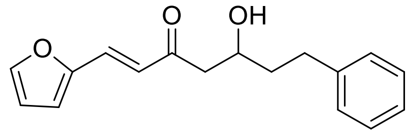

Yellowish solid; yield 63%; m.p.: 55-56 *°C*. ^{1}H NMR (500 MHz, CD_3OD) δ 7.57 (s, 1H), 7.33 (d, *J* = 15 Hz, 1H), 7.19-7.12 (m, 4H), 7.08-7.05 (m, 1H), 6.75 (d, *J* = 5 Hz, 1H), 6.56 (d, *J* = 15 Hz, 1H), 6.49-6.48 (m, 1H), 4.05-4.01 (m, 1H), 2.80-2.57 (m, 4H), 1.75-1.69 (m, 2H); ^{13}C NMR (125 MHz, CD_3OD) δ 201.4, 152.6, 147.0, 143.5, 131.2, 129.6, 129.5, 126.9, 124.9, 117.7, 113.9, 68.9, 49.1, 40.51, 33.0. HRMS $[ESI]^+$ calculated for $C_{17}H_{19}O_3$: 271.1329 $[M + H]^+$; found: 271.1334.

(1E,4E)-1-(furan-2-yl)-7-phenylhepta-1,4-dien-3-one (12a)

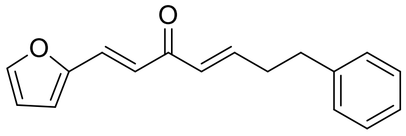

Yellow oil; yield 66%. ^{1}H NMR (500 MHz, CD_3OD) δ 7.56 (s, 1H), 7.34 (d, *J* = 15 Hz, 1H,), 7.20-7.17 (m, 2H), 7.13-7.07 (m, 3H), 6.96-6.90 (m, 1H), 6.79-6.73 (m, 2H), 6.48-6.47 (m, 1H), 6.34 (d, *J* = 15Hz, 1H), 2.74-2.71 (m, 2H), 2.52-2.48 (m, 2H); ^{13}C NMR (125 MHz, CD_3OD) δ 191.3, 152.8, 149.1, 147.0, 142.4, 131.3, 130.8, 129.6, 127.3, 123.0, 117.7, 114.0, 35.7, 35.6. HRMS $[ESI]^+$ calculated for $C_{17}H_{17}O_2$: 253.1223 $[M + H]^+$; found: 253.1215.

(E)-1-(furan-3-yl)-5-hydroxy-7-phenylhept-1-en-3-one (13)

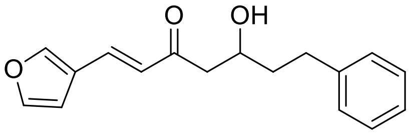

Brown oil; yield 70.5%. ^{1}H NMR (500 MHz, CD_3OD) δ 7.74 (s, 1H), 7.47 (d, *J* = 15 Hz, 1H), 7.18-7.14 (m, 2H), 7.11-7.05 (m, 4H), 6.51 (s, 1H), 6.40 (d, *J* = 15 Hz, 1H), 4.78-4.01 (m, 1H), 2.64-2.55

(m, 2H), 2.23 (m, 2H), 2.06-1.99 (m, 2H). HRMS [ESI]$^+$ calculated for $C_{17}H_{19}O_3$: 271.1246 [M + H]$^+$; found: 271.1245.

(E)-5-hydroxy-7-phenyl-1-(thiophen-2-yl)hept-1-en-3-one (14)

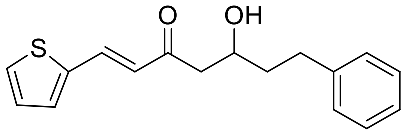

Yellow solid; yield 65%; m.p.: 94.8-95.7 ^{o}C. ^{1}H NMR (500 MHz, CD_3OD) δ 7.59 (s, 2H), 7.52 (d, *J* = 15 Hz, 1H), 7.31-7.27 (m, 1H), 7.16-7.09 (m, 4H), 7.06-7.03 (m, 1H), 6.55 (d, *J* = 15 Hz, 1H), 4.05 (brs, 1H), 2.81-2.76 (m, 1H), 2.72-2.65 (m, 2H), 2.64-2.56 (m, 1H), 1.73-1.68 (m, 2H); ^{13}C NMR (125 MHz, CD_3OD) δ 202.0, 143.4, 139.2, 138.6, 130.8, 129.5, 129.5, 128.3, 127.4, 126.9, 126.4, 68.7, 48.9, 40.3, 33.0. HRMS [ESI]$^+$ calculated for $C_{17}H_{19}O_2S$: 287.1100 [M + H]$^+$; found: 287.1108.

(1E,4E)-7-phenyl-1-(thiophen-2-yl)hepta-1,4-dien-3-one (14a)

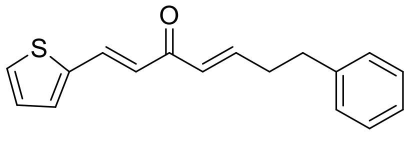

Yellowish oil; yield 72%. ^{1}H NMR (500 MHz, CD_3OD) δ 7.70 (s, 1H), 7.58 (d, *J* = 15 Hz, 1H), 7.40-7.38 (m, 2H), 7.22-7.14 (m, 2H), 7.12-7.09 (m, 2H), 7.01-6.96 (m, 1H), 6.85 (d, *J* = 15 Hz, 1H), 6.42 (d, *J* = 15 Hz, 1H), 2.78-2.75 (m, 2H), 2.56-2.52 (m, 2H); ^{13}C NMR (125 MHz, CD_3OD) δ 192.1, 149.1, 142.5, 139.6, 138.9, 130.8, 130.7, 129.6, 128.4, 127.3, 126.5, 125.6, 35.7, 35.6. HRMS [ESI]$^+$ calculated for $C_{17}H_{17}OS$: 269.0995 [M + H]$^+$; found: 269.1000.

(E)-5-hydroxy-1-(5-methylthiophen-2-yl)-7-phenylhept-1-en-3-one (15)

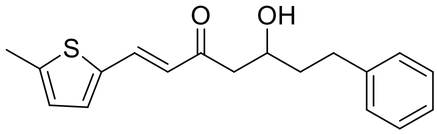

Yellowish oil; yield 60.4%. ^{1}H NMR (500 MHz, $CDCl_3$) δ 7.50 (d, *J* = 15 Hz, 1H), 7.22-7.17 (m, 2H), 7.14-7.09 (m, 3H), 7.04-7.03 (m, 1H), 6.66-6.65 (m, 1H), 6.30 (d, *J* = 15 Hz, 1H), 4.07-4.02 (m, 1H), 2.79-2.65 (m, 5H), 2.42 (s, 3H), 1.83-1.78 (m, 1H), 1.68-1.66 (m, 1H); ^{13}C NMR (125 MHz, $CDCl_3$) δ 200.2, 145.1, 141.9, 137.5, 136.4, 133.0, 128.4, 128.3, 126.8, 125.7, 123.5, 67.2, 46.7, 38.1, 31.7, 15.8. HRMS [ESI]$^+$ calculated for $C_{18}H_{21}O_2S$: 301.1257 [M + H]$^+$; found: 301.1255.

(E)-5-hydroxy-7-phenyl-1-(1H-pyrrol-2-yl)hept-1-en-3-one (16)

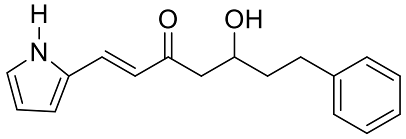

Yellow solid; yield 68.7%; m.p.: 109.5-110.3 °C. ^{1}H NMR (500 MHz, CD_3OD) δ 7.43 (d, *J* = 15 Hz, 1H), 7.18-7.11 (m, 4H), 7.07-7.05 (m, 1H), 6.91(s, 1H), 6.53-6.52 (m, 1H), 6.39 (d, *J* = 15 Hz, 1H), 6.15-6.14 (m, 1H), 4.05-4.01 (m, 1H), 2.77-2.57 (m, 4H), 1.76-1.69 (m, 2H); ^{13}C NMR (125 MHz, CD_3OD) δ 201.4, 143.6, 135.8, 129.6, 129.5, 126.9, 125.4, 120.5, 117.7, 111.9, 69.2, 48.7, 40.5, 33.1. HRMS [ESI]$^+$ calculated for $C_{17}H_{20}O_2N$: 279.1489 [M + H]$^+$; found: 279.1480.

(E)-5-hydroxy-1-(1H-indol-3-yl)-7-phenylhept-1-en-3-one (17)

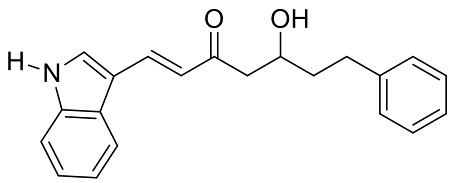

Yellow solid; yield 62.2%; m.p.: 145.6-146.0 °C. ^{1}H NMR (500 MHz, CD_3OD) δ 7.84-7.79 (m, 2H), 7.61 (s, 1H), 7.35 (d, *J* = 5 Hz, 1H), 7.17-7.03 (m, 8H), 6.73 (d, *J* = 15 Hz, 1H), 4.08-4.03 (m, 1H), 2.82-2.68 (m, 3H), 2.63-2.59 (m, 1H), 1.76-1.71 (m, 2H); ^{13}C NMR (125 MHz, CD_3OD) δ 202.3, 143.6, 140.7, 139.5, 133.4, 129.6, 129.5, 126.9, 126.8, 124.2, 122.3, 121.4, 114.3, 113.4, 69.3, 48.6, 40.5, 33.1. HRMS [ESI]$^+$ calculated for $C_{21}H_{22}O_2N$: 320.1654 [M + H]$^+$; found: 320.1655.

(E)-5-chloro-1-(4-fluorophenyl)-7-phenylhept-1-en-3-one (18)

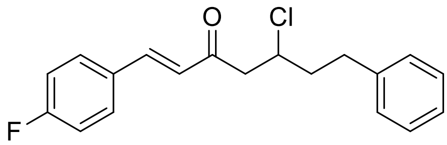

Yellow oil; yield 5.3%. ^{1}H NMR (500 MHz, CD_3OD) δ 7.62-7.59 (m, 2H), 7.59-7.54 (m, 1H), 7.22-7.06 (m, 7H), 6.72 (d, *J* = 15 Hz, 1H), 4.36-4.32 (m, 1H, H5), 3.26-3.25 (m, 1H), 3.23-3.00 (m, 1H), 2.85-2.80 (m, 1H), 2.74-2.69 (m, 1H), 2.09-2.05 (m, 1H), 2.00-1.97 (m, 1H); ^{13}C NMR (125 MHz, CD_3OD) δ 198.8, 166.7, 164.7, 143.9, 142.4, 132.4, 132.0, 131.9, 129.7, 129.7, 127.3, 127.1, 117.2, 117.1, 58.5, 49.9, 41.2, 33.7. HRMS [ESI]$^+$ calculated for $C_{19}H_{19}OClF$: 317.1103 [M + H]$^+$; found: 317.1105.

(1E,4Z)-5-hydroxy-1,7-diphenylhepta-1,4-dien-3-one (19)

Reference compound [1]

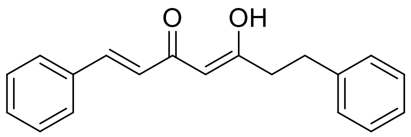

Known compound; yellow solid; yield 88%. HRMS $[ESI]^-$ calculated for $C_{19}H_{17}O_2$: 277.1223 $[M]^-$; found: 277.1232.

(1E,4Z)-5-hydroxy-1-(4-methoxyphenyl)-7-phenylhepta-1,4-dien-3-one (20)

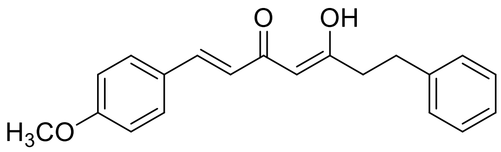

Yellow solid; yield 76%; m.p.: 80.1-81 °C. ^{1}H NMR (500 MHz, CD_3OD) δ 7.46-7.43 (m, 3H), 7.19-7.06 (m, 6H), 6.86 (d, *J* = 5 Hz, 2H), 6.40 (d, *J* = 15 Hz, 1H), 5.66 (s, 1H), 3.76 (s, 3H), 2.86 (t, 2H), 2.61 (t, 2H); ^{13}C NMR (125 MHz, CD_3OD) δ 201.1, 179.0, 163.0, 142.4, 141.0, 130.9, 129.6, 129.5, 127.3, 121.5, 115.6, 101.4, 56.0, 43.0, 32.6. HRMS $[ESI]^-$ calculated for $C_{20}H_{19}O_3$: 307.1329 $[M]^-$; found: 307.1337.

(1E,4Z)-1-(3,4-dimethoxyphenyl)-5-hydroxy-7-phenylhepta-1,4-dien-3-one (21)

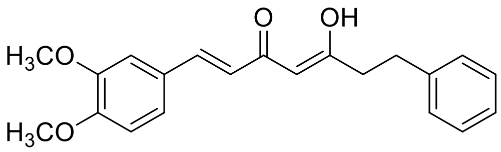

Yellow oil; yield 69.3%. ^{1}H NMR (500 MHz, CD_3OD) δ 7.43 (d, *J* = 15 Hz, 1H), 7.19-7.03 (m, 8H), 6.86 (d, *J* = 5 Hz, 1H,), 6.41 (d, *J* =15 Hz, 1H), 3.77-3.70 (m, 6H), 2.87-2.84 (m, 2H), 2.62-2.59 (m, 2H). HRMS $[ESI]^-$ calculated for $C_{21}H_{21}O_4$: 337.1434 $[M]^-$; found: 337.1437.

(1E,4Z)-5-hydroxy-7-phenyl-1-(3,4,5 trimethoxyphenyl)hepta-1,4-dien-3-one (22)

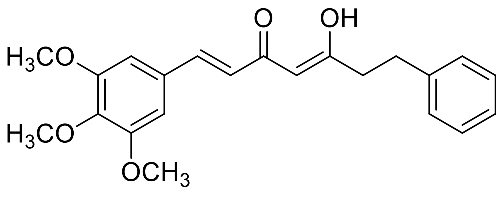

Yellow solid; yield 78%; m.p.: 82.8-83.4 °C. ^{1}H NMR (500 MHz, CD_3OD) δ 7.41 (d, *J* = 15 Hz, 1H), 7.19-7.06 (m, 5H), 6.78 (s, 2H), 6.47 (d, *J* = 15 Hz, 1H), 5.67 (s, 1H), 3.77 (s, 6H), 3.71 (s, 3H), 2.85 (t, *J* = 7.5 Hz, 2H), 2.63-2.60 (m, 2H); ^{13}C NMR (125 MHz, CD_3OD) δ 201.8, 177.9, 154.9, 142.4, 141.1, 141.0, 132.5, 129.6, 129.5, 127.3, 123.3, 106.7, 101.9, 61.3, 56.8, 43.1, 32.4. HRMS $[ESI]^-$ calculated for $C_{22}H_{23}O_5$: 367.1532 $[M]^-$; found: 367.1540.

(1E,4Z)-1-(4-fluorophenyl)-5-hydroxy-7-phenylhepta-1,4-dien-3-one (23)

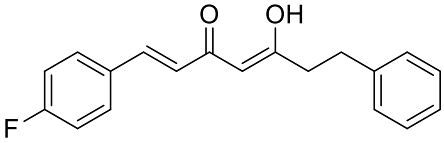

White solid; yield 62.5%; m.p.: 56-57 ºC. ^{1}H NMR (500 MHz, CD_3OD) δ 7.53-7.51 (m, 2H), 7.45 (d, *J* = 15 Hz, 1H), 7.18-7.11 (m, 5H), 7.08-7.01 (m, 3H), 6.48 (d, 1H, *J* = 15 Hz), 5.69 (s, 1H), 2.85 (t, *J* = 7.5 Hz, 2H), 2.65-2.62 (m, 2H). HRMS [ESI]$^-$ calculated for $C_{19}H_{16}O_2F$: 295.1140 [M]$^-$; found: 295.1130.

(1E,4Z)-1-(4-chlorophenyl)-5-hydroxy-7-phenylhepta-1,4-dien-3-one (24)

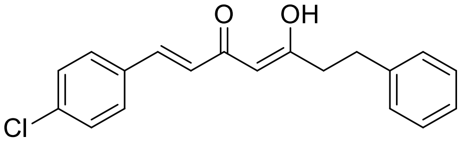

Brown solid; yield 72%; m.p.: 79.5-80.2 ºC. ^{1}H NMR (500 MHz, CD_3OD) δ 7.56-7.50 (m, 3H), 7.38-7.37 (m, 2H), 7.27-7.20 (m, 5H), 7.17-7.14 (m, 1H), 6.62 (d, *J* = 15 Hz, 1H), 5.78 (s, 1H), 2.94 (t, 2H), 2.73 (t, 2H); ^{13}C NMR (125 MHz, CD_3OD) δ 202.5, 177.0, 142.4, 139.2, 136.8, 135.5, 130.5, 130.3, 129.6, 129.5, 127.3, 124.8, 102.2, 43.3, 32.4. HRMS [ESI]$^-$ calculated for $C_{19}H_{16}O_2Cl$: 311.0833 [M]$^-$; found: 311.0843.

(E)-5-hydroxy-1-(4-hydroxyphenyl)-7-(4methoxyphenyl)hept-1-en-3-one (MD1)

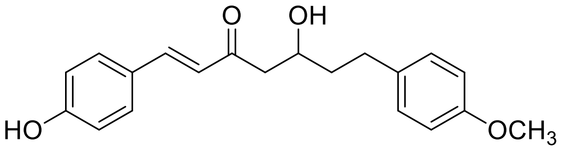

White solid; yield 70%; m.p.: 123-130 ºC. ^{1}H NMR (500 MHz, CD_3OD) δ 7.45 (d, *J* = 15 Hz, 1H), 7.38 (d, *J* = 5 Hz, 2H), 7.01 (d, *J* = 5 Hz, 2H), 6.71-6.69 (m, 4H), 6.55 (d, *J* = 15 Hz, 1H), 4.01-3.96 (m, 1H), 3.63, (s, 3H), 2.77-2.73 (m, 1H), 2.69-2.60 (m, 2H), 2.54-2.48 (m, 1H), 1.68-1.62 (m, 2H); ^{13}C NMR (125 MHz, CD_3OD) δ 202.0, 161.8, 159.5, 145.7, 135.5, 131.8, 130.5, 127.4, 124.6, 117.1, 114.9, 68.9, 55.8, 48.9, 40.6, 32.1. HRMS [ESI]$^+$ calculated for $C_{20}H_{23}O_4$: 327.1591 [M + H]$^+$; found: 327.1585.

(1E,4E)-1-(4-hydroxyphenyl)-7-(4-methoxyphenyl)hepta-1,4-dien-3-one (MD1a)

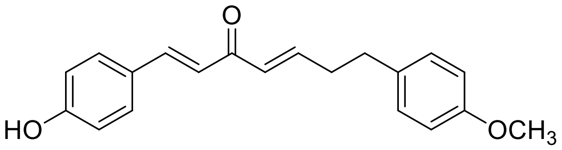

Yellow oil; yield 73%. ^{1}HNMR (500 MHz, CD_3OD) δ 7.50 (d, *J* = 15 Hz, 1H), 7.44 (d, *J* = 5 Hz, 2H), 7.05 (d, *J*=5 Hz, 2H), 6.96-6.91 (m, 1H), 6.83-6.79 (m, 1H), 6.76-6.73 (m, 4H), 6.40 (d, *J* = 15 Hz, 1H), 3.67, (s, 3H), 2.70-2.67 (m, 2H), 2.51-2.47 (m, 2H); ^{13}C NMR (125 MHz, CD_3OD) δ 192.0, 161.8, 159.7, 148.8, 145.9, 134.5, 131.8, 130.8, 130.6, 127.7, 122.8, 117.1, 115.0, 55.8, 36.0, 34.8. HRMS [ESI]$^+$ calculated for $C_{20}H_{21}O_3$: 309.1485 [M + H]$^+$; found: 309.1478.

(E)-5-hydroxy-1,7-bis(4-methoxyphenyl)hept-1-en-3-one (MD2)

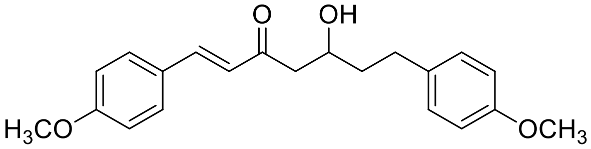

Yellow solid; yield 67.8%; m.p.: 43-44 °C. ^{1}H NMR (500 MHz, CD_3OD) δ 7.51-7.48 (m, 3H), 7.04 (d, *J* = 5 Hz, 2H), 6.88-6.86 (m, 2H), 6.73 (d, *J* = 5Hz, 2H), 6.62 (d, *J* = 15 Hz, 1H), 4.05-4.00 (m, 1H), 3.75, (s, 3H), 3.74 (s, 3H), 2.81-2.50 (m, 4H), 2.51-2.47 (m, 2H), 1.71-1.67 (m, 2H); ^{13}C NMR (125 MHz, CD_3OD) δ 202.0, 163.6, 159.4, 145.2, 135.5, 131.5, 130.5, 128.6, 125.4, 115.6, 115.0, 68.9, 56.1, 55.8, 49.01, 40.6, 32.1. HRMS [ESI]$^+$ calculated for $C_{21}H_{25}O_4$: 341.1747 [M + H]$^+$; found: 341.1744.

(1E,4E)-1,7-bis(4-methoxyphenyl)hepta-1,4-dien-3-one (MD2a)

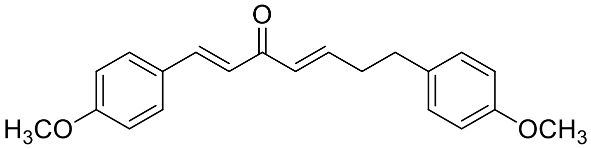

Yellow solid; yield 70.4%; m.p.: 55.5-56 °C. ^{1}H NMR (500 MHz, CD_3OD) δ 7.52-7.49 (m, 3H), 7.04 (d, *J* = 5 Hz, 2H), 6.97-6.91 (m, 1H), 6.88-6.83 (m, 3H), 6.75 (d, *J* = 5 Hz, 2H), 6.39 (d, *J* = 15 Hz, 1H), 3.74, (s, 3H), 3.66 (s, 3H), 2.69-2.66 (m, 2H), 2.50-2.46 (m, 2H); ^{13}C NMR (125 MHz, CD_3OD) δ 191.9, 163.6, 159.7, 149.0, 145.3, 134.4, 131.6, 130.8, 130.6, 128.8, 123.6, 115.7, 115.0, 56.1, 55.8, 36.0, 34.8. HRMS [ESI]$^+$ calculated for $C_{21}H_{23}O_3$: 323.1642 [M + H]$^+$; found: 323.1633.

(E)-1-(3,4-dimethoxyphenyl)-5-hydroxy-7-(4-methoxyphenyl)hept-1-en-3-one (MD3)

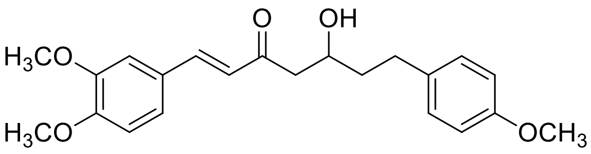

Yellow oil; yield 61%. ^{1}H NMR (500 MHz, CD_3OD) δ 7.49 (d, *J* = 15 Hz, 1H), 7.14-7.10 (m, 2H), 7.04 (d, *J* = 5 Hz, 2H), 6.89 (d, *J* = 5 Hz, 1H), 6.74 (d, *J* = 5 Hz, 2H), 6.65 (d, *J* = 15 Hz, 1H), 4.07-4.02 (m, 1H), 3.78, (s, 6H), 3.66 (s, 3H), 2.82-2.69 (m, 2H), 2.68-2.52 (m, 2H), 1.72-1.68 (m, 2H); ^{13}C NMR (125 MHz, CD_3OD) δ 201.8, 159.4, 153.2, 150.9, 145.4, 135.5, 130.5, 129.0, 125.7, 124.7, 115.0, 112.8, 111.89, 68.9, 56.6, 56.6, 55.8, 49.0, 40.6, 32.1. HRMS $[ESI]^+$ calculated for $C_{22}H_{27}O_5$: 371.1853 $[M + H]^+$; found: 371.1849.

(1E,4E)-1-(3,4-dimethoxyphenyl)-7-(4-methoxyphenyl)hepta-1,4-dien-3-one (MD3a)

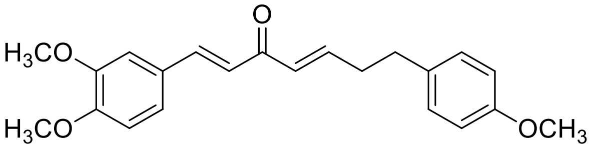

Yellow oil; yield 65.3%. ^{1}H NMR (500 MHz, CD_3OD) δ 7.49 (d, *J* = 15 Hz, 1H), 7.17-7.13 (m, 1H), 7.12-7.11 (m, 1H), 7.03 (d, *J* = 5 Hz, 2H), 6.98-6.95 (m, 1H), 6.94-6.86 (m, 2H), 6.75-6.73 (m, 2H), 6.39 (d, *J* = 15 Hz, 1H), 3.78 (s, 3H), 3.77 (s, 3H), 3.65 (s, 3H), 2.65 (t, *J* = 7.5 Hz, 2H), 2.50-2.45 (m, 2H); ^{13}C NMR (125 MHz, CD_3OD) δ 191.9, 159.7, 153.3, 151.0, 149.1, 145.6, 134.4, 130.8, 130.6, 129.3, 124.8, 123.8, 115.0, 112.8, 111.8, 56.7, 56.6, 55.8, 49.1, 36.0, 34.8. HRMS $[ESI]^+$ calculated for $C_{22}H_{25}O_4$: 353.1658 $[M + H]^+$; found: 353.1654.

(E)-5-hydroxy-7-(4-methoxyphenyl)-1-(3,4,5-trimethoxyphenyl)hept-1-en-3-one (MD4)

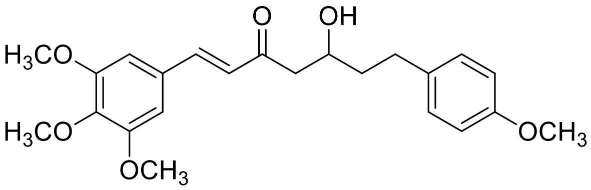

Yellow oil; yield 63.7%. ^{1}H NMR (500 MHz, CD_3OD) δ 7.46 (d, *J* = 15 Hz, 1H), 7.03 (d, *J* = 5 Hz, 2H), 6.84 (s, 2H), 6.73-6.67 (m, 3H), 4.07-4.01 (m, 1H), 3.78 (s, 6H), 3.71 (s, 3H), 3.64 (s, 3H), 2.83-2.78 (m, 1H), 2.72-2.62 (m, 2H), 2.59-2.50 (m, 1H), 1.71-1.67 (m, 2H); ^{13}C NMR (125 MHz, MeOD-d_4) δ 201.8, 159.4, 154.9, 145.2, 141.6, 135.4, 131.9, 130.5, 130.4, 127.2, 115.0, 107.2, 68.8, 61.4, 56.9, 56.6, 56.9, 55.8, 40.6, 32.1. HRMS $[ESI]^+$ calculated for $C_{23}H_{29}O_6$: 401.1884 $[M + H]^+$; found: 401.1890.

(1E,4E)-7-(4-methoxyphenyl)-1-(3,4,5-trimethoxyphenyl)hepta-1,4-dien-3-one (MD4a)

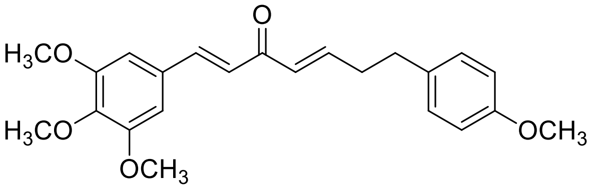

Yellow solid; yield 68.6%; m.p.: 59-60 °C. ^{1}H NMR (500 MHz, $CDCl_3$) δ 7.50 (d, *J* = 15 Hz, 1H), 7.09 (d, *J* = 5 Hz, 1H), 7.02-6.96 (m, 1H), 6.83-6.76 (m, 5H), 6.43 (d, *J* = 15 Hz, 1H), 3.88 (s, 9H), 3.76 (s, 3H), 2.77-2.74 (m, 2H), 2.57-2.53 (m, 2H); ^{13}C NMR (125 MHz, $CDCl_3$) δ 189.0, 158.0, 153.4, 146.9, 143.2, 140.3, 132.8, 130.2, 129.3, 129.3, 124.4, 113.9, 105.4, 61.0, 56.1, 55.2, 34.6, 33.6. HRMS [ESI]+ calculated for $C_{23}H_{27}O_5$: 383.1756 $[M + H]^+$; found: 383.1753.

(E)-7-(3,4-dimethoxyphenyl)-5-hydroxy-1-(4-hydroxyphenyl)hept-1-en-3-one (MD5)

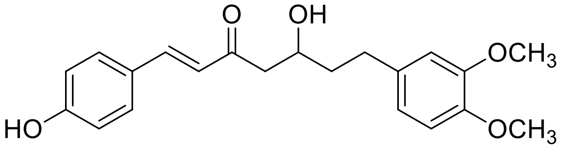

A yellowish oil; yield 60.2%. ^{1}H NMR (500 MHz, CD_3OD) δ 7.46 (d, *J* = 15 Hz, 1H), 7.38 (d, *J* = 5 Hz, 2H), 6.74-6.72 (m, 4H), 6.66-6.65 (m, 1H), 6.56 (d, *J* = 15 Hz, 1H), 4.04-3.99 (m, 1H), 3.70 (s, 3H), 3.68 (s, 3H), 2.79-2.75 (m, 1H), 2.70-2.62 (m, 2H), 2.57-2.51 (m, 1H),1.72-1.67 (m, 2H); ^{13}C NMR (125 MHz, CD_3OD) δ 202.0, 161.7, 150.4, 148.7, 145.7, 136.5, 131.7, 127.3, 124.6, 121.8, 117.1, 113.7, 113.3, 68.9, 56.7, 56.5, 48.8, 40.4, 32.5. ESI-MS (positive, m/z): 357.3 [M+H]

(1E,4E)-7-(3,4-dimethoxyphenyl)-1-(4-hydroxyphenyl)hepta-1,4-dien-3-one (MD5a)

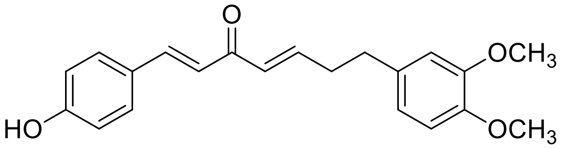

Yellow oil; yield 68%. ^{1}H NMR (500 MHz, $CDCl_3$) δ 7.53 (d, *J* = 15 Hz, 1H), 7.38 (d, *J* = 5 Hz, 2H), 6.99-6.93 (m, 1H), 6.81 (d, *J* = 5 Hz, 2H), 6.75-6.72 (m, 2H), 6.67-6.65 (m, 2H), 6.39 (d, *J* = 15 Hz, 1H), 3.78 (s, 3H), 3.74 (s, 3H), 2.71-2.68 (m, 2H), 2.53-2.49 (m, 2H); ^{13}C NMR (125 MHz, $CDCl_3$) δ 189.8, 158.7, 148.8, 147.3, 147.0, 143.9, 133.4, 130.3, 129.5, 126.9, 122.2, 120.2, 116.0, 111.7, 111.3, 55.9, 55.8, 34.6, 34.1. HRMS [ESI]+ calculated for $C_{21}H_{23}O_4$: 339.1591 $[M + H]^+$; found: 339.1584.

(E)-7-(3,4-dimethoxyphenyl)-5-hydroxy-1-(4-methoxyphenyl)hept-1-en-3-one (MD6)

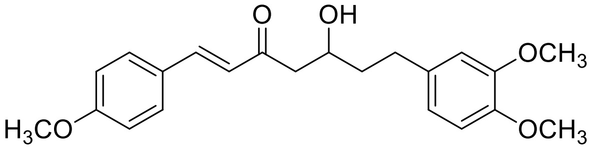

White solid; yield 61.3%; m.p.: 50-51 °C. ^{1}H NMR (500 MHz, CD_3OD) δ 7.50-7.47 (m, 3H), 6.86

(m, 2H), 6.75-6.73 (m, 2H), 6.67-6.59 (m, 2H), 4.02 (brs, 1H), 3.73 (s, 3H), 3.71 (s, 3H), 3.68 (s, 3H), 2.81-2.76 (m, 1H), 2.71-2.63 (m, 2H), 2.57-2.51 (m, 1H), 1.70 (brs, 2H); ^{13}C NMR (125 MHz, CD_3OD) δ 201.9, 163.6, 150.5, 148.8, 145.2, 136.5, 131.5, 128.6, 125.5, 121.9, 115.7, 113.7, 113.4, 68.9, 56.7, 56.6, 56.1, 49.02, 40.4, 32.5. HRMS $[ESI]^+$ calculated for $C_{22}H_{27}O_5$: 371.1853 $[M + H]^+$; found: 371.1846..

(1E,4E)-7-(3,4-dimethoxyphenyl)-1-(4-methoxyphenyl)hepta-1,4-dien-3-one (MD6a)

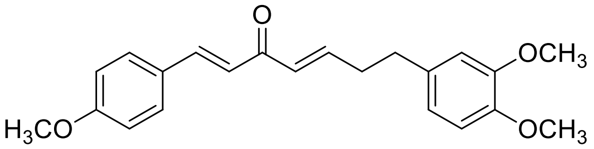

Yellow oil; yield 72.5%. ^{1}H NMR (500 MHz, CD_3OD) δ 7.52-7.49 (m, 3H), 6.97-6.91 (m, 1H), 6.87-6.83 (m, 3H), 6.77-6.73 (m, 2H), 6.66 (d, J = 5 Hz, 1H), 6.39 (d, J = 15 Hz, 1H), 3.73 (s, 3H), 3.70 (s, 3H), 3.68 (s, 3H), 2.69-2.66 (m, 2H), 2.51-2.47 (m, 2H); ^{13}C NMR (125 MHz, CD_3OD) δ 191.9, 163.6, 150.5, 149.0, 145.4, 135.5, 131.6, 130.8, 128.8, 123.6, 121.9, 115.7, 113.8, 113.3, 56.7, 56.6, 56.1, 35.9, 35.3. HRMS $[ESI]^+$ calculated for $C_{22}H_{25}O_4$: 353.1747 $[M + H]^+$; found: 353.1738.

(E)-1,7-bis(3,4-dimethoxyphenyl)-5-hydroxyhept-1-en-3-one (MD7)
Reference compound [12]

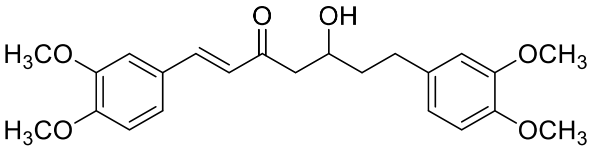

Known compound; yellow solid; yield 66.1%; m.p.: 87-88 ^{o}C. ^{1}H NMR (500 MHz, CD_3OD) δ 7.47 (d, J = 15 Hz, 1H), 7.12-7.08 (m, 2H), 6.87 (d, J = 5 Hz, 1H), 6.74-6.72 (m, 2H), 6.66-6.61 (m, 2H), 4.04-4.00 (m, 1H), 3.76 (s, 6H), 3.70 (s, 3H), 3.67 (s, 3H), 2.80-2.76 (m, 1H), 2.71-2.62 (m, 2H), 2.59-2.50 (m, 1H), 1.72-1.66 (m, 2H); ^{13}C NMR (125 MHz, CD_3OD) δ 201.9, 153.3, 151.0, 150.5, 148.8, 145.4, 136.5, 129.1, 125.7, 124.7, 121.9, 113.7, 113.4, 112.8, 111.8, 68.9, 56.7, 56.6, 56.5, 49.07, 40.5, 32.5. HRMS $[ESI]^+$ calculated for $C_{23}H_{29}O_6$: 401.1959 $[M + H]^+$; found: 401.1948.

(1E,4E)-1,7-bis(3,4-dimethoxyphenyl)hepta-1,4-dien-3-one (MD7a)

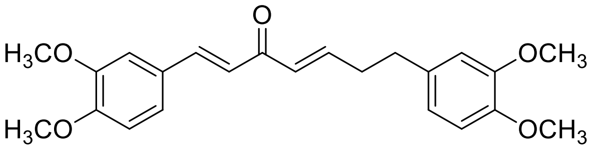

Yellow oil; yield 70.5%. ^{1}H NMR (500 MHz, CD_3OD) δ 7.51 (d, J = 15 Hz, 1H), 7.18 (s, 1H), 7.13 (d, J = 5 Hz, 1H), 6.99-6.94 (m, 1H), 6.90-6.87 (m, 2H), 6.78-6.74 (m, 2H), 6.68 (d, J = 5 Hz, 1H), 6.41 (d, J = 15 Hz, 1H), 3.79(s, 3H), 3.78 (s, 3H), 3.72 (s, 3H), 3.70 (s, 3H), 2.69 (t, J =7.5 Hz, 2H),

2.55-2.49 (m, 2H); ^{13}C NMR (125 MHz, CD_3OD) δ 191.8, 153.3, 151.0, 150.6, 149.1, 149.0, 145.6, 135.5, 130.9, 129.3, 124.8, 123.9, 121.9, 113.8, 113.3, 112.8, 111.9, 56.7, 56.6, 35.9, 35.2. HRMS [ESI]$^+$ calculated for $C_{23}H_{26}O_5$: 382.1780 [M + H]$^+$; found: 382.1779.

(E)-7-(3,4-dimethoxyphenyl)-5-hydroxy-1-(3,4,5-trimethoxyphenyl)hept-1-en-3-one (MD8)

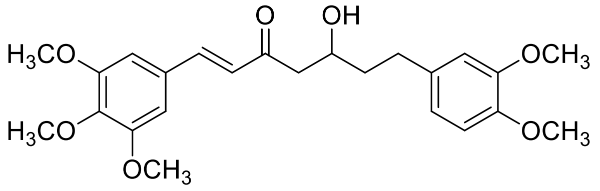

Colorless oil; yield 62%. ^{1}H NMR (500 MHz, CD_3OD) δ 7.47 (d, *J* = 15 Hz, 1H), 6.86 (s, 2H), 6.76-6.67 (m, 4H), 4.08-4.00 (m, 1H), 3.79 (s, 3H), 3.73 (s, 3H), 3.69 (s, 9H) , 2.84-2.80 (m, 1H), 2.75-2.65 (m, 2H), 2.61-2.53 (m, 1H), 1.75-1.70 (m, 2H); ^{13}C NMR (125 MHz, CD_3OD) δ 201.7, 154.9, 150.5, 148.8, 145.1, 141.7, 136.5, 131.8, 127.2, 121.8, 113.8, 113.4, 107.2, 68.8, 61.3, 56.9, 56.7, 56.5, 49.1, 40.4, 32.5. HRMS [ESI]$^+$ calculated for $C_{24}H_{31}O_7$: 431.2064 [M + H]$^+$; found: 431.2055.

(1E,4E)-7-(3,4-dimethoxyphenyl)-1-(3,4,5-trimethoxyphenyl)hepta-1,4-dien-3-one (MD8a)

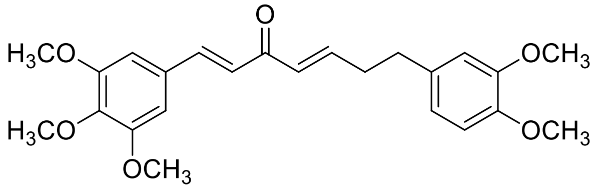

Yellow solid; yield 65.6%; m.p.: 86-87 °C. ^{1}H NMR (500 MHz, CD_3OD) δ 7.46 (d, *J* = 15 Hz, 1H), 7.01-6.92 (m, 2H), 6.85 (s, 2H), 6.75-6.72 (m, 2H), 6.64 (m, 1H), 6.39 (d, *J* = 15 Hz, 1H), 3.77 (s, 6H), 3.71(s, 3H), 3.70 (s, 3H), 3.69 (s, 3H), 2.67-2.64 (m, 2H), 2.49-2.45 (m, 2H); ^{13}C NMR (125 MHz, CD_3OD) δ 191.6, 154.9, 150.5, 149.3, 149.0, 145.3, 141.7, 135.4, 132.0, 130.8, 125.3, 121.9, 113.7, 113.3, 107.2, 61.3, 56.8, 56.6, 56.5, 35.8, 35.2. HRMS [ESI]$^+$ calculated for $C_{24}H_{29}O_6$: 413.1959 [M + H]$^+$; found: 413.1954.

(E)-5-hydroxy-1-(4-hydroxyphenyl)-7-(3,4,5-trimethoxyphenyl)hept-1-en-3-one (MD9)

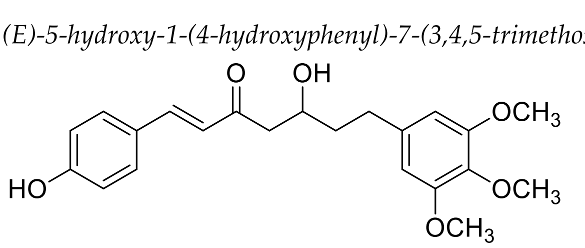

Yellow oil; yield 73 %. ^{1}H NMR (500 MHz, CD_3OD) δ 7.50-7.39 (m, 3H), 6.73 (d, *J* = 5 Hz, 2H), 6.58 (d, *J* = 15 Hz, 1H), 6.43 (s, 2H), 4.05-4.00 (m, 1H), 3.71 (s, 6H), 3.63 (s, 3H), 2.81-2.77 (m, 1H), 2.72-2.64 (m, 2H), 2.58-2.52 (m, 1H), 1,75-1.67 (m, 2H); ^{13}C NMR (125 MHz, CD_3OD) δ 202.0, 161.8, 154.5, 145.7, 139.8, 137.3, 131.7, 127.3, 124.6, 117.1, 106.9, 68.9, 61.3, 56.7, 48.9, 40.2, 33.3. HRMS [ESI]$^+$ calculated for $C_{22}H_{27}O_6$: 387.1802 [M + H]$^+$; found: 387.1792.

(1E,4E)-1-(4-hydroxyphenyl)-7-(3,4,5-trimethoxyphenyl)hepta-1,4-dien-3-one (MD9a)

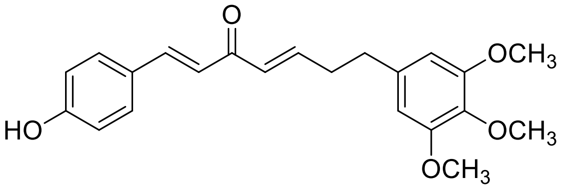

Yellow oil; yield 77 %. ^{1}H NMR (500 MHz, $CDCl_3$) δ 7.58 (d, *J* = 15 Hz, 1H), 7.44 (d, *J* = 5 Hz, 2H), 7.03-6.97 (m, 1H), 6.85 (d, *J* = 5 Hz, 2H), 6.79 (d, *J* = 15 Hz, 1H), 6.45 (d, *J* = 15 Hz, 1H), 6.39-6.37 (m, 2H), 3.82 (s, 6H), 3.81 (s, 3H), 2.76-2.73 (m, 2H), 2.59-2.55 (m, 2H); ^{13}C NMR (125 MHz, $CDCl_3$) δ 189.5, 158.3, 153.1, 146.5, 143.5, 136.6, 136.3, 130.3, 129.6, 127.2, 122.4, 116.0, 105.3, 60.8, 56.0, 34.9, 34.6. HRMS [ESI]$^+$ calculated for $C_{22}H_{25}O_5$: 369.1697 [M + H]$^+$; found: 369.1687.

(E)-5-hydroxy-1-(4-methoxyphenyl)-7-(3,4,5-trimethoxyphenyl)hept-1-en-3-one (MD10)

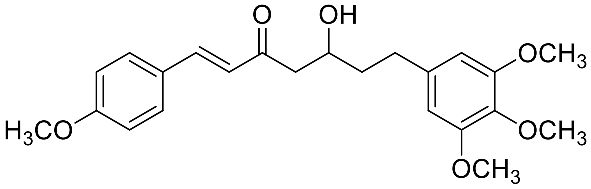

Yellow oil; yield 64 %. ^{1}H NMR (500 MHz, CD_3OD δ 7.53-7.49 (m, 3H), 6.88 (d, *J* = 5 Hz, 2H), 6.64 (d, *J* = 15 Hz, 1H), 6.44 (s, 2H), 4.08-4.00 (m, 1H), 3.75 (s, 3H), 3.72 (m, 6H), 3.63 (s, 3H), 2.84-2.80 (m, 1H), 2.79-2.65 (m, 2H), 2.60-2.54 (m, 1H), 1.79-1.73 (m, 2H); ^{13}C NMR (125 MHz, CD_3OD) δ 201.9, 163.6, 154.5, 145.2, 139.8, 137.3, 133.4, 131.5, 128.6, 125.5, 115.7, 106.9, 68.8, 61.3, 56.7, 56.1, 49.0, 40.3, 33.3. HRMS [ESI]$^+$ calculated for $C_{23}H_{29}O_6$: 401.1959 [M + H]$^+$; found: 401.1956.

(1E,4E)-1-(4-methoxyphenyl)-7-(3,4,5-trimethoxyphenyl)hepta-1,4-dien-3-one (MD10a)

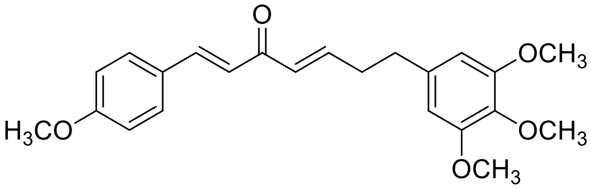

Yellow oil; yield 65.7%. ^{1}H NMR (500 MHz, $CDCl_3$) δ 7.58 (d, *J* = 15 Hz, 1H), 7.50 (d, *J* = 5 Hz, 2H), 7.02-6.96 (m, 1H), 6.91-6.88 (m, 2H), 6.80 (d, *J* = 15 Hz, 1H), 6.44 (d, *J*=15 Hz, 1H), 6.39 (s, 2H), 3.82 (s, 9H), 3.80 (s, 3H), 2.76-2.73 (m, 2H), 2.59-2.55 (m, 2H); ^{13}C NMR (125 MHz, $CDCl_3$) δ 189.1, 161.6, 153.1, 146.1, 143.1, 136.6, 130.0, 129.7, 127.4, 122.7, 114.4, 105.3, 60.8, 56.0, 55.3, 34.9, 34.4. HRMS [ESI]$^+$ calculated for $C_{23}H_{27}O_5$: 383.1853 [M + H]$^+$; found: 383.1844.

(E)-1-(3,4-dimethoxyphenyl)-5-hydroxy-7-(3,4,5-trimethoxyphenyl) hept-1-en-3-one (MD11)

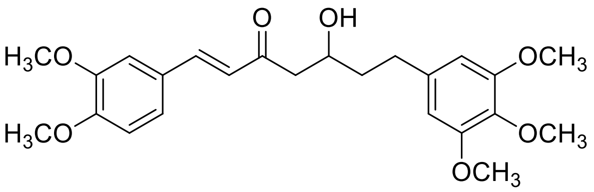

Yellow oil; yield 62.4%. ^{1}H NMR (500 MHz, CD_3OD) δ 7.49 (d, *J* = 15 Hz, 1H), 7.14-7.10 (m, 2H), 6.88 (d, *J* = 5 Hz, 1H), 6.66-6.63 (m, 1H), 6.44-6.40 (m, 2H), 4.05-4.03 (m, 1H), 3.77-3.70 (m, 12H), 3.63 (s, 3H), 2.83-2.79 (m, 1H), 2.74-2.66 (m, 2H), 2.58-2.56 (m, 1H), 1.74-1.71 (m, 2H); ^{13}C NMR (125 MHz, CD_3OD) δ 201.9, 154.5, 153.3, 150.9, 145.4, 139.8, 137.3, 129.0, 125.7, 124.7, 112.8, 111.9, 106.9, 68.8, 61.3, 56.7, 56.6, 49.05, 40.3, 33.3. HRMS [ESI]$^+$ calculated for $C_{24}H_{31}O_7$: 431.2064 [M + H]$^+$; found: 431.2054.

(1E,4E)-1-(3,4-dimethoxyphenyl)-7-(3,4,5-trimethoxyphenyl)hepta-1,4-dien-3-one (MD11a)

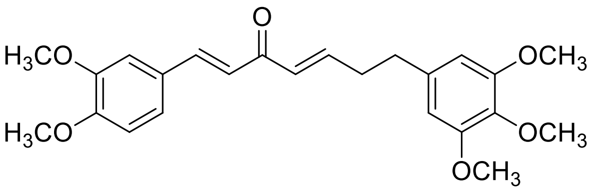

Yellow oil; yield 67.5%. ^{1}H NMR (500 MHz, $CDCl_3$) δ 7.55 (d, *J* = 15 Hz, 1H), 7.14-7.12 (m, 1H), 7.06 (s, 1H), 7.02-6.98 (m, 1H), 6.85 (d, *J* = 5 Hz, 1H), 6.78 (d, *J* = 15 Hz, 1H), 6.46 (d, *J* = 15 Hz, 1H), 6.39 (s, 2H), 3.90 (s, 6H), 3.82 (s, 6H), 3.80 (s, 3H), 2.76-2.73 (m, 2H), 2.59-2.55 (m, 2H); ^{13}C NMR (125 MHz, $CDCl_3$) δ 189.0, 153.1, 151.3, 149.2, 146.1, 143.3, 136.6, 129.4, 127.6, 123.1, 123.0, 111.0, 109.8, 105.3, 60.8, 56.0, 55.9, 55.9, 34.9, 34.4. HRMS [ESI]$^+$ calculated for $C_{24}H_{29}O_6$: 413.1959 [M + H]$^+$; found: 413.1951.

(E)-5-hydroxy-1,7-bis(3,4,5-trimethoxyphenyl)hept-1-en-3-one (MD12)

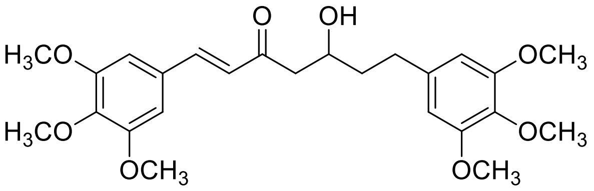

Yellow oil; yield 69.1%. ^{1}H NMR (500 MHz, CD_3OD) δ 7.48 (d, *J* = 15 Hz, 1H), 6.86 (s, 2H), 6.71 (d, *J* = 15 Hz, 1H), 6.45 (s, 2H), 4.07-4.05 (m, 1H), 3.79 (s, 6H), 3.73 (s, 9H), 3.64 (s, 3H), 2.85-2.81 (m, 1H), 2.76-2.66 (m, 2H), 2.62-2.54 (m, 1H), 1.77-1.70 (m, 2H); ^{13}C NMR (125 MHz, CD_3OD) δ 201.7, 155.0, 154.5, 145.1, 141.7, 139.8, 137.3, 131.9, 127.2, 107.2, 106.9, 68.7, 61.3, 61.2, 56.9, 56.7, 49.1, 40.3, 33.6. HRMS [ESI]$^+$ calculated for $C_{25}H_{33}O_8$: 461.2170 [M + H]$^+$; found: 461.2161.

(1E,4E)-1,7-bis(3,4,5-trimethoxyphenyl)hepta-1,4-dien-3-one (MD12a)

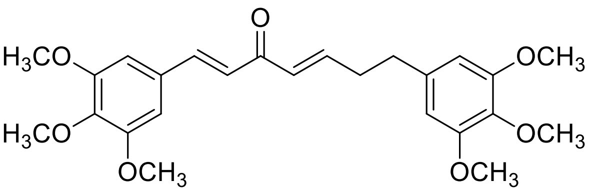

Yellow solid; yield 66.5%; m.p.: 98-99 ^{o}C. ^{1}H NMR (500 MHz, $CDCl_3$) δ 7.51 (d, *J* = 15 Hz, 1H), 7.04-6.98 (m, 1H), 6.81-6.77 (m, 2H), 6.46 (d, *J* = 15 Hz, 1H), 6.39 (s, 2H), 3.88 (s, 6H), 3.86 (s, 3H), 3.82 (s, 6H), 3.80 (m, 3H), 2.77-2.74 (m, 2H), 2.60-2.56 (m, 2H); ^{13}C NMR (125 MHz, $CDCl_3$) δ 188.9, 153.4, 153.2, 146.5, 143.3, 140.3, 136.5, 136.4, 130.1, 129.3, 124.5, 105.5, 105.3, 60.9, 60.8, 56.1, 56.0, 55.9, 34.9, 34.4. HRMS [ESI]$^+$ calculated for $C_{25}H_{31}O_7$: 443.2064 [M + H]$^+$; found: 443.2058.

(1E,4Z)-5-hydroxy-7-(4-methoxyphenyl)-1-(3,4,5-trimethoxyphenyl)hepta-1,4-dien-3-one (MD13)

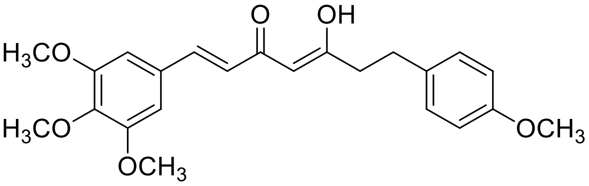

Yellow oil; yield 69.3%; m.p.: 98-99 ^{o}C. ^{1}H NMR (500 MHz, DMSO-d_6) δ 7.48 (d, *J* = 15 Hz, 1H), 7.15-7.14 (m, 2H), 7.02 (s, 2H), 6.84-6.78 (m, 3H), 5.90 (s, 1H), 5.73 (s, 1H), 3.81 (s, 6H), 3.70 (s, 6H), 2.83 (brs, 2H), 2.71-2.70 (m, 2H); ^{13}C NMR (125 MHz, DMSO-d_6) δ 200.2, 176.4, 157.6, 153.1, 139.5, 132.6, 130.4, 129.2, 122.3, 113.8, 105.7, 100.7, 60.1, 56.0, 55.0, 41.4, 29.63. HRMS [ESI]$^+$ calculated for $C_{23}H_{27}O_6$: 399.1802 [M + H]$^+$; found: 399.1791.

NMR Spectra ^{1}H and ^{13}C

Compound 1

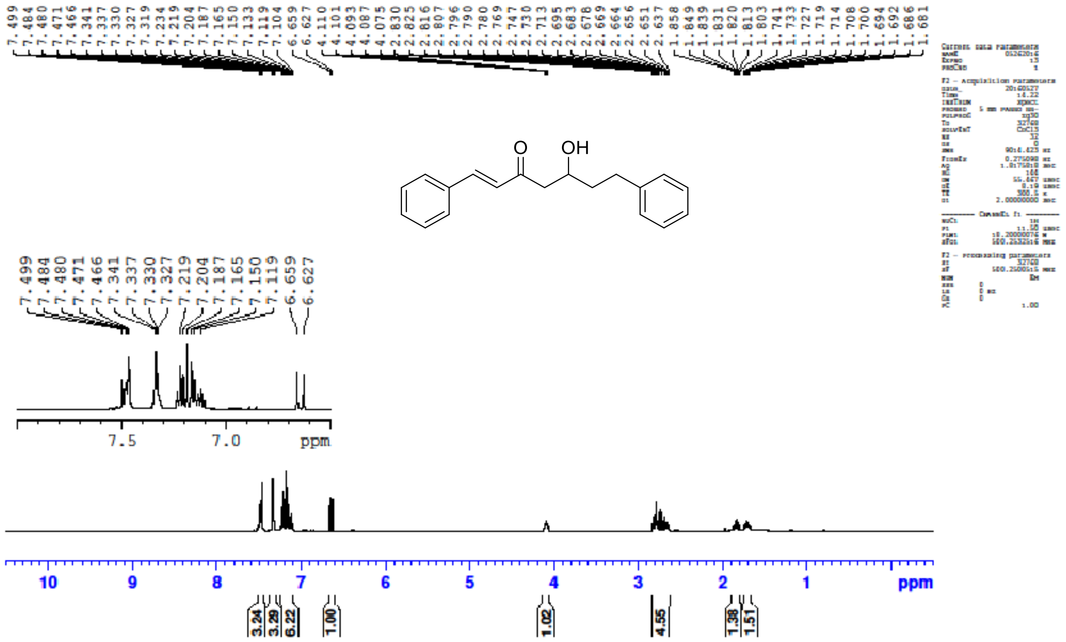

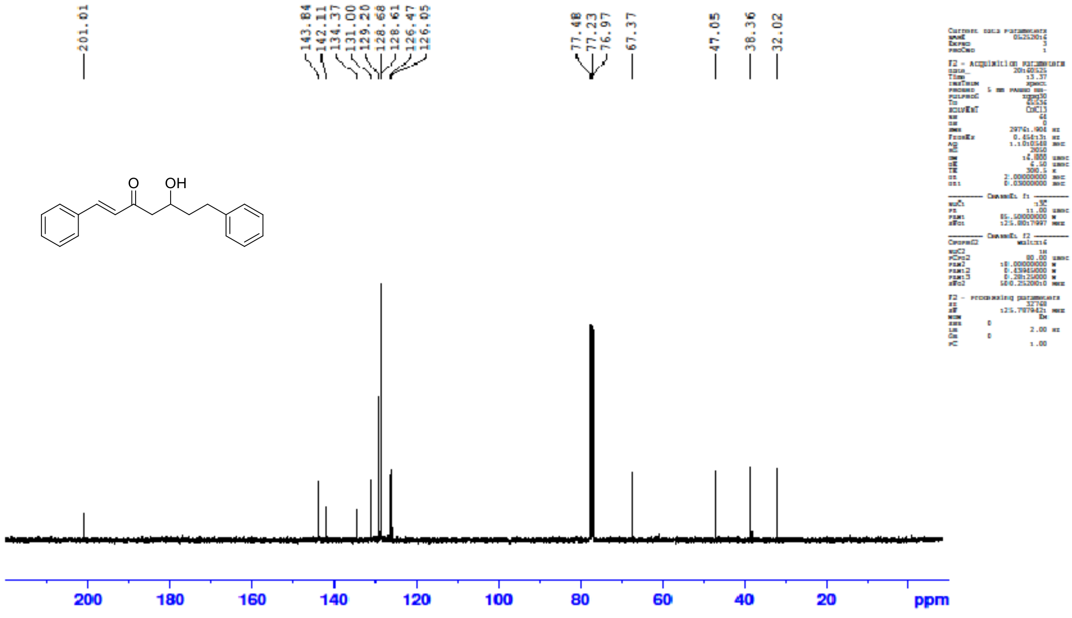

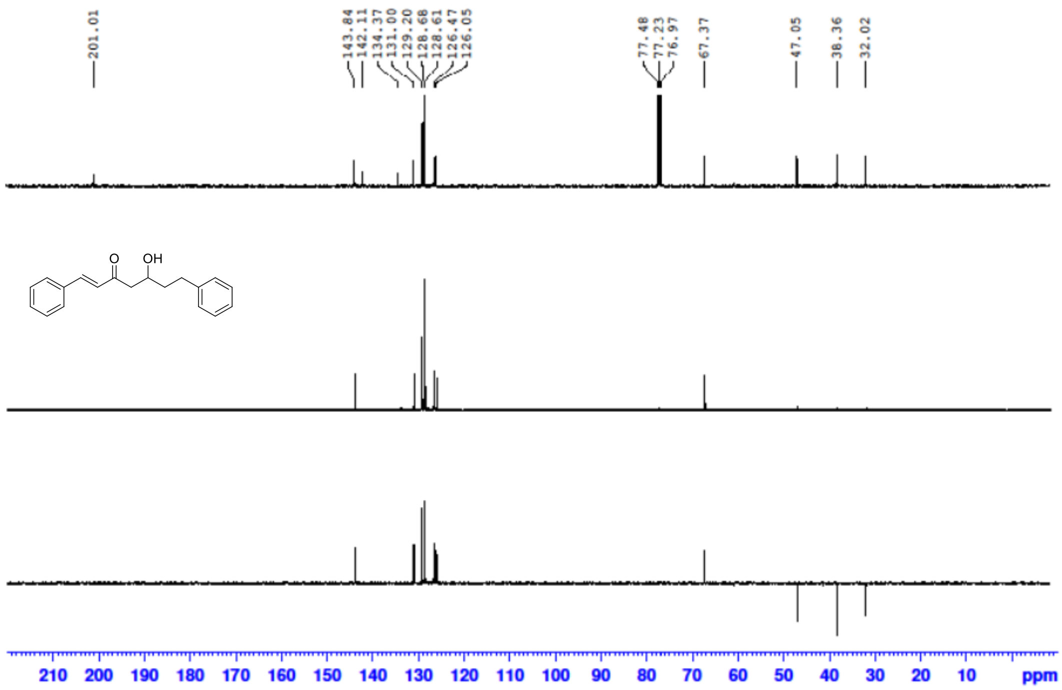

Compound 1a

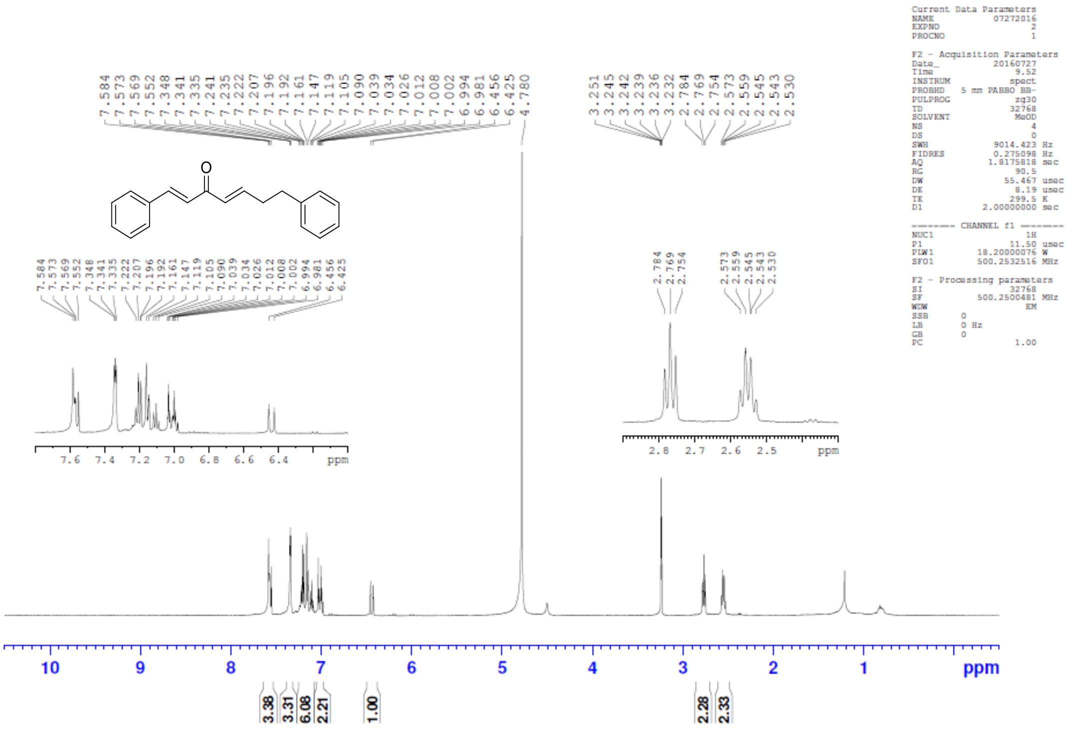

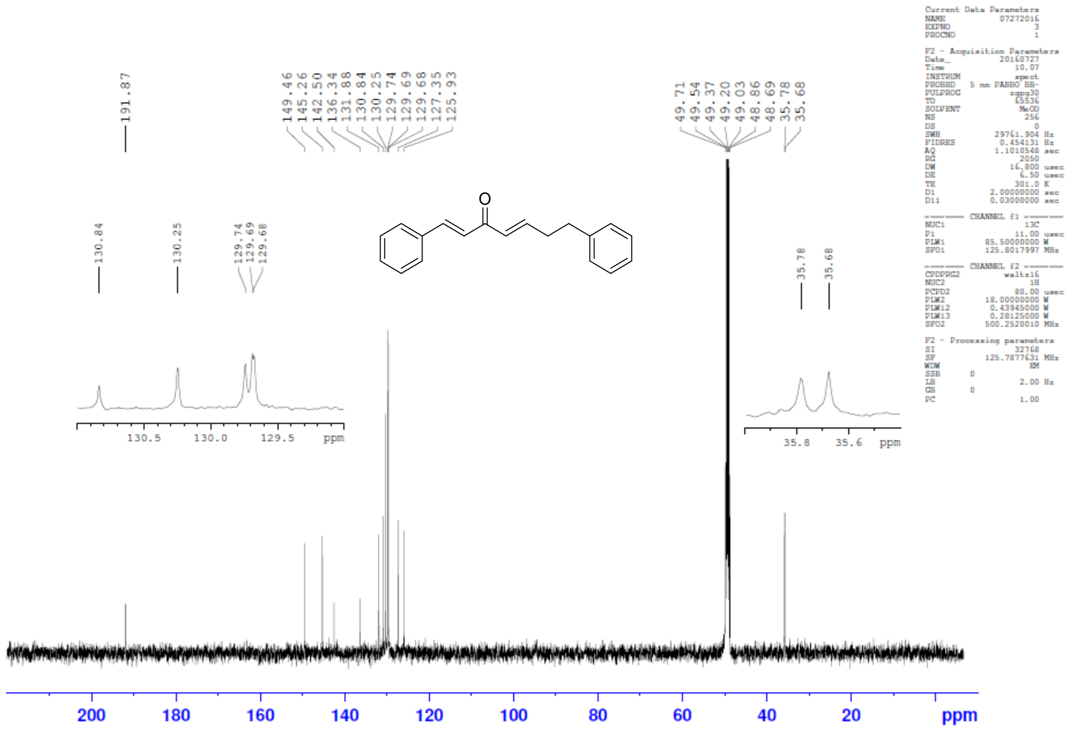
191.87
149.46 145.26 142.50 136.34 131.88 130.84 130.25 129.74 129.69 129.68 127.35 125.93
49.71 49.54 49.37 49.20 49.03 48.86 48.69 35.78 35.68
130.84
130.25
129.74 129.69 129.68
130.5 130.0 129.5 ppm
35.78
35.68
35.8 35.6 ppm
O
200 180 160 140 120 100 80 60 40 20 ppm

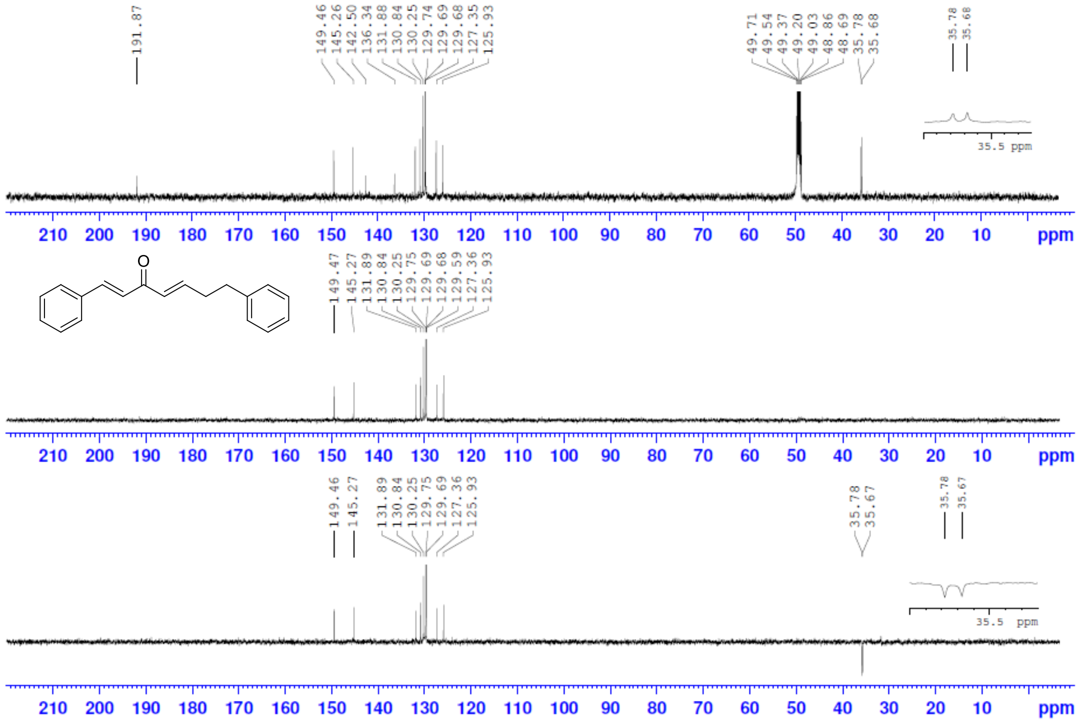
191.87
149.46 145.26 142.50 136.34 131.88 130.84 130.25 129.74 129.69 129.68 127.35 125.93
49.71 49.54 49.37 49.20 49.03 48.86 48.69 35.78 35.68
35.78 35.68
35.5 ppm
210 200 190 180 170 160 150 140 130 120 110 100 90 80 70 60 50 40 30 20 10 ppm
O
149.47 145.27 131.89 130.84 130.25 129.75 129.69 129.68 129.59 127.36 125.93
210 200 190 180 170 160 150 140 130 120 110 100 90 80 70 60 50 40 30 20 10 ppm
149.46 145.27 131.89 130.84 130.25 129.75 129.69 127.36 125.93
35.78 35.67
35.78 35.67
35.5 ppm
210 200 190 180 170 160 150 140 130 120 110 100 90 80 70 60 50 40 30 20 10 ppm

Compound 2

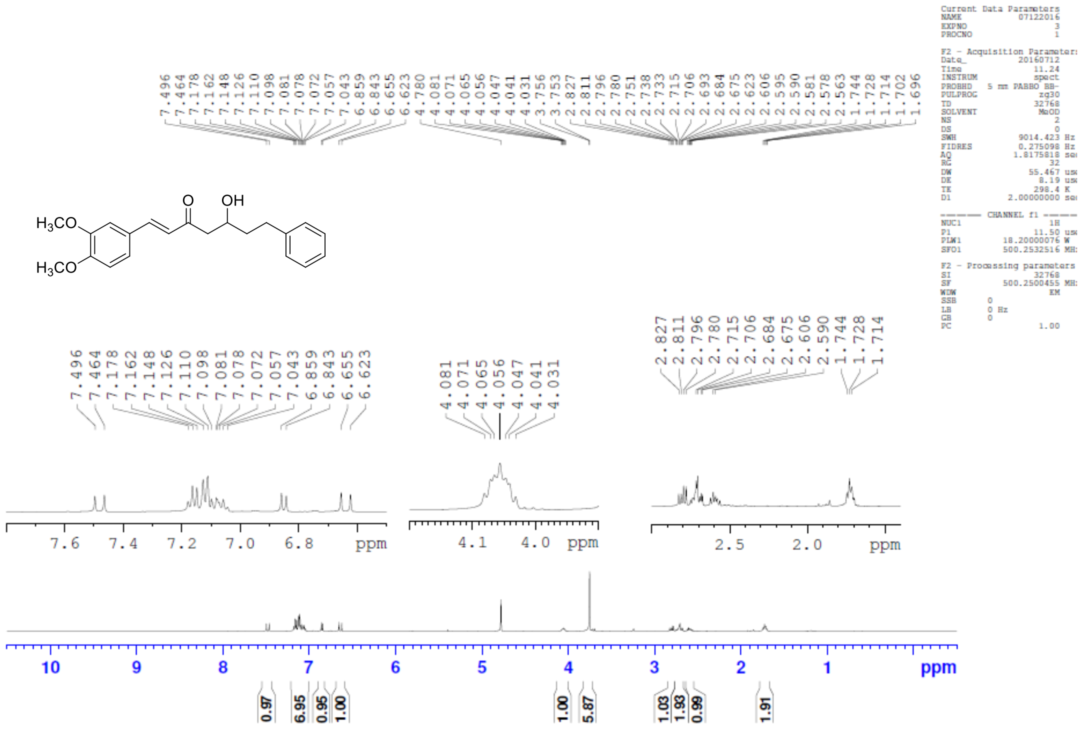
7.496 7.464 7.178 7.162 7.148 7.126 7.110 7.098 7.081 7.078 7.072 7.057 7.043 6.859 6.843 6.655 6.623 4.780 4.081 4.071 4.065 4.056 4.047 4.041 4.031 3.756 3.753 2.827 2.811 2.796 2.780 2.751 2.738 2.733 2.715 2.706 2.693 2.684 2.675 2.623 2.606 2.595 2.590 2.581 2.578 2.563 1.744 1.728 1.714 1.702 1.696
H_3CO
H_3CO
O
OH
0.97
6.95
0.95
1.00
1.00
5.87
1.03
1.93
0.99
1.91
ppm

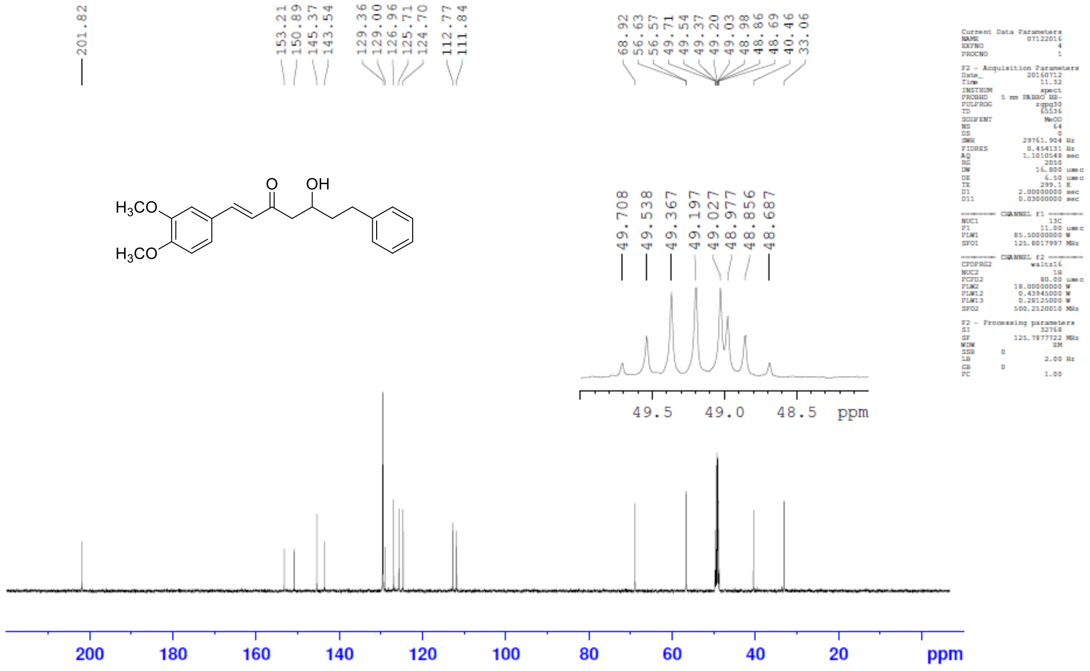
201.82
153.21 150.89 145.37 143.54
129.36 129.00 126.96 125.71 124.70
112.77 111.84
68.92 56.63 56.57 49.71 49.54 49.37 49.20 49.03 48.86 48.69 40.46 33.06
H_3CO
H_3CO
O
OH
49.708 49.538 49.367 49.197 49.027 48.977 48.856 48.687
ppm

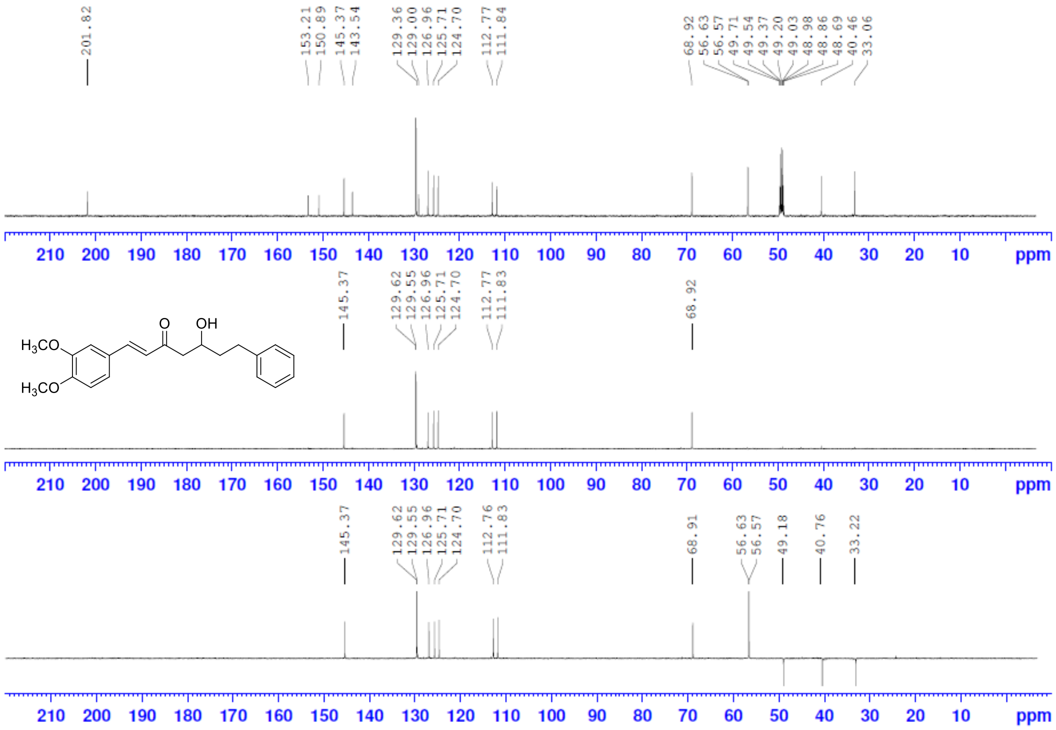

Compound 3

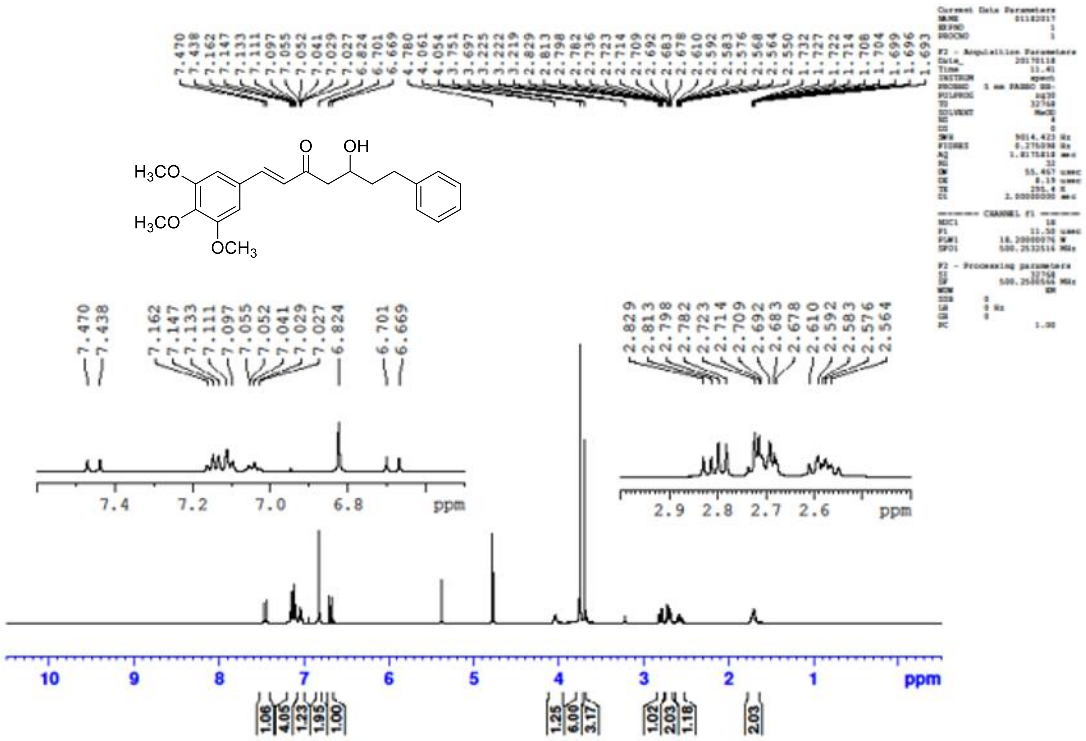

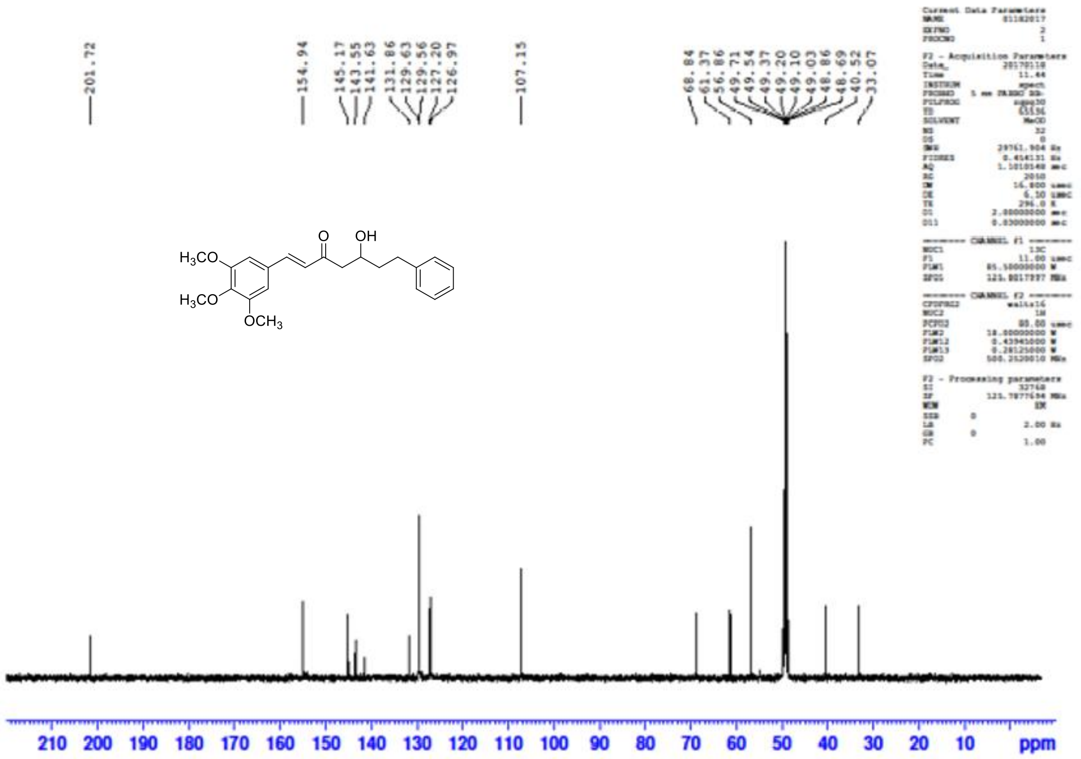

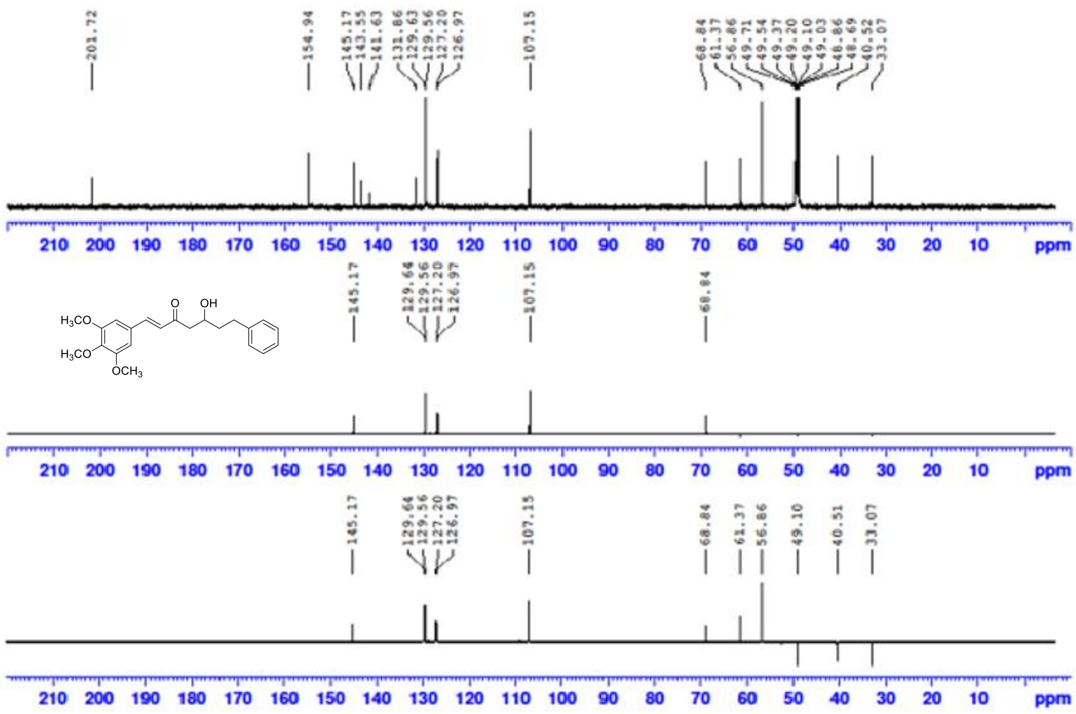

Compound 3a

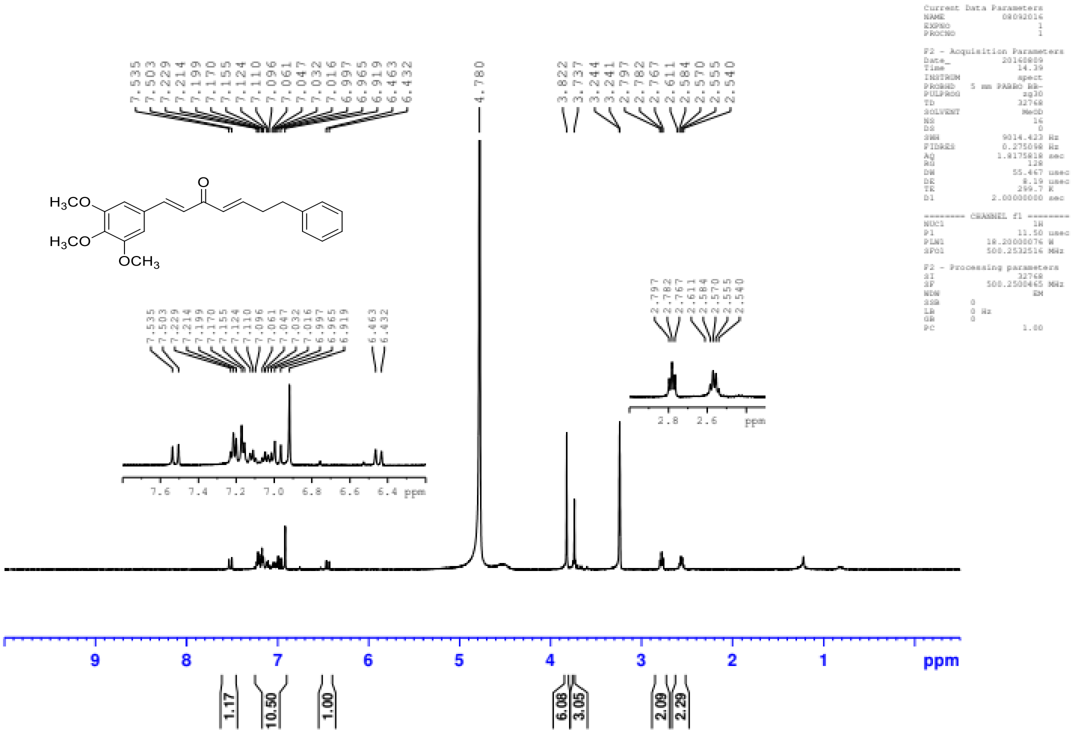
7.535
7.503
7.229
7.214
7.199
7.170
7.155
7.124
7.110
7.096
7.061
7.047
7.032
7.016
6.997
6.965
6.919
6.463
6.432
4.780
3.822
3.737
3.244
3.241
2.797
2.782
2.767
2.611
2.584
2.570
2.555
2.540
H_3CO
H_3CO
OCH_3
O
7.6 7.4 7.2 7.0 6.8 6.6 6.4 ppm
2.8 2.6 ppm
9 8 7 6 5 4 3 2 1 ppm
1.17
10.50
1.00
6.08
3.05
2.09
2.29

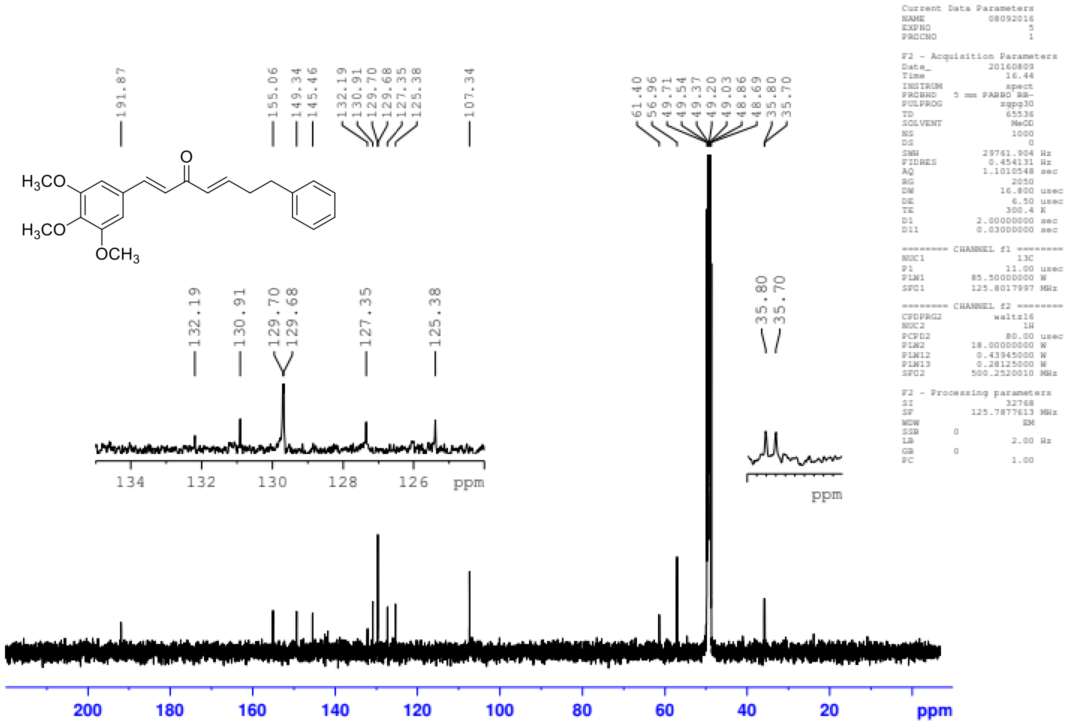
191.87
155.06
149.34
145.46
132.19
130.91
129.70
129.68
127.35
125.38
107.34
61.40
56.96
49.71
49.54
49.37
49.20
49.03
48.86
48.69
35.80
35.70
H_3CO
H_3CO
OCH_3
O
134 132 130 128 126 ppm
ppm
200 180 160 140 120 100 80 60 40 20 ppm

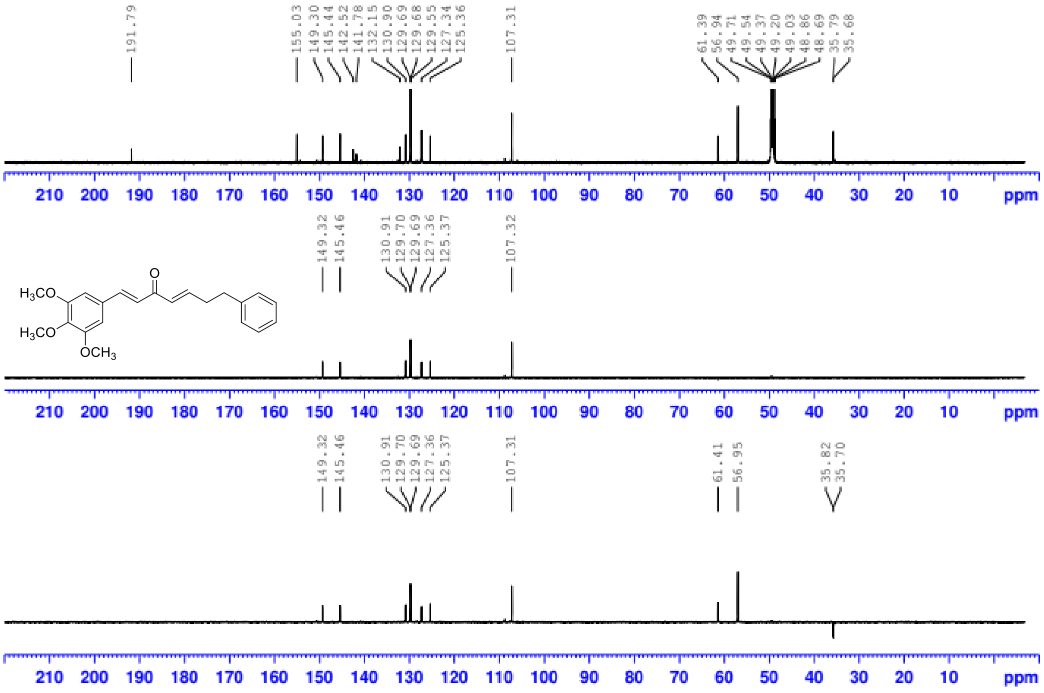

Compound 4

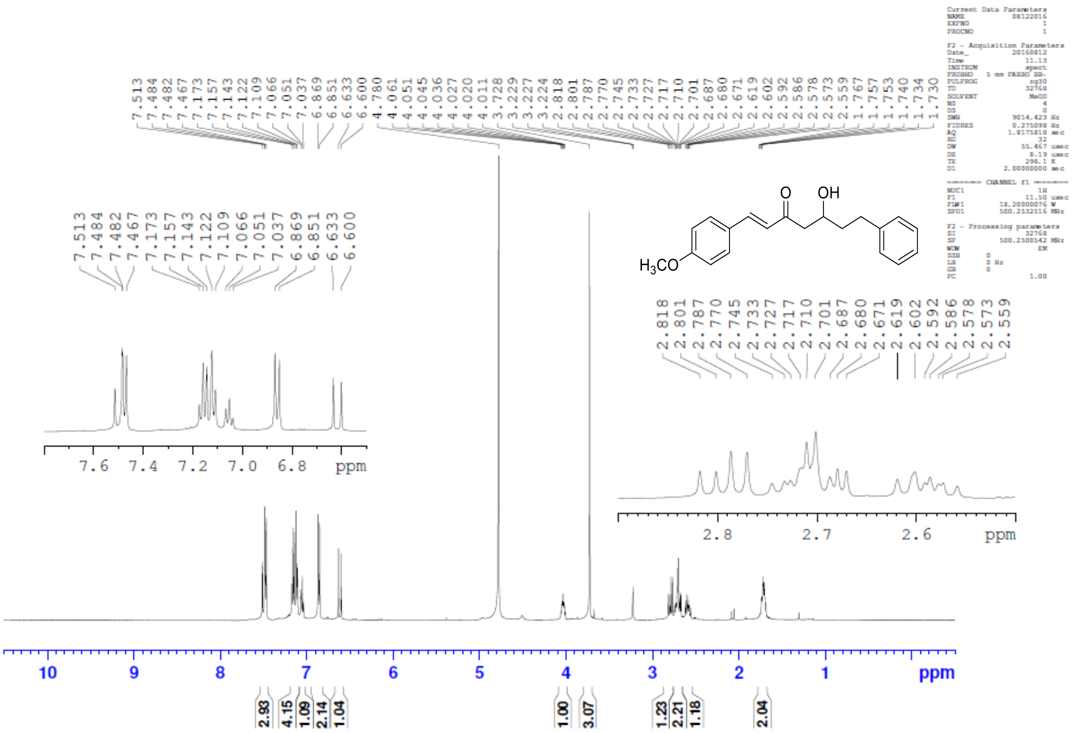

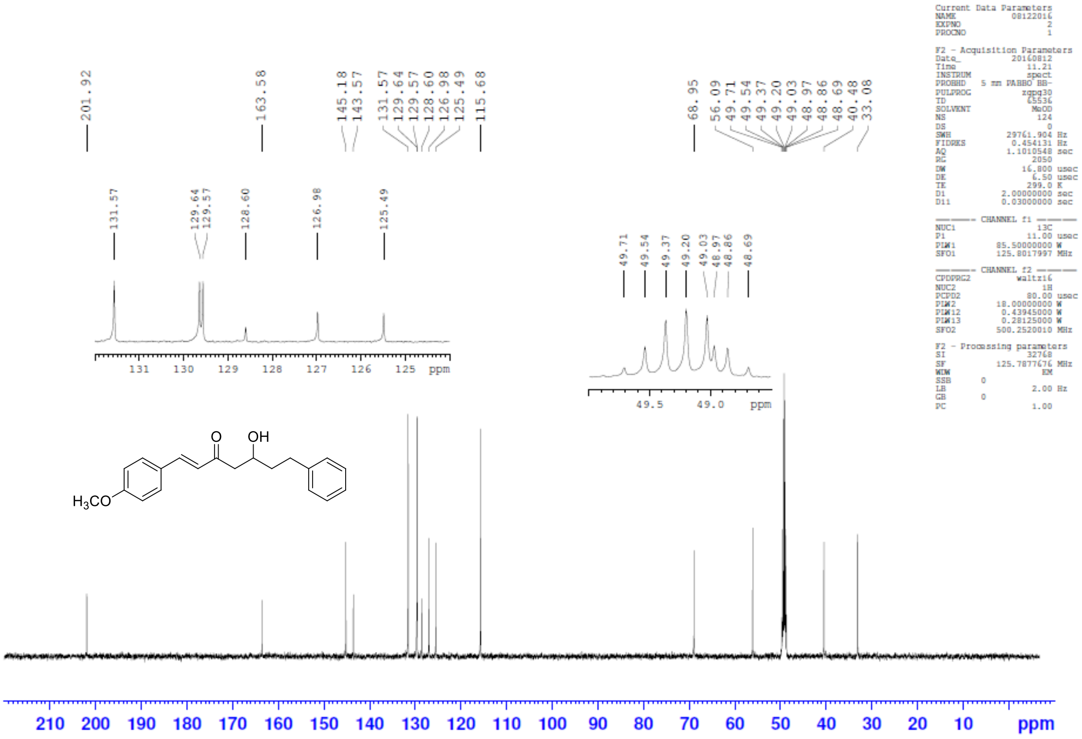
201.92
163.58
145.18
143.57
131.57
129.64
129.57
128.60
126.98
125.49
115.68
68.95
56.09
49.71
49.54
49.37
49.20
49.03
48.97
48.86
48.69
40.48
33.08
OH
O
H3CO
ppm

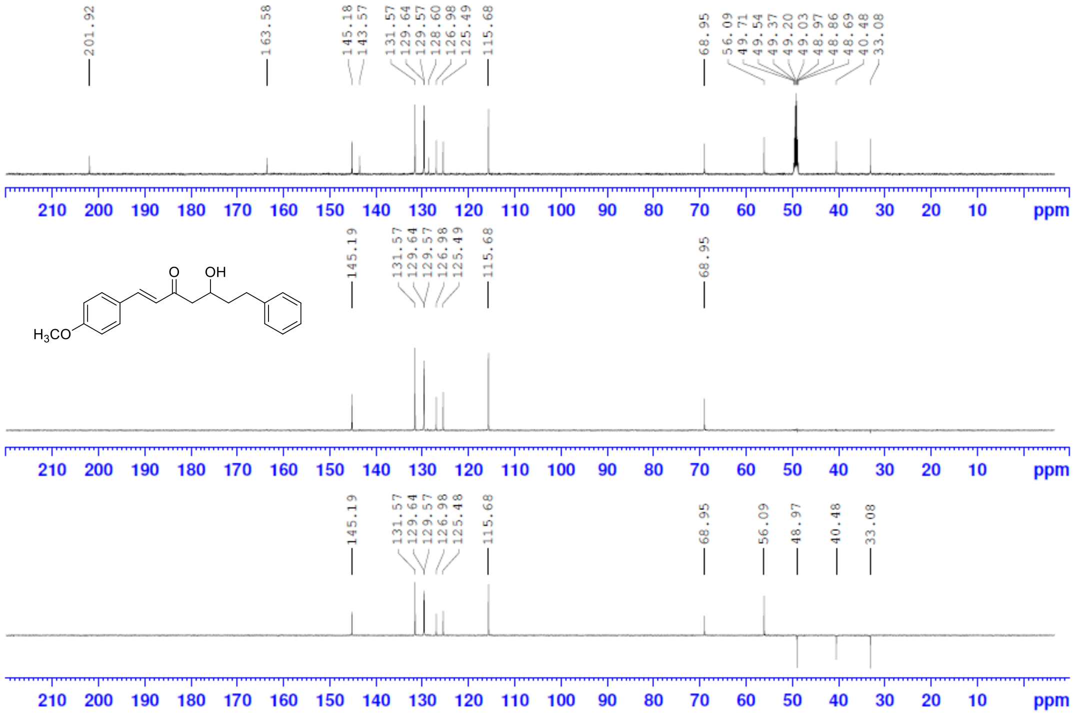
201.92
163.58
145.18
143.57
131.57
129.64
129.57
128.60
126.98
125.49
115.68
68.95
56.09
49.71
49.54
49.37
49.20
49.03
48.97
48.86
48.69
40.48
33.08
145.19
68.95
56.09
48.97
125.48
OH
O
H3CO
ppm

Compound 4a

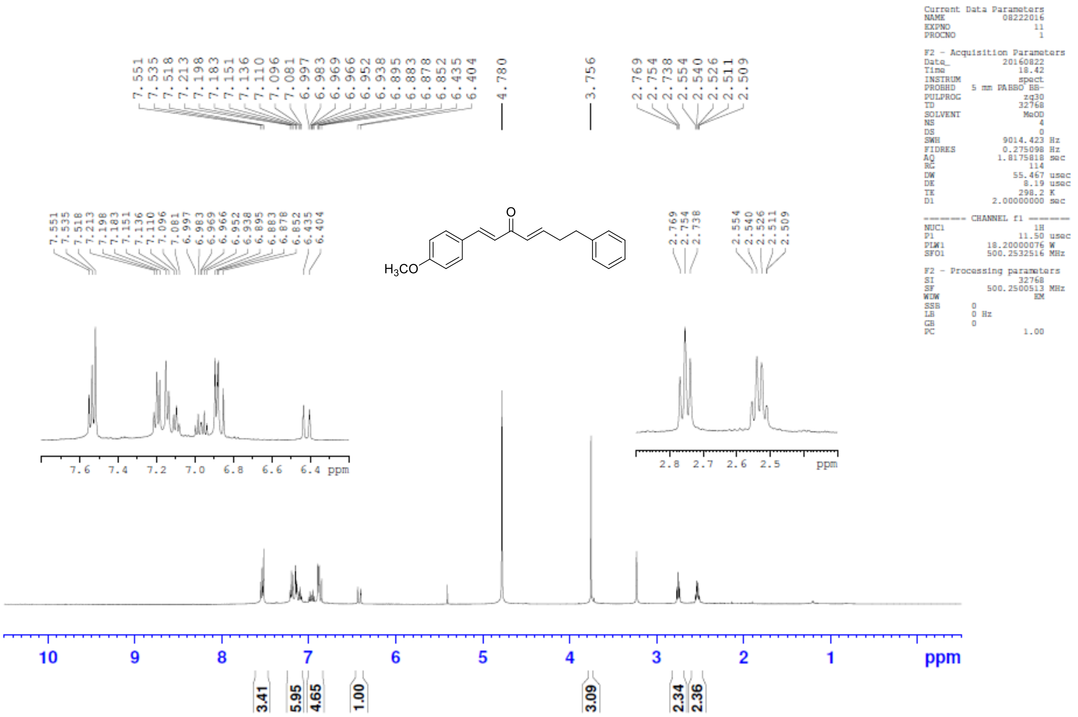
7.551 7.535 7.518 7.213 7.198 7.183 7.151 7.136 7.110 7.096 7.081 6.997 6.983 6.969 6.966 6.952 6.938 6.895 6.883 6.878 6.852 6.435 6.404
4.780
3.756
2.769 2.754 2.738 2.554 2.540 2.526 2.511 2.509
H_3CO
O
3.41 5.95 4.65 1.00 3.09 2.34 2.36
ppm
Current Data Parameters
NAME 08222016
EXPNO 11
PROCNO 1
F2 - Acquisition Parameters
Date_ 20160822
Time 18.42
INSTRUM spect
PROBHD 5 mm PABBO BB-
PULPROG zg30
TD 32768
SOLVENT MeOD
NS 4
DS 0
SWH 9014.423 Hz
FIDRES 0.275098 Hz
AQ 1.8175818 sec
RG 114
DW 55.467 usec
DE 8.19 usec
TE 298.2 K
D1 2.00000000 sec
======== CHANNEL f1 ========
NUC1 1H
P1 11.50 usec
PLW1 18.20000076 W
SFO1 500.2532516 MHz
F2 - Processing parameters
SI 32768
SF 500.2500513 MHz
WDW EM
SSB 0
LB 0 Hz
GB 0
PC 1.00

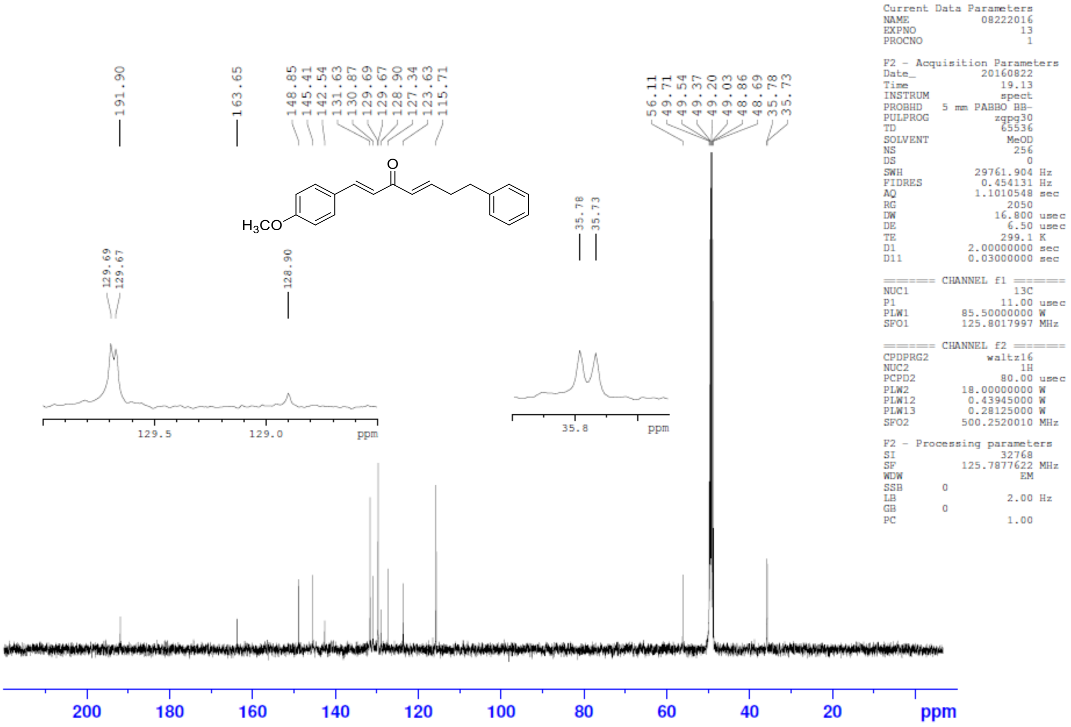
191.90
163.65
148.85 145.41 142.54 131.63 130.87 129.69 129.67 128.90 127.34 123.63 115.71
56.11 49.71 49.54 49.37 49.20 49.03 48.86 48.69 35.78 35.73
H_3CO
O
ppm
Current Data Parameters
NAME 08222016
EXPNO 13
PROCNO 1
F2 - Acquisition Parameters
Date_ 20160822
Time 19.13
INSTRUM spect
PROBHD 5 mm PABBO BB-
PULPROG zgpg30
TD 65536
SOLVENT MeOD
NS 256
DS 0
SWH 29761.904 Hz
FIDRES 0.454131 Hz
AQ 1.1010548 sec
RG 2050
DW 16.800 usec
DE 6.50 usec
TE 299.1 K
D1 2.00000000 sec
D11 0.03000000 sec
======== CHANNEL f1 ========
NUC1 13C
P1 11.00 usec
PLW1 85.50000000 W
SFO1 125.8017997 MHz
======== CHANNEL f2 ========
CPDPRG2 waltz16
NUC2 1H
PCPD2 80.00 usec
PLW2 18.00000000 W
PLW12 0.43945000 W
PLW13 0.28125000 W
SFO2 500.2520010 MHz
F2 - Processing parameters
SI 32768
SF 125.7877622 MHz
WDW EM
SSB 0
LB 2.00 Hz
GB 0
PC 1.00

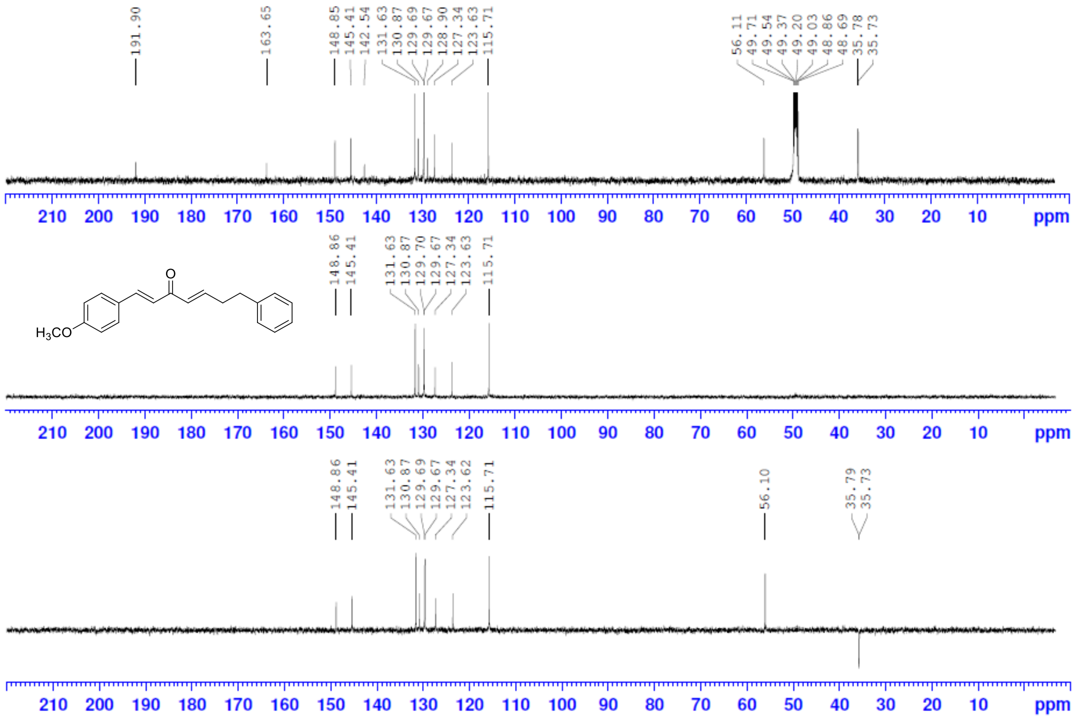
191.90
163.65
148.85
145.41
142.54
131.63
130.87
129.69
129.67
128.90
127.34
123.63
115.71
56.11
49.71
49.54
49.37
49.20
49.03
48.86
48.69
35.78
35.73
ppm
O
H3CO
148.86
145.41
131.63
130.87
129.70
129.67
127.34
123.63
115.71
148.86
145.41
131.63
130.87
129.69
129.67
127.34
123.62
115.71
56.10
35.79
35.73

Compound 5

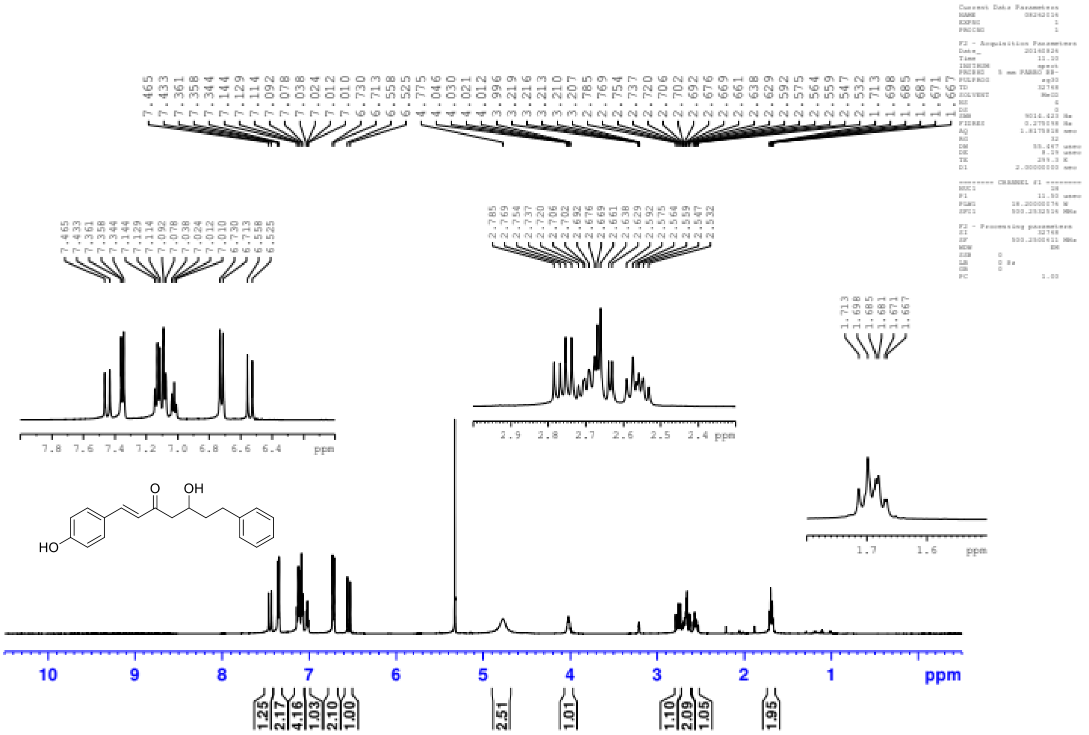
O
OH
HO
ppm
1.25
2.17
4.16
1.03
2.10
1.00
2.51
1.01
1.10
2.09
1.05
1.95

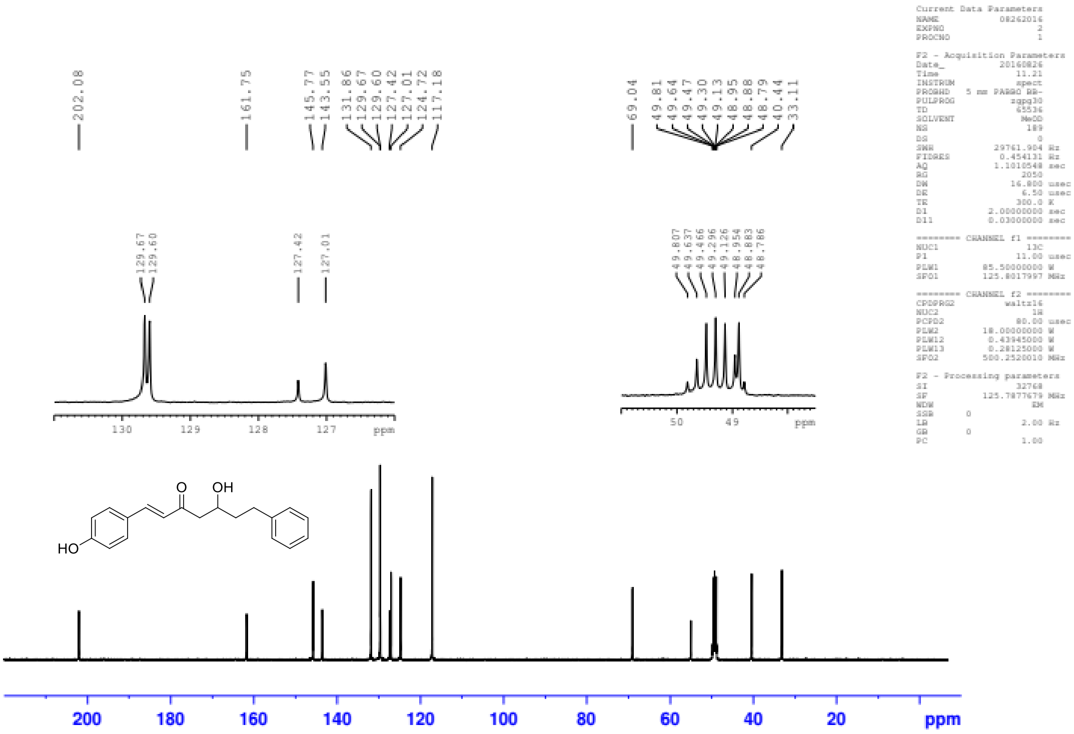
202.08
161.75
145.77
143.55
131.86
129.67
129.60
127.42
127.01
124.72
117.18
69.04
49.81
49.64
49.47
49.30
49.13
48.95
48.88
48.79
40.44
33.11
129.67
129.60
127.42
127.01
49.807
49.637
49.466
49.296
49.126
48.954
48.883
48.786
Current Data Parameters
O
OH
HO
ppm

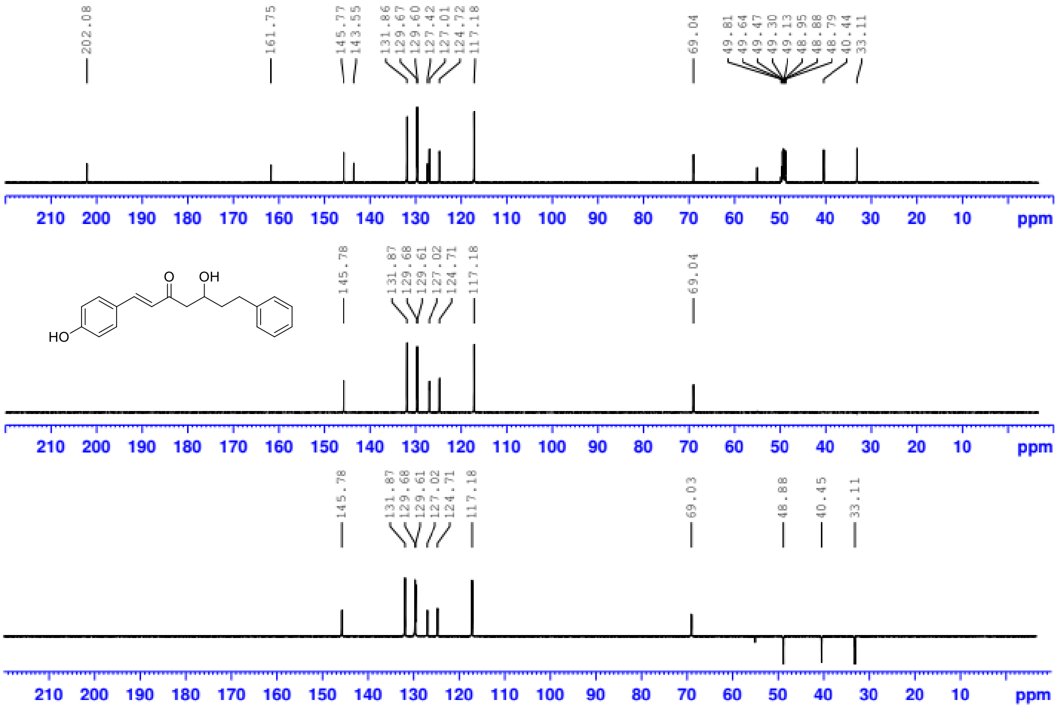
202.08
161.75
145.77
143.55
131.86
129.67
129.60
127.42
127.01
124.72
117.18
69.04
49.81
49.64
49.47
49.30
49.13
48.95
48.88
48.79
40.44
33.11
145.78
131.87
129.68
129.61
127.02
124.71
117.18
69.04
O
OH
HO
145.78
131.87
129.68
129.61
127.02
124.71
117.18
69.03
48.88
40.45
33.11
ppm

Compound 5a

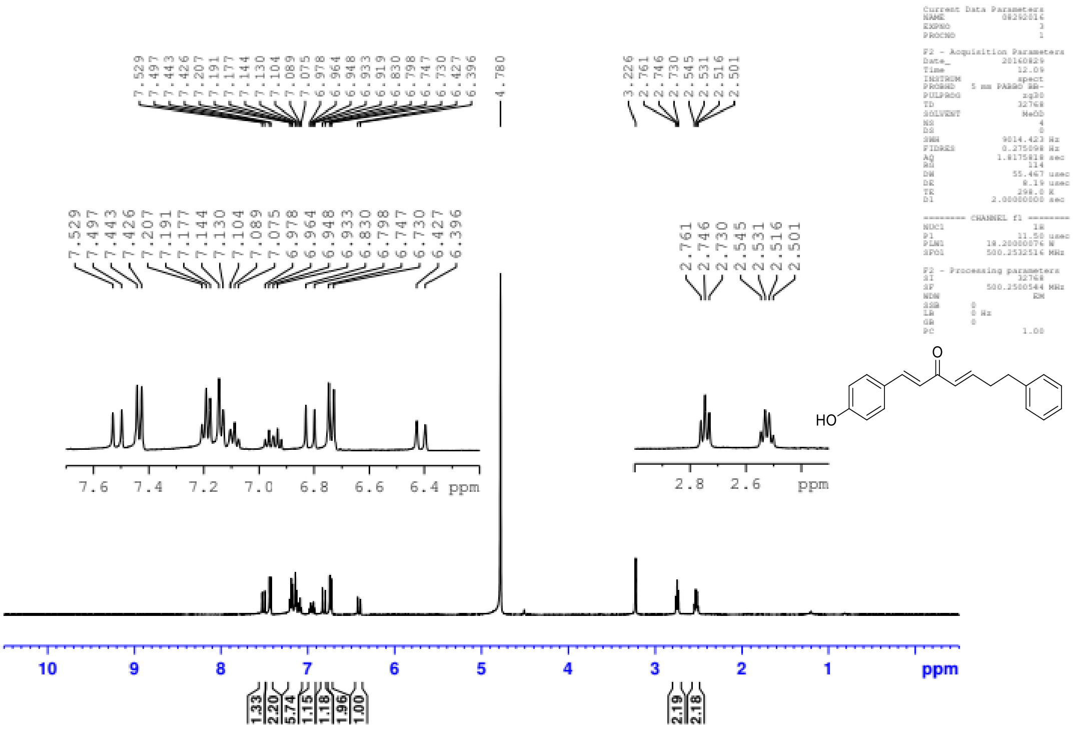

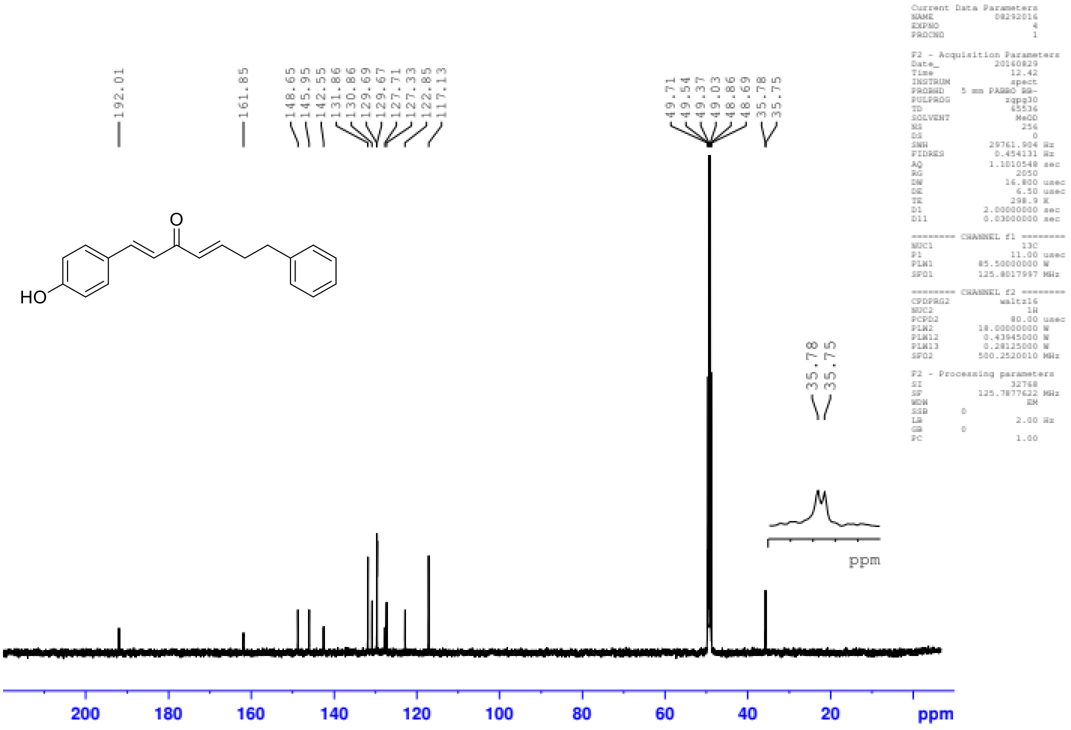

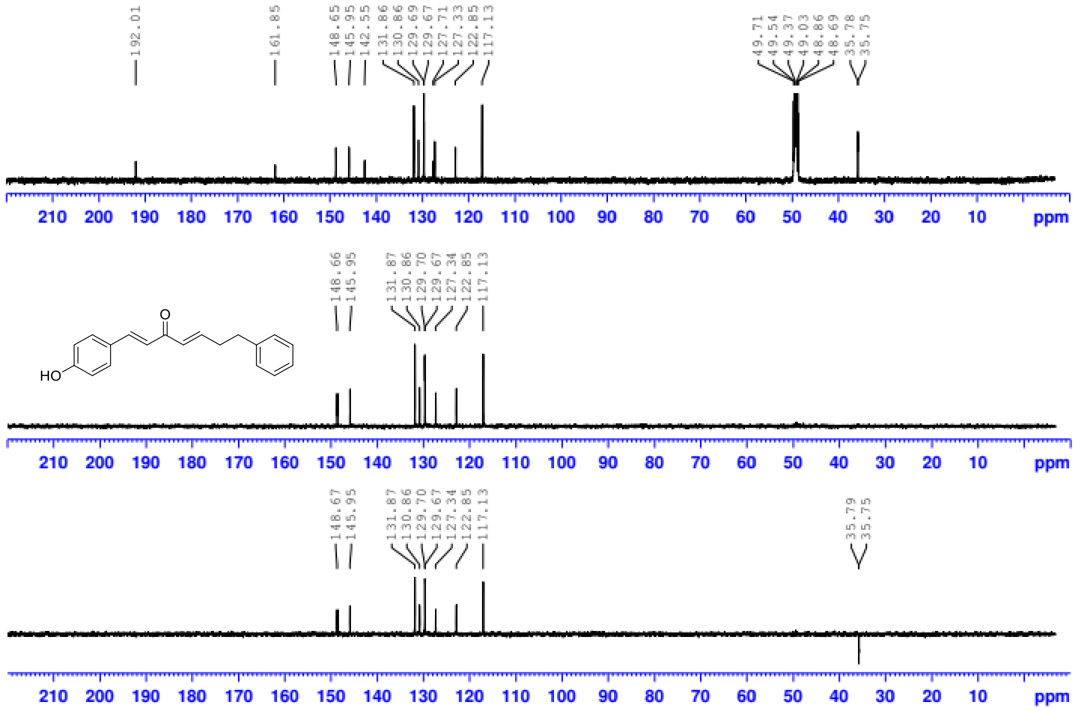

Compound 6

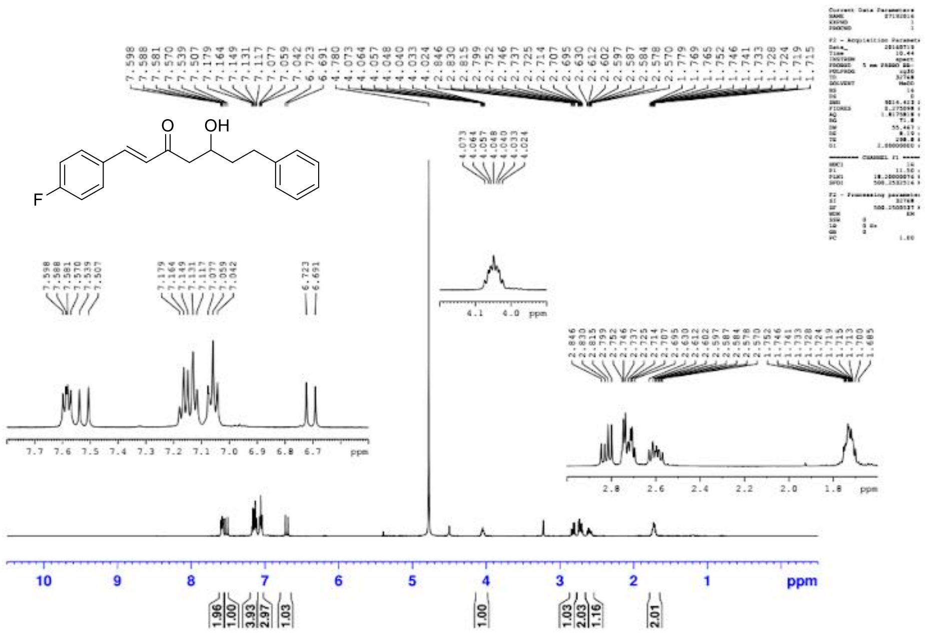

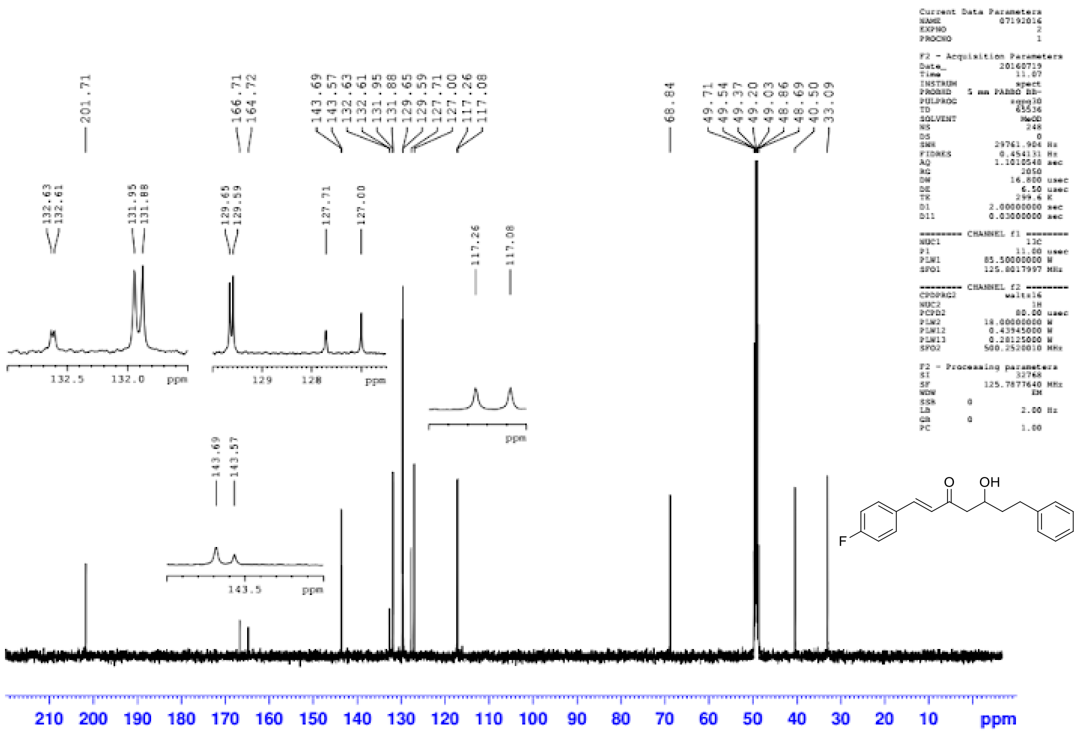
201.71
166.71
164.72
143.69
143.57
132.63
132.61
131.95
131.88
129.65
129.59
127.71
127.00
117.26
117.08
68.84
49.71
49.54
49.37
49.20
49.03
48.86
48.69
40.50
33.09
O
OH
F
ppm

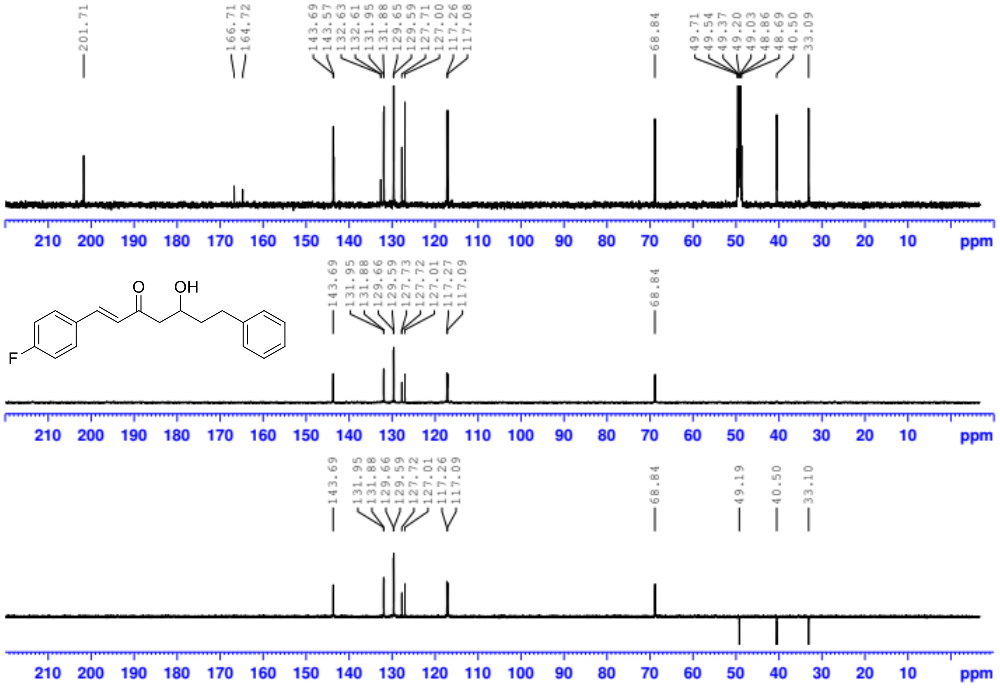
201.71
166.71
164.72
143.69
143.57
132.63
132.61
131.95
131.88
129.65
129.59
127.71
127.00
117.26
117.08
68.84
49.71
49.54
49.37
49.20
49.03
48.86
48.69
40.50
33.09
O
OH
F
143.69
131.95
131.88
129.66
129.59
127.73
127.72
127.01
117.27
117.09
68.84
143.69
131.95
131.88
129.66
129.59
127.72
127.01
117.26
117.09
68.84
49.19
40.50
33.10
ppm

Compound 6a

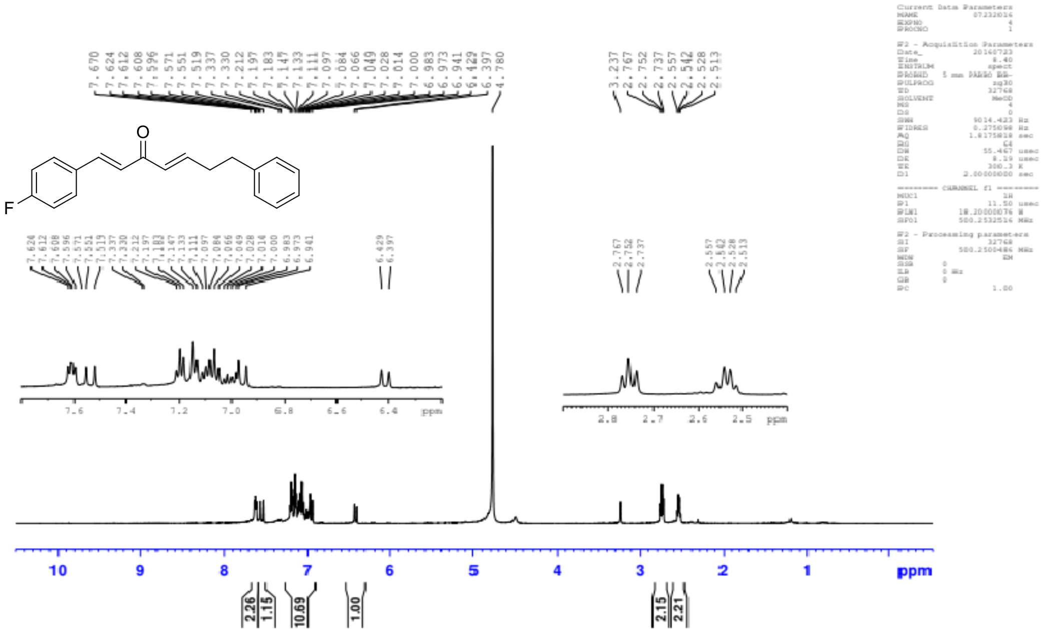
7.670 7.624 7.612 7.608 7.596 7.571 7.551 7.519 7.337 7.330 7.212 7.197 7.183 7.147 7.133 7.111 7.097 7.084 7.066 7.049 7.028 7.014 7.000 6.983 6.973 6.941 6.429 6.397 4.780
3.237 2.767 2.752 2.737 2.557 2.542 2.528 2.513
O
F
2.26 1.15 10.69 1.00 2.15 2.21
ppm

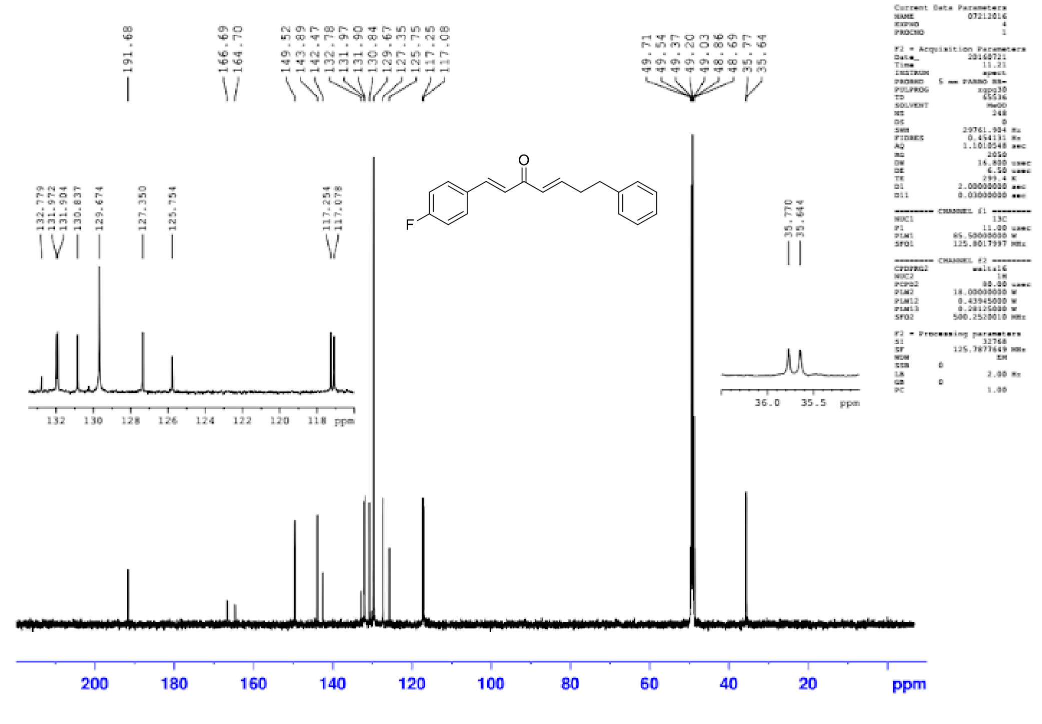
191.68
166.69 164.70
149.52 143.89 142.47 132.78 131.97 131.90 130.84 129.67 127.35 125.75 117.25 117.08
49.71 49.54 49.37 49.20 49.03 48.86 48.69 35.77 35.64
132.779 131.972 131.904 130.837 129.674 127.350 125.754 117.254 117.078
35.770 35.644
O
F
ppm

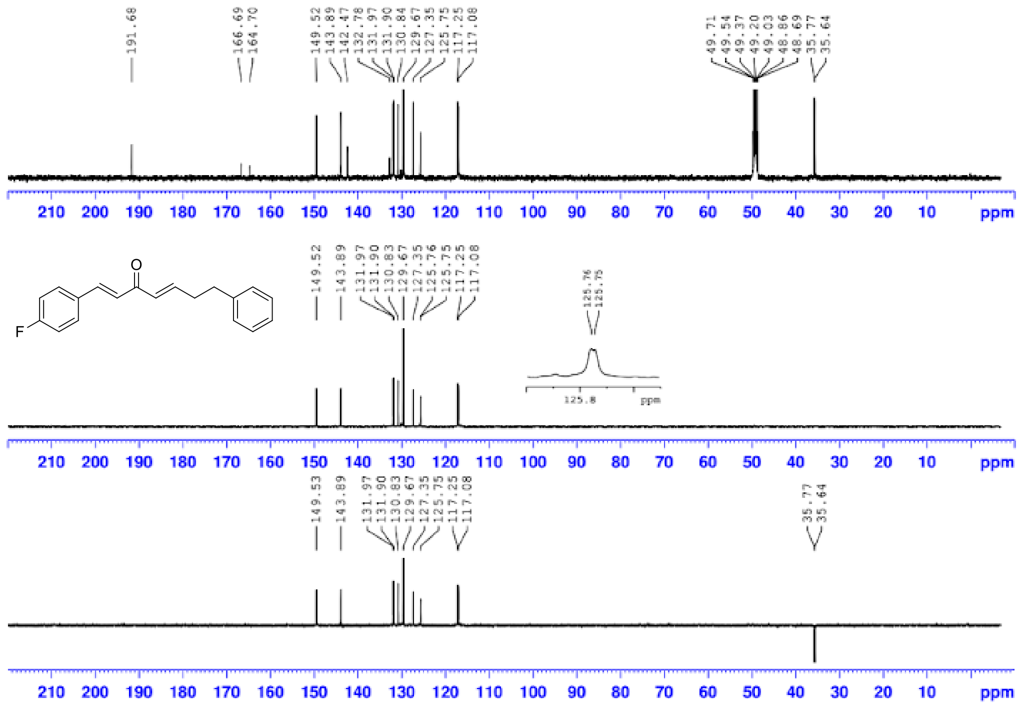
191.68
166.69
164.70
149.52
143.89
142.47
132.78
131.97
131.90
130.84
129.67
127.35
125.75
117.25
117.08
49.71
49.54
49.37
49.20
49.03
48.86
48.69
35.77
35.64
F
O
125.76
125.75
125.8
ppm
149.53
143.89
131.97
131.90
130.83
129.67
127.35
125.75
117.25
117.08
35.77
35.64
210 200 190 180 170 160 150 140 130 120 110 100 90 80 70 60 50 40 30 20 10 ppm

Compound 7

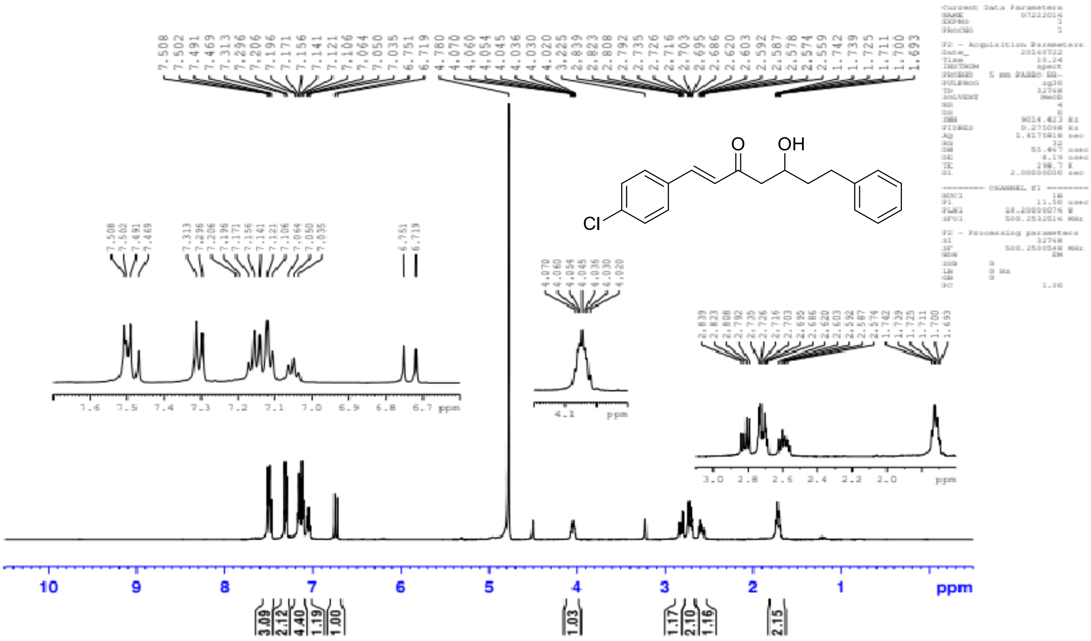
O
OH
Cl
7.5 7.4 7.3 7.2 7.1 7.0 6.9 6.8 6.7 ppm
4.1 ppm
3.0 2.8 2.6 2.4 2.2 2.0 ppm
10 9 8 7 6 5 4 3 2 1 ppm
3.09
2.12
4.40
1.19
1.00
1.03
1.17
2.10
1.16
2.15

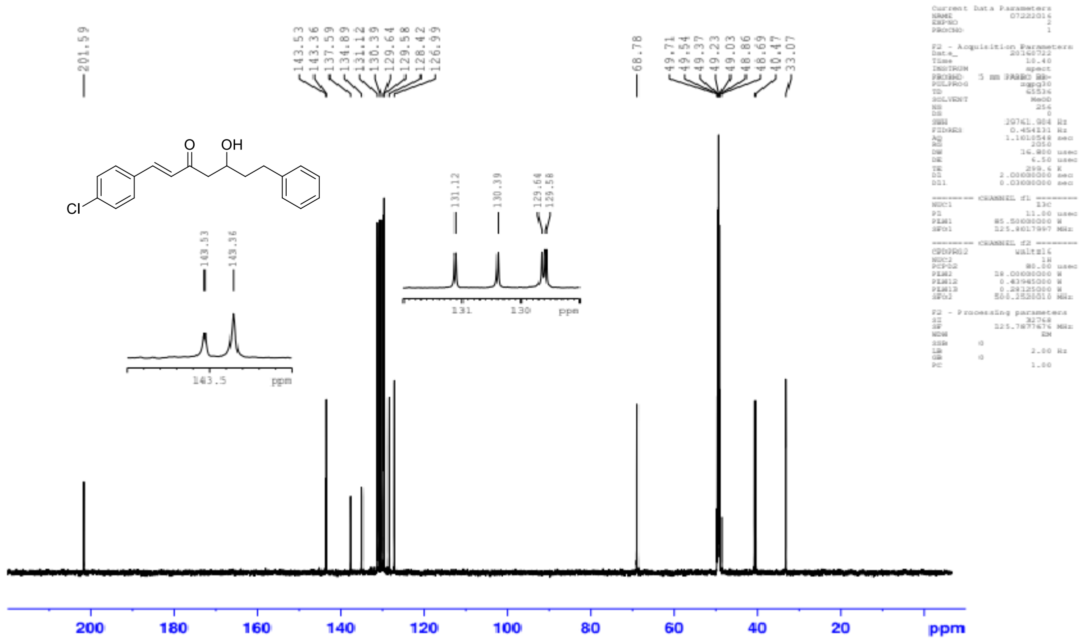
201.59
143.53
143.36
137.59
134.89
131.12
130.39
129.64
129.58
128.42
126.99
68.78
49.71
49.54
49.37
49.23
49.03
48.86
48.69
40.47
33.07
O
OH
Cl
143.53
143.36
143.5
ppm
131.12
130.39
129.64
129.58
131
130
ppm
200
180
160
140
120
100
80
60
40
20
ppm

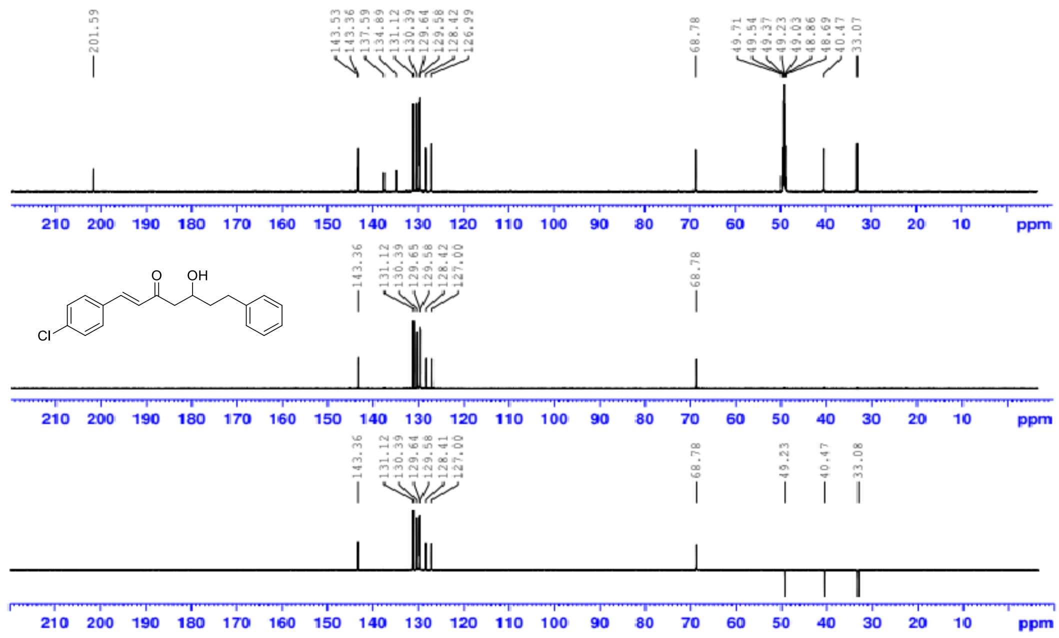
201.59
143.53
143.36
137.59
134.89
131.12
130.39
129.64
129.58
128.42
126.99
68.78
49.71
49.54
49.37
49.23
49.03
48.86
48.69
40.47
33.07
143.36
131.12
130.39
129.65
129.58
128.42
127.00
68.78
O
OH
Cl
143.36
131.12
130.39
129.64
129.58
128.41
127.00
68.78
49.23
40.47
33.08
210
200
190
180
170
160
150
140
130
120
110
100
90
80
70
60
50
40
30
20
10
ppm

Compound 7a

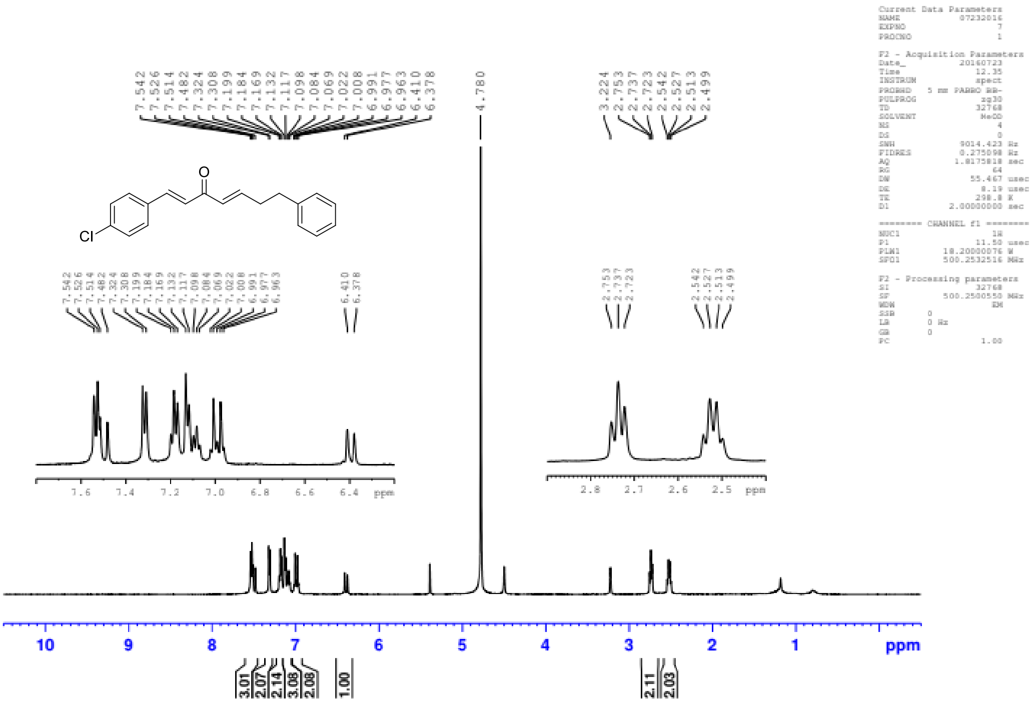
7.542 7.526 7.514 7.482 7.324 7.308 7.199 7.184 7.169 7.132 7.117 7.098 7.084 7.069 7.022 7.008 6.991 6.977 6.963 6.410 6.378
4.780
3.224 2.753 2.737 2.723 2.542 2.527 2.513 2.499
Cl
O
3.01 2.07 2.14 3.08 2.08 1.00 2.11 2.03
ppm

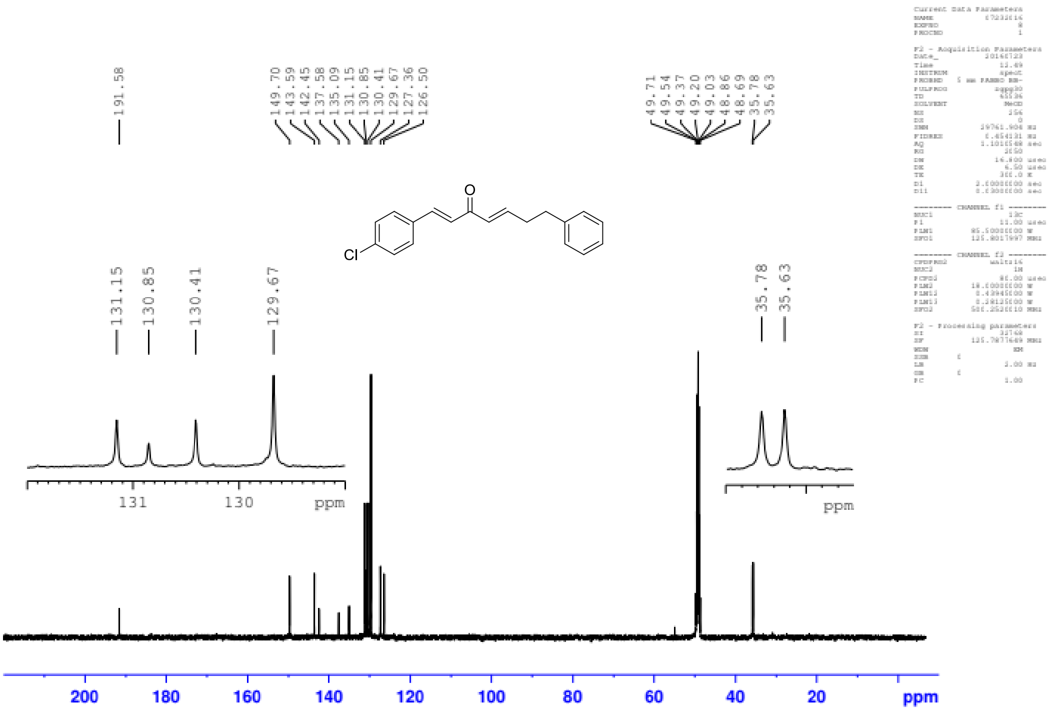
191.58
149.70 143.59 142.45 137.58 135.09 131.15 130.85 130.41 129.67 127.36 126.50
49.71 49.54 49.37 49.20 49.03 48.86 48.69 35.78 35.63
Cl
O
131.15 130.85 130.41 129.67
35.78 35.63
ppm

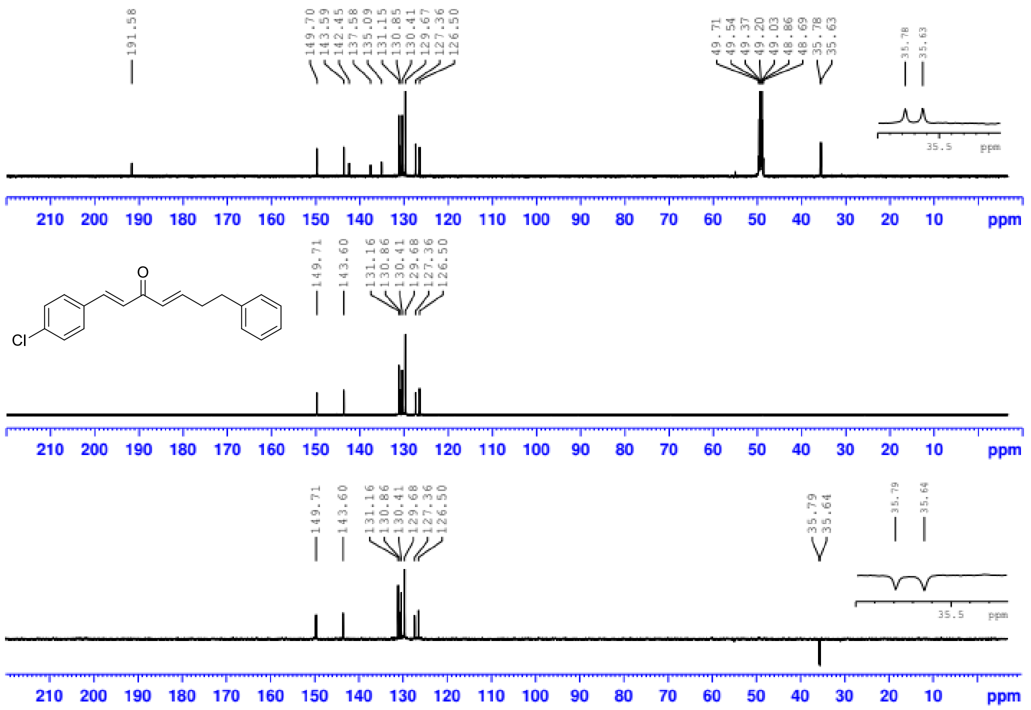

Compound 8

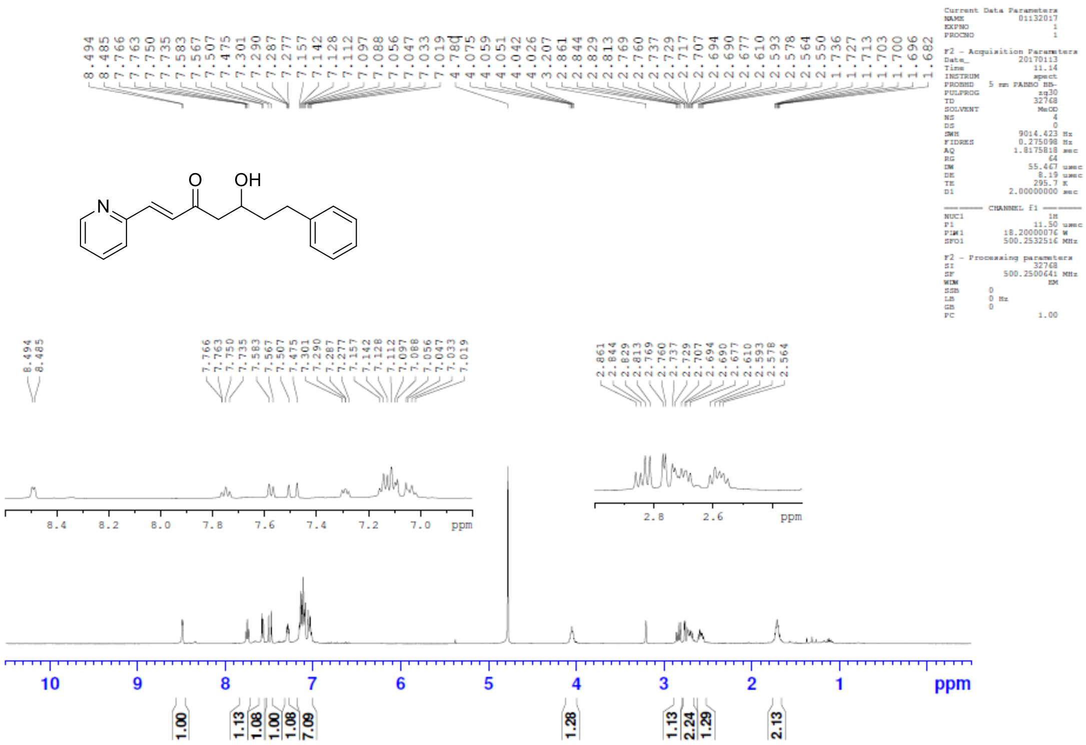

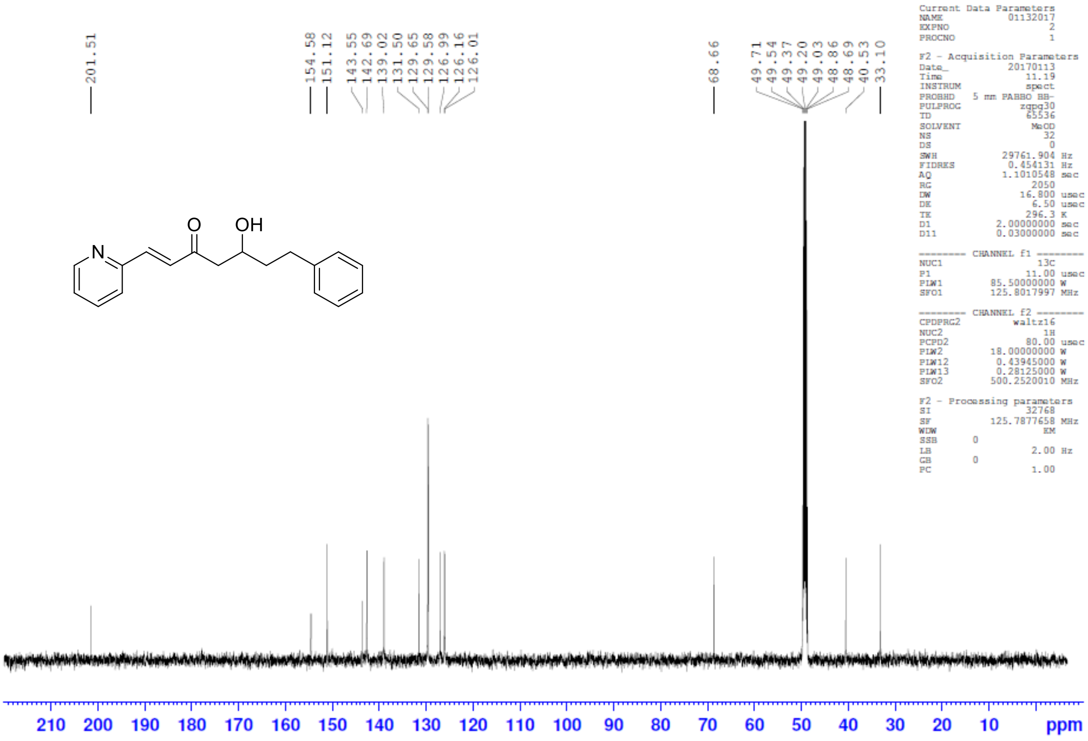
201.51
154.58
151.12
143.55
142.69
139.02
131.50
129.65
129.58
126.99
126.16
126.01
68.66
49.71
49.54
49.37
49.20
49.03
48.86
48.69
40.53
33.10
O
OH
N
Current Data Parameters
NAME 01132017
EXPNO 2
PROCNO 1
F2 - Acquisition Parameters
Date_ 20170113
Time 11.19
INSTRUM spect
PROBHD 5 mm PABBO BB-
PULPROG zgpg30
TD 65536
SOLVENT MeOD
NS 32
DS 0
SWH 29761.904 Hz
FIDRES 0.454131 Hz
AQ 1.1010548 sec
RG 2050
DW 16.800 usec
DE 6.50 usec
TE 296.3 K
D1 2.00000000 sec
D11 0.03000000 sec
======== CHANNEL f1 ========
NUC1 13C
P1 11.00 usec
PLW1 85.50000000 W
SFO1 125.8017997 MHz
======== CHANNEL f2 ========
CPDPRG2 waltz16
NUC2 1H
PCPD2 80.00 usec
PLW2 18.00000000 W
PLW12 0.43945000 W
PLW13 0.28125000 W
SFO2 500.2520010 MHz
F2 - Processing parameters
SI 32768
SF 125.7877658 MHz
WDW EM
SSB 0
LB 2.00 Hz
GB 0
PC 1.00
210 200 190 180 170 160 150 140 130 120 110 100 90 80 70 60 50 40 30 20 10 ppm

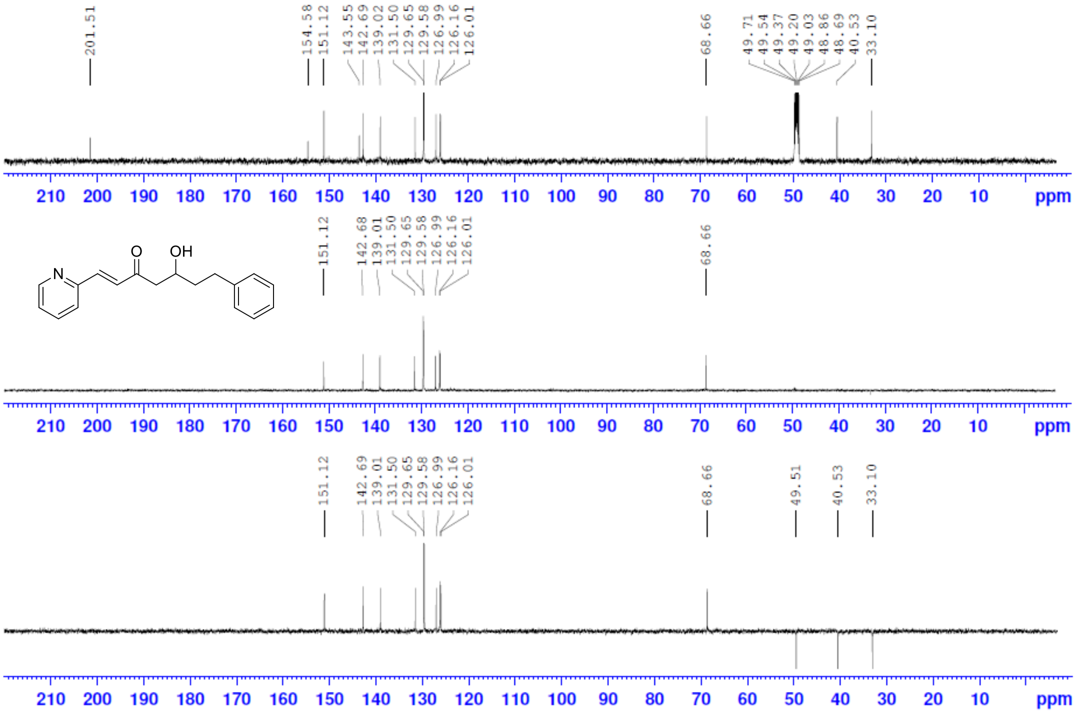
201.51
154.58
151.12
143.55
142.69
139.02
131.50
129.65
129.58
126.99
126.16
126.01
68.66
49.71
49.54
49.37
49.20
49.03
48.86
48.69
40.53
33.10
210 200 190 180 170 160 150 140 130 120 110 100 90 80 70 60 50 40 30 20 10 ppm
O
OH
N
151.12
142.68
139.01
131.50
129.65
129.58
126.99
126.16
126.01
68.66
210 200 190 180 170 160 150 140 130 120 110 100 90 80 70 60 50 40 30 20 10 ppm
151.12
142.69
139.01
131.50
129.65
129.58
126.99
126.16
126.01
68.66
49.51
40.53
33.10
210 200 190 180 170 160 150 140 130 120 110 100 90 80 70 60 50 40 30 20 10 ppm

Compound 9

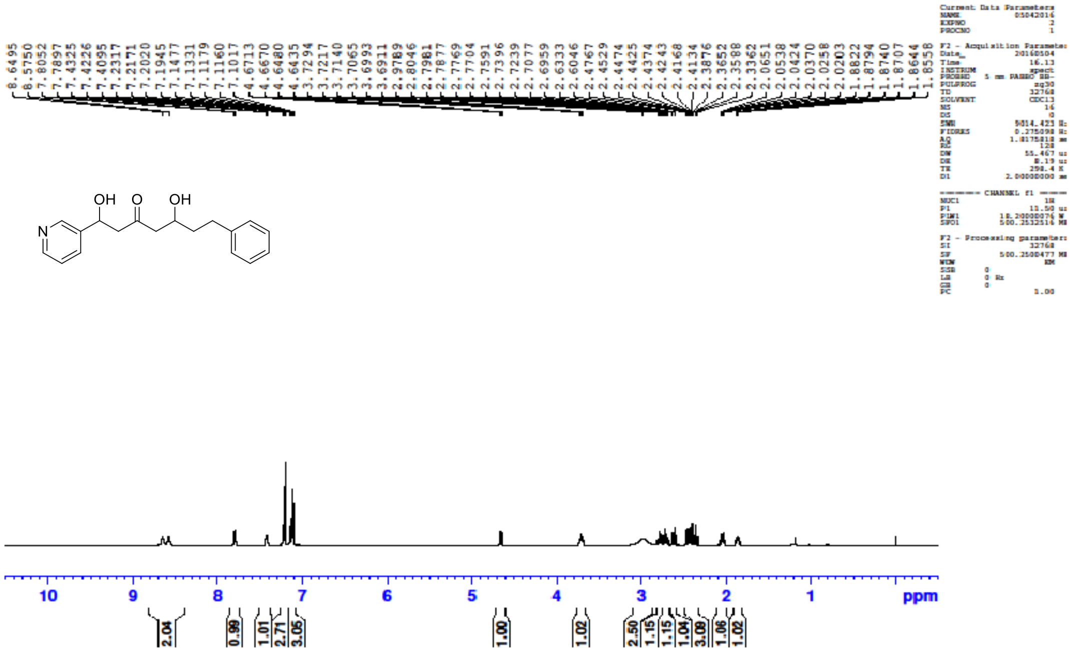

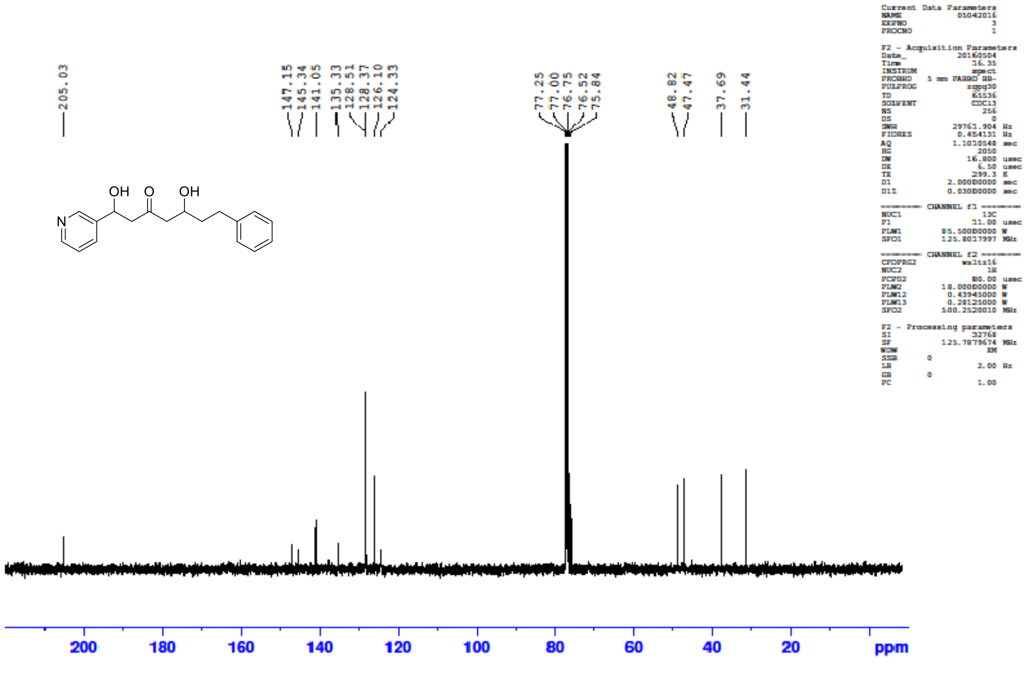

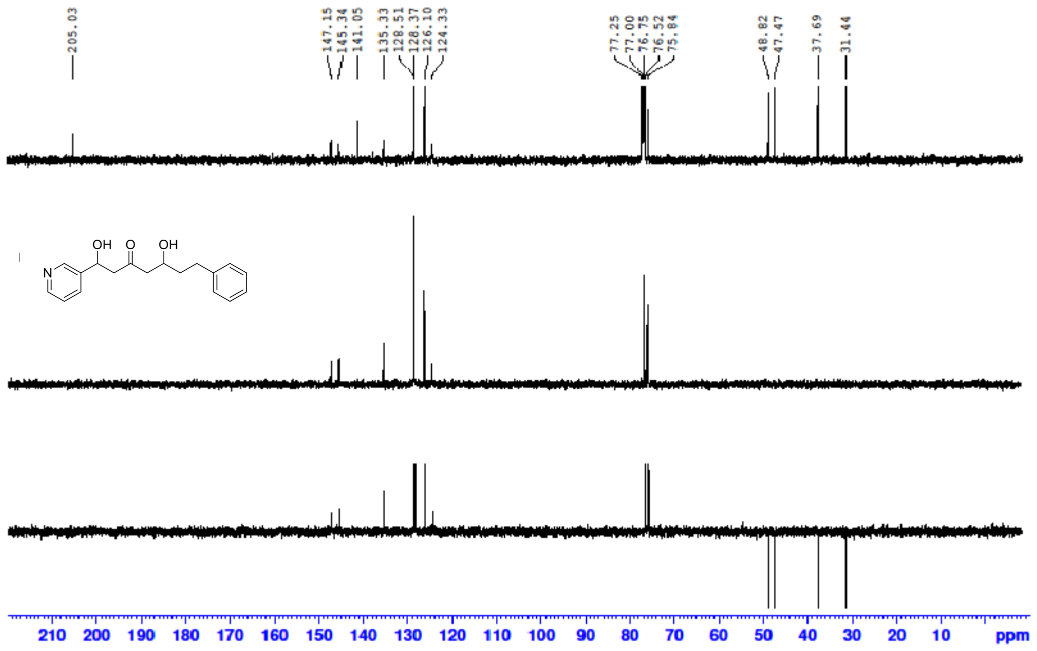
205.03
147.15
145.34
141.05
135.33
128.51
128.37
126.10
124.33
77.25
77.00
76.75
76.52
75.84
48.82
47.47
37.69
31.44
OH
O
OH
N
210
200
190
180
170
160
150
140
130
120
110
100
90
80
70
60
50
40
30
20
10
ppm

Compound 10

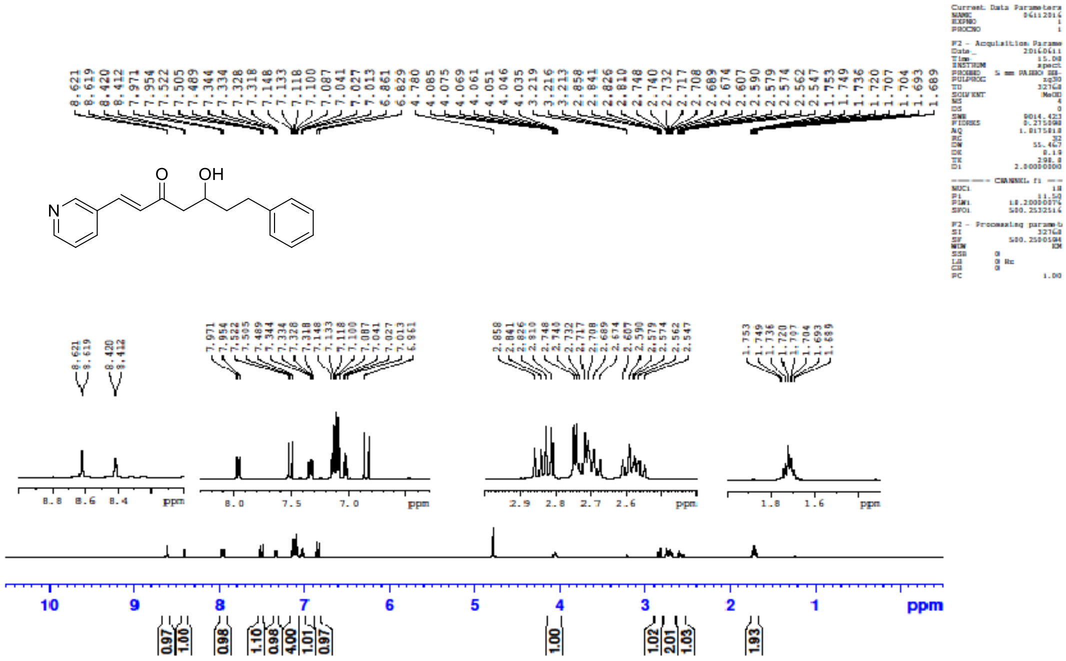
O
OH
N
10
9
8
7
6
5
4
3
2
1
ppm
0.97
1.00
0.98
1.10
0.98
4.00
1.01
0.97
1.00
1.02
2.01
1.03
1.93

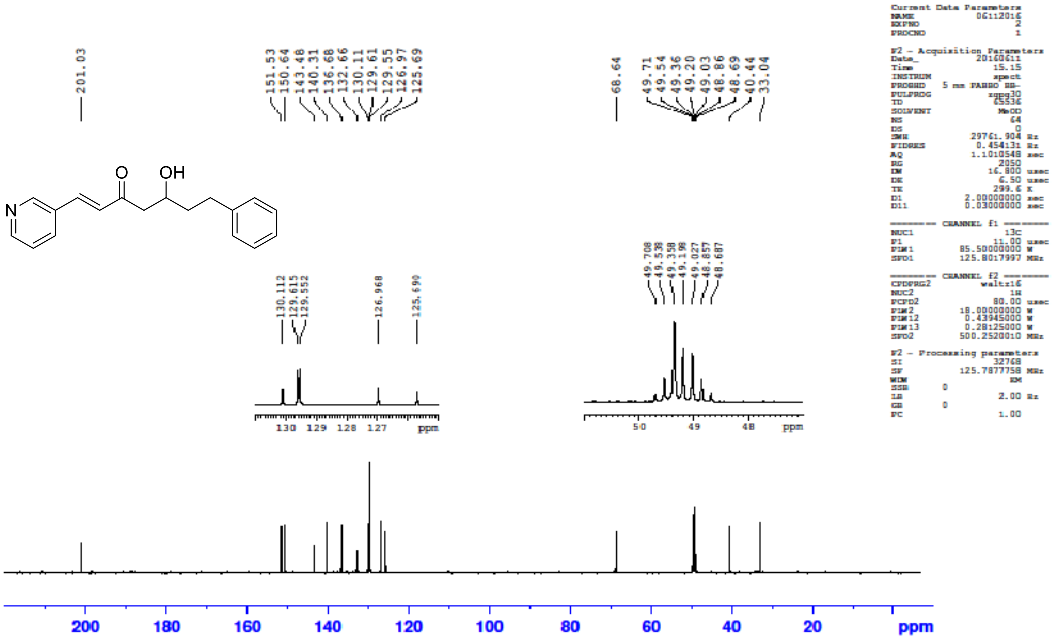
201.03
151.53 150.64 143.48 140.31 136.68 132.66 130.11 129.61 129.55 126.97 125.69
68.64
49.71 49.54 49.36 49.20 49.03 48.86 48.69 40.44 33.04
O
OH
N
130.112 129.615 129.552 126.968 125.690
49.708 49.538 49.358 49.198 49.027 48.857 48.687
ppm

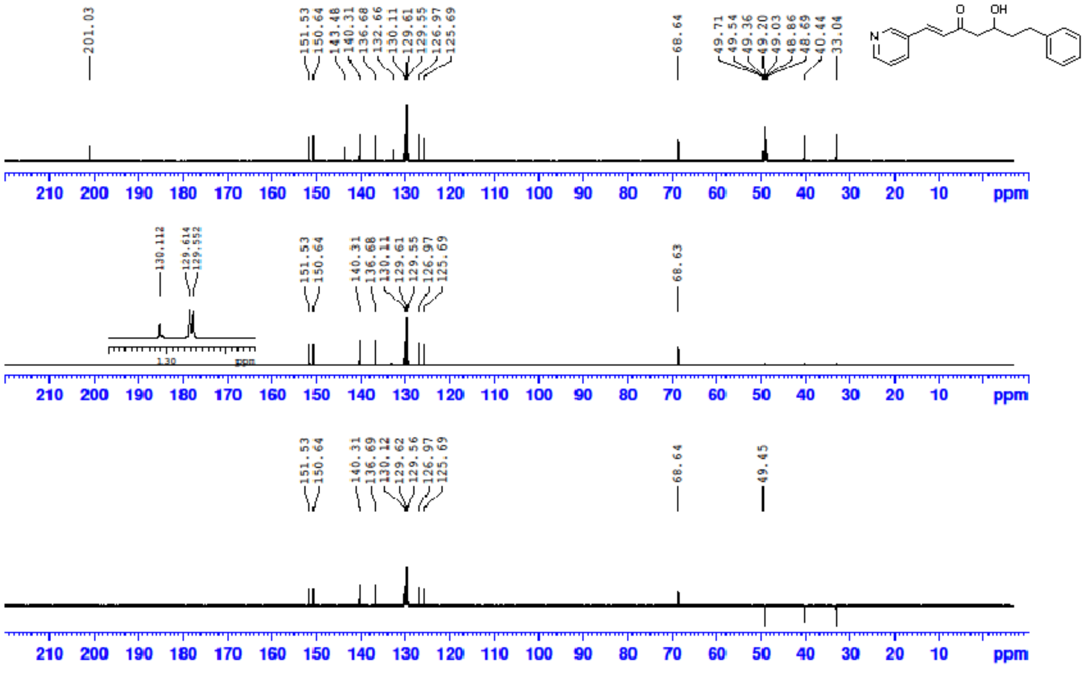
201.03
151.53 150.64 143.48 140.31 136.68 132.66 130.11 129.61 129.55 126.97 125.69
68.64
49.71 49.54 49.36 49.20 49.03 48.86 48.69 40.44 33.04
O
OH
N
ppm
130.112 129.614 129.552
151.53 150.64 140.31 136.68 130.11 129.61 129.55 126.97 125.69
68.63
151.53 150.64 140.31 136.69 130.12 129.62 129.56 126.97 125.69
68.64
49.45

Compound 11

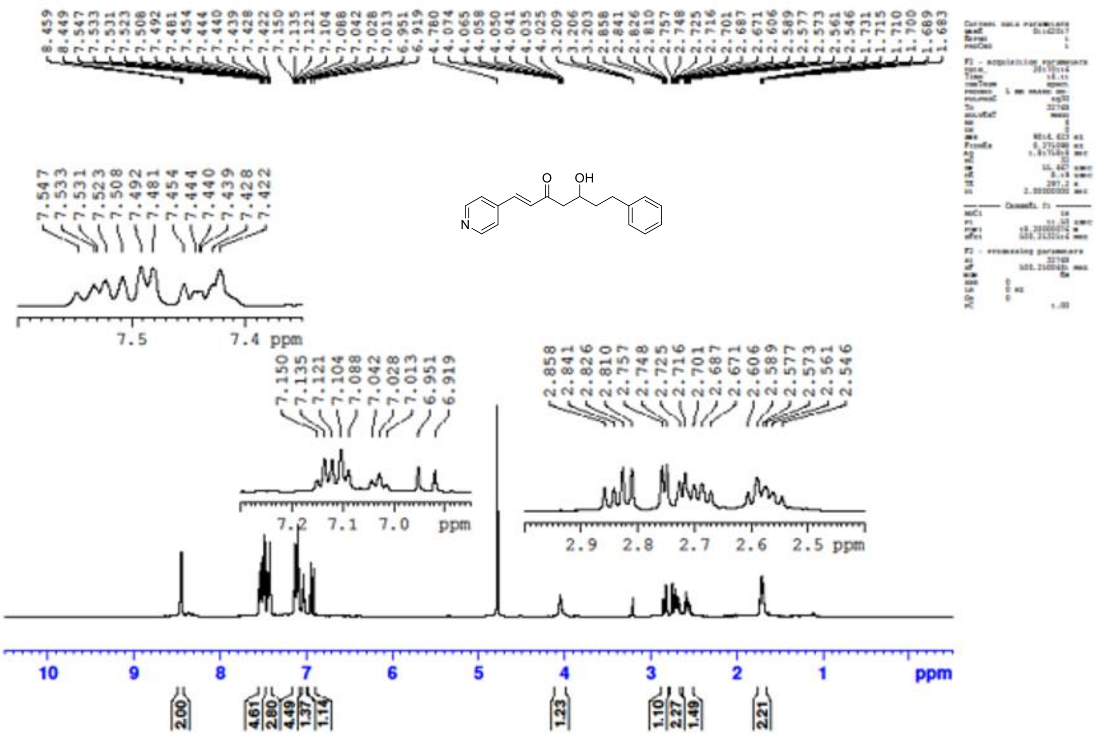

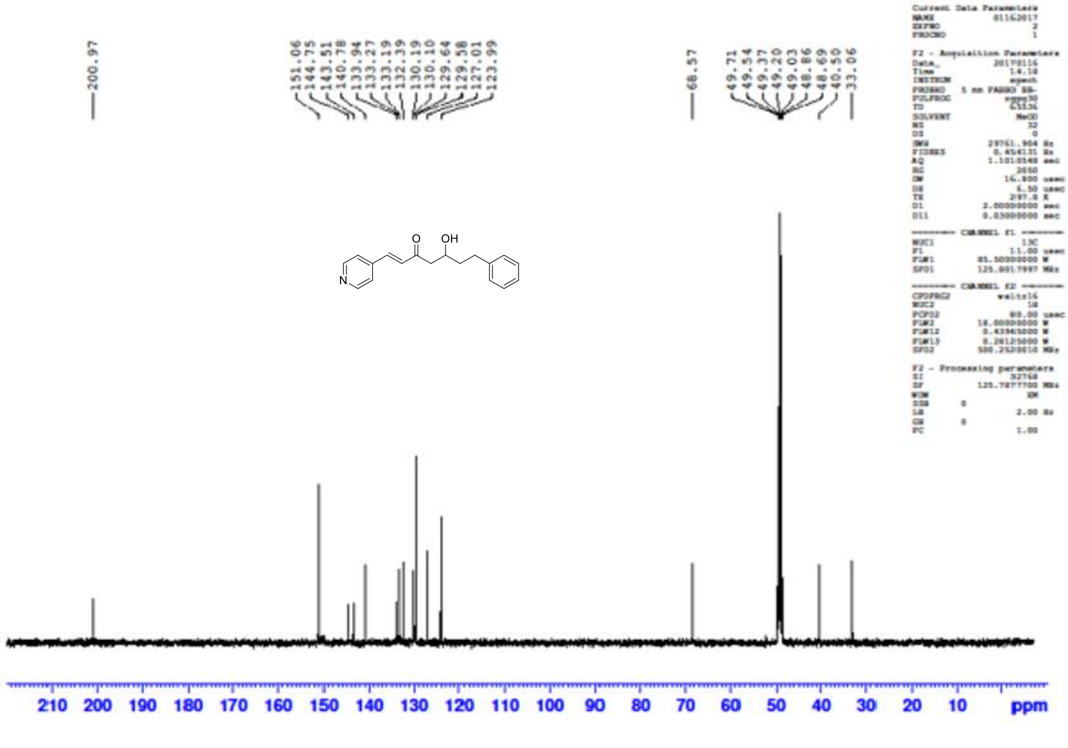
200.97
151.06
144.75
143.51
140.78
133.94
133.27
133.19
132.39
130.19
130.10
129.64
129.58
127.01
123.99
68.57
49.71
49.54
49.37
49.20
49.03
48.86
48.69
40.50
33.06
O
OH
N
ppm

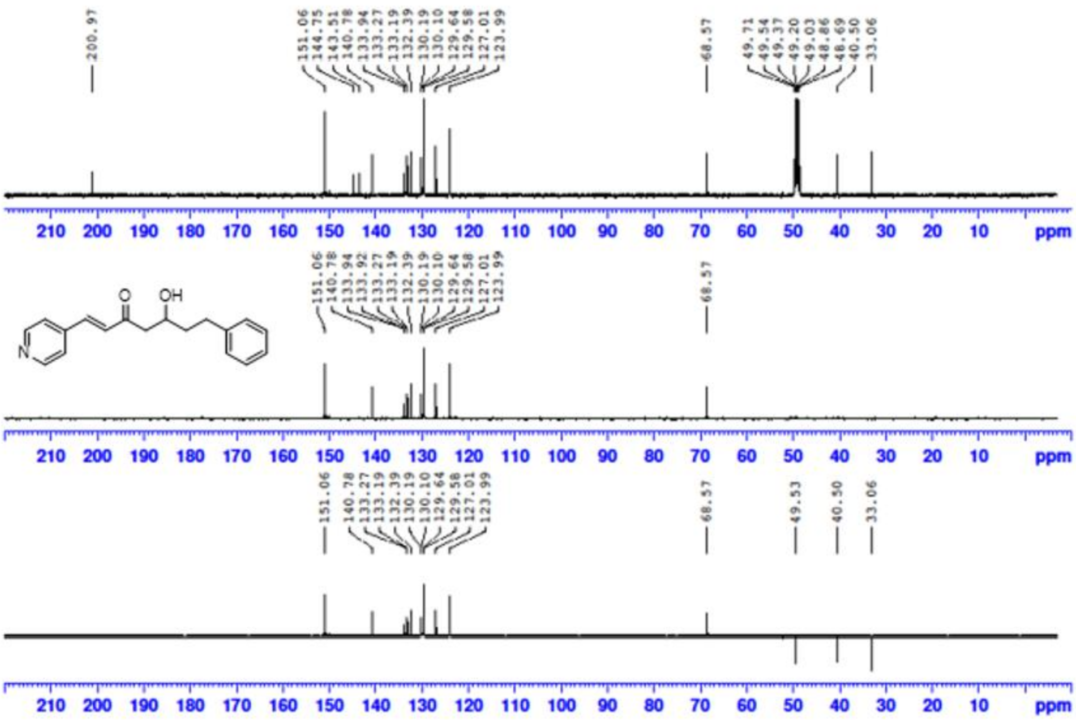
200.97
151.06
144.75
143.51
140.78
133.94
133.27
133.19
132.39
130.19
130.10
129.64
129.58
127.01
123.99
68.57
49.71
49.54
49.37
49.20
49.03
48.86
48.69
40.50
33.06
ppm
O
OH
N
151.06
140.78
133.94
133.92
133.27
133.19
132.39
130.19
130.10
129.64
129.58
127.01
123.99
68.57
ppm
151.06
140.78
133.27
133.19
132.39
130.19
130.10
129.64
129.58
127.01
123.99
68.57
49.53
40.50
33.06
ppm

Compound 12

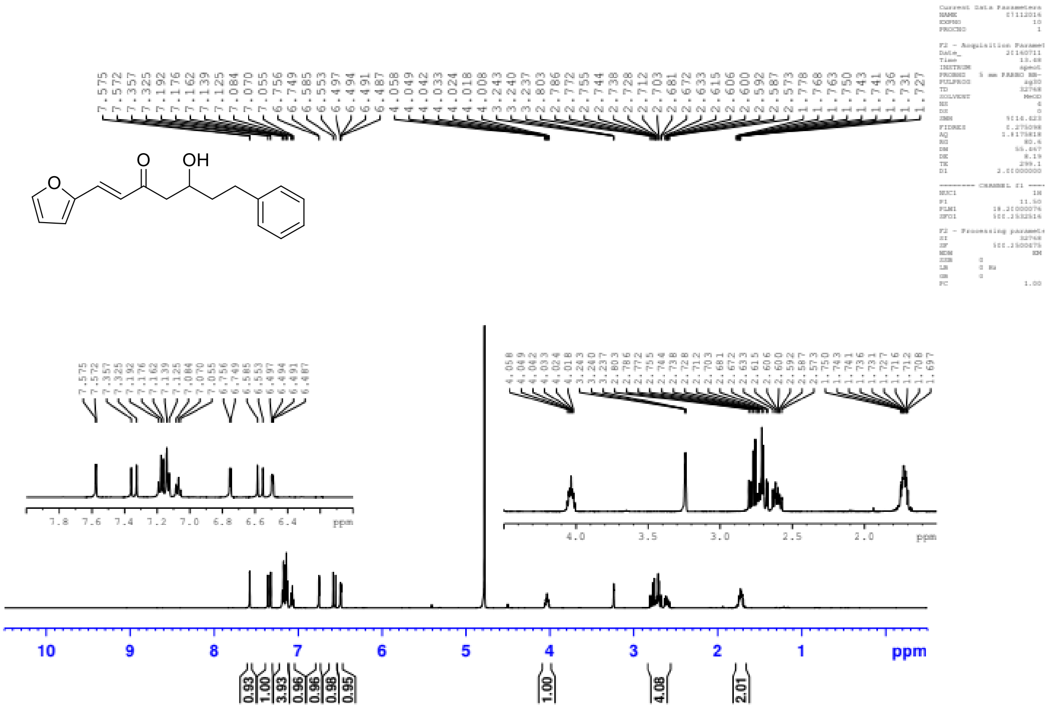
O
OH
ppm
0.93
1.00
3.93
0.96
0.96
0.98
0.95
1.00
4.08
2.01

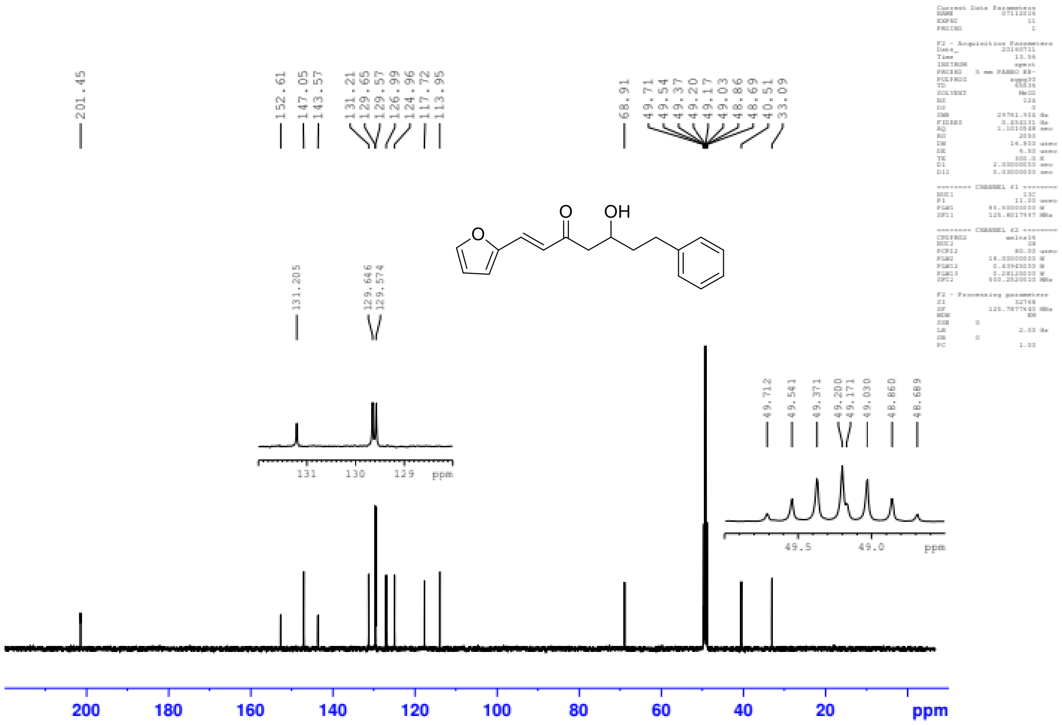

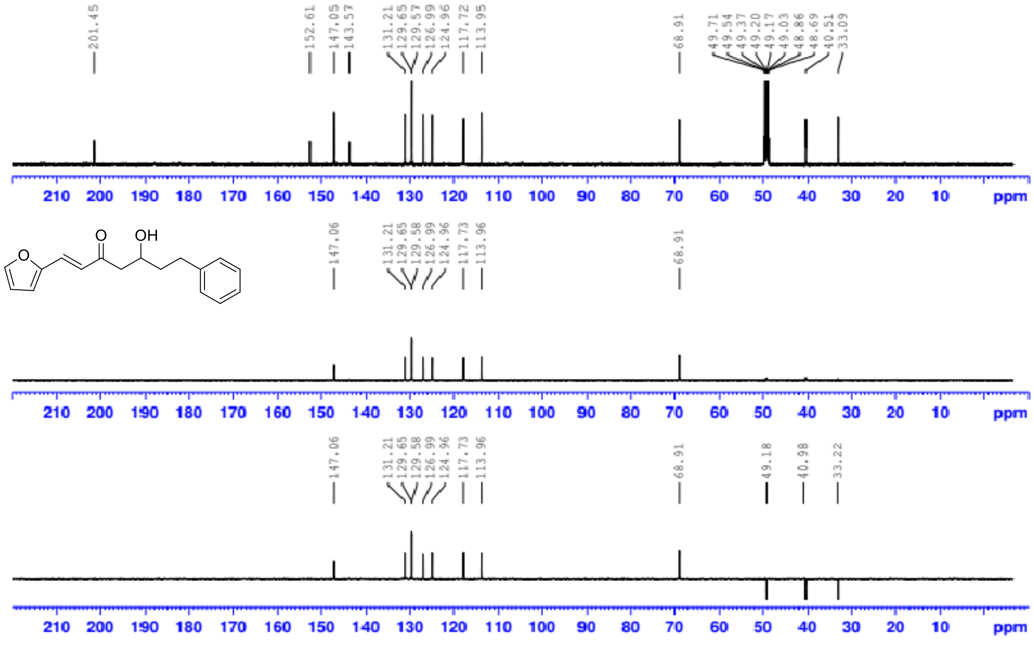

Compound 12a

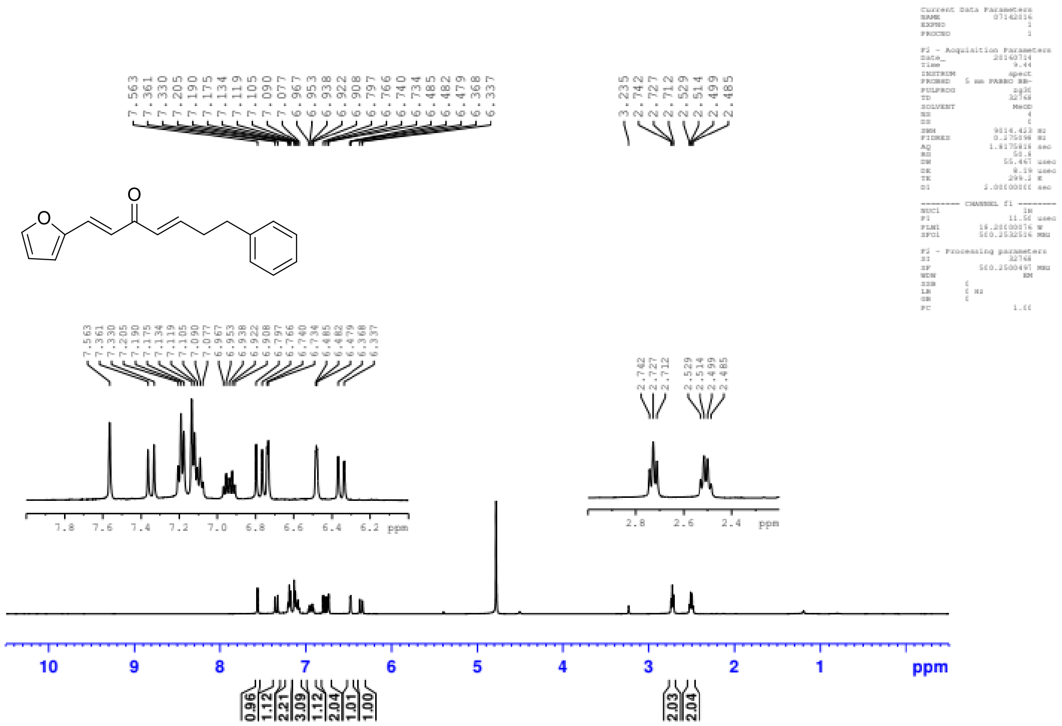

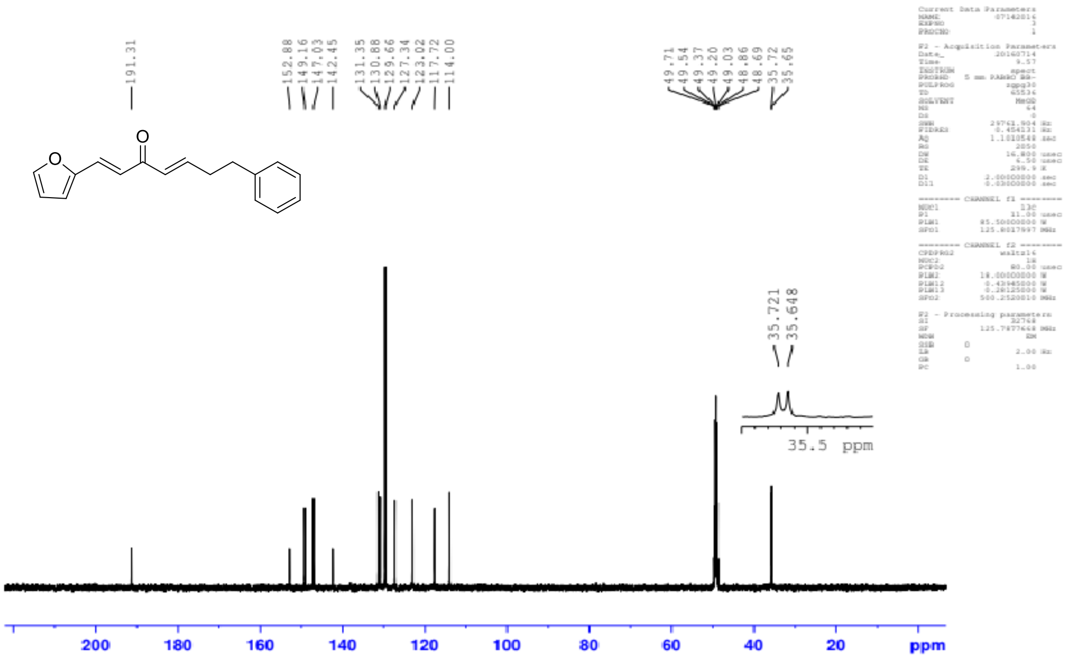

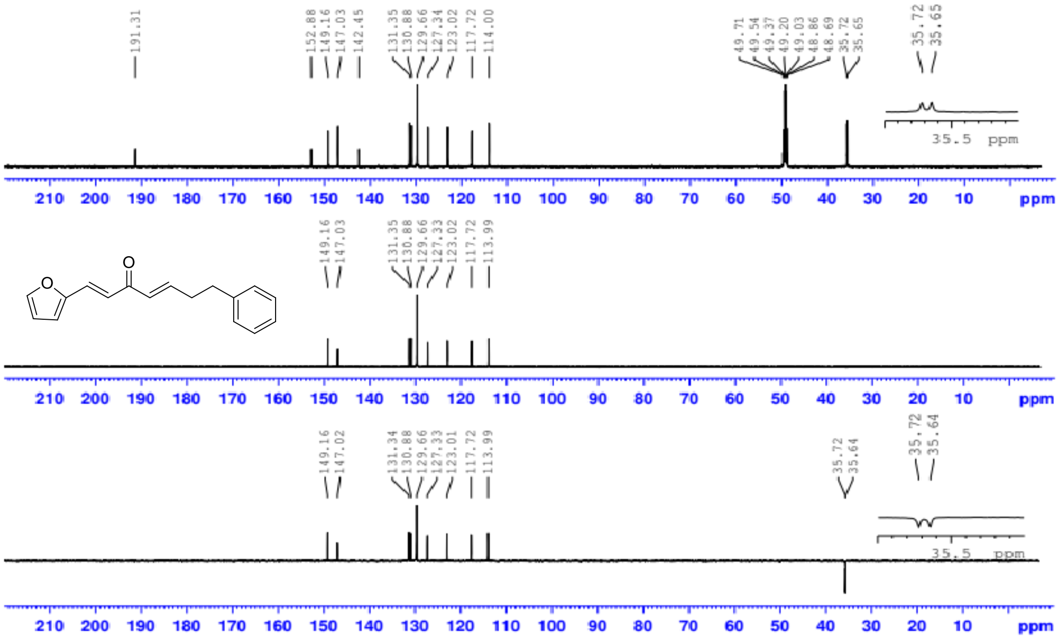
191.31
152.88
149.16
147.03
142.45
131.35
130.88
129.66
127.34
123.02
117.72
114.00
49.71
49.54
49.37
49.20
49.03
48.86
48.69
35.72
35.65
35.72
35.65
35.5 ppm
210 200 190 180 170 160 150 140 130 120 110 100 90 80 70 60 50 40 30 20 10 ppm
149.16
147.03
131.35
130.88
129.66
127.33
123.02
117.72
113.99
O
O
210 200 190 180 170 160 150 140 130 120 110 100 90 80 70 60 50 40 30 20 10 ppm
149.16
147.02
131.34
130.88
129.66
127.33
123.01
117.72
113.99
35.72
35.64
35.72
35.64
35.5 ppm
210 200 190 180 170 160 150 140 130 120 110 100 90 80 70 60 50 40 30 20 10 ppm

Compound 14

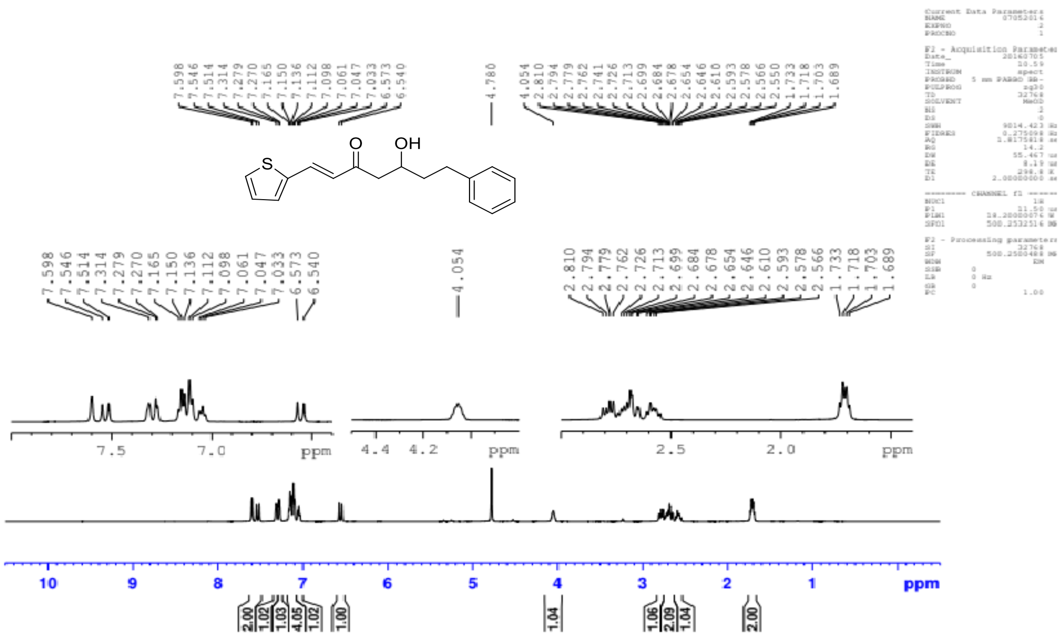
7.598
7.546
7.514
7.314
7.279
7.270
7.165
7.150
7.136
7.112
7.098
7.061
7.047
7.033
6.573
6.540
4.780
4.054
2.810
2.794
2.779
2.762
2.741
2.726
2.713
2.699
2.684
2.678
2.654
2.646
2.610
2.593
2.578
2.566
2.550
1.733
1.718
1.703
1.689
S
O
OH
7.5 7.0 ppm
4.4 4.2 ppm
2.5 2.0 ppm
10 9 8 7 6 5 4 3 2 1 ppm
2.00
1.02
1.03
4.05
1.02
1.00
1.04
1.06
2.09
1.04
2.00

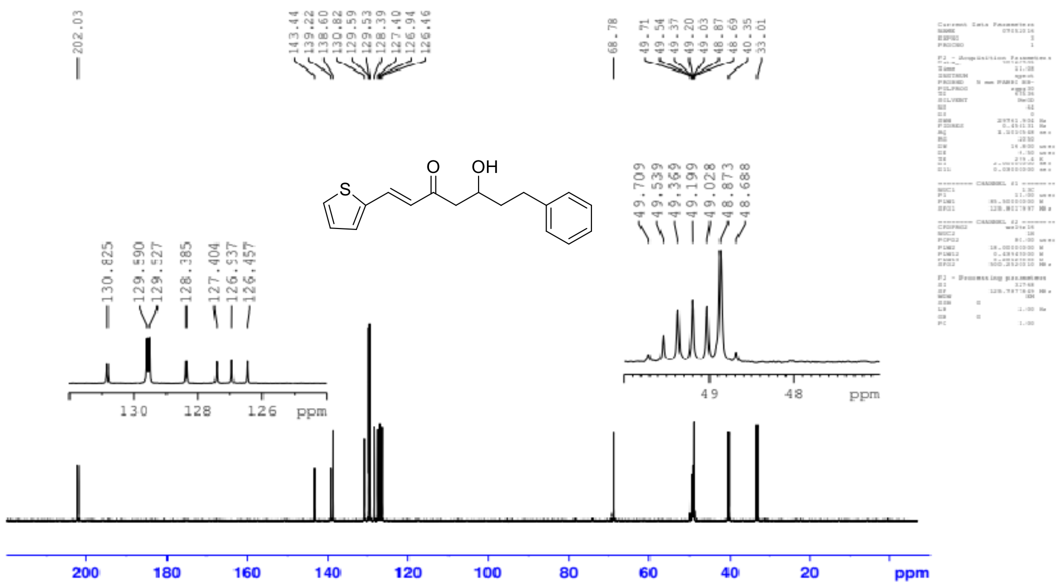

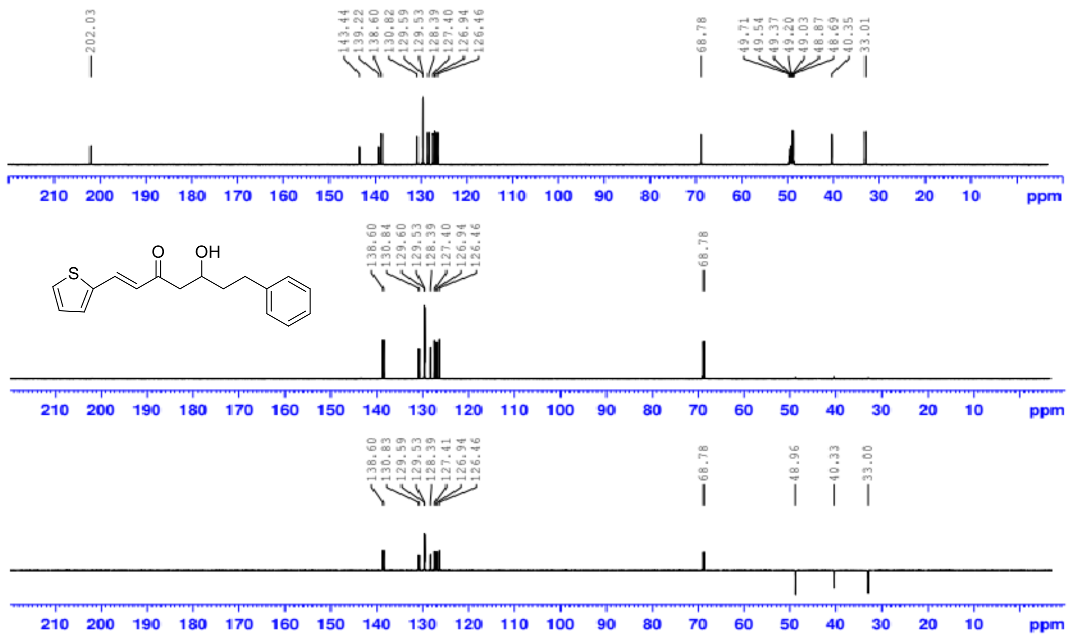

Compound 14a

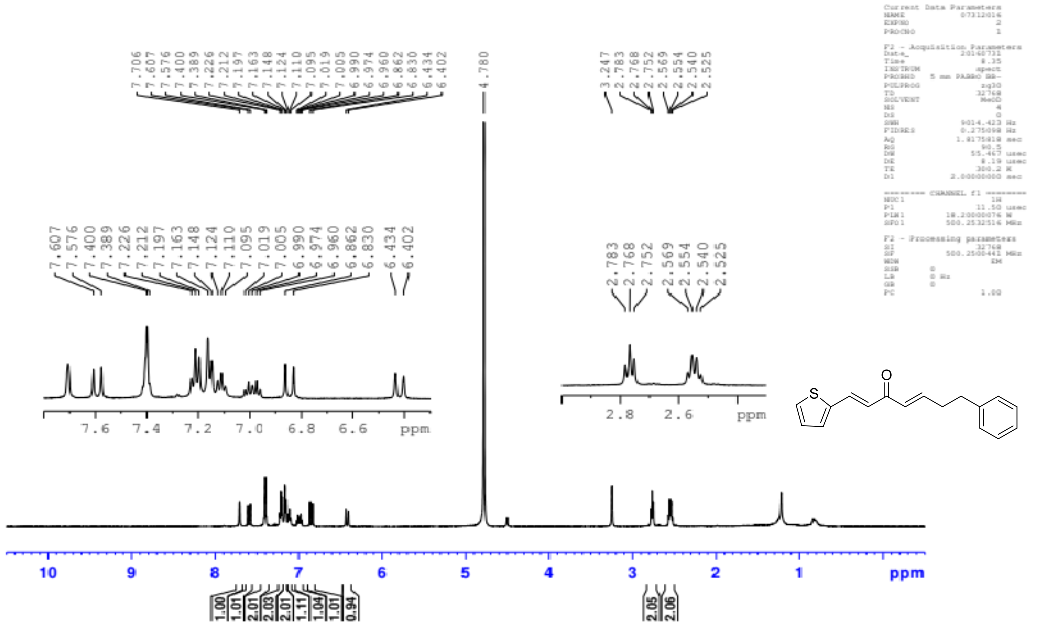
7.706 7.607 7.576 7.400 7.389 7.226 7.212 7.197 7.163 7.148 7.124 7.110 7.095 7.019 7.005 6.990 6.974 6.960 6.862 6.830 6.434 6.402
4.780
3.247 2.783 2.768 2.752 2.569 2.554 2.540 2.525
S
O
ppm

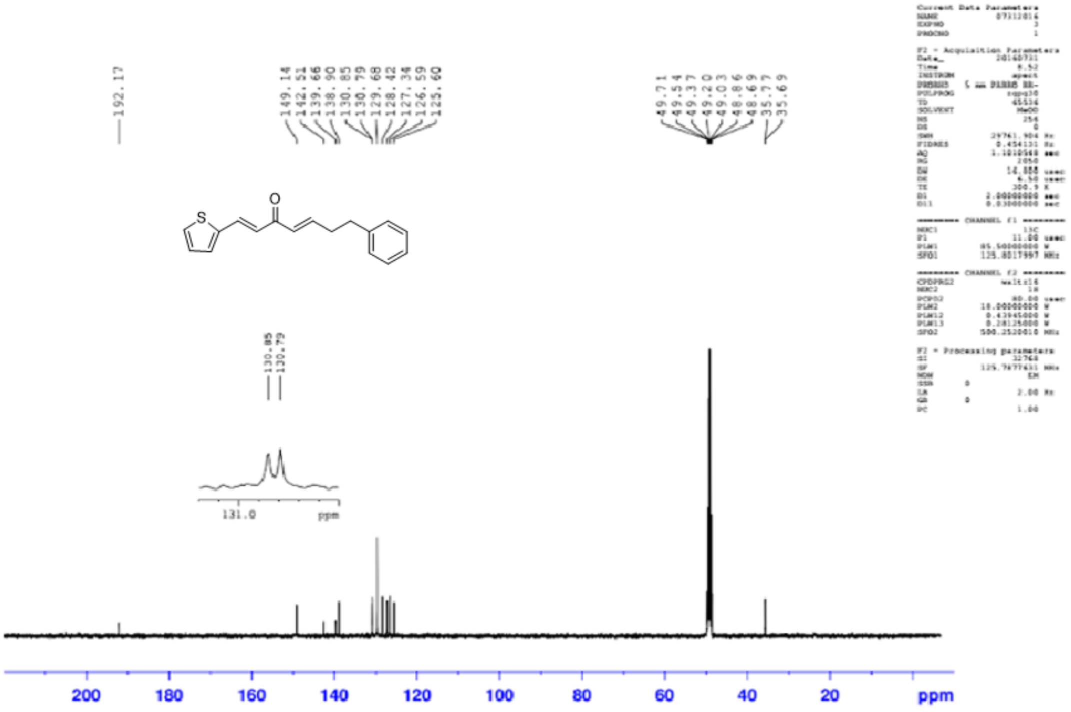
192.17
149.14 142.51 139.66 138.90 130.85 130.79 129.68 128.42 127.34 126.59 125.60
35.77 35.69
S
O
ppm

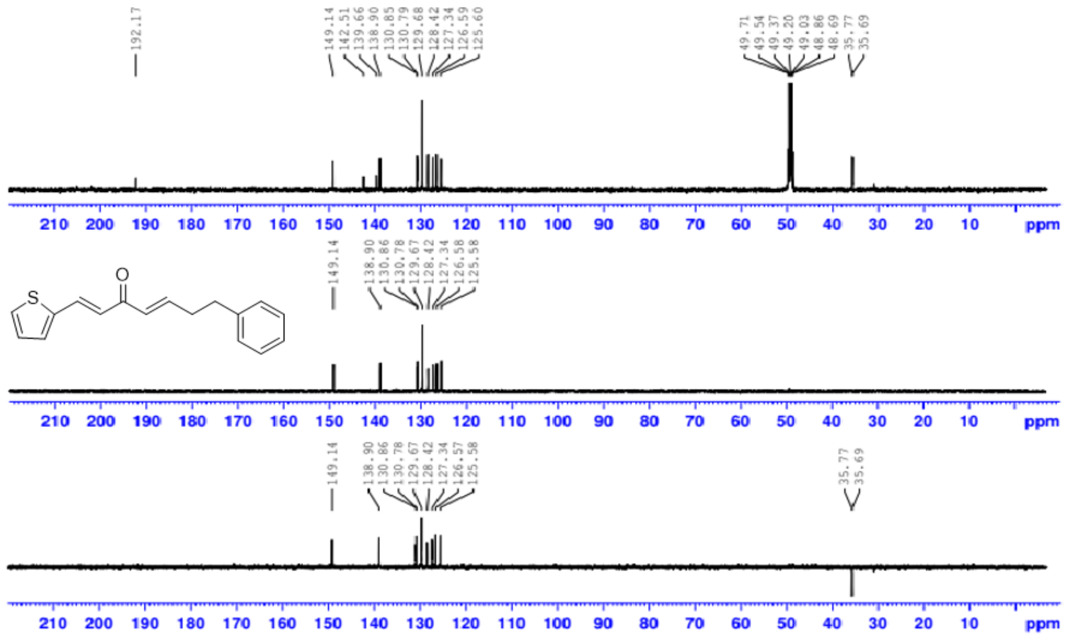

Compound 15

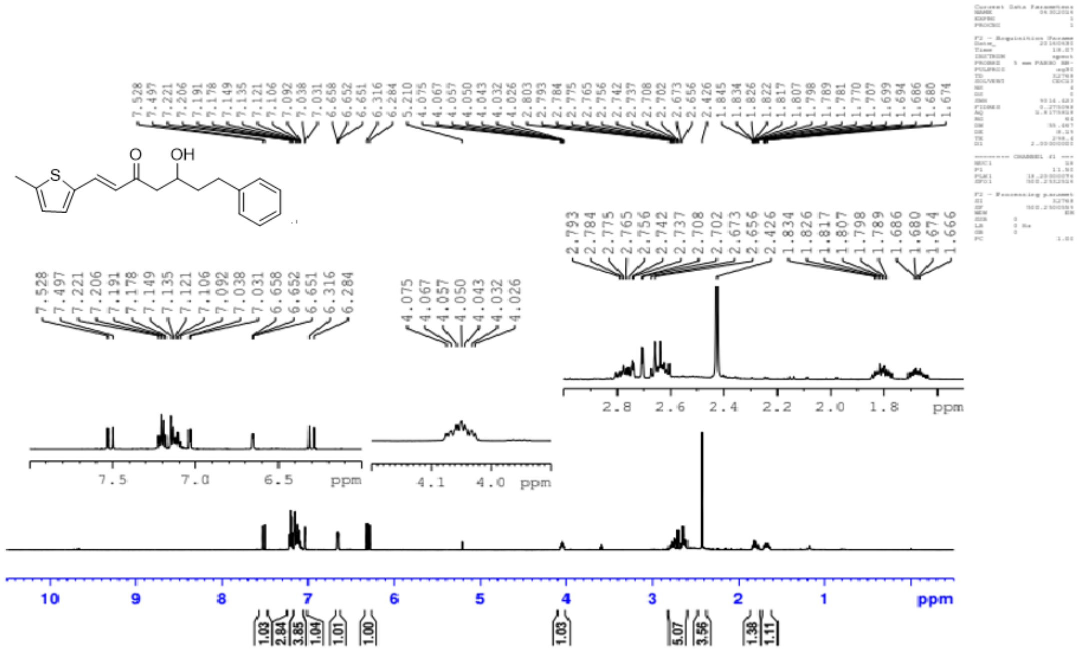

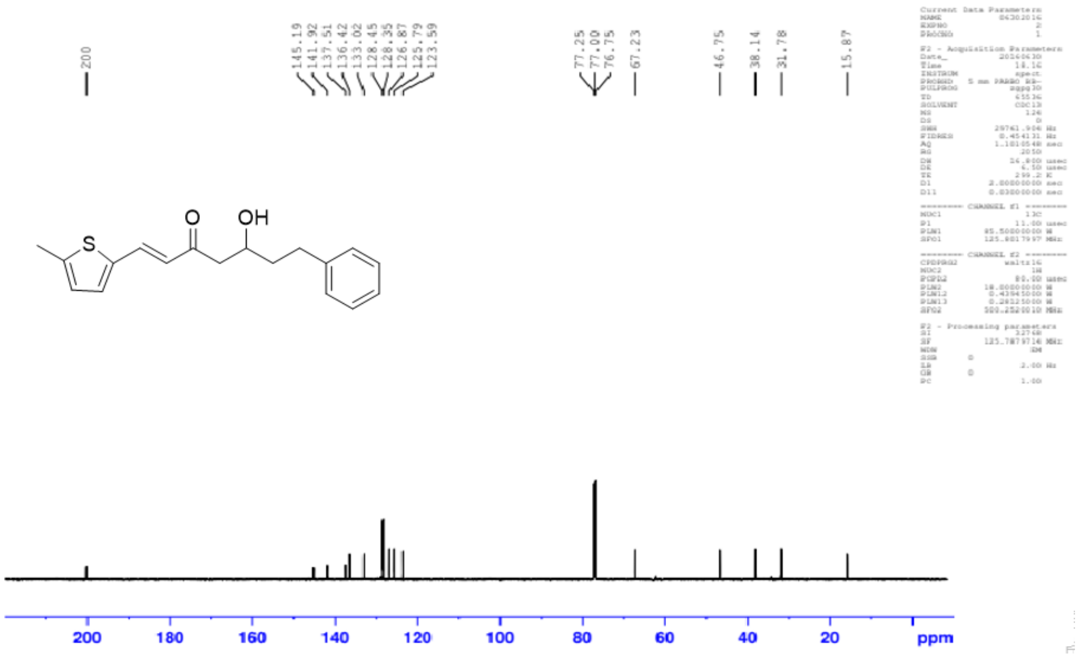
200
145.19
141.92
137.51
136.42
133.02
128.45
128.35
126.87
125.79
123.59
77.25
77.00
76.75
67.23
46.75
38.14
31.78
15.87
O
OH
S
ppm

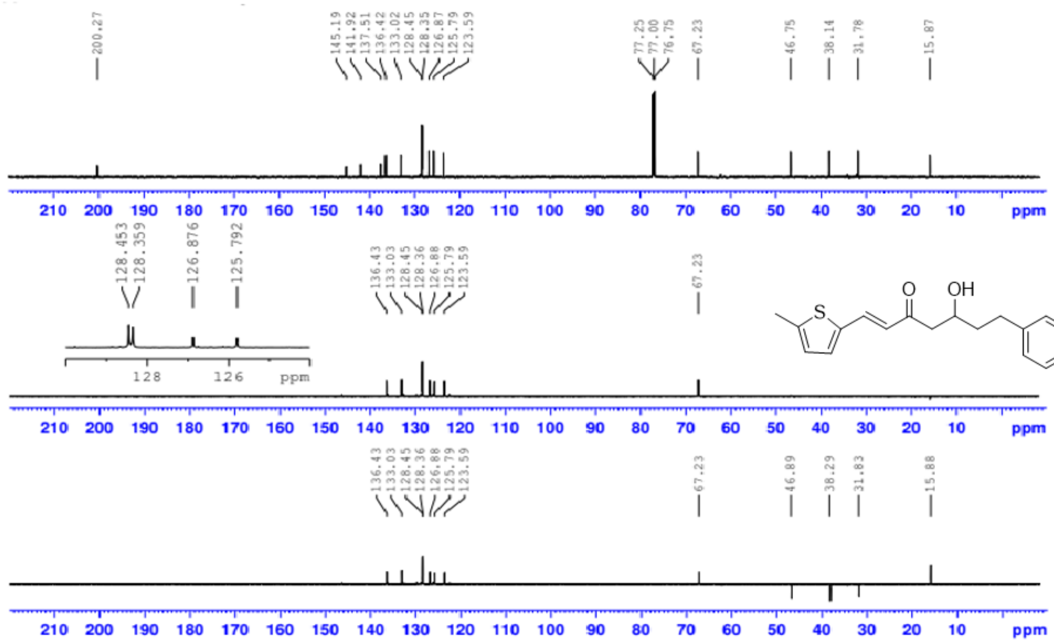
200.27
145.19
141.92
137.51
136.42
133.02
128.45
128.35
126.87
125.79
123.59
77.25
77.00
76.75
67.23
46.75
38.14
31.78
15.87
128.453
128.359
126.876
125.792
136.43
133.03
128.45
128.36
126.88
125.79
123.59
67.23
46.89
38.29
31.83
15.88
O
OH
S
ppm

Compound 16

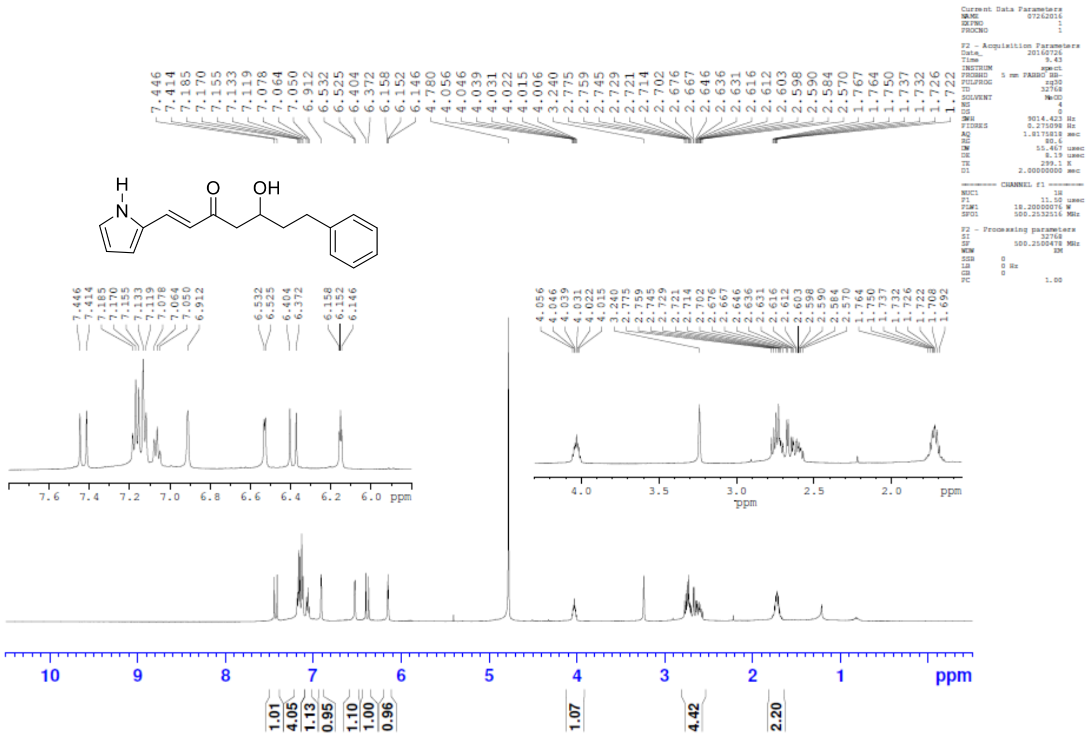

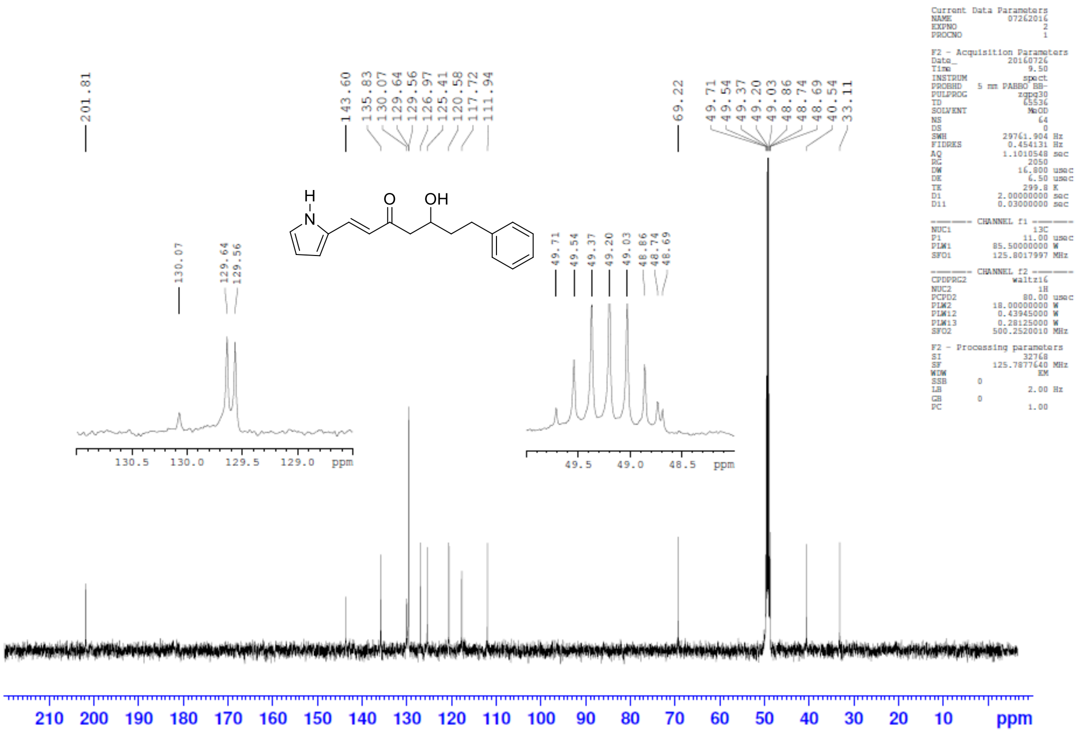

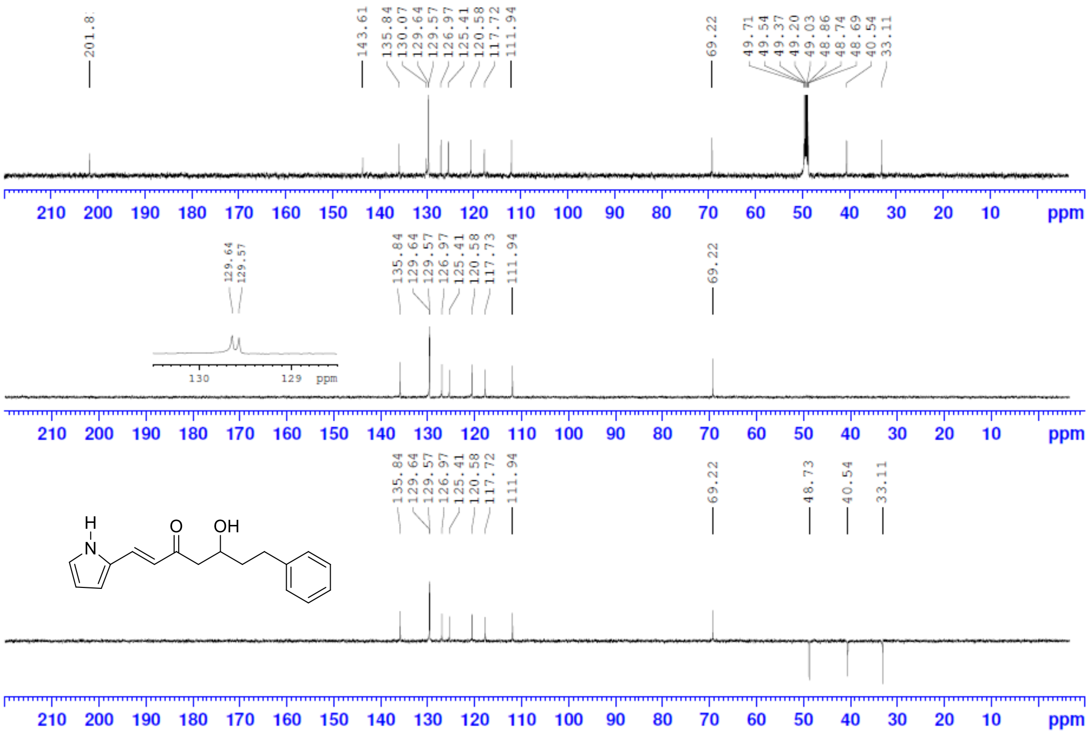
201.8
143.61
135.84
130.07
129.64
129.57
126.97
125.41
120.58
117.72
111.94
69.22
49.71
49.54
49.37
49.20
49.03
48.86
48.74
48.69
40.54
33.11
129.64
129.57
130
129
117.73
48.73
H
N
O
OH
ppm

Compound 17

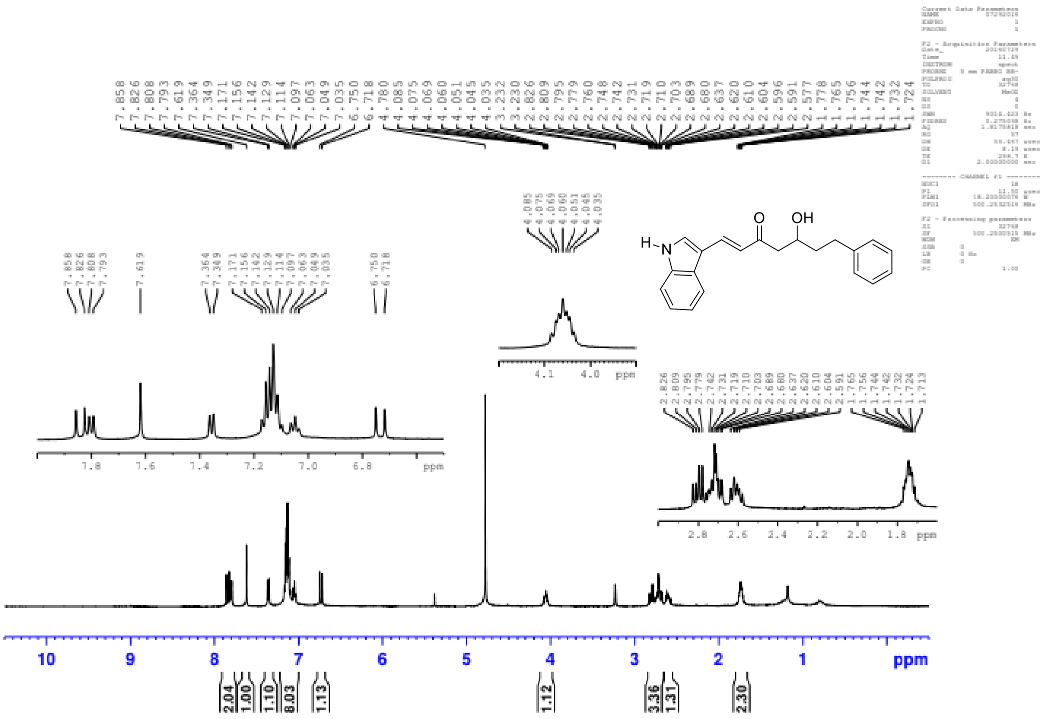
7.858
7.826
7.808
7.793
7.619
7.364
7.349
7.171
7.156
7.142
7.129
7.114
7.097
7.063
7.049
7.035
6.750
6.718
4.085
4.075
4.069
4.060
4.051
4.045
4.035
3.232
2.826
2.809
2.795
2.779
2.760
2.748
2.742
2.731
2.719
2.710
2.703
2.689
2.680
2.637
2.620
2.610
2.604
2.596
2.591
2.577
1.778
1.765
1.756
1.744
1.742
1.732
1.724
H
N
O
OH
ppm
2.04
1.00
1.10
8.03
1.13
1.12
3.36
1.31
2.30

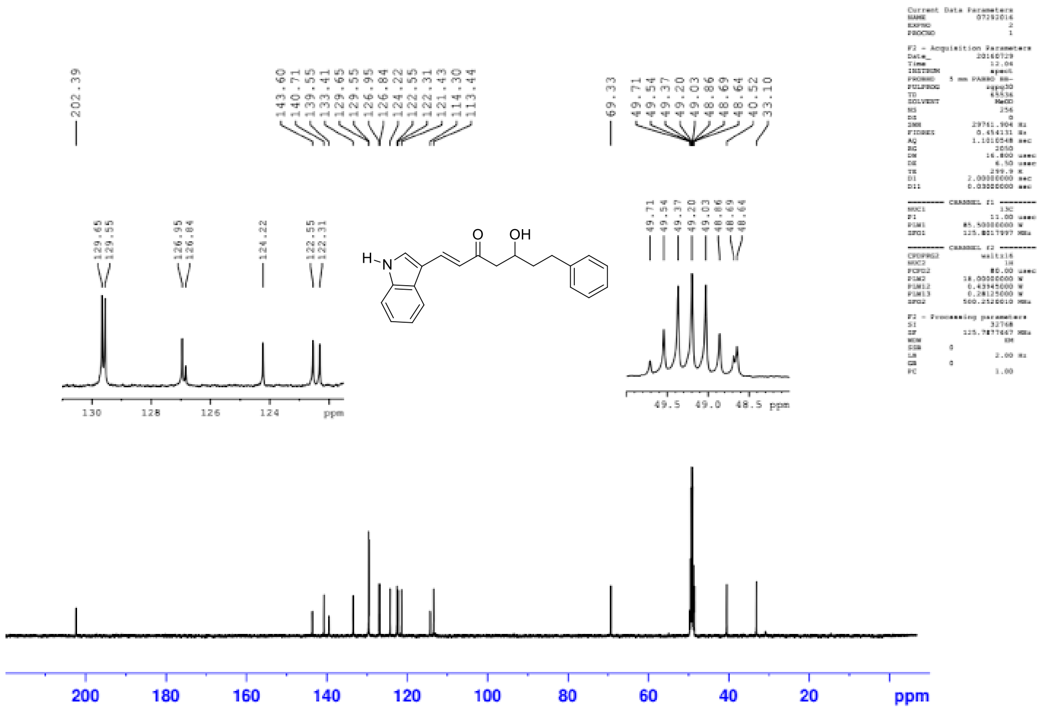

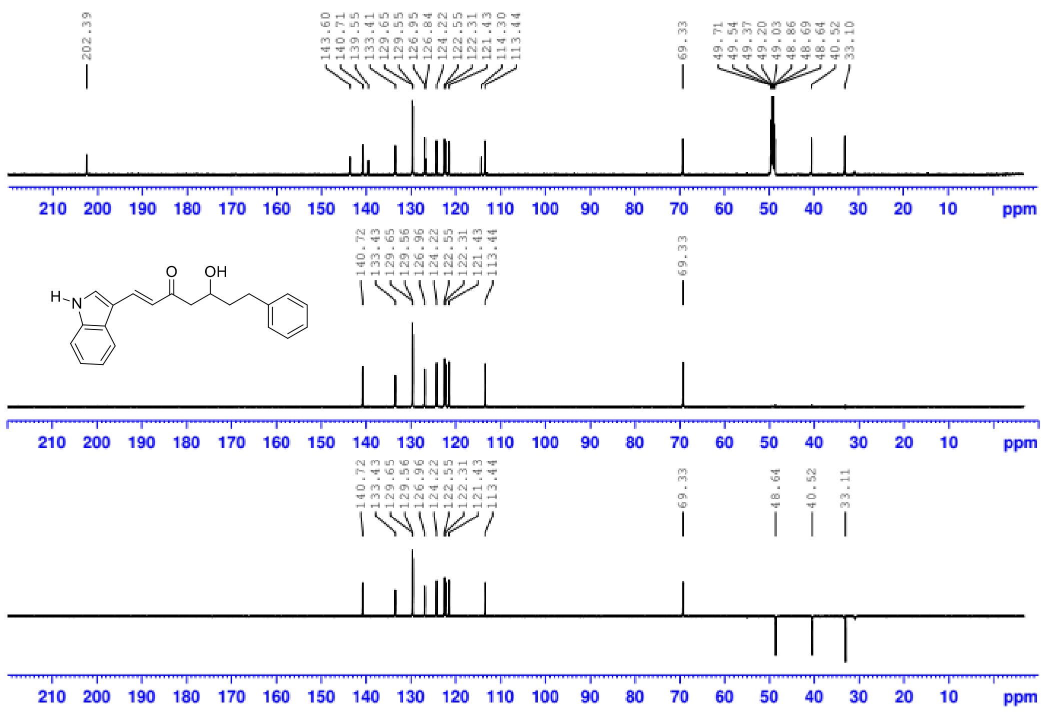

Compound 18

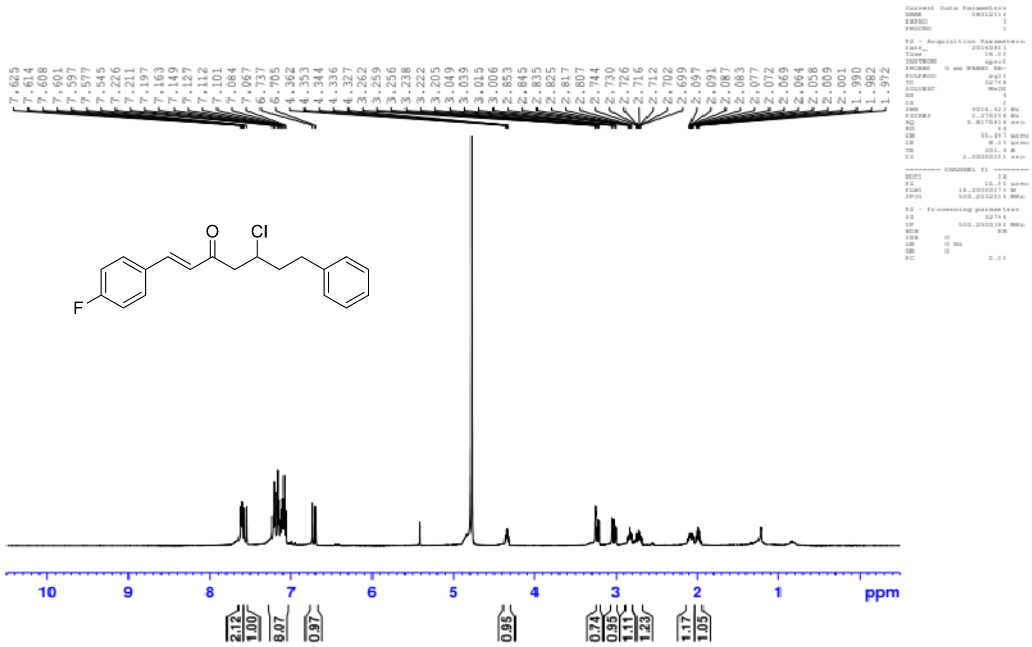

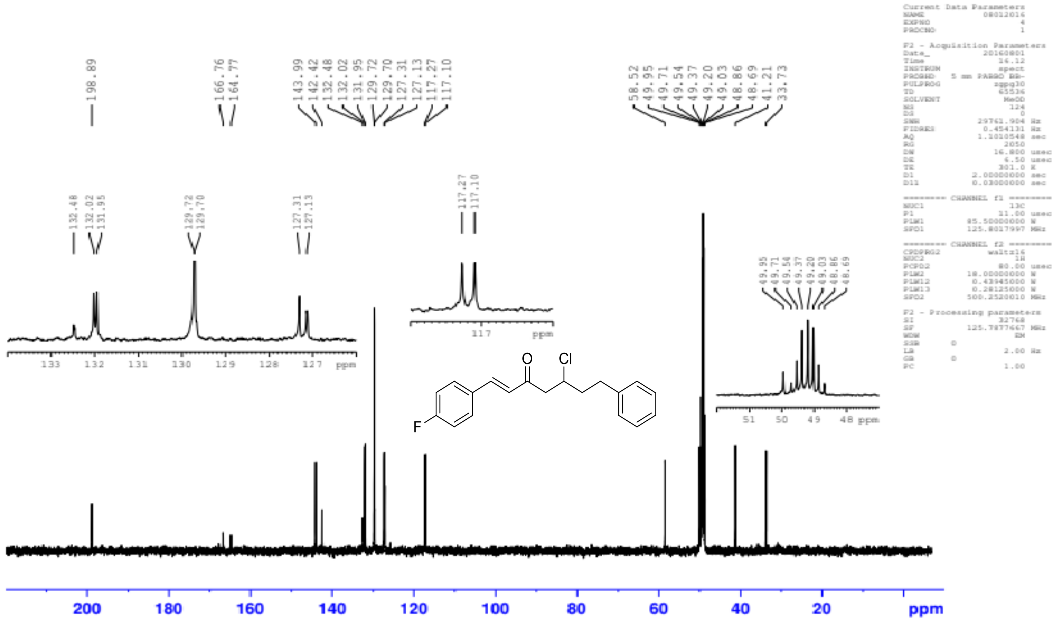

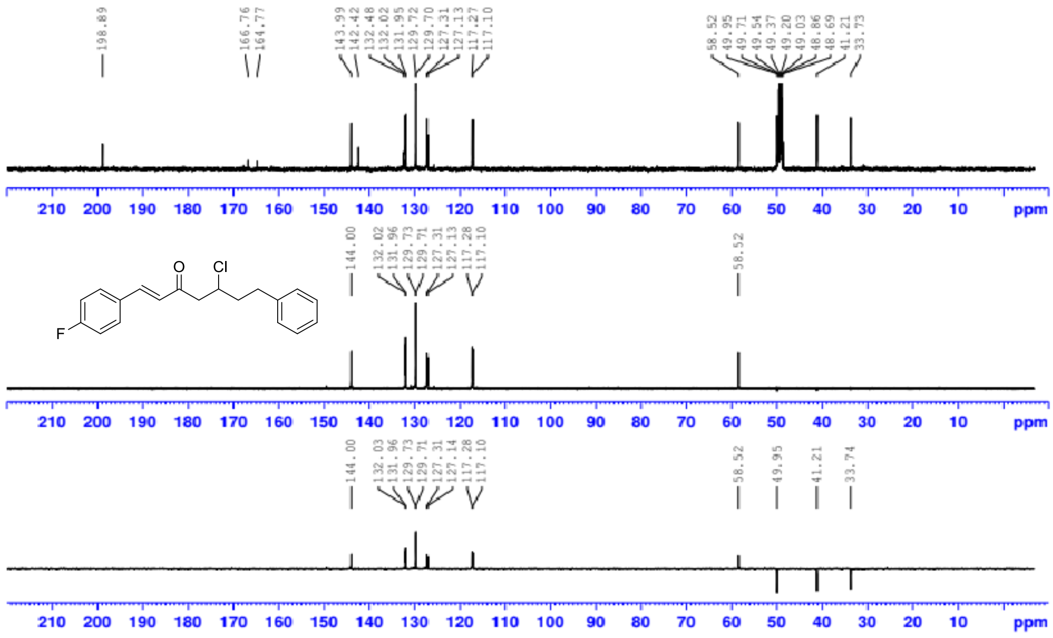
198.89
166.76
164.77
143.99 142.42 132.48 132.02 131.95 129.72 129.70 127.31 127.13 117.27 117.10
58.52 49.95 49.71 49.54 49.37 49.20 49.03 48.86 48.69 41.21 33.73
O
Cl
F
144.00 132.02 131.96 129.73 129.71 127.31 127.13 117.28 117.10
58.52
144.00 132.03 131.96 129.73 129.71 127.31 127.14 117.28 117.10
58.52
49.95
41.21
33.74
ppm

Compound 20

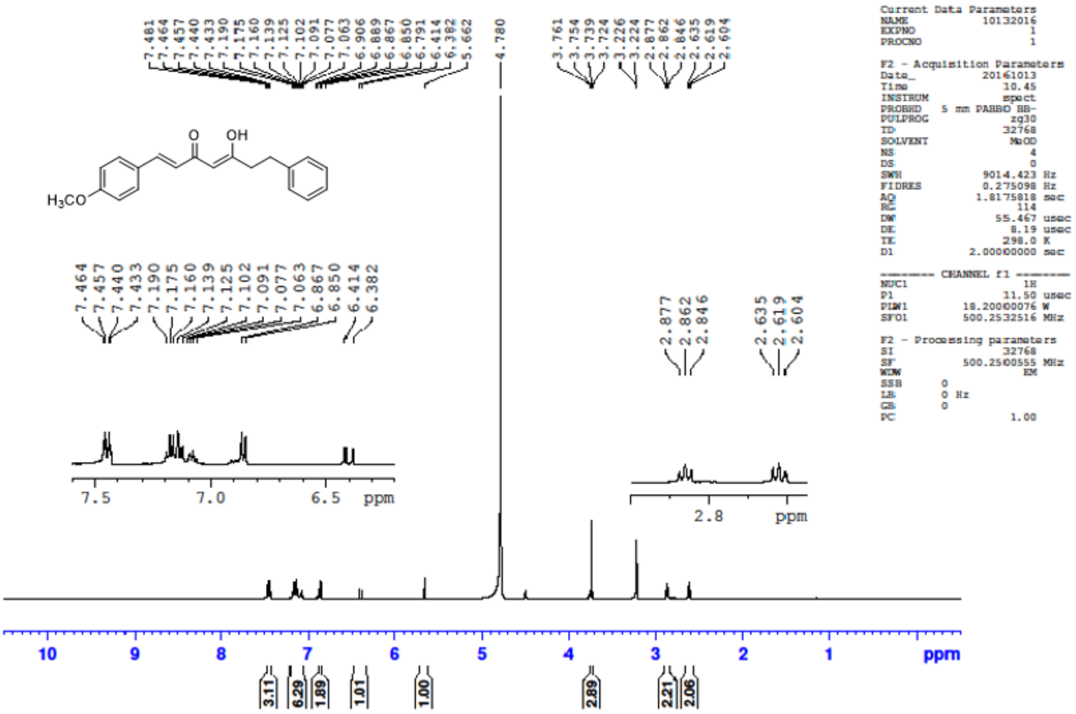
7.481 7.464 7.457 7.440 7.433 7.190 7.175 7.160 7.139 7.125 7.102 7.091 7.077 7.063 6.906 6.889 6.867 6.850 6.791 6.414 6.382 5.662
4.780
3.761 3.754 3.739 3.724 3.226 3.224 2.877 2.862 2.846 2.635 2.619 2.604
O
OH
H3CO
Current Data Parameters
NAME 10132016
EXPNO 1
PROCNO 1
F2 - Acquisition Parameters
Date_ 20161013
Time 10.45
INSTRUM spect
PROBHD 5 mm PABBO BB-
PULPROG zg30
TD 32768
SOLVENT MeOD
NS 4
DS 0
SWH 9014.423 Hz
FIDRES 0.275098 Hz
AQ 1.8175818 sec
RG 114
DW 55.467 usec
DE 8.19 usec
TE 298.0 K
D1 2.00000000 sec
CHANNEL f1
NUC1 1H
P1 11.50 usec
PLW1 18.20000076 W
SFO1 500.2532516 MHz
F2 - Processing parameters
SI 32768
SF 500.2500555 MHz
WDW EM
SSB 0
LB 0 Hz
GB 0
PC 1.00
ppm
3.11
6.29
1.89
1.01
1.00
2.89
2.21
2.06

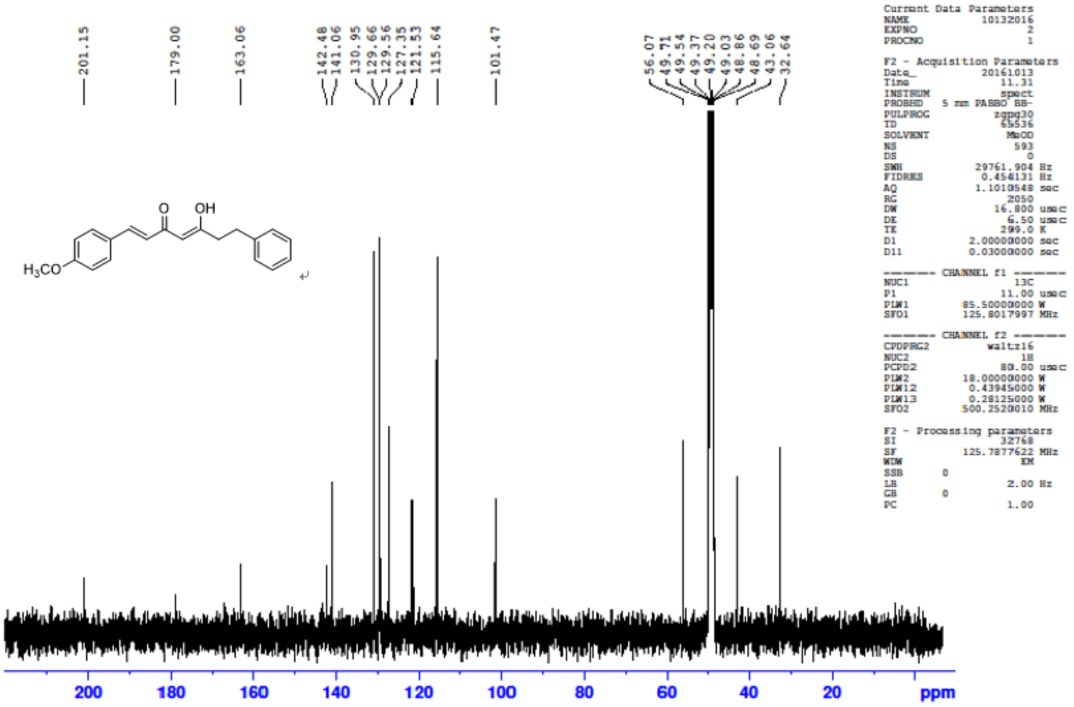
201.15
179.00
163.06
142.48
141.06
130.95
129.66
129.56
127.35
121.53
115.64
101.47
56.07
49.71
49.54
49.37
49.20
49.03
48.86
48.69
43.06
32.64
O OH
H3CO
Current Data Parameters
NAME 10132016
EXPNO 2
PROCNO 1
F2 - Acquisition Parameters
Date_ 20161013
Time 11.31
INSTRUM spect
PROBHD 5 mm PABBO BB-
PULPROG zgpg30
TD 65536
SOLVENT MeOD
NS 593
DS 0
SWH 29761.904 Hz
FIDRES 0.454131 Hz
AQ 1.1010548 sec
RG 2050
DW 16.800 usec
DE 6.50 usec
TE 299.0 K
D1 2.00000000 sec
D11 0.03000000 sec
CHANNEL f1
NUC1 13C
P1 11.00 usec
PLW1 85.50000000 W
SFO1 125.8017997 MHz
CHANNEL f2
CPDPRG2 waltz16
NUC2 1H
PCPD2 80.00 usec
PLW2 18.00000000 W
PLW12 0.43945000 W
PLW13 0.28125000 W
SFO2 500.2520010 MHz
F2 - Processing parameters
SI 32768
SF 125.7877622 MHz
WDW EM
SSB 0
LB 2.00 Hz
GB 0
PC 1.00
200
180
160
140
120
100
80
60
40
20
ppm

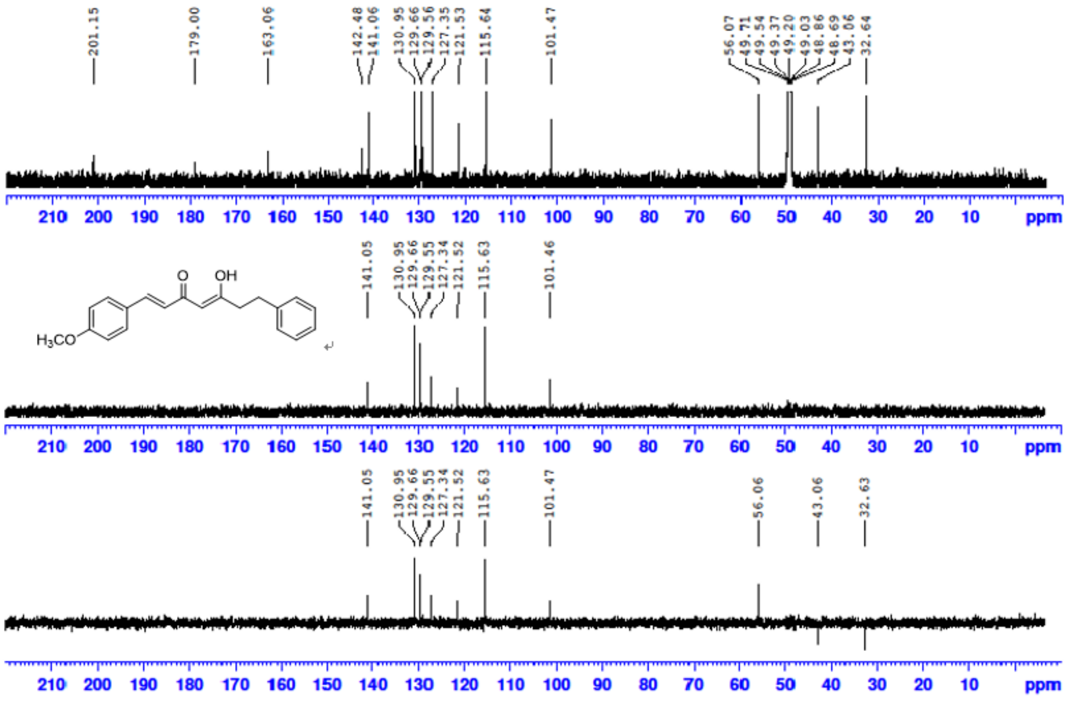
201.15
179.00
163.06
142.48
141.06
130.95
129.66
129.56
127.35
121.53
115.64
101.47
56.07
49.71
49.54
49.37
49.20
49.03
48.86
48.69
43.06
32.64
210 200 190 180 170 160 150 140 130 120 110 100 90 80 70 60 50 40 30 20 10 ppm
O OH
H3CO
141.05
130.95
129.66
129.55
127.34
121.52
115.63
101.46
210 200 190 180 170 160 150 140 130 120 110 100 90 80 70 60 50 40 30 20 10 ppm
141.05
130.95
129.66
129.55
127.34
121.52
115.63
101.47
56.06
43.06
32.63
210 200 190 180 170 160 150 140 130 120 110 100 90 80 70 60 50 40 30 20 10 ppm

Compound 21

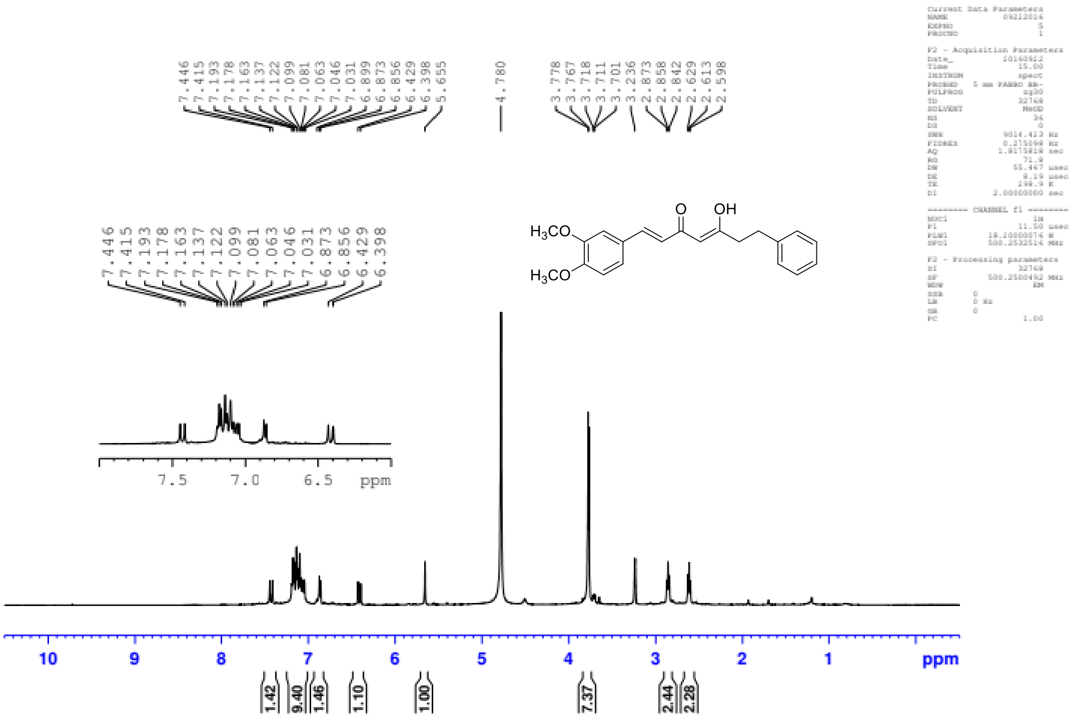
7.446 7.415 7.193 7.178 7.163 7.137 7.122 7.099 7.081 7.063 7.046 7.031 6.899 6.873 6.856 6.429 6.398 5.655
4.780
3.778 3.767 3.718 3.711 3.701 3.236 2.873 2.858 2.842 2.629 2.613 2.598
7.446 7.415 7.193 7.178 7.163 7.137 7.122 7.099 7.081 7.063 7.046 7.031 6.873 6.856 6.429 6.398
7.5 7.0 6.5 ppm
O OH
H3CO
H3CO
10 9 8 7 6 5 4 3 2 1 ppm
1.42 9.40 1.46 1.10 1.00 7.37 2.44 2.28

Compound 22

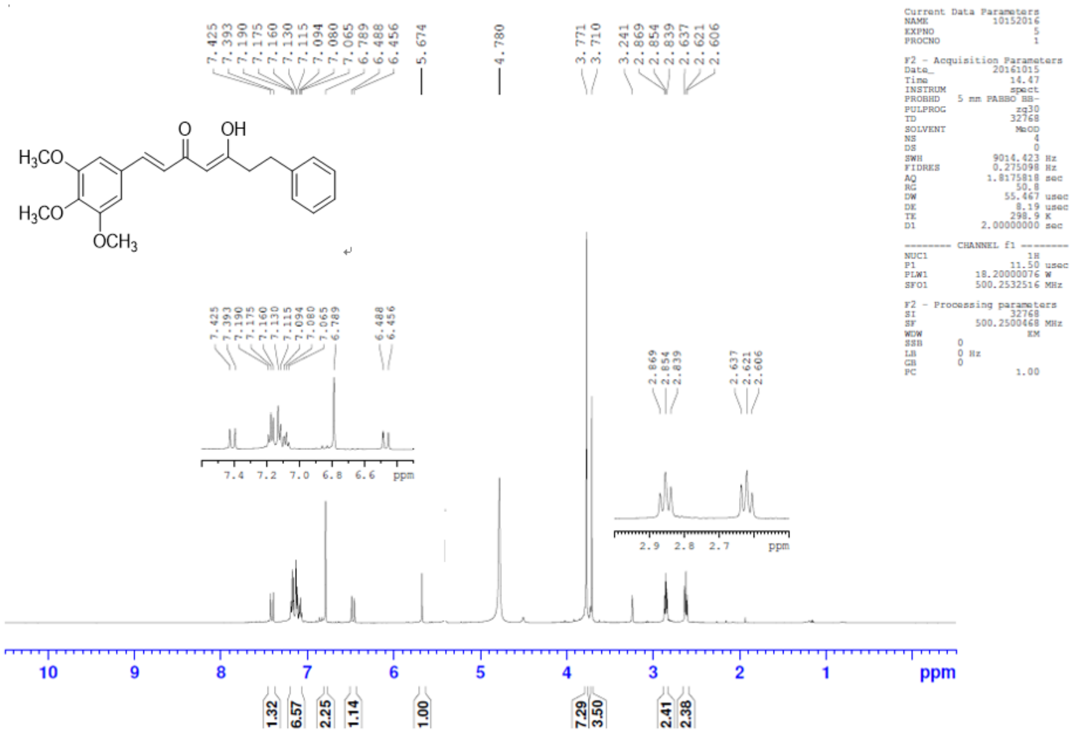
7.425 7.393 7.190 7.175 7.160 7.130 7.115 7.094 7.080 7.065 6.789 6.488 6.456 5.674
4.780
3.771 3.710
3.241 2.869 2.854 2.839 2.637 2.621 2.606
Current Data Parameters
NAME 10152016
EXPNO 5
PROCNO 1
F2 - Acquisition Parameters
Date_ 20161015
Time 14.47
INSTRUM spect
PROBHD 5 mm PABBO BB-
PULPROG zg30
TD 32768
SOLVENT MeOD
NS 4
DS 0
SWH 9014.423 Hz
FIDRES 0.275098 Hz
AQ 1.8175818 sec
RG 50.8
DW 55.467 usec
DE 8.19 usec
TE 298.9 K
D1 2.00000000 sec
======== CHANNEL f1 ========
NUC1 1H
P1 11.50 usec
PLW1 18.20000076 W
SFO1 500.2532516 MHz
F2 - Processing parameters
SI 32768
SF 500.2500468 MHz
WDW EM
SSB 0
LB 0 Hz
GB 0
PC 1.00
O OH
H3CO
H3CO
OCH3
7.425 7.393 7.190 7.175 7.160 7.130 7.115 7.094 7.080 7.065 6.789 6.488 6.456
7.4 7.2 7.0 6.8 6.6 ppm
2.869 2.854 2.839 2.637 2.621 2.606
2.9 2.8 2.7 ppm
10 9 8 7 6 5 4 3 2 1 ppm
1.32 6.57 2.25 1.14 1.00 7.29 3.50 2.41 2.38

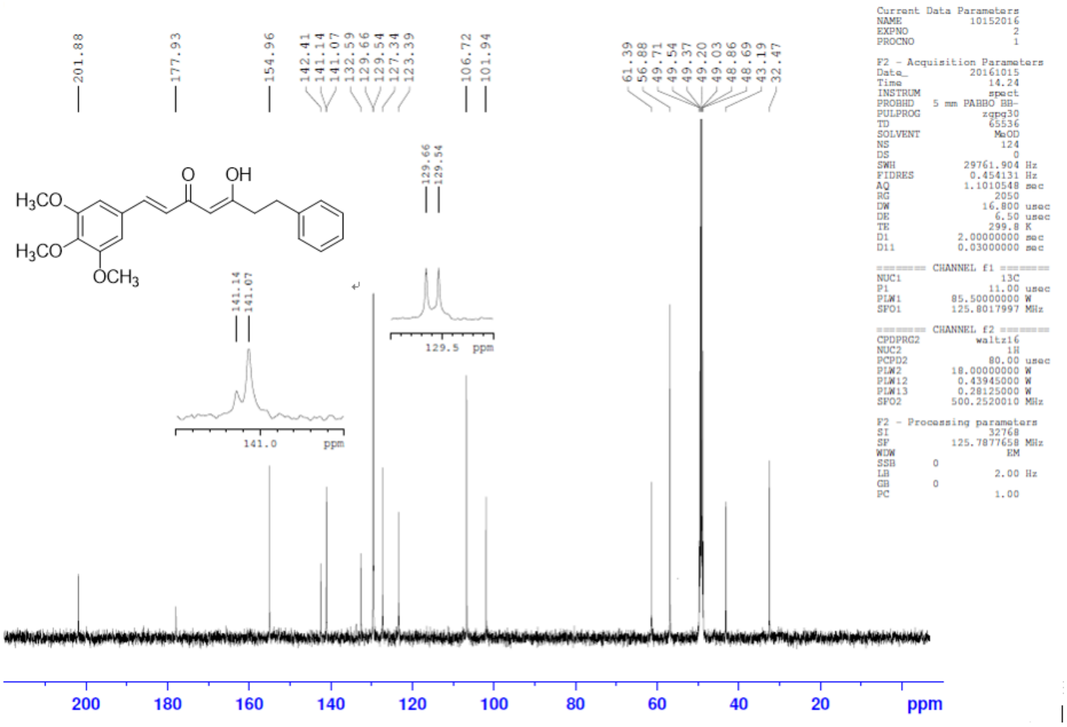
201.88
177.93
154.96
142.41
141.14
141.07
132.59
129.66
129.54
127.34
123.39
106.72
101.94
61.39
56.88
49.71
49.54
49.37
49.20
49.03
48.86
48.69
43.19
32.47
O
OH
H_3CO
H_3CO
OCH_3
141.14
141.07
141.0
ppm
129.66
129.54
129.5
ppm
Current Data Parameters
NAME 10152016
EXPNO 2
PROCNO 1
F2 - Acquisition Parameters
Date_ 20161015
Time 14.24
INSTRUM spect
PROBHD 5 mm PABBO BB-
PULPROG zgpg30
TD 65536
SOLVENT MeOD
NS 124
DS 0
SWH 29761.904 Hz
FIDRES 0.454131 Hz
AQ 1.1010548 sec
RG 2050
DW 16.800 usec
DE 6.50 usec
TE 299.8 K
D1 2.00000000 sec
D11 0.03000000 sec
======= CHANNEL f1 =======
NUC1 13C
P1 11.00 usec
PLW1 85.50000000 W
SFO1 125.8017997 MHz
======= CHANNEL f2 =======
CPDPRG2 waltz16
NUC2 1H
PCPD2 80.00 usec
PLW2 18.00000000 W
PLW12 0.43945000 W
PLW13 0.28125000 W
SFO2 500.2520010 MHz
F2 - Processing parameters
SI 32768
SF 125.7877658 MHz
WDW EM
SSB 0
LB 2.00 Hz
GB 0
PC 1.00
200
180
160
140
120
100
80
60
40
20
ppm

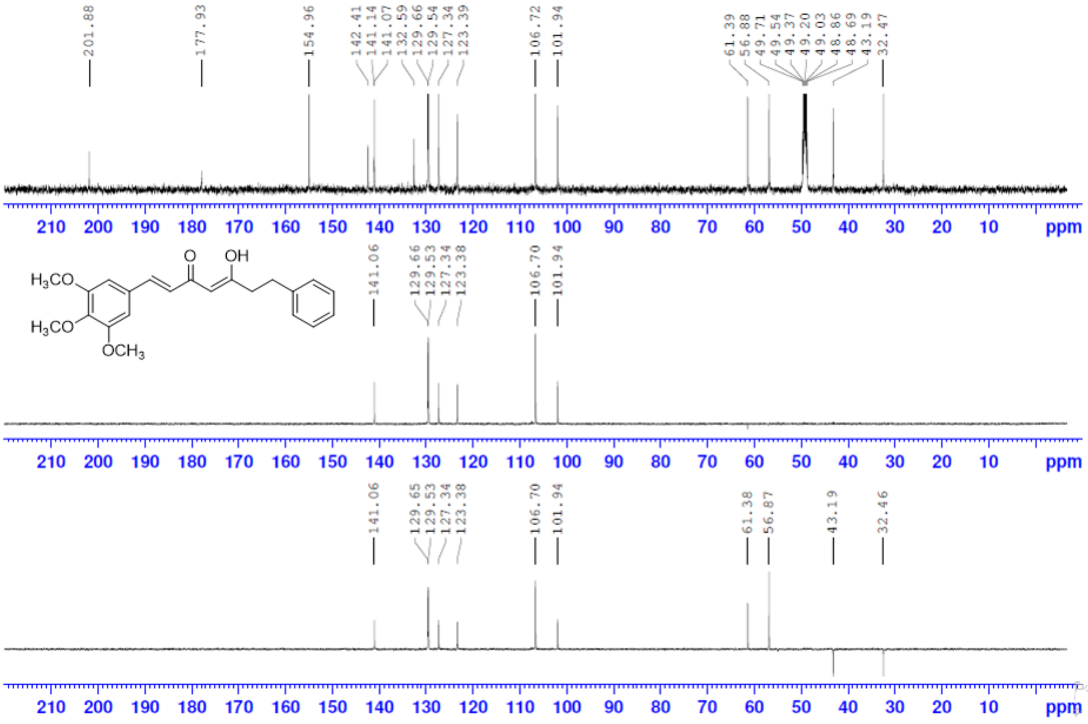
201.88
177.93
154.96
142.41
141.14
141.07
132.59
129.66
129.54
127.34
123.39
106.72
101.94
61.39
56.88
49.71
49.54
49.37
49.20
49.03
48.86
48.69
43.19
32.47
210 200 190 180 170 160 150 140 130 120 110 100 90 80 70 60 50 40 30 20 10 ppm
O
OH
H_3CO
H_3CO
OCH_3
141.06
129.66
129.53
127.34
123.38
106.70
101.94
210 200 190 180 170 160 150 140 130 120 110 100 90 80 70 60 50 40 30 20 10 ppm
141.06
129.65
129.53
127.34
123.38
106.70
101.94
61.38
56.87
43.19
32.46
210 200 190 180 170 160 150 140 130 120 110 100 90 80 70 60 50 40 30 20 10 ppm

Compound 23

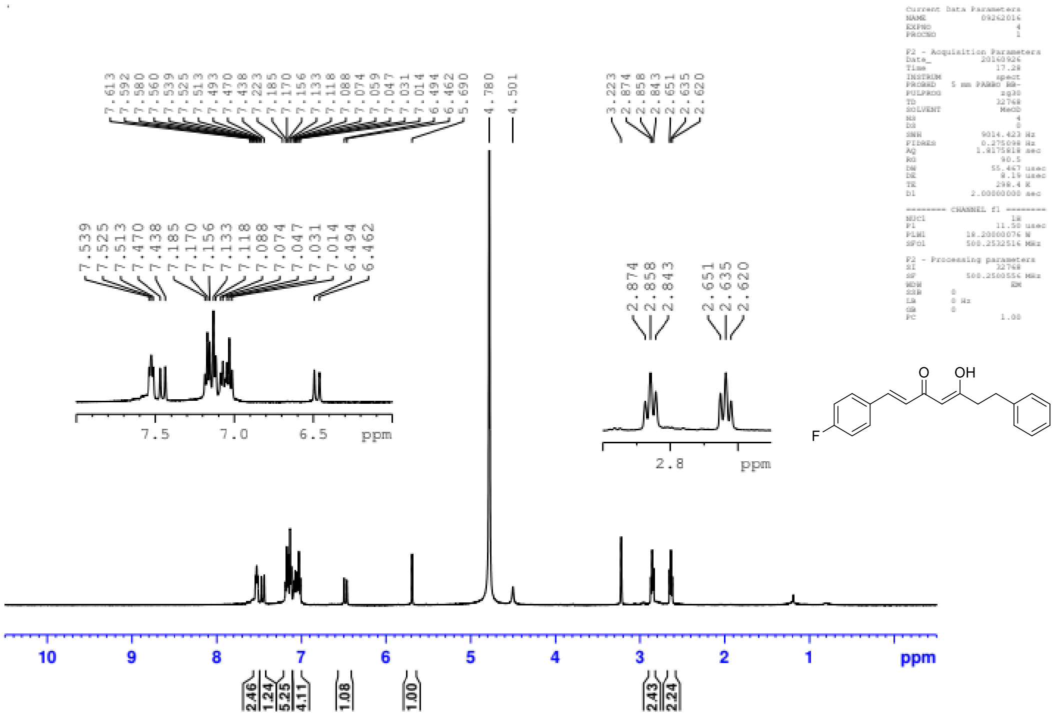
O OH
F
ppm
2.46 1.24 5.25 4.11 1.08 1.00 2.43 2.24

Compound 24

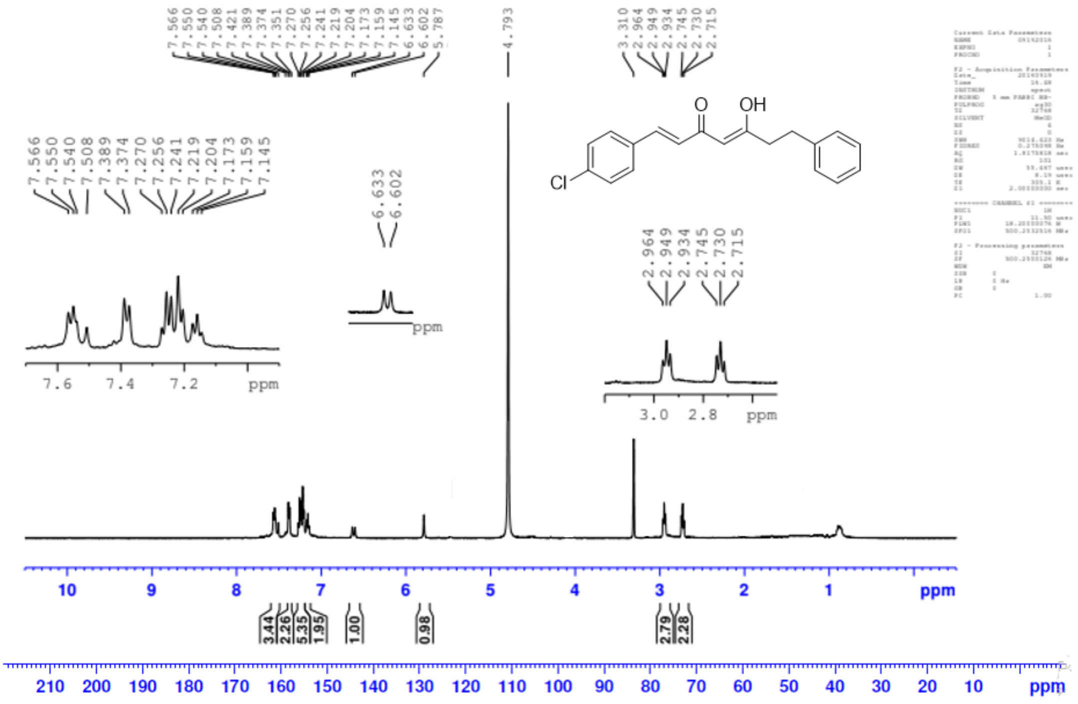
O OH
Cl
ppm
3.44 2.26 5.35 1.95 1.00 0.98 2.79 2.28

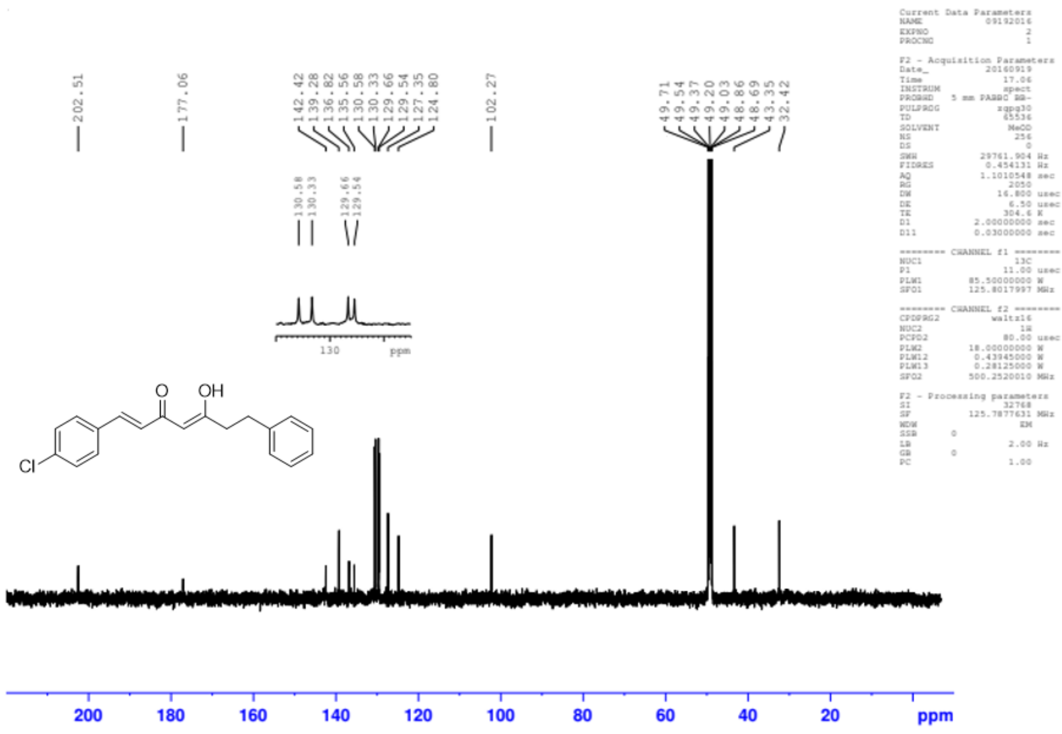
202.51
177.06
142.42
139.28
136.82
135.56
130.58
130.33
129.66
129.54
127.35
124.80
102.27
49.71
49.54
49.37
49.20
49.03
48.86
48.69
43.35
32.42
130.58
130.33
129.66
129.54
130
ppm
O
OH
Cl
ppm

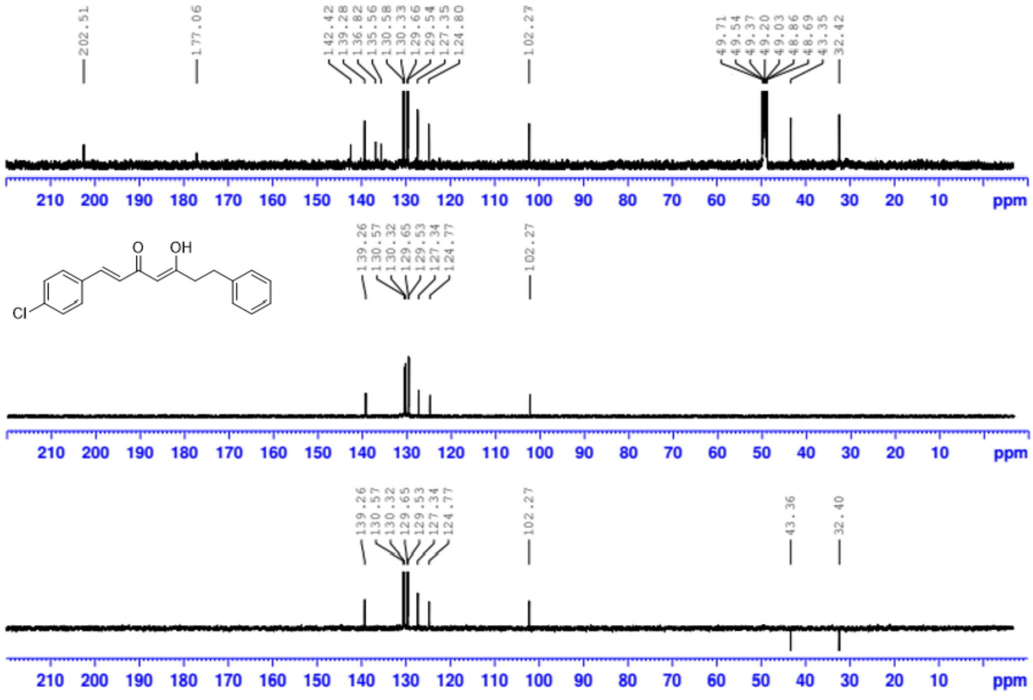
202.51
177.06
142.42
139.28
136.82
135.56
130.58
130.33
129.66
129.54
127.35
124.80
102.27
49.71
49.54
49.37
49.20
49.03
48.86
48.69
43.35
32.42
ppm
O
OH
Cl
139.26
130.57
130.32
129.65
129.53
127.34
124.77
102.27
ppm
139.26
130.57
130.32
129.65
129.53
127.34
124.77
102.27
43.36
32.40
ppm

MD1

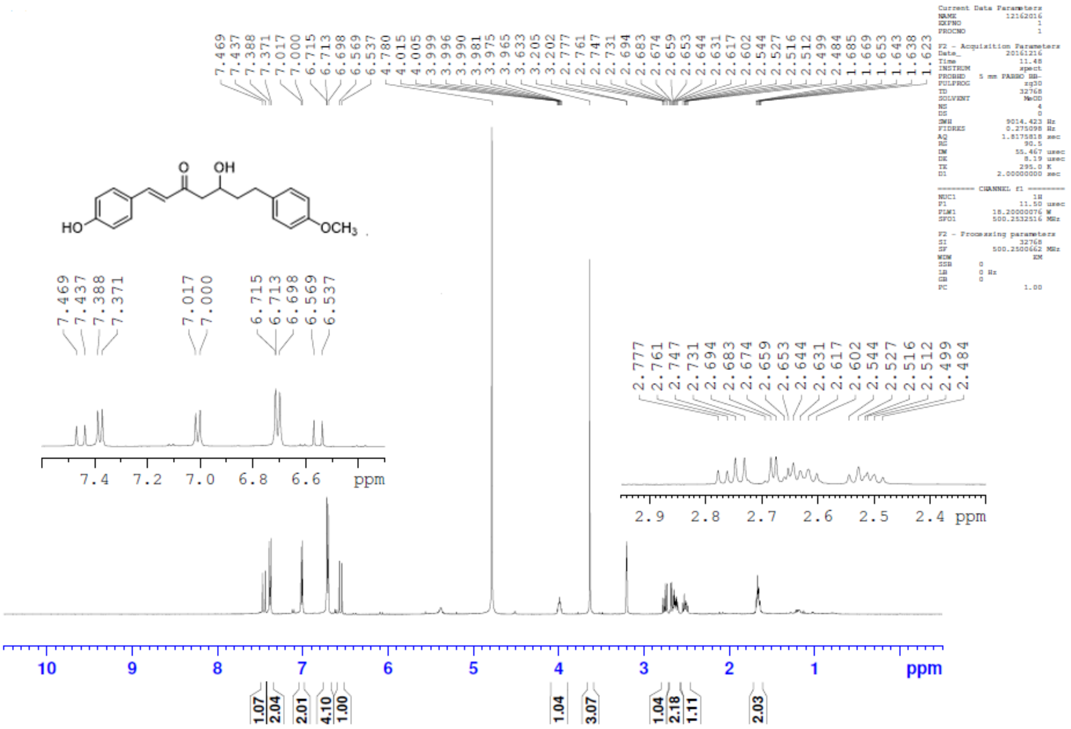
HO
O
OH
OCH3
ppm
10
9
8
7
6
5
4
3
2
1
1.07
2.04
2.01
4.10
1.00
1.04
3.07
1.04
2.18
1.11
2.03

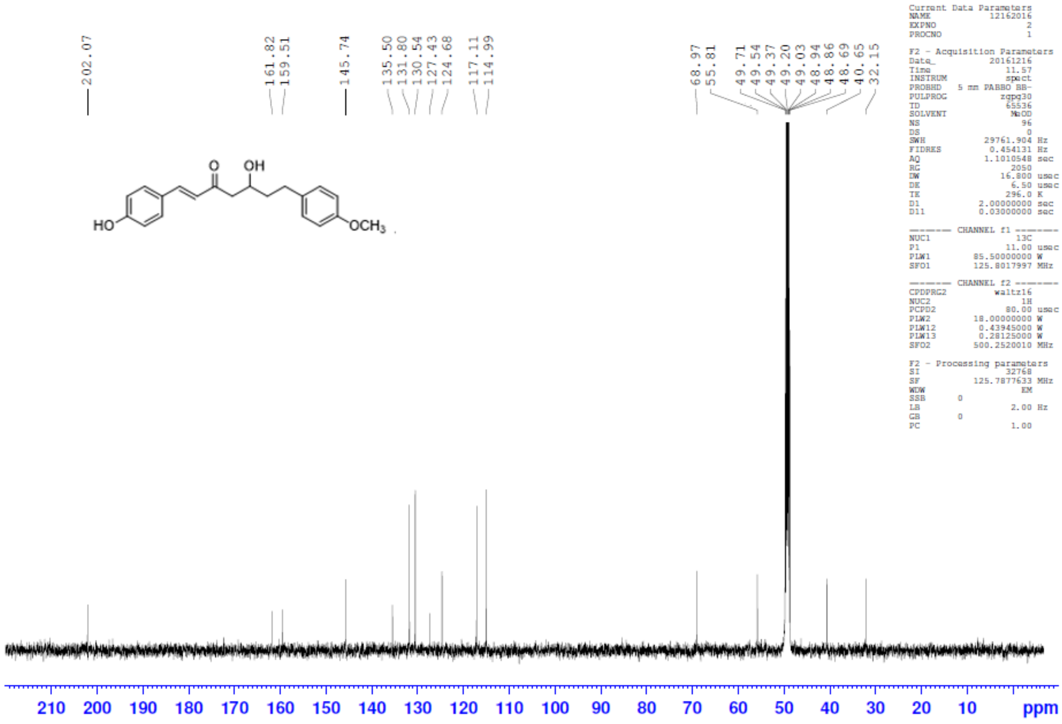
202.07
161.82
159.51
145.74
135.50
131.80
130.54
127.43
124.68
117.11
114.99
68.97
55.81
49.71
49.54
49.37
49.20
49.03
48.86
48.69
40.65
32.15
HO
O
OH
OCH3
210
200
190
180
170
160
150
140
130
120
110
100
90
80
70
60
50
40
30
20
10
ppm

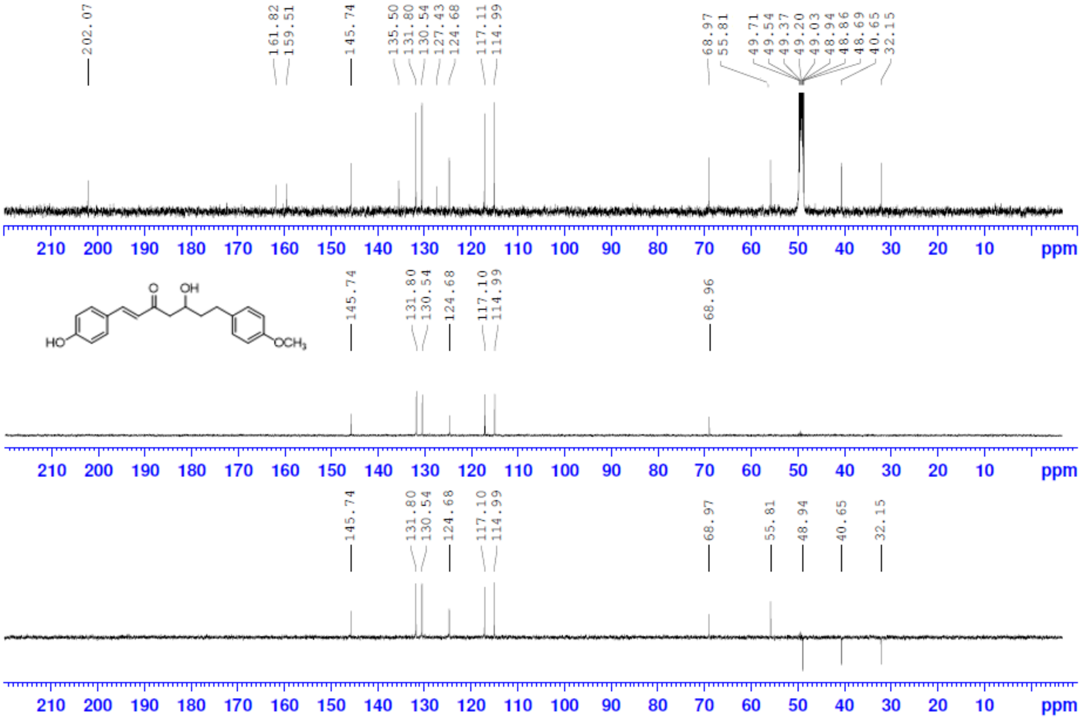
202.07
161.82
159.51
145.74
135.50
131.80
130.54
127.43
124.68
117.11
114.99
68.97
55.81
49.71
49.54
49.37
49.20
49.03
48.94
48.86
48.69
40.65
32.15
145.74
131.80
130.54
124.68
117.10
114.99
68.96
145.74
131.80
130.54
124.68
117.10
114.99
68.97
55.81
48.94
40.65
32.15
ppm

MD1a

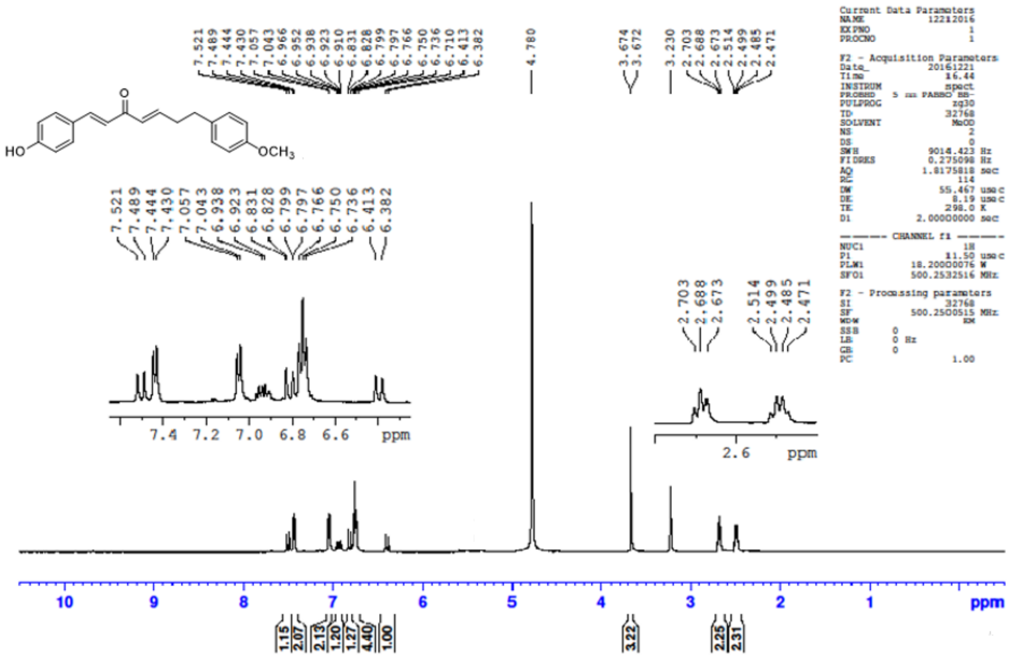
7.521
7.489
7.444
7.430
7.057
7.043
6.966
6.952
6.938
6.923
6.910
6.831
6.828
6.799
6.797
6.766
6.750
6.736
6.710
6.413
6.382
4.780
3.674
3.672
3.230
2.703
2.688
2.673
2.514
2.499
2.485
2.471
ppm
1.15
2.07
2.13
1.20
1.27
4.40
1.00
3.22
2.25
2.31

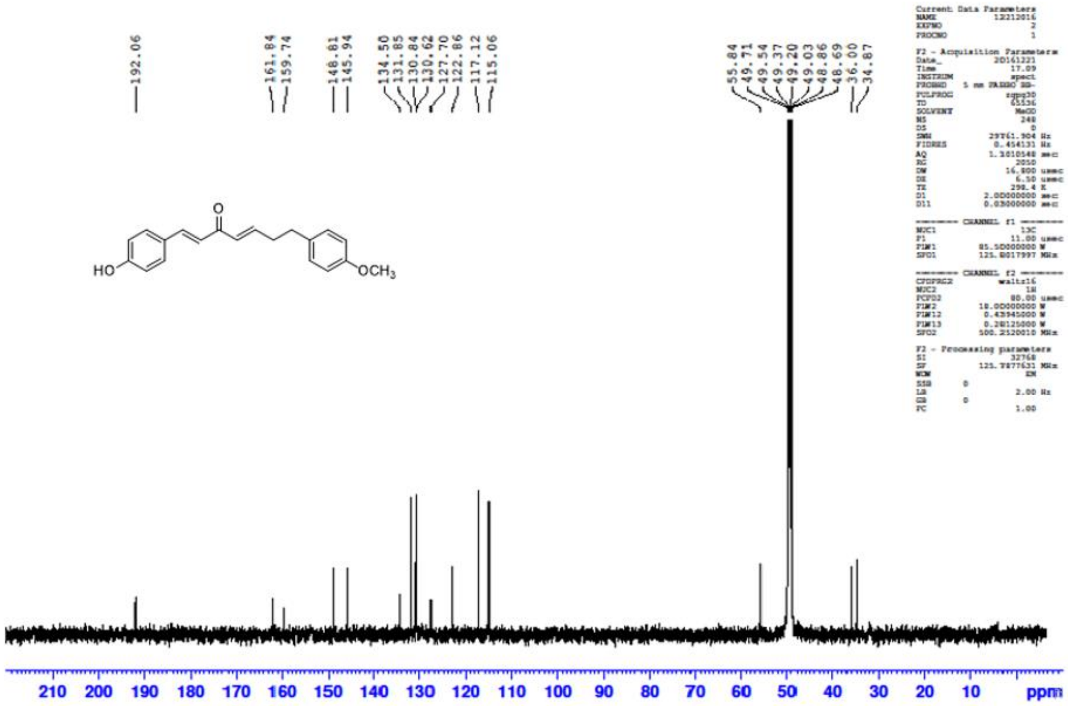
192.06
161.84
159.74
148.81
145.94
134.50
131.85
130.84
130.62
127.70
122.86
117.12
115.06
55.84
49.71
49.54
49.37
49.20
49.03
48.86
48.69
36.00
34.87
HO
O
OCH3
210 200 190 180 170 160 150 140 130 120 110 100 90 80 70 60 50 40 30 20 10 ppm

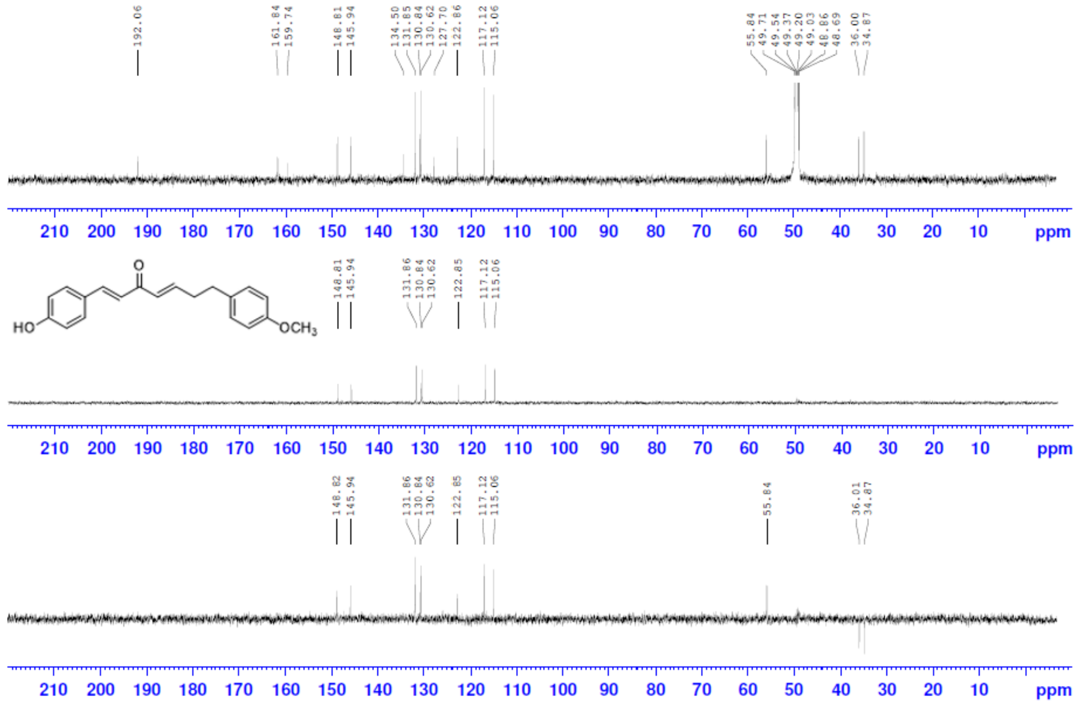
192.06
161.84
159.74
148.81
145.94
134.50
131.85
130.84
130.62
127.70
122.86
117.12
115.06
55.84
49.71
49.54
49.37
49.20
49.03
48.86
48.69
36.00
34.87
210 200 190 180 170 160 150 140 130 120 110 100 90 80 70 60 50 40 30 20 10 ppm
HO
O
OCH3
148.81
145.94
131.86
130.84
130.62
122.85
117.12
115.06
210 200 190 180 170 160 150 140 130 120 110 100 90 80 70 60 50 40 30 20 10 ppm
148.82
145.94
131.86
130.84
130.62
122.85
117.12
115.06
55.84
36.01
34.87
210 200 190 180 170 160 150 140 130 120 110 100 90 80 70 60 50 40 30 20 10 ppm

MD2

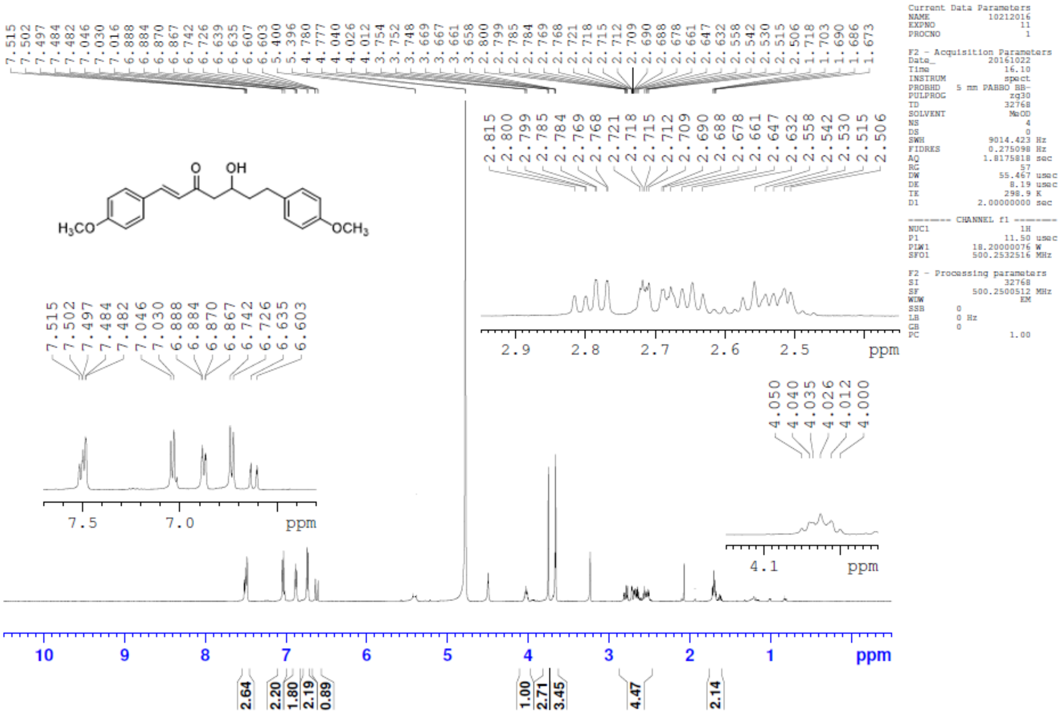
7.515 7.502 7.497 7.484 7.482 7.046 7.030 7.016 6.888 6.884 6.870 6.867 6.742 6.726 6.639 6.635 6.607 6.603 5.400 5.396 4.780 4.777 4.040 4.026 4.012 3.754 3.752 3.748 3.669 3.667 3.661 3.658 2.800 2.799 2.785 2.784 2.769 2.768 2.721 2.718 2.715 2.712 2.709 2.690 2.688 2.678 2.661 2.647 2.632 2.558 2.542 2.530 2.515 2.506 1.718 1.703 1.690 1.686 1.673
OH
O
H3CO
OCH3
ppm
2.64 2.20 1.80 2.19 0.89 1.00 2.71 3.45 4.47 2.14

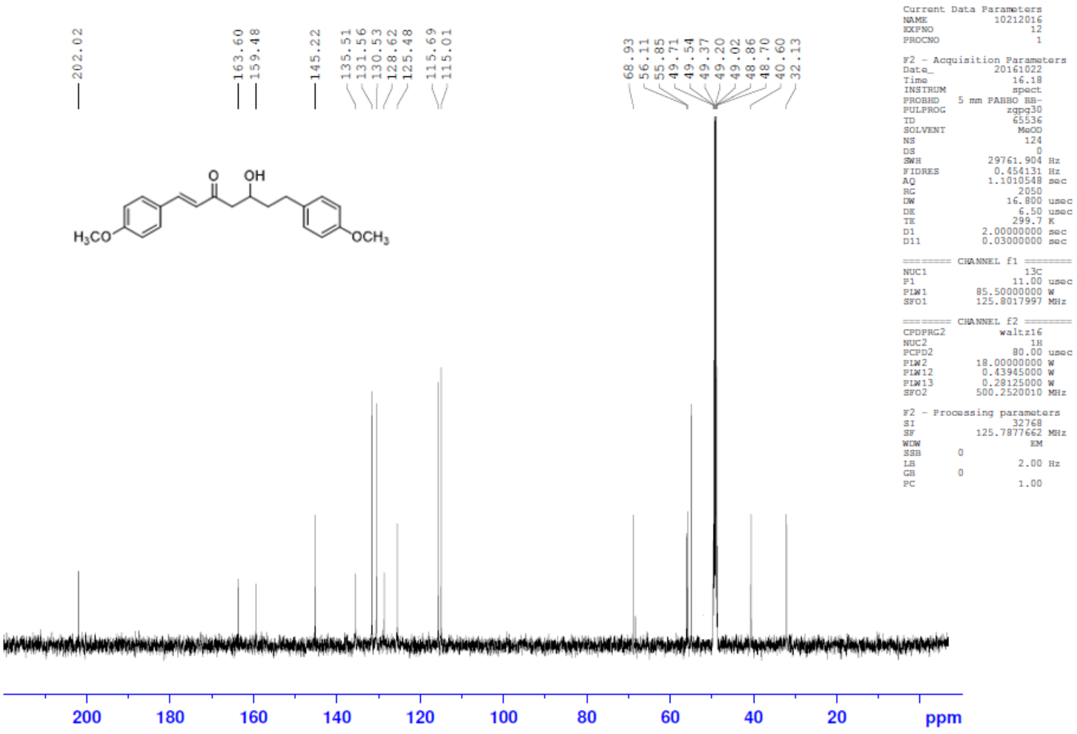
202.02
163.60 159.48
145.22
135.51 131.56 130.53 128.62 125.48
115.69 115.01
68.93 56.11 55.85 49.71 49.54 49.37 49.20 49.02 48.86 48.70 40.60 32.13
O OH
H3CO
OCH3
ppm

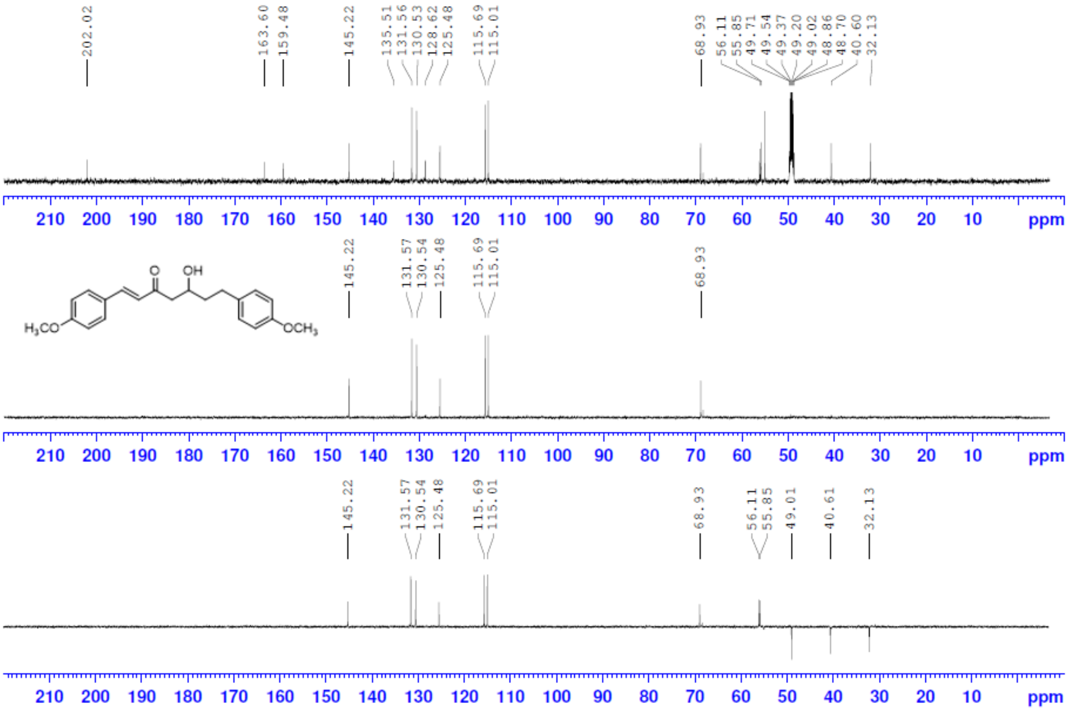
202.02
163.60
159.48
145.22
135.51
131.56
130.53
128.62
125.48
115.69
115.01
68.93
56.11
55.85
49.71
49.54
49.37
49.20
49.02
48.86
48.70
40.60
32.13
H3CO
OCH3
O
OH
131.57
130.54
40.61
32.13
49.01
ppm

MD2a

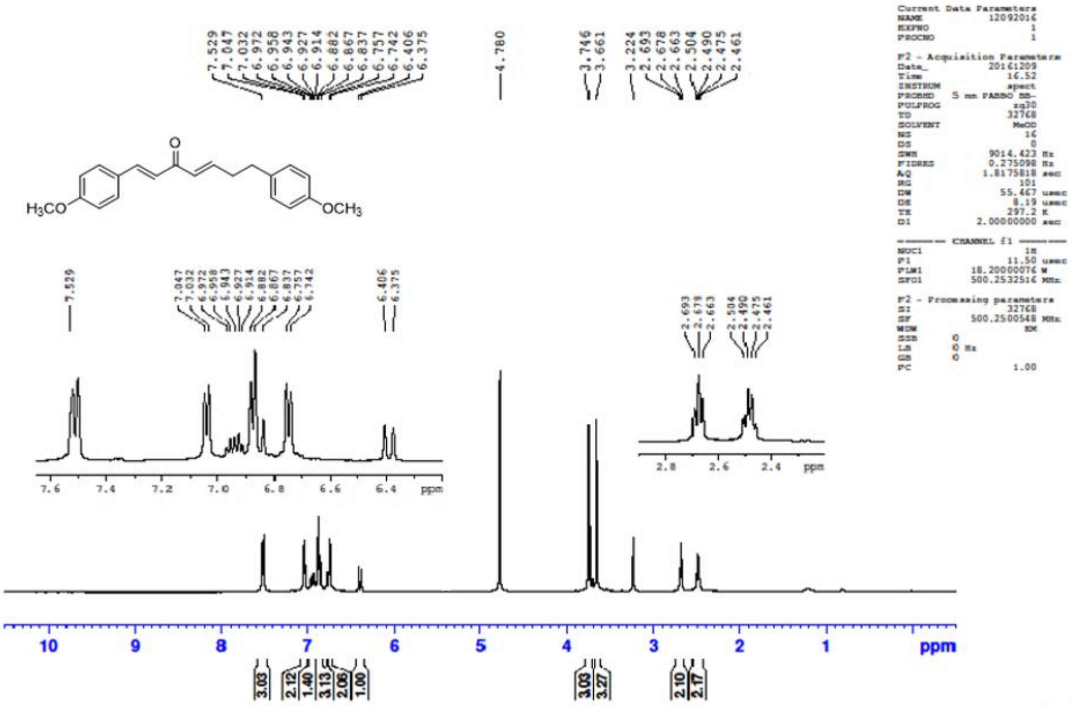
7.529
7.047
7.032
6.972
6.958
6.943
6.927
6.914
6.882
6.867
6.837
6.757
6.742
6.406
6.375
4.780
3.746
3.661
3.224
2.693
2.678
2.663
2.504
2.490
2.475
2.461
H3CO
OCH3
O
ppm
3.03
2.12
1.40
3.13
2.06
1.00
3.03
3.27
2.10
2.17

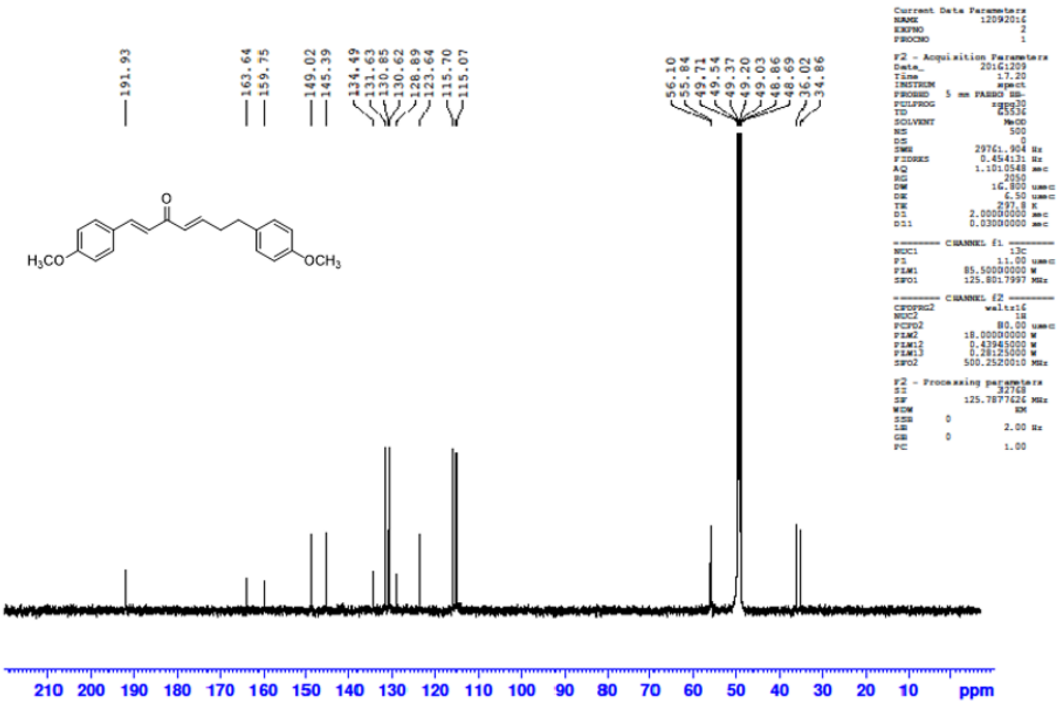
191.93
163.64
159.75
149.02
145.39
134.49
131.63
130.85
130.62
128.89
123.64
115.70
115.07
56.10
55.84
49.71
49.54
49.37
49.20
49.03
48.86
48.69
36.02
34.86
H3CO
OCH3
O
Current Data Parameters
ppm

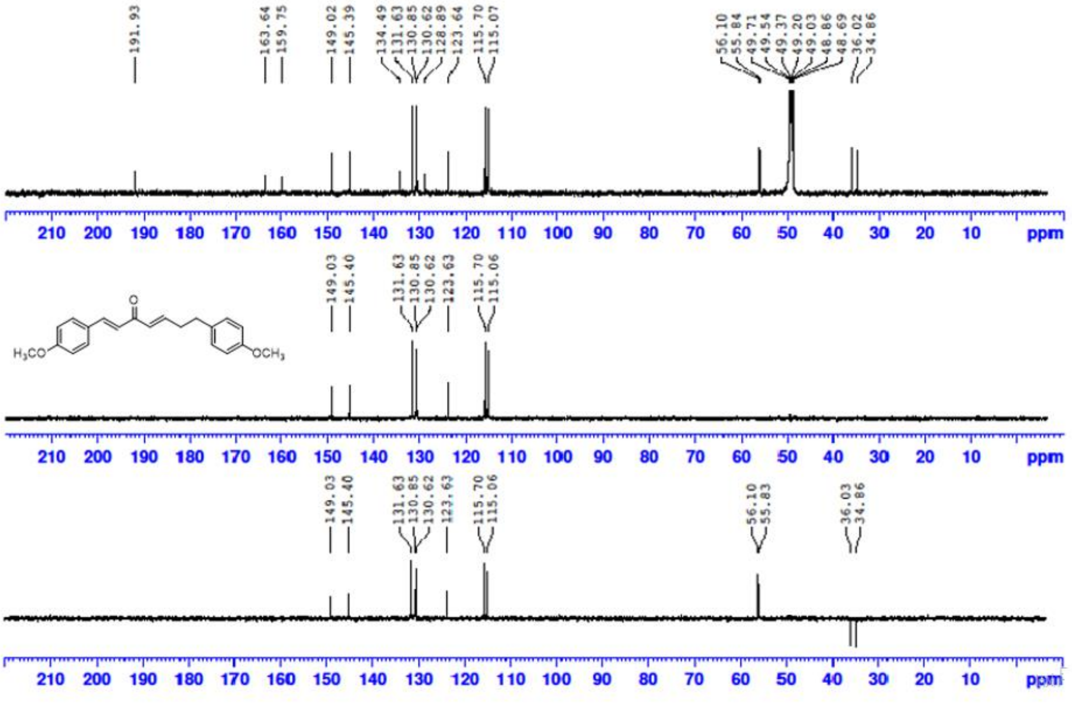
191.93
163.64
159.75
149.02
145.39
134.49
131.63
130.85
130.62
128.89
123.64
115.70
115.07
56.10
55.84
49.71
49.54
49.37
49.20
49.03
48.86
48.69
36.02
34.86
ppm
149.03
145.40
131.63
130.85
130.62
123.63
115.70
115.06
H3CO
OCH3
O
ppm
149.03
145.40
131.63
130.85
130.62
123.63
115.70
115.06
56.10
55.83
36.03
34.86
ppm

MD3

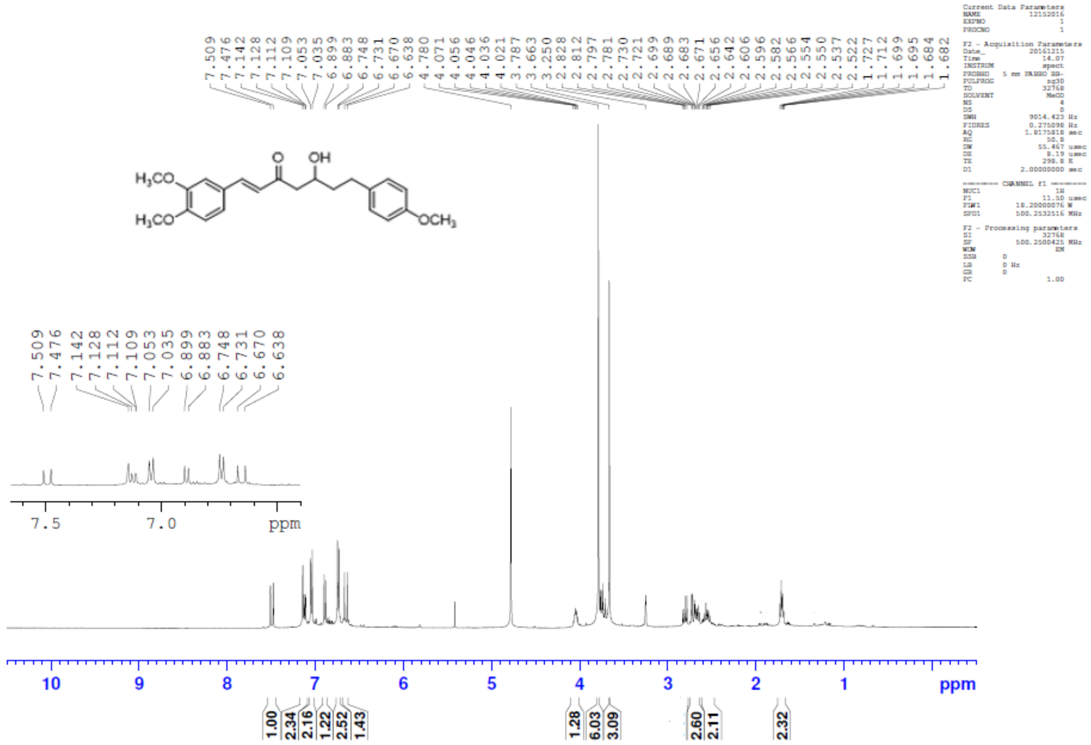
7.509
7.476
7.142
7.128
7.112
7.109
7.053
7.035
6.899
6.883
6.748
6.731
6.670
6.638
4.780
4.071
4.056
4.046
4.036
4.021
3.787
3.663
3.250
2.828
2.812
2.797
2.781
2.730
2.721
2.699
2.689
2.683
2.671
2.656
2.642
2.606
2.596
2.582
2.566
2.554
2.550
2.537
2.522
1.727
1.712
1.699
1.695
1.684
1.682
H3CO
H3CO
O
OH
OCH3
7.5
7.0
ppm
10
9
8
7
6
5
4
3
2
1
ppm
1.00
2.34
2.16
1.22
2.52
1.43
1.28
6.03
3.09
2.60
2.11
2.32

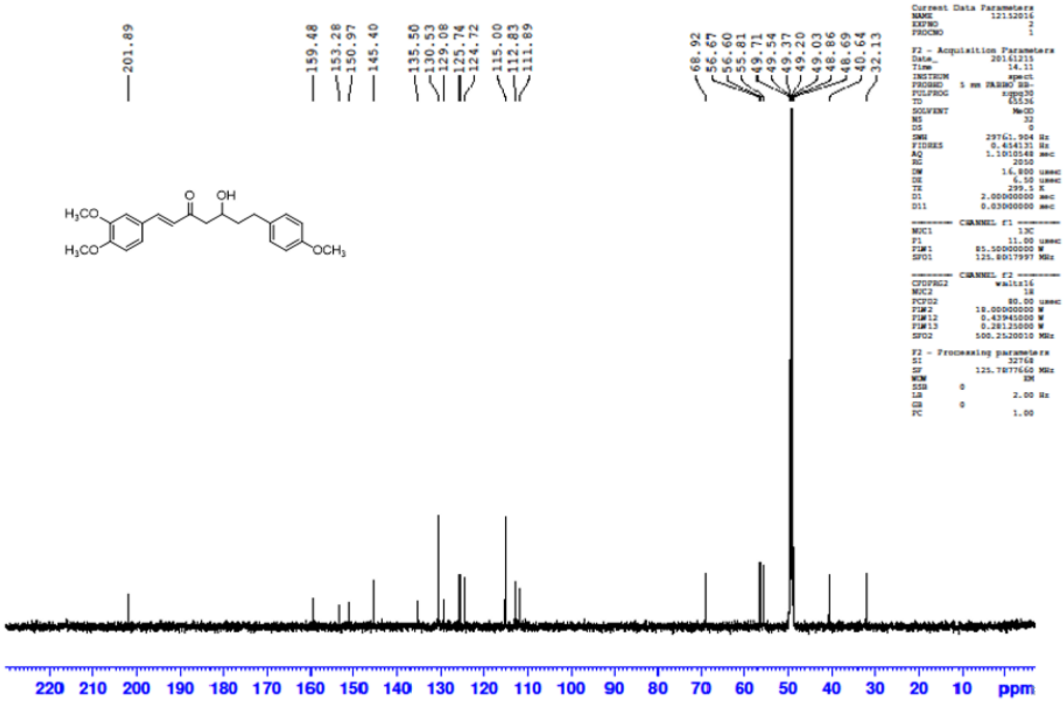
201.89
159.48
153.28
150.97
145.40
135.50
130.53
129.08
125.74
124.72
115.00
112.83
111.89
68.92
56.67
56.60
55.81
49.71
49.54
49.37
49.20
49.03
48.86
48.69
40.64
32.13
H3CO
H3CO
O
OH
OCH3
220
210
200
190
180
170
160
150
140
130
120
110
100
90
80
70
60
50
40
30
20
10
ppm

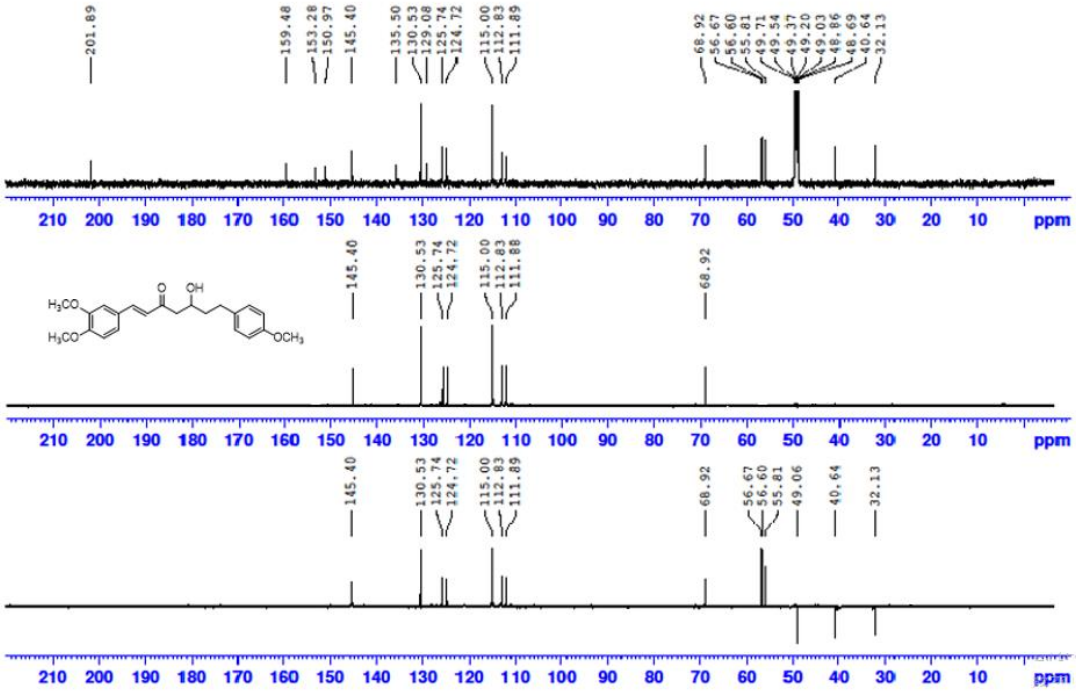

MD3a

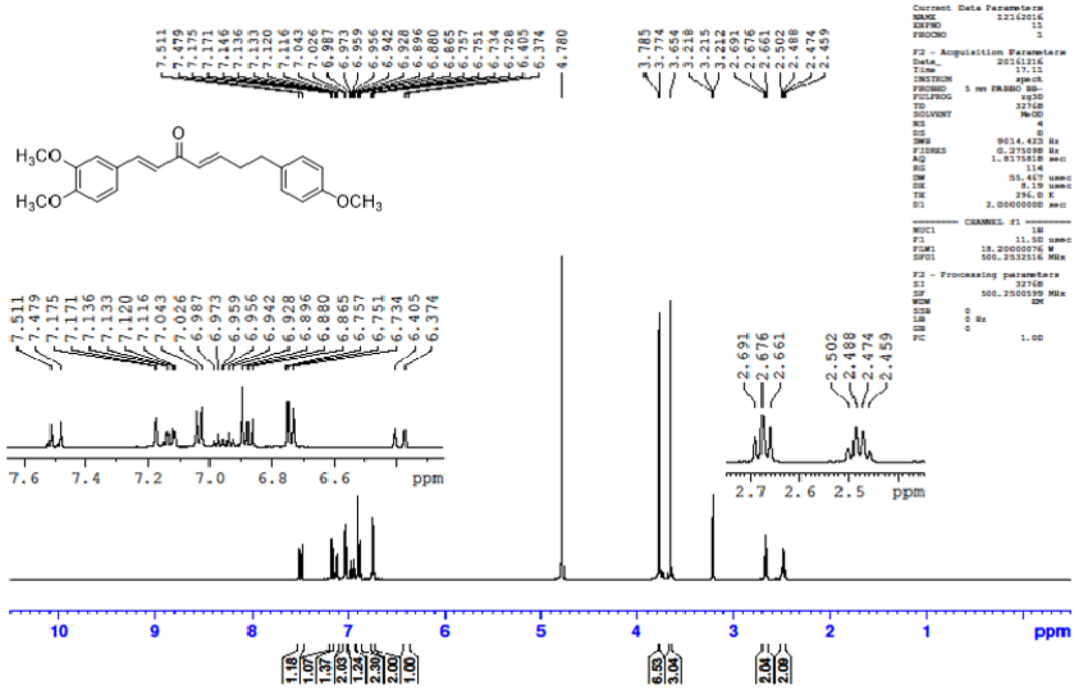

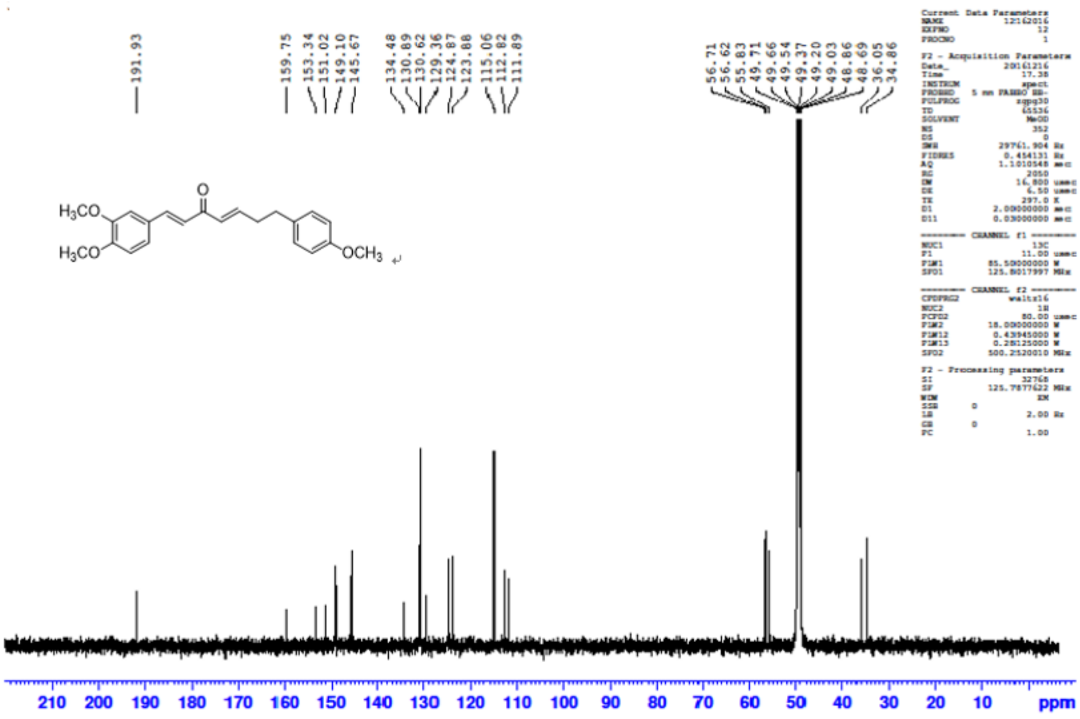

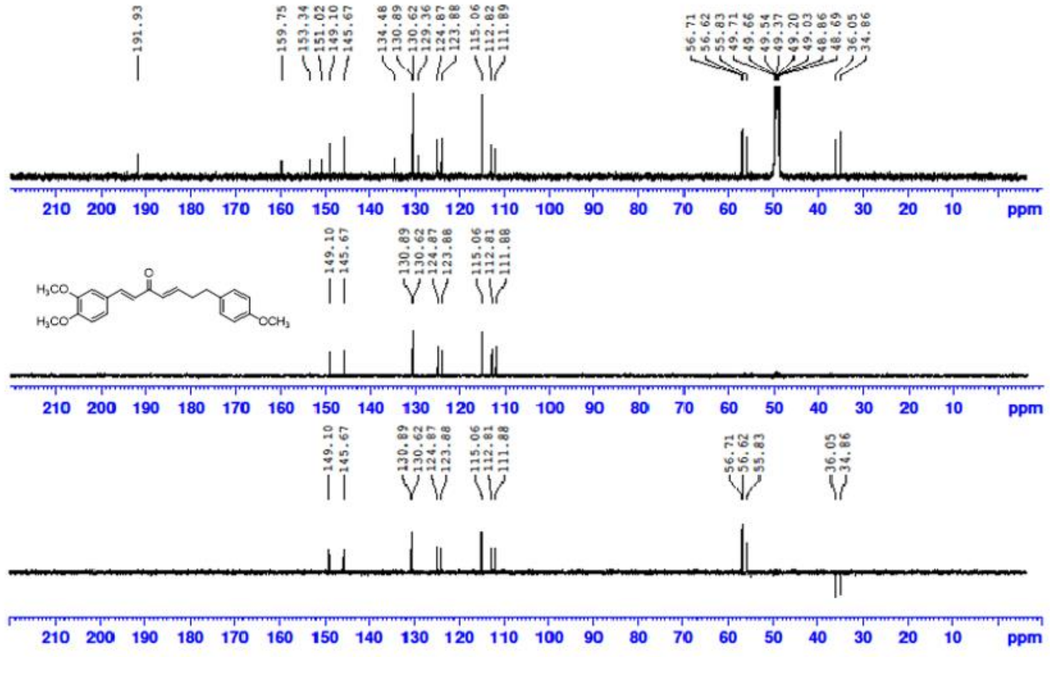

MD4

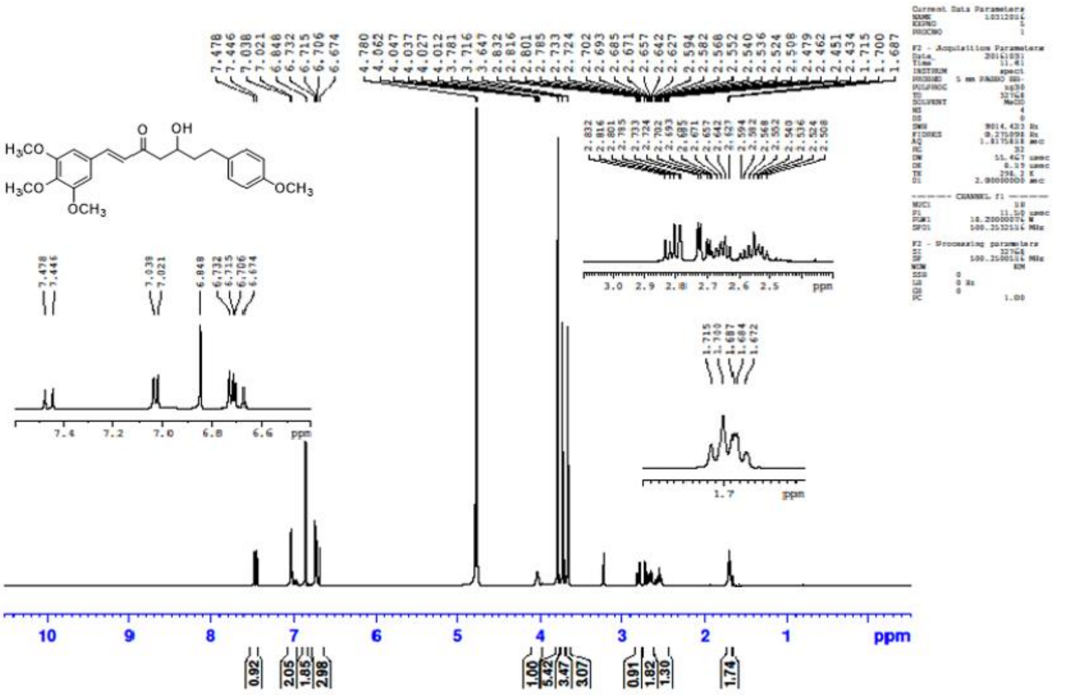

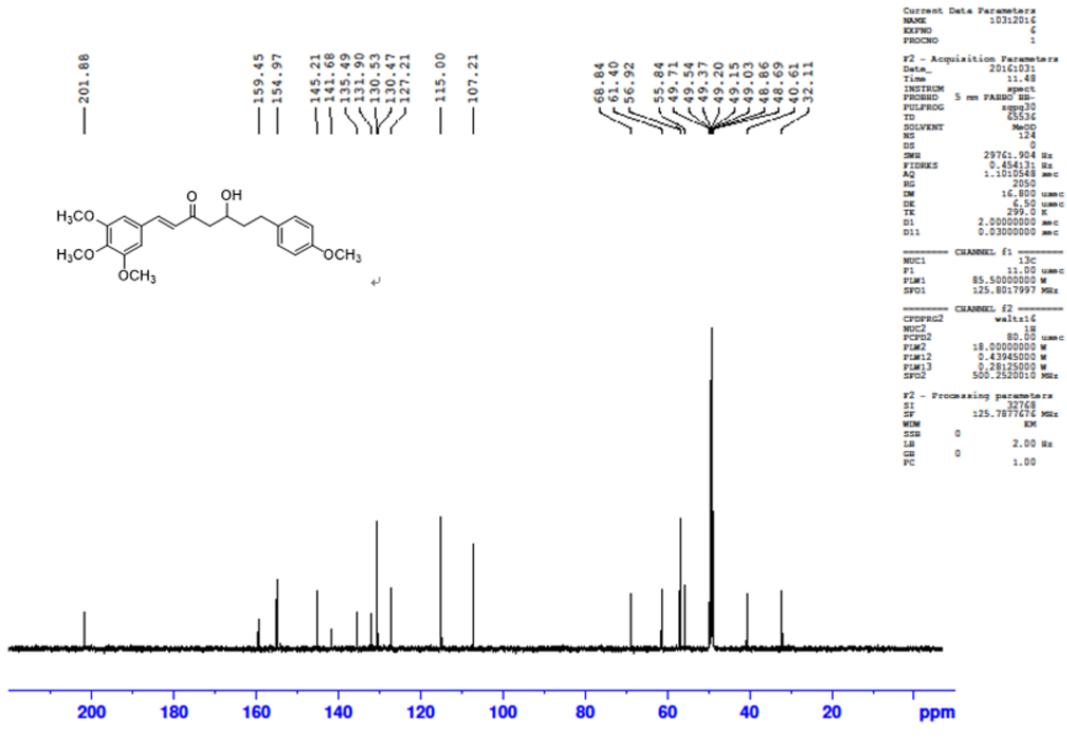

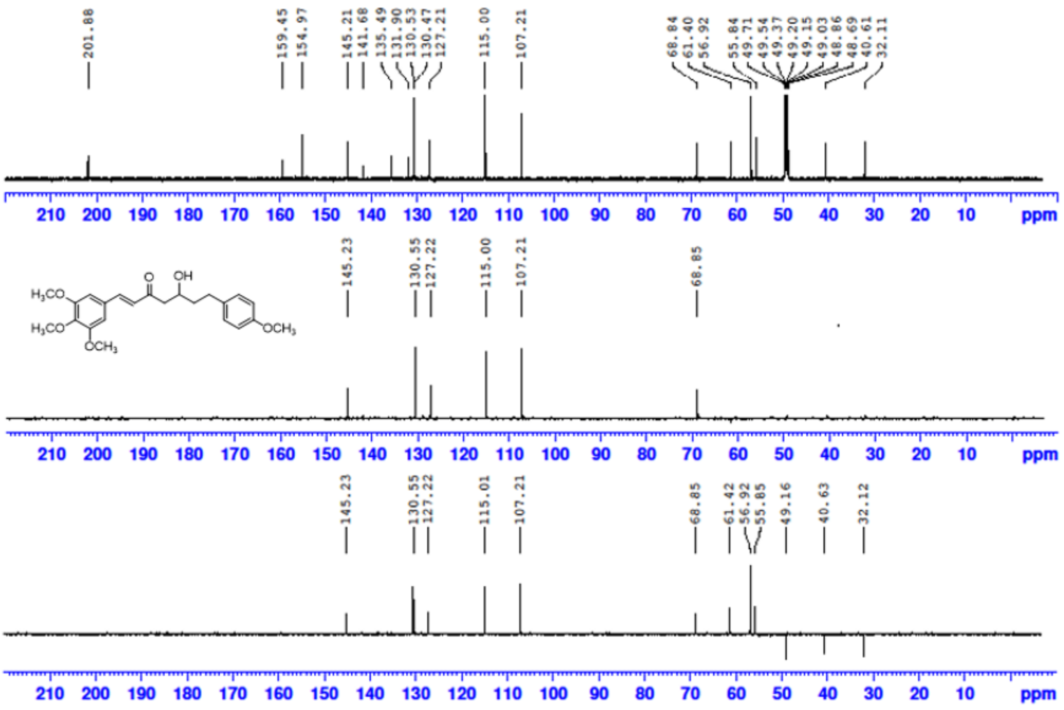

MD4a

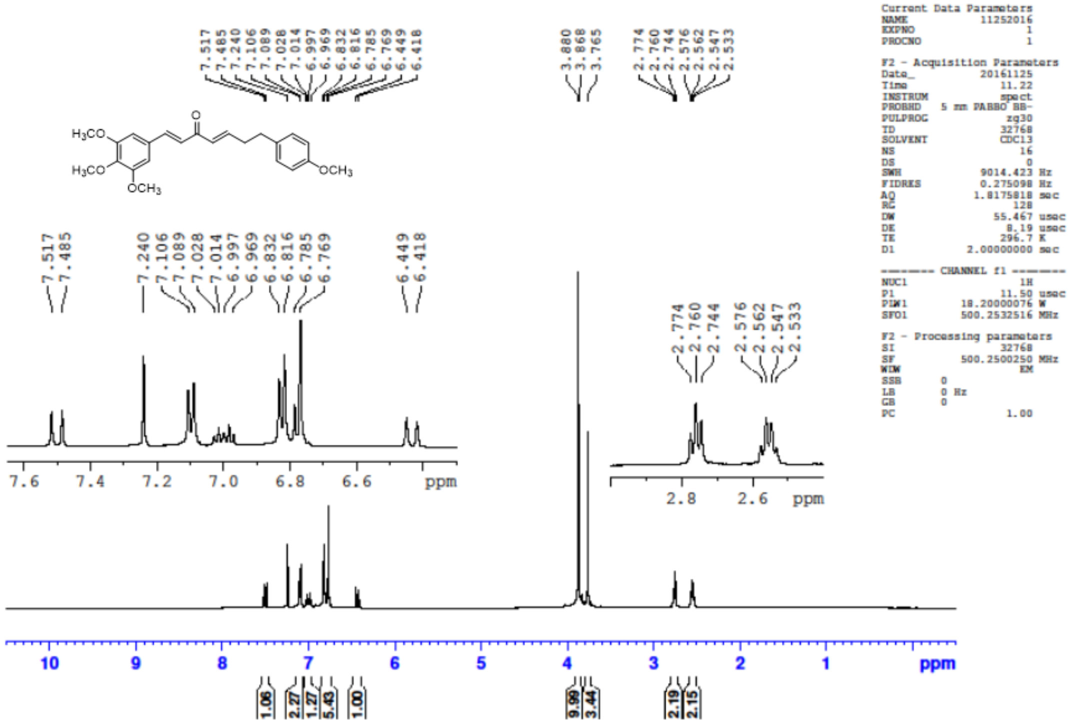

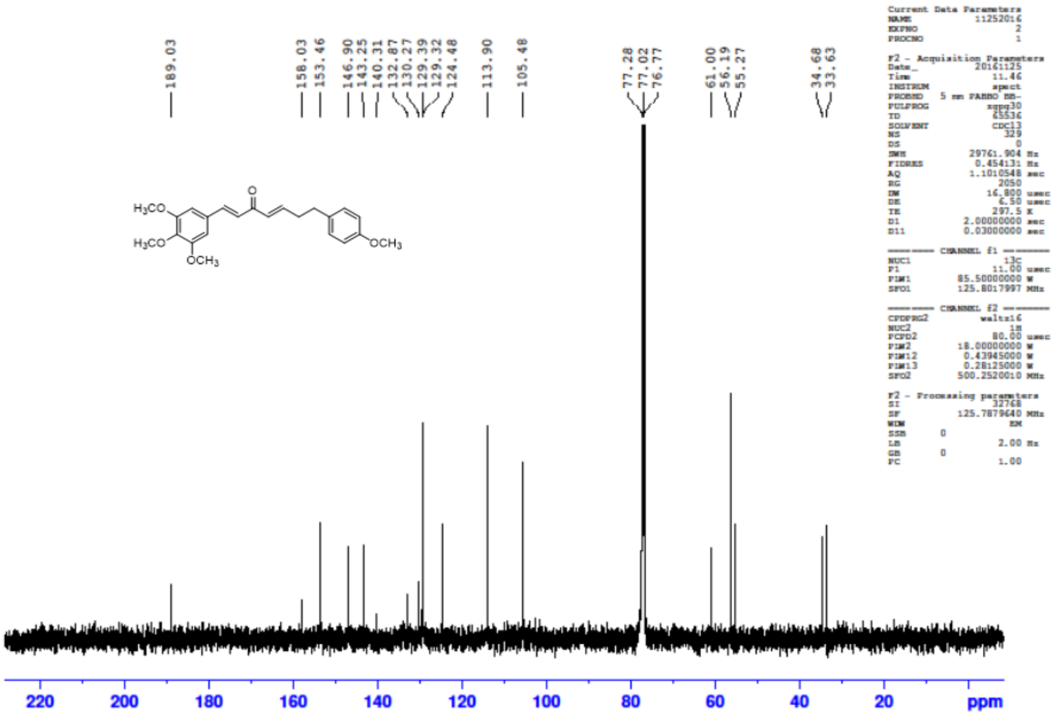

189.03
158.03
153.46
146.90
143.25
140.31
132.87
130.27
129.39
129.32
124.48
113.90
105.48
77.28
77.02
76.77
61.00
56.19
55.27
34.68
33.63
H3CO
H3CO
OCH3
O
OCH3
ppm

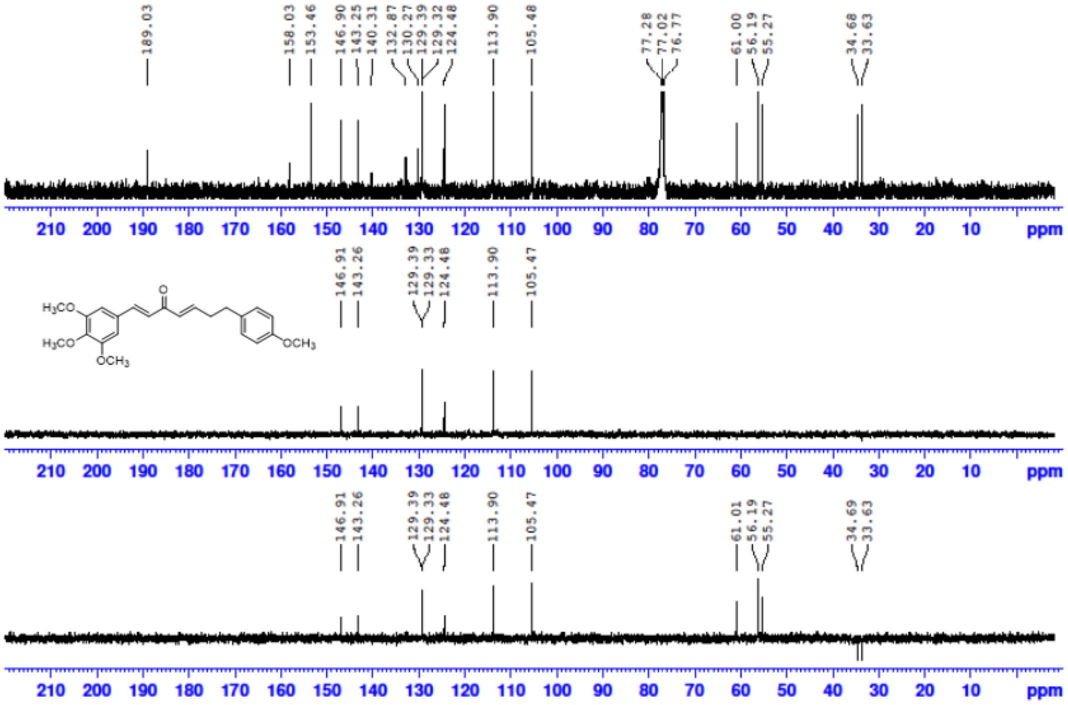

189.03
158.03
153.46
146.90
143.25
140.31
132.87
130.27
129.39
129.32
124.48
113.90
105.48
77.28
77.02
76.77
61.00
56.19
55.27
34.68
33.63
ppm
146.91
143.26
129.39
129.33
124.48
113.90
105.47
H3CO
H3CO
OCH3
O
OCH3
ppm
146.91
143.26
129.39
129.33
124.48
113.90
105.47
61.01
56.19
55.27
34.69
33.63
ppm

MD5

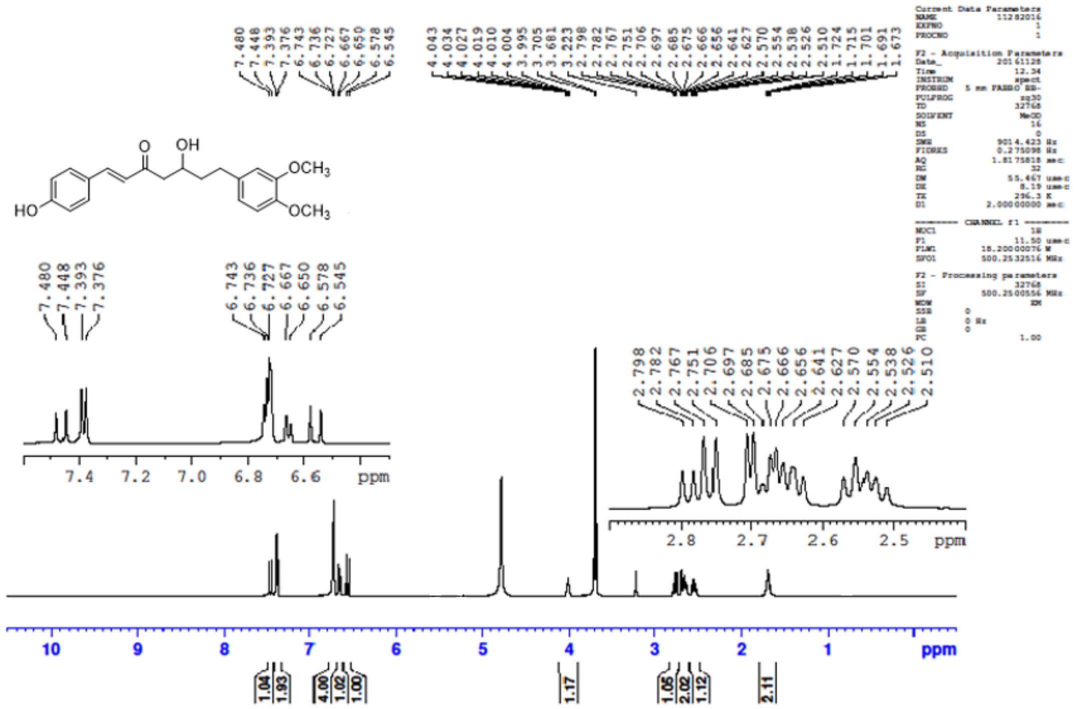

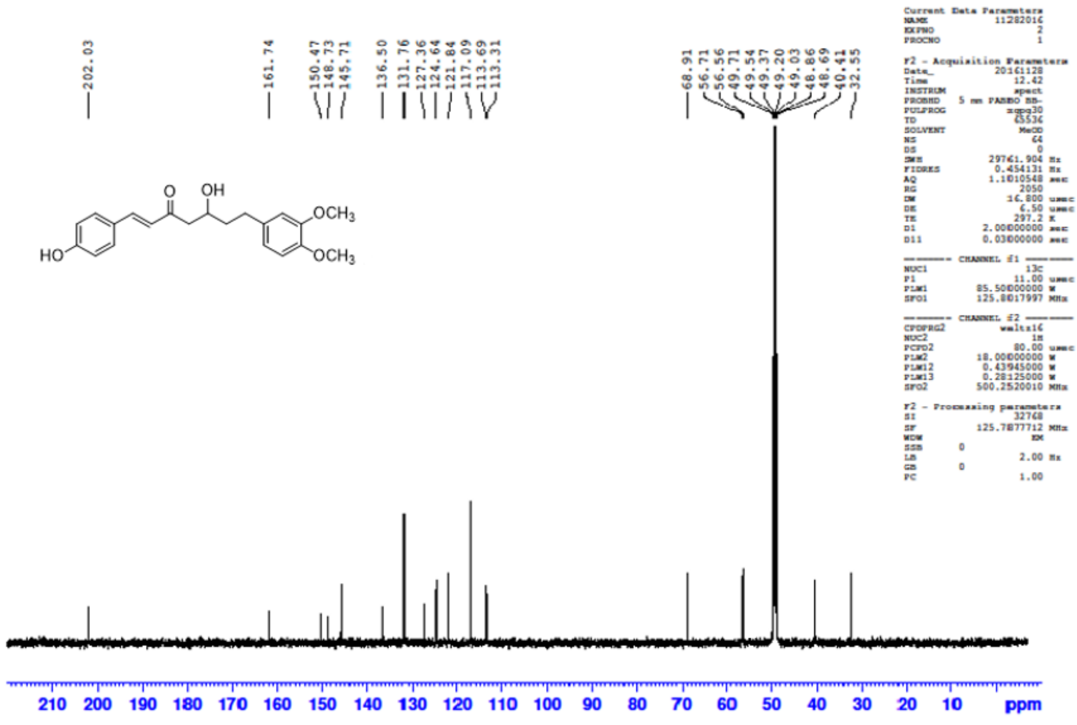

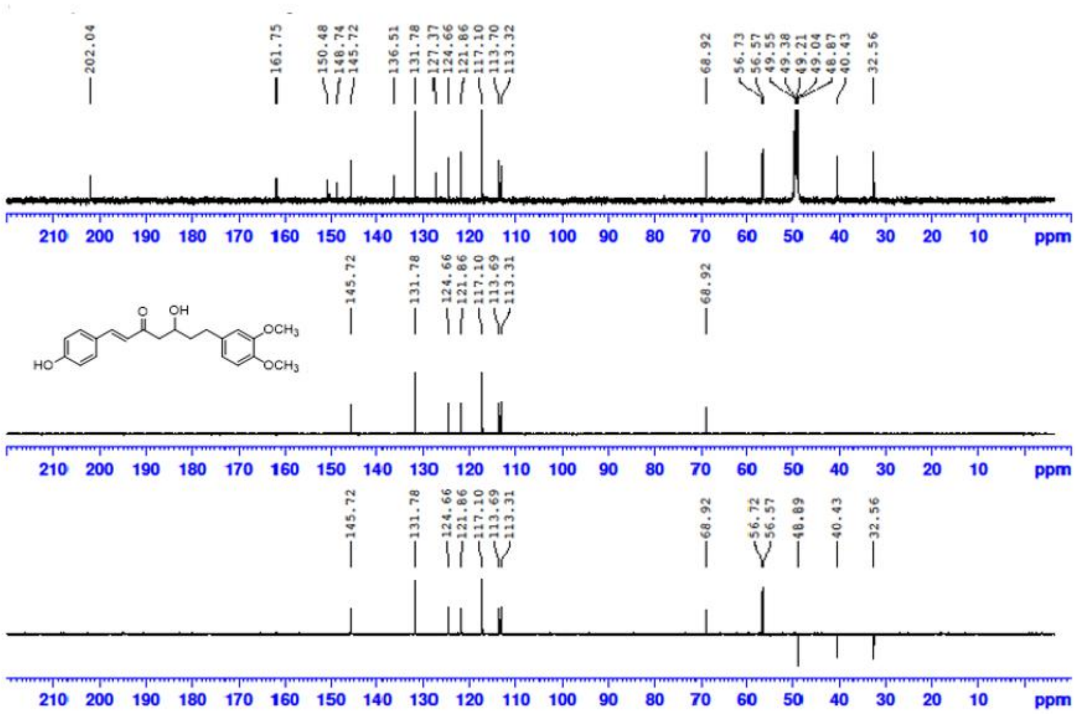
202.04
161.75
150.48
148.74
145.72
136.51
131.78
127.37
124.66
121.86
117.10
113.70
113.32
68.92
56.73
56.57
49.55
49.38
49.21
49.04
48.87
40.43
32.56
OH
OCH3
HO
OCH3
56.72
48.89
ppm

MD5a

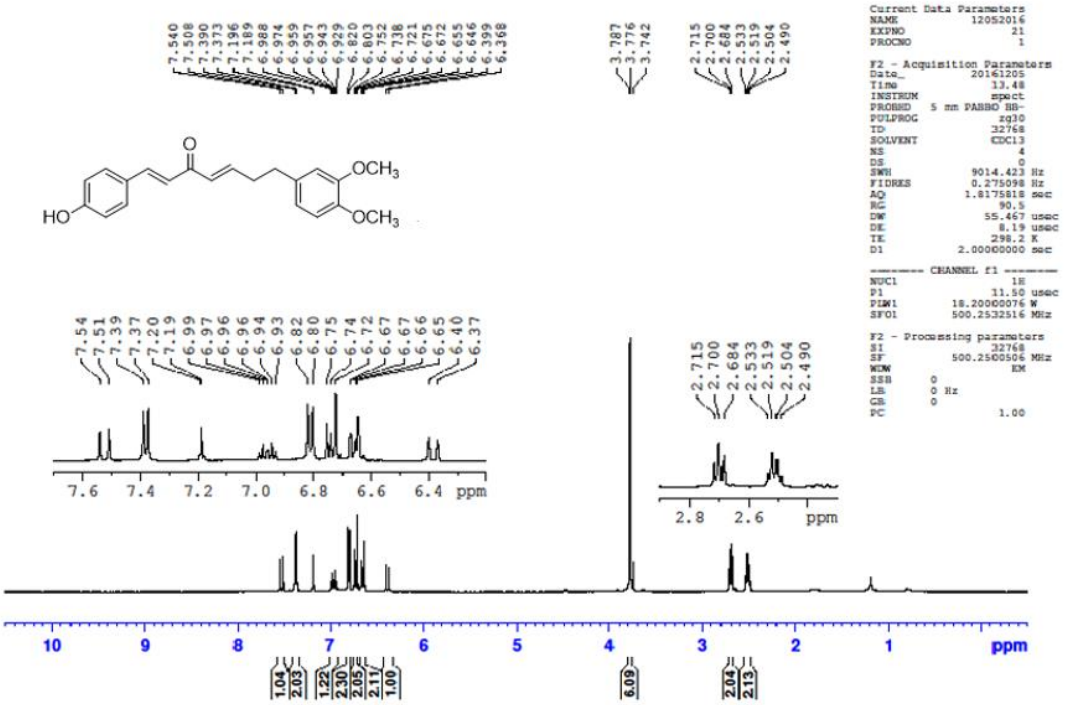
Current Data Parameters
NAME 12052016
EXPNO 21
PROCNO 1
F2 - Acquisition Parameters
Date_ 20161205
Time 13.48
SOLVENT CDCl3
NS 4
SFO1 500.2532516 MHz
HO
OCH3
OCH3
ppm

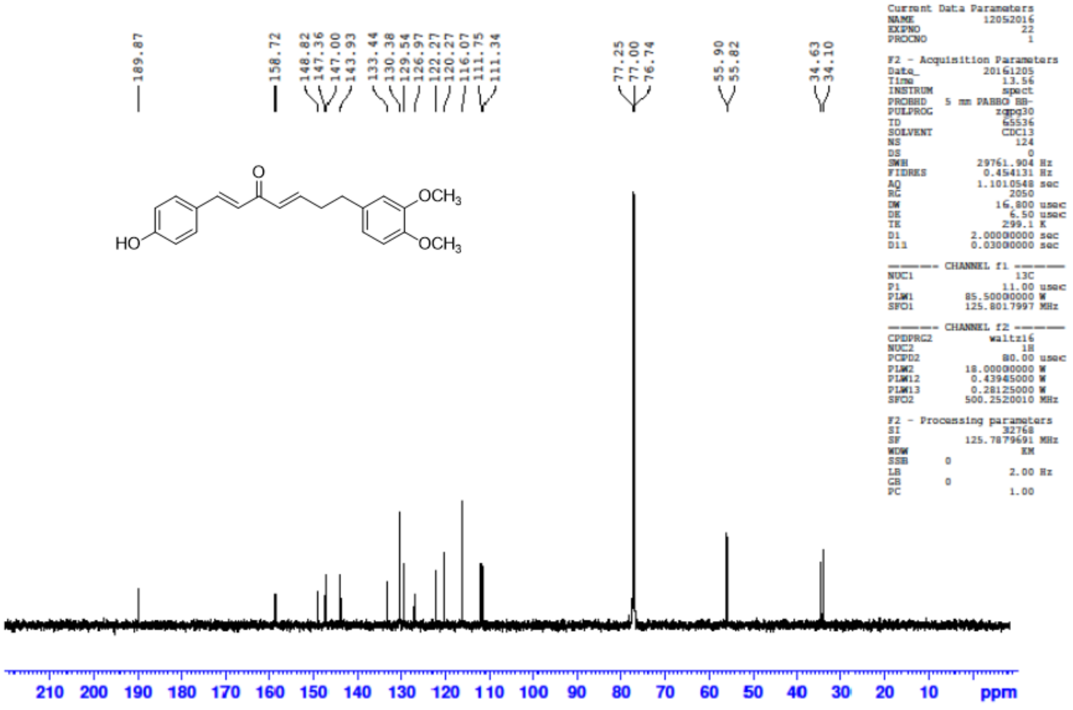
189.87
158.72
148.82
147.36
147.00
143.93
133.44
130.38
129.54
126.97
122.27
120.27
116.07
111.75
111.34
77.25
77.00
76.74
55.90
55.82
34.63
34.10
O
OCH3
HO
OCH3
Current Data Parameters
NAME 12052016
EXPNO 22
PROCNO 1
F2 - Acquisition Parameters
Date_ 20161205
Time 13.56
INSTRUM spect
PROBHD 5 mm PABBO BB-
PULPROG zgpg30
TD 65536
SOLVENT CDCl3
NS 124
DS 0
SWH 29761.904 Hz
FIDRES 0.454131 Hz
AQ 1.1010548 sec
RG 2050
DW 16.800 usec
DE 6.50 usec
TE 299.1 K
D1 2.00000000 sec
D11 0.03000000 sec
CHANNEL f1
NUC1 13C
P1 11.00 usec
PLW1 85.50000000 W
SFO1 125.8017997 MHz
CHANNEL f2
CPDPRG2 waltz16
NUC2 1H
PCPD2 80.00 usec
PLW2 18.00000000 W
PLW12 0.43945000 W
PLW13 0.28125000 W
SFO2 500.2520010 MHz
F2 - Processing parameters
SI 32768
SF 125.7879691 MHz
WDW EM
SSB 0
LB 2.00 Hz
GB 0
PC 1.00
210 200 190 180 170 160 150 140 130 120 110 100 90 80 70 60 50 40 30 20 10 ppm

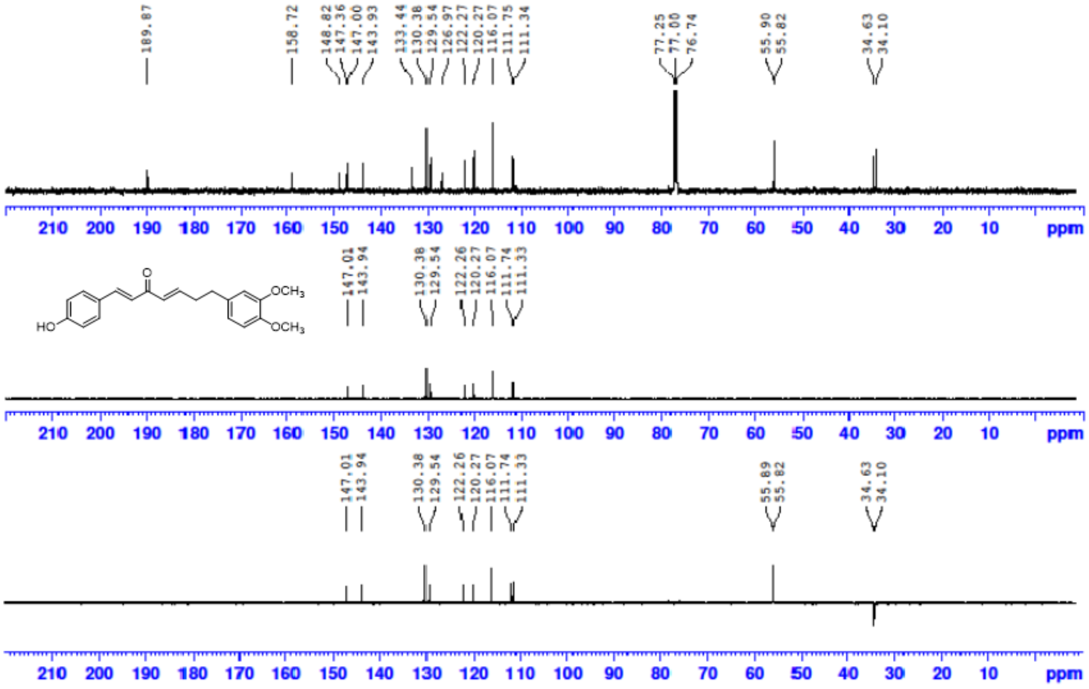
189.87
158.72
148.82
147.36
147.00
143.93
133.44
130.38
129.54
126.97
122.27
120.27
116.07
111.75
111.34
77.25
77.00
76.74
55.90
55.82
34.63
34.10
210 200 190 180 170 160 150 140 130 120 110 100 90 80 70 60 50 40 30 20 10 ppm
147.01
143.94
130.38
129.54
122.26
120.27
116.07
111.74
111.33
O
OCH3
HO
OCH3
210 200 190 180 170 160 150 140 130 120 110 100 90 80 70 60 50 40 30 20 10 ppm
147.01
143.94
130.38
129.54
122.26
120.27
116.07
111.74
111.33
55.89
55.82
34.63
34.10
210 200 190 180 170 160 150 140 130 120 110 100 90 80 70 60 50 40 30 20 10 ppm

MD6

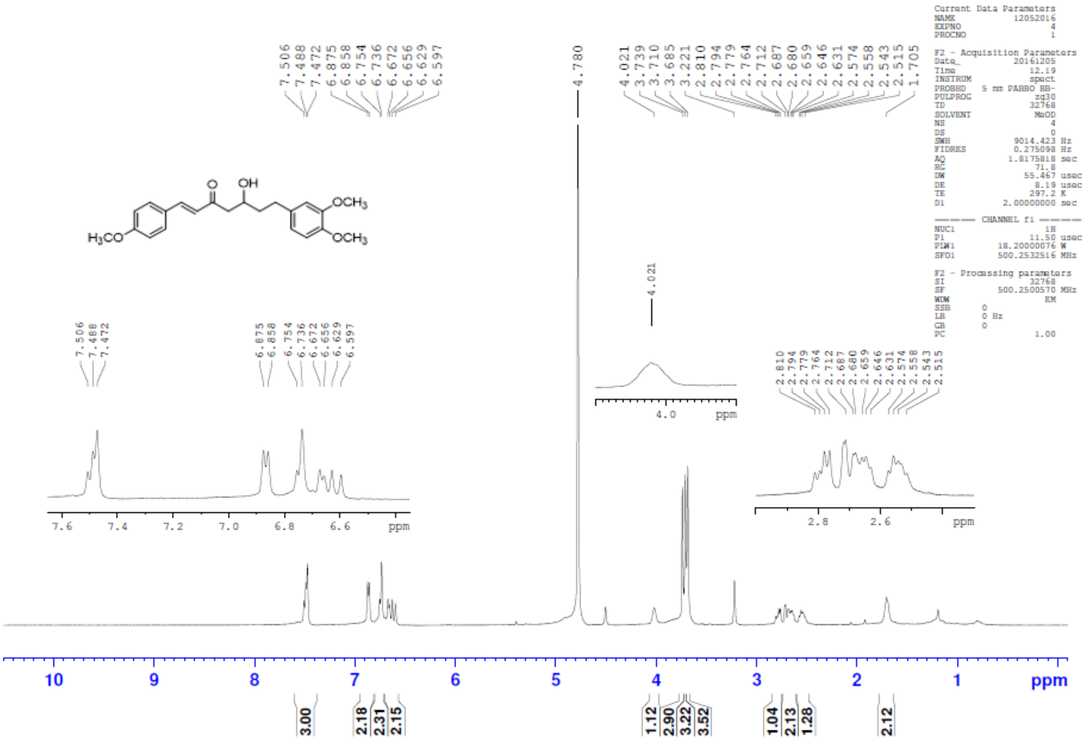

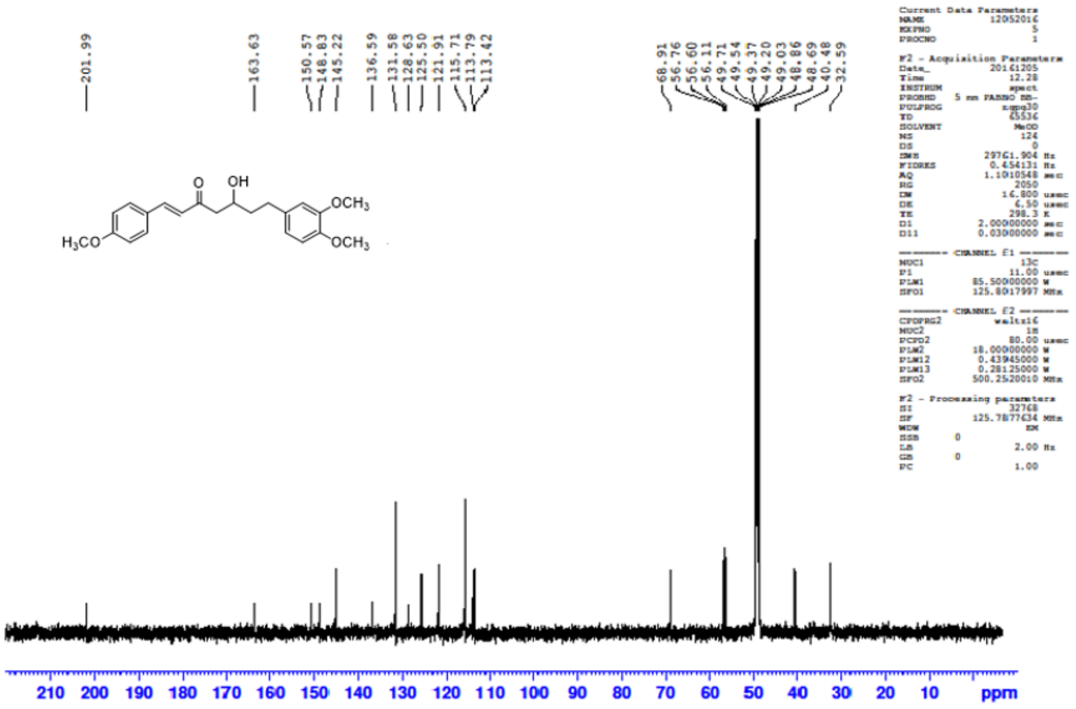

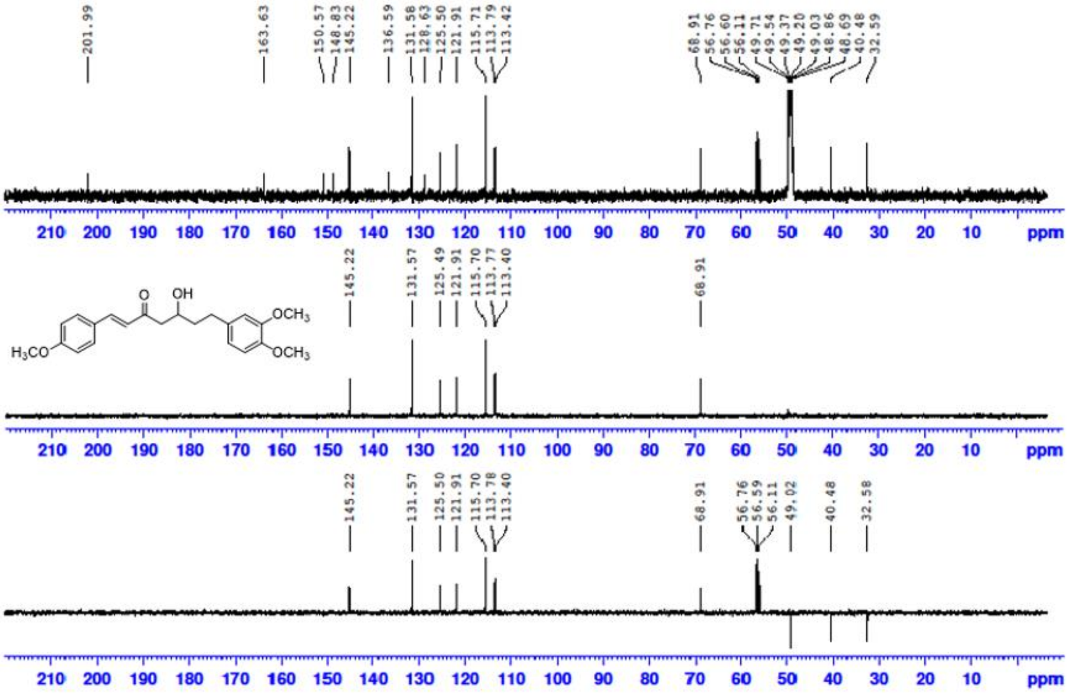
201.99
163.63
150.57 148.83 145.22
136.59
131.58 128.63 125.50 121.91 115.71 113.79 113.42
68.91 56.76 56.60 56.11 49.71 49.54 49.37 49.20 49.03 48.86 48.69 40.48 32.59
210 200 190 180 170 160 150 140 130 120 110 100 90 80 70 60 50 40 30 20 10 ppm
145.22
131.57 125.49 121.91 115.70 113.77 113.40
68.91
OH
O
OCH3
H3CO
OCH3
210 200 190 180 170 160 150 140 130 120 110 100 90 80 70 60 50 40 30 20 10 ppm
145.22
131.57 125.50 121.91 115.70 113.78 113.40
68.91
56.76 56.59 56.11
49.02
40.48
32.58
210 200 190 180 170 160 150 140 130 120 110 100 90 80 70 60 50 40 30 20 10 ppm

MD6a

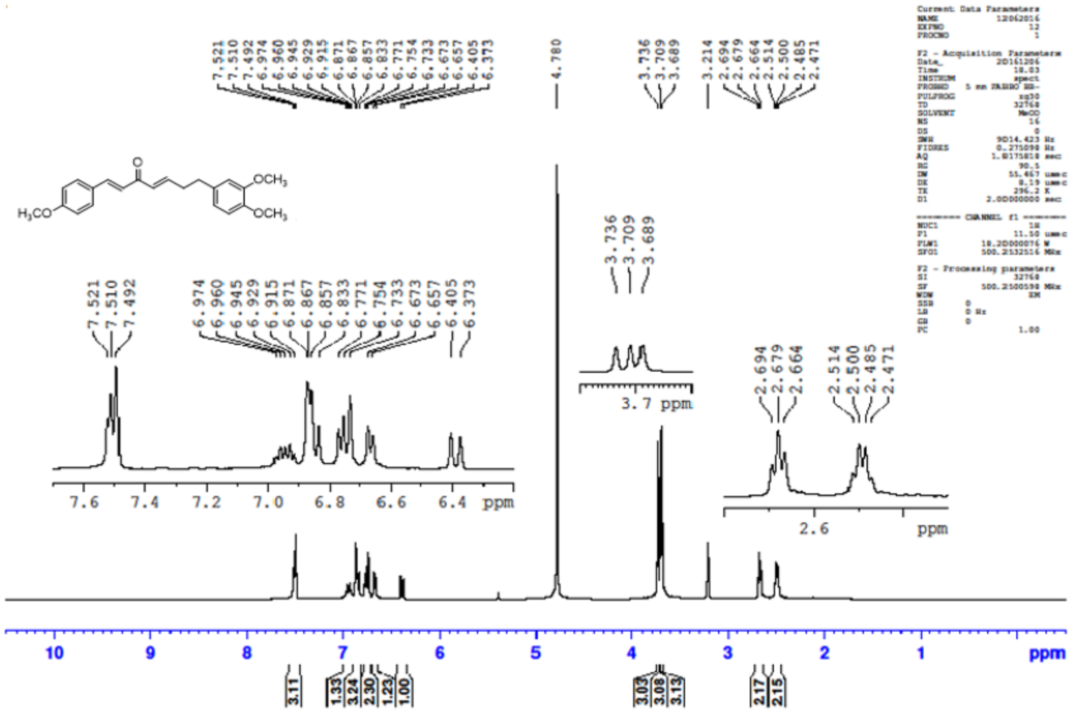
7.521 7.510 7.492 6.974 6.960 6.945 6.929 6.915 6.871 6.867 6.857 6.833 6.771 6.754 6.733 6.673 6.657 6.405 6.373
4.780
3.736 3.709 3.689
3.214
2.694 2.679 2.664 2.514 2.500 2.485 2.471
O
OCH3
H3CO
OCH3
7.6 7.4 7.2 7.0 6.8 6.6 6.4 ppm
3.7 ppm
2.6 ppm
10 9 8 7 6 5 4 3 2 1 ppm
3.11 1.33 3.24 2.30 1.23 1.00 3.03 3.08 3.13 2.17 2.15

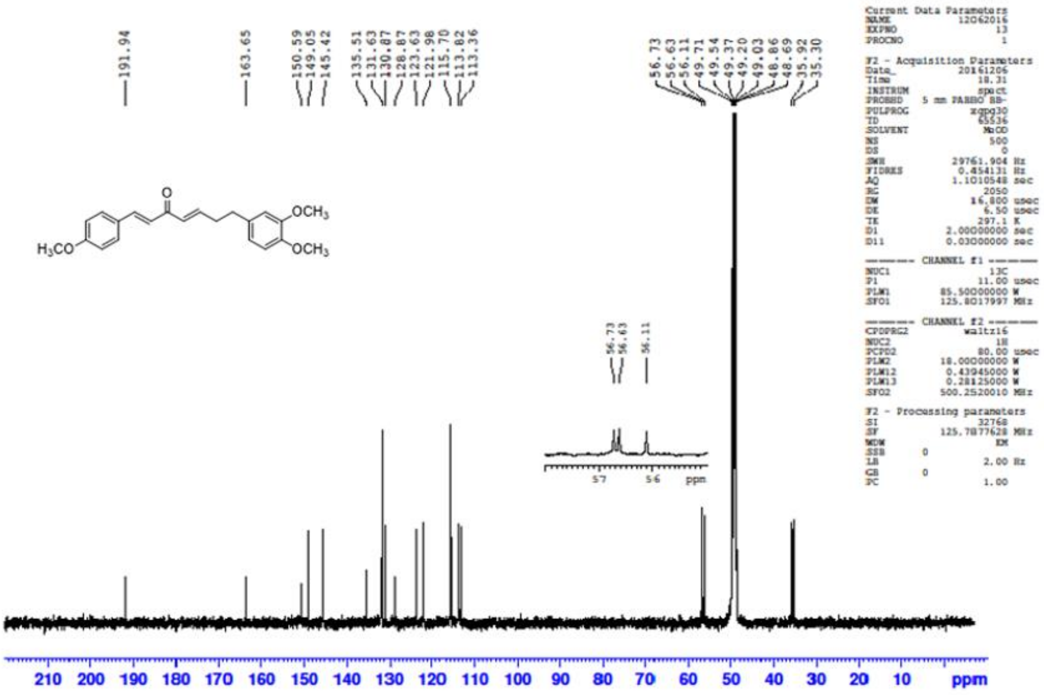
191.94
163.65
150.59
149.05
145.42
135.51
131.63
130.87
128.87
123.63
121.98
115.70
113.82
113.36
56.73
56.63
56.11
49.71
49.54
49.37
49.20
49.03
48.86
48.69
35.92
35.30
O
OCH3
H3CO
OCH3
ppm

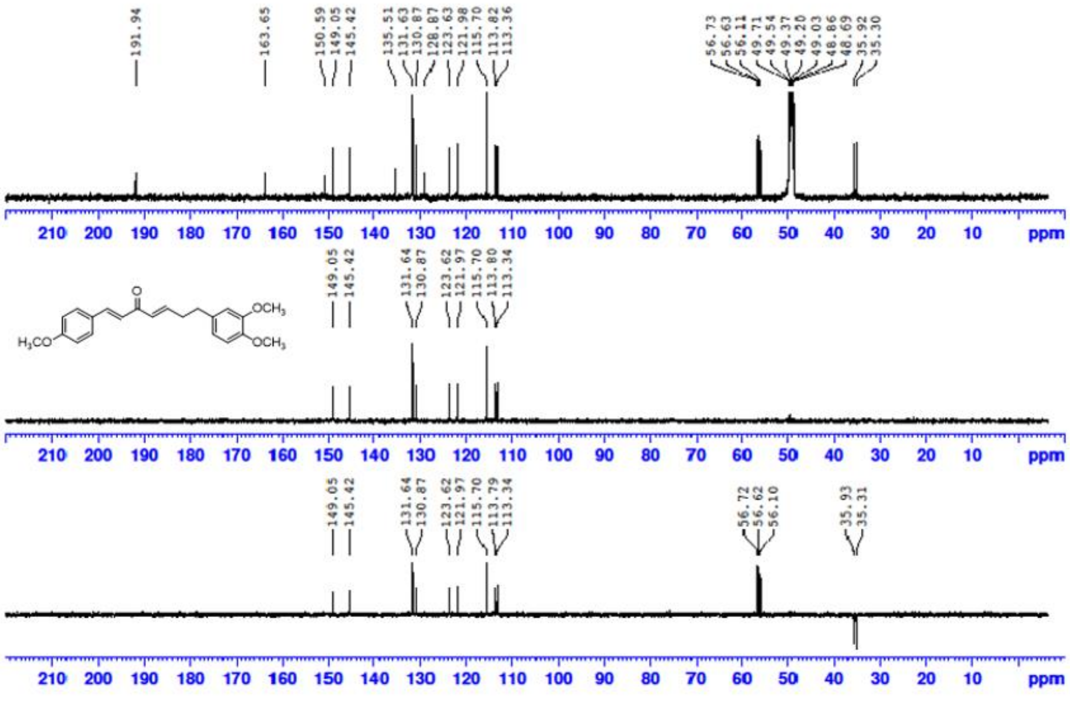
191.94
163.65
150.59
149.05
145.42
135.51
131.63
130.87
128.87
123.63
121.98
115.70
113.82
113.36
56.73
56.63
56.11
49.71
49.54
49.37
49.20
49.03
48.86
48.69
35.92
35.30
ppm
149.05
145.42
131.64
130.87
123.62
121.97
115.70
113.80
113.34
O
OCH3
H3CO
OCH3
ppm
149.05
145.42
131.64
130.87
123.62
121.97
115.70
113.79
113.34
56.72
56.62
56.10
35.93
35.31
ppm

MD7

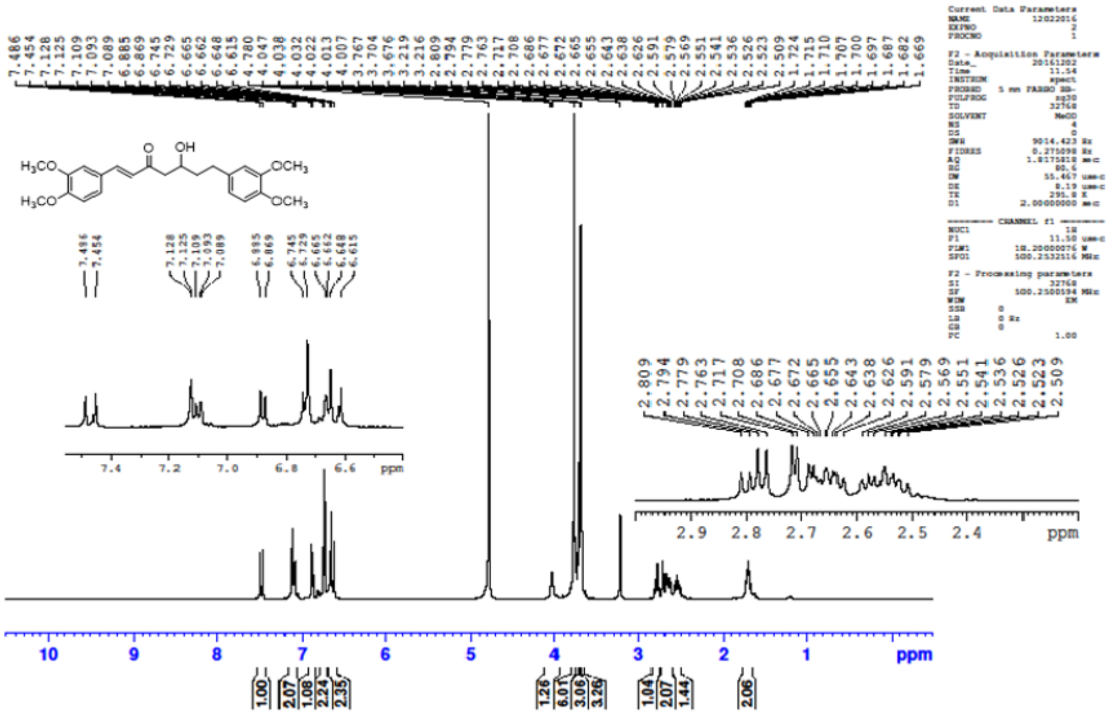

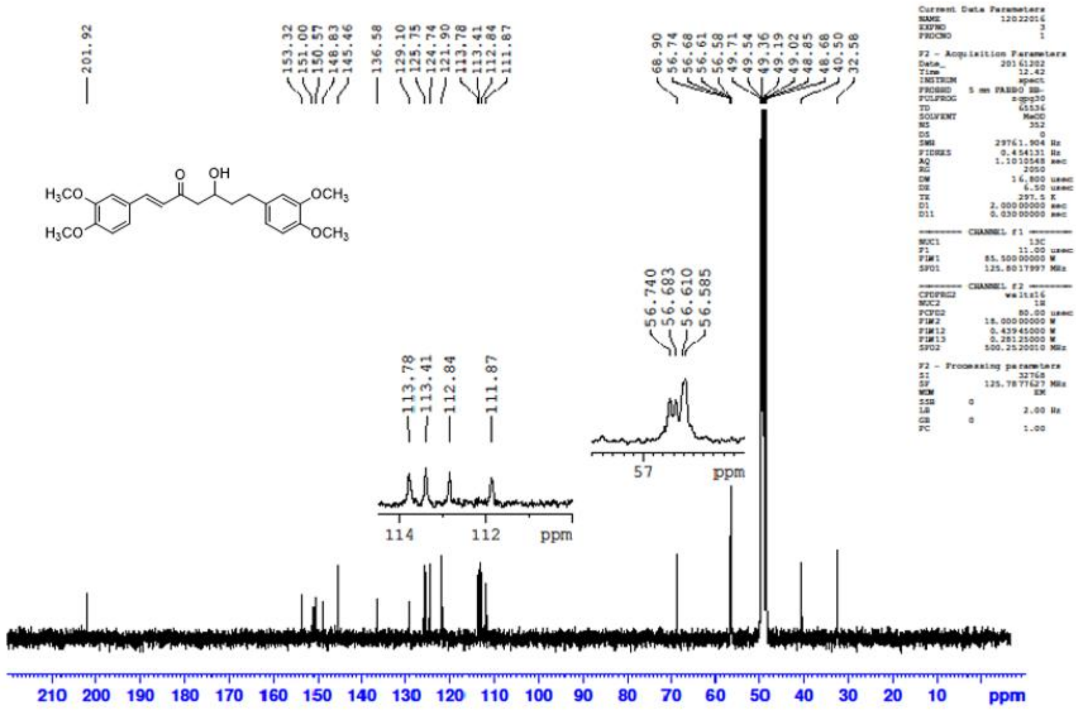

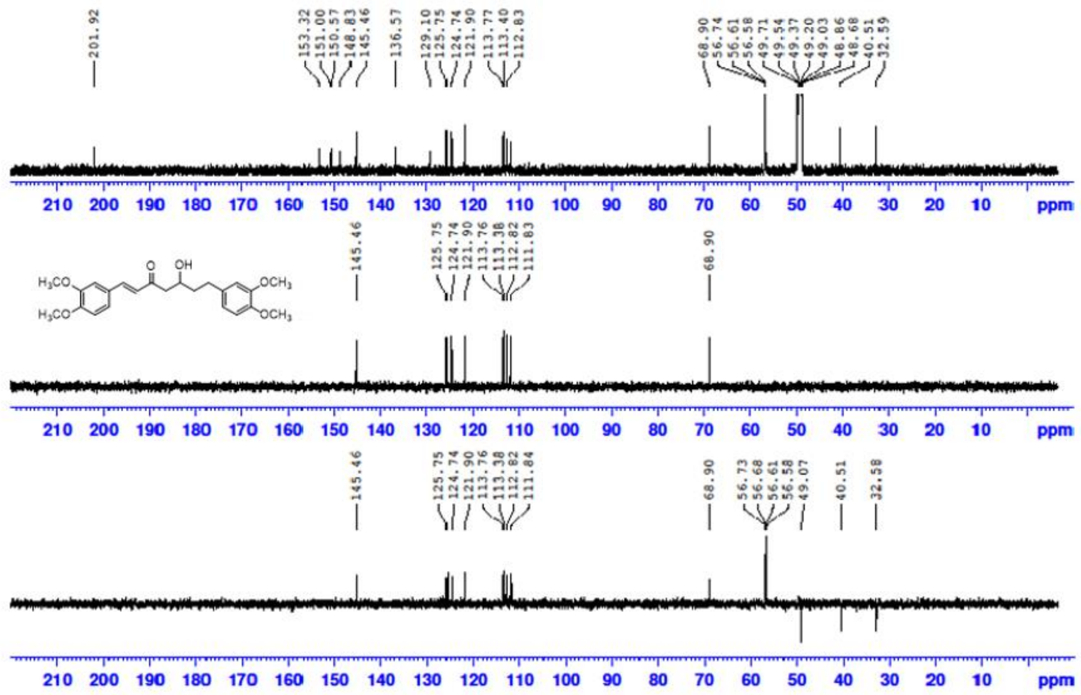

MD7a

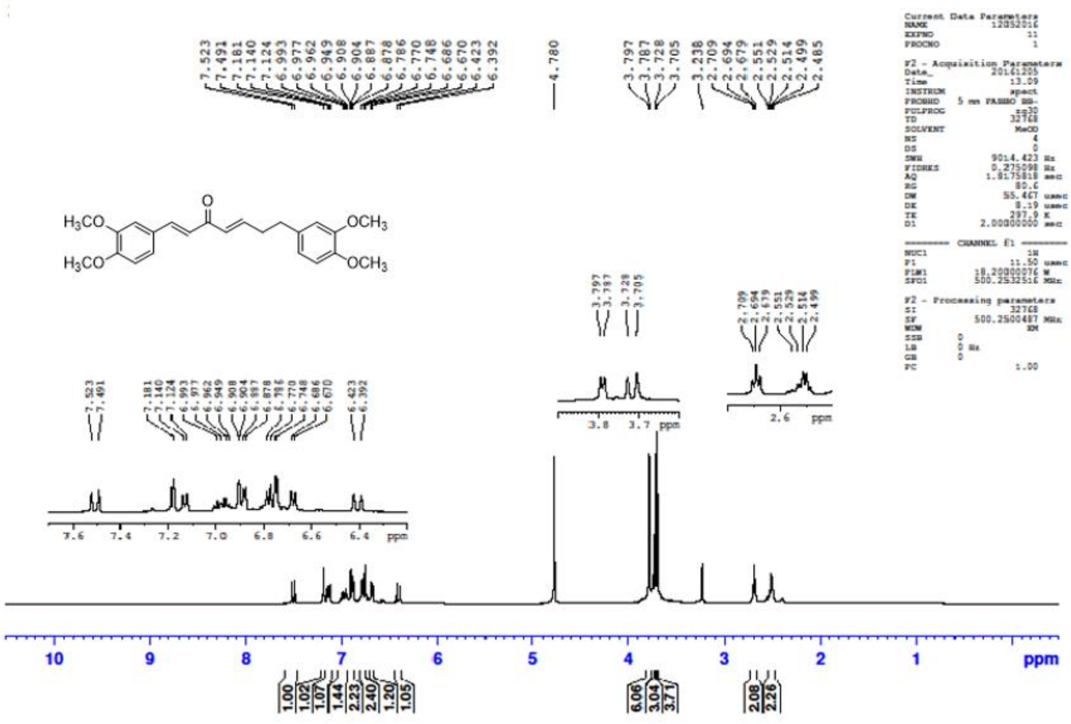

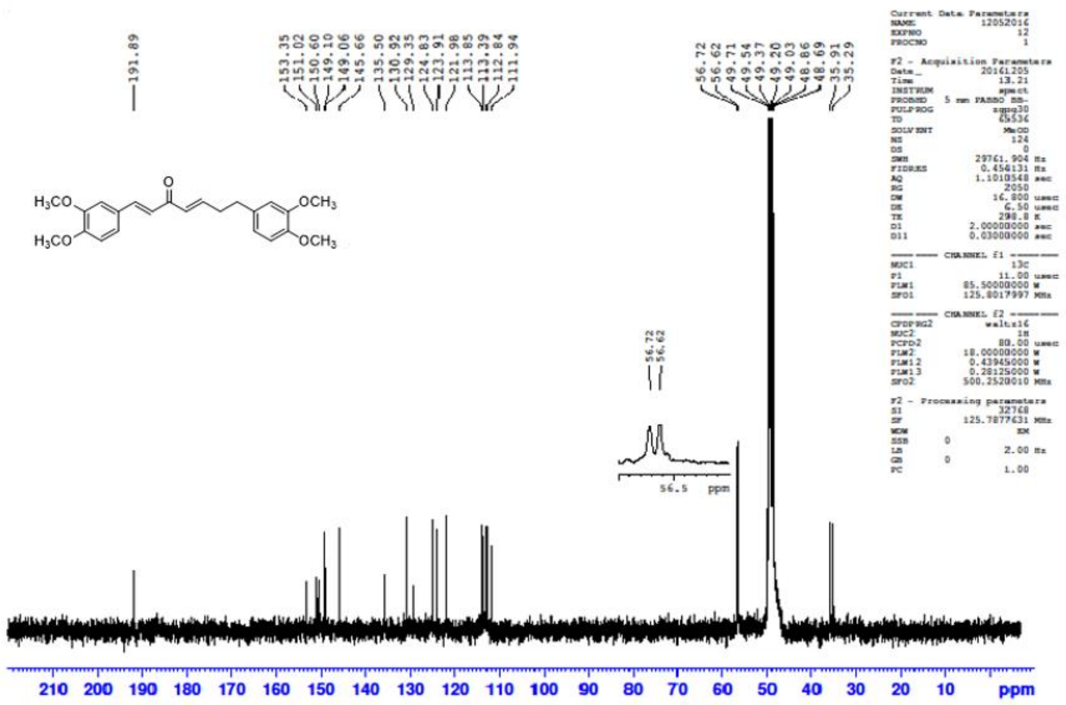
191.89
153.35 151.02 150.60 149.10 149.06 145.66 135.50 130.92 129.35 124.83 123.91 121.98 113.85 113.39 112.84 111.94
56.72 56.62 49.71 49.54 49.37 49.20 49.03 48.86 48.69 35.91 35.29
Current Data Parameters
56.72
56.62
56.5 ppm
210 200 190 180 170 160 150 140 130 120 110 100 90 80 70 60 50 40 30 20 10 ppm

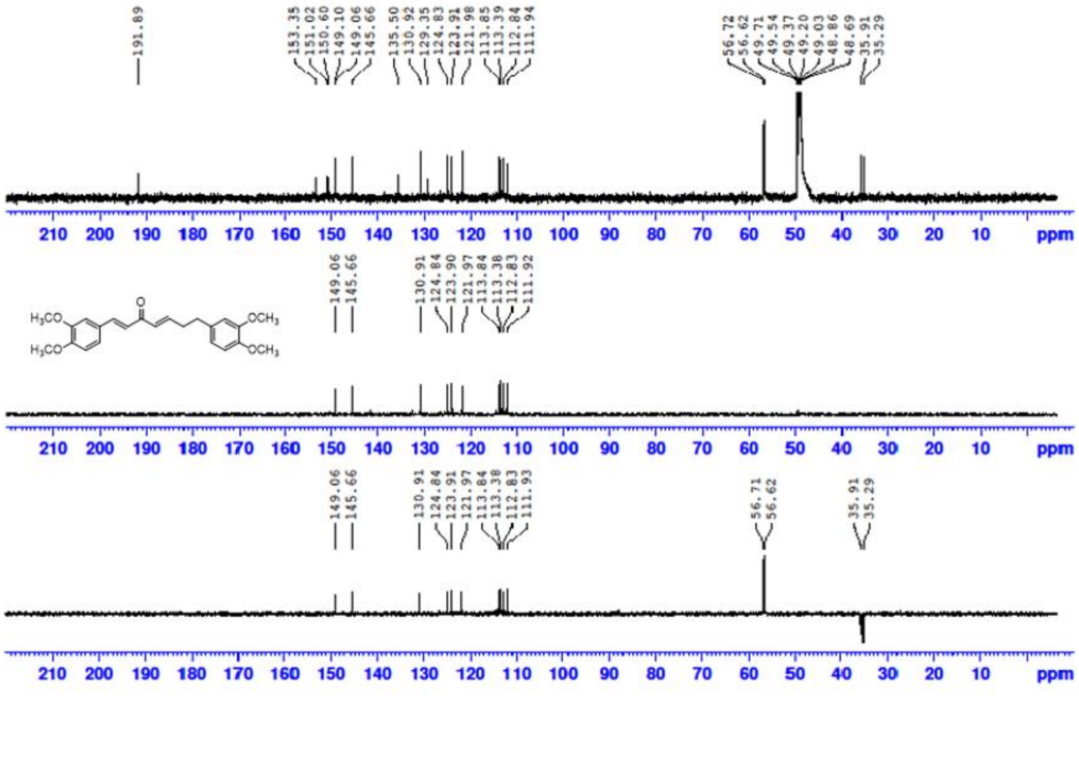
191.89
153.35 151.02 150.60 149.10 149.06 145.66 135.50 130.92 129.35 124.83 123.91 121.98 113.85 113.39 112.84 111.94
56.72 56.62 49.71 49.54 49.37 49.20 49.03 48.86 48.69 35.91 35.29
210 200 190 180 170 160 150 140 130 120 110 100 90 80 70 60 50 40 30 20 10 ppm
149.06 145.66 130.91 124.84 123.90 121.97 113.84 113.38 112.83 111.92
210 200 190 180 170 160 150 140 130 120 110 100 90 80 70 60 50 40 30 20 10 ppm
149.06 145.66 130.91 124.84 123.91 121.97 113.84 113.38 112.83 111.93 56.71 56.62 35.91 35.29
210 200 190 180 170 160 150 140 130 120 110 100 90 80 70 60 50 40 30 20 10 ppm

MD8

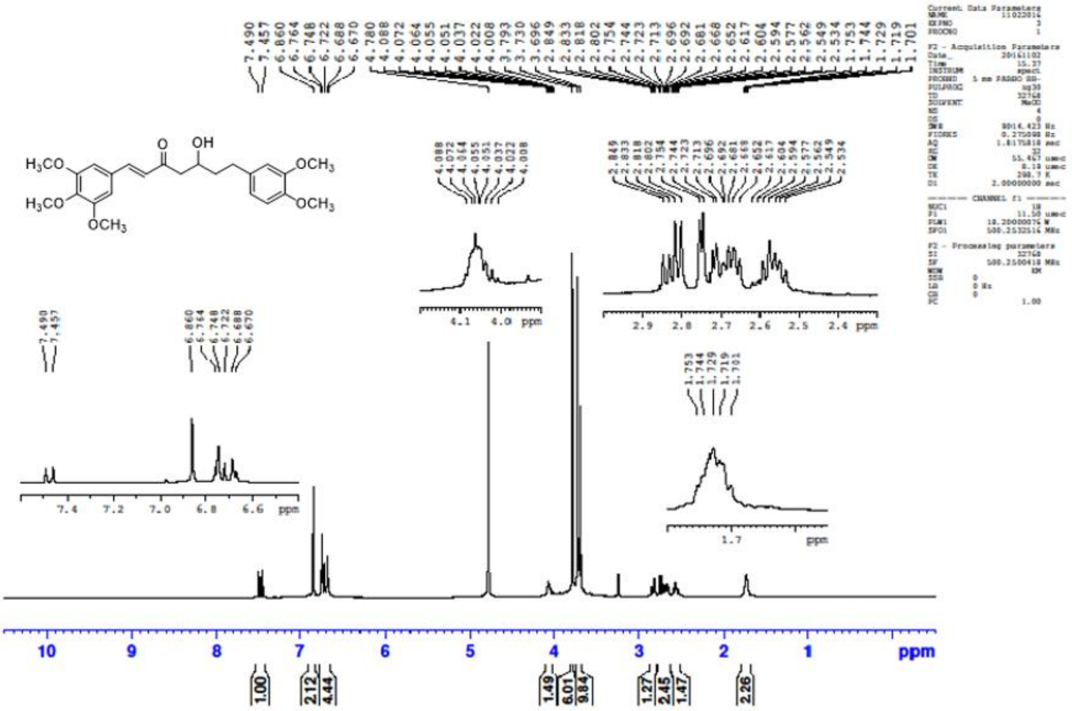

H3CO
H3CO
OCH3
O
OH
OCH3
OCH3
10
9
8
7
6
5
4
3
2
1
ppm
1.00
2.12
4.44
1.49
6.01
9.84
1.27
2.45
1.47
2.26

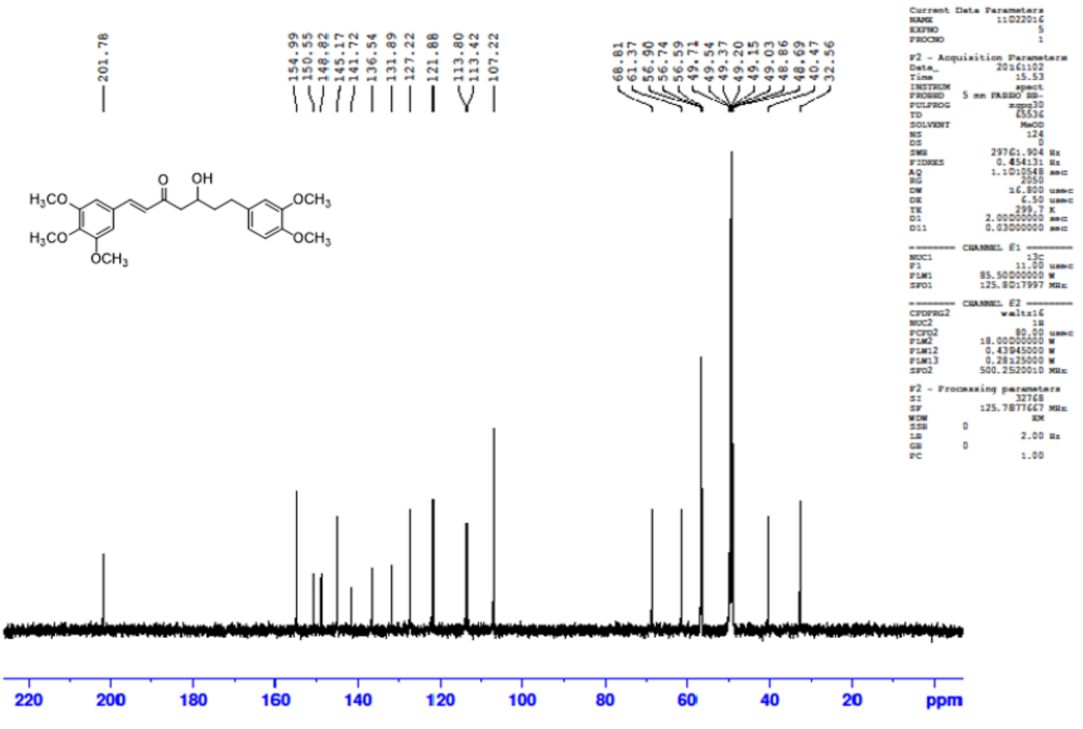

201.78
154.99
150.55
148.82
145.17
141.72
136.54
131.89
127.22
121.88
113.80
113.42
107.22
68.81
61.37
56.90
56.74
56.59
49.71
49.54
49.37
49.20
49.03
48.86
48.69
40.47
32.56
H3CO
H3CO
OCH3
O
OH
OCH3
OCH3
220
200
180
160
140
120
100
80
60
40
20
ppm

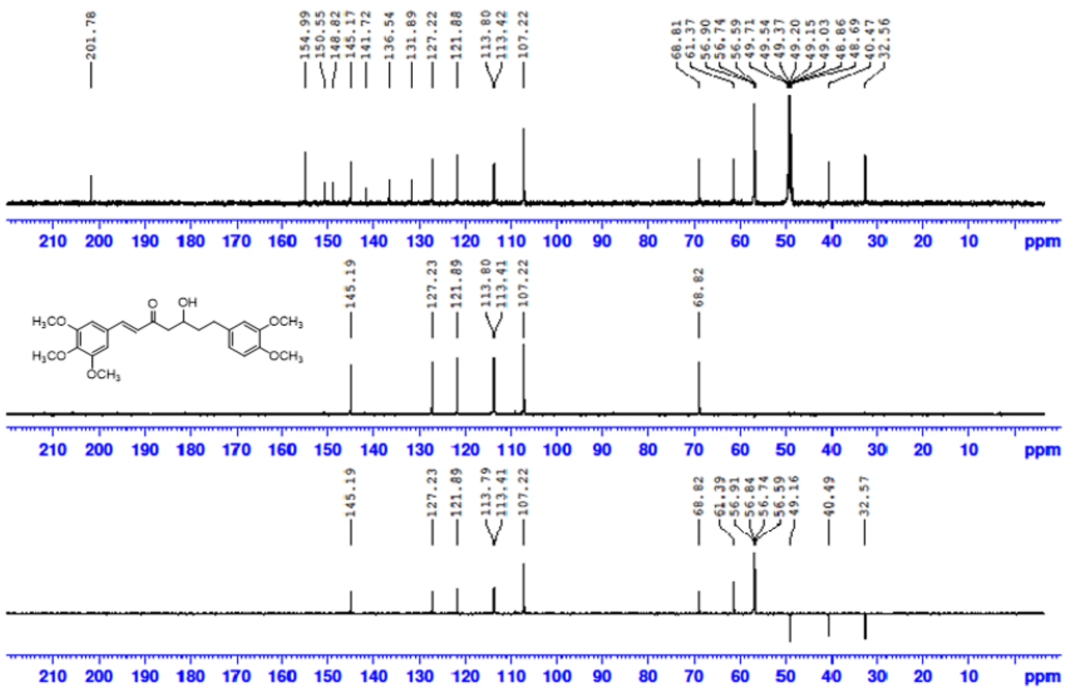

MD8a

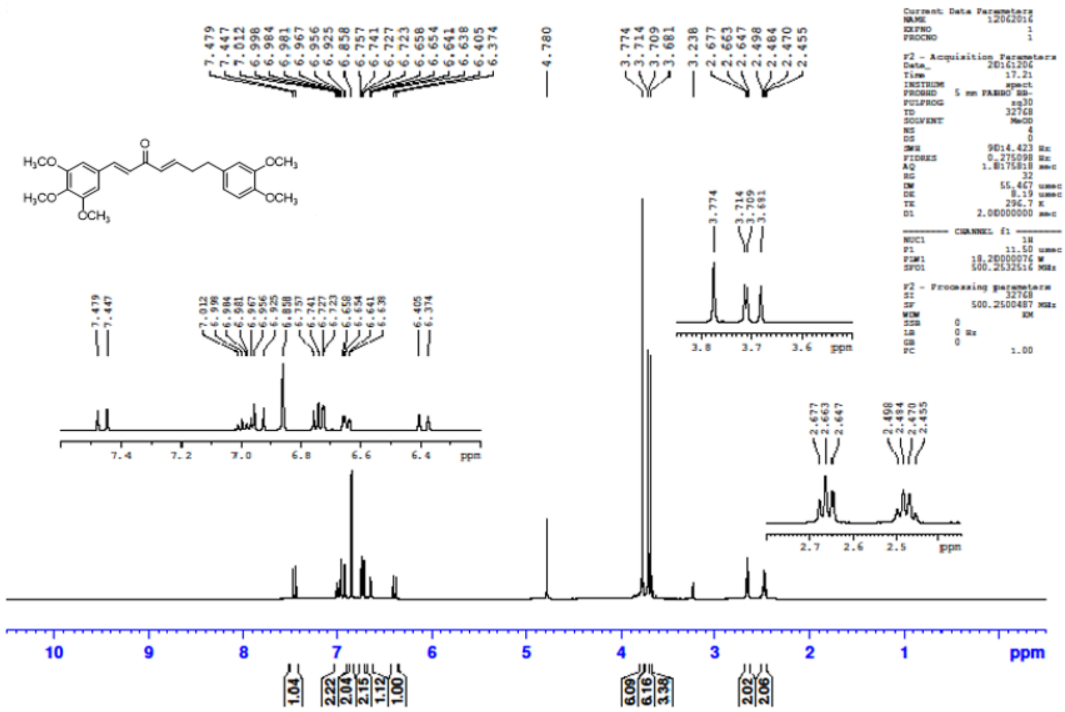

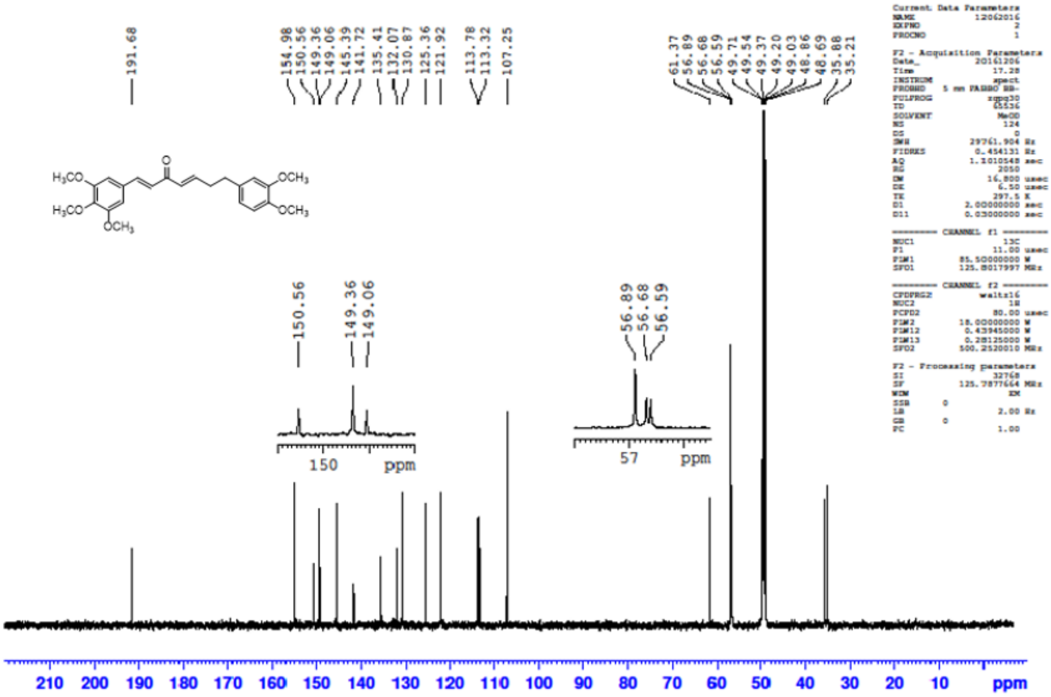
191.68
154.98
150.56
149.36
149.06
145.39
141.72
135.41
132.07
130.87
125.36
121.92
113.78
113.32
107.25
61.37
56.89
56.68
56.59
49.71
49.54
49.37
49.20
49.03
48.86
48.69
35.88
35.21
H3CO
H3CO
OCH3
O
OCH3
OCH3
150.56
149.36
149.06
150
ppm
56.89
56.68
56.59
57
ppm
Current Data Parameters
NAME 12062016
EXPNO 2
PROCNO 1
F2 - Acquisition Parameters
Date_ 20161206
SOLVENT MeOD
NS 124
==== CHANNEL f1 ====
NUC1 13C
==== CHANNEL f2 ====
CPDPRG2 waltz16
NUC2 1H
F2 - Processing parameters
SI 32768
210 200 190 180 170 160 150 140 130 120 110 100 90 80 70 60 50 40 30 20 10 ppm

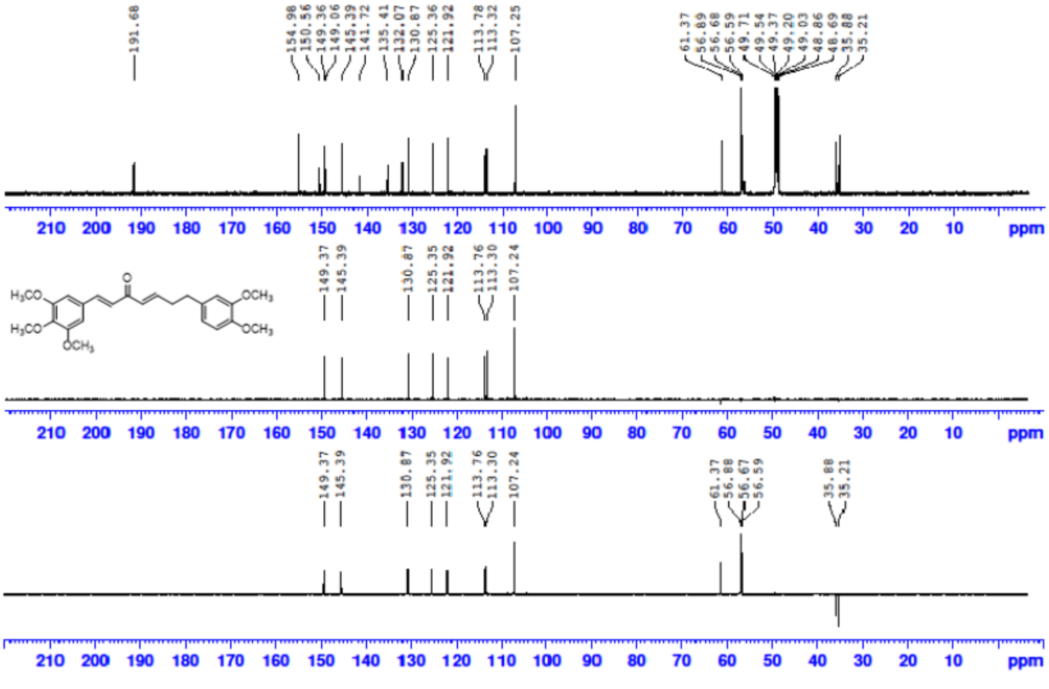
191.68
154.98
150.56
149.36
149.06
145.39
141.72
135.41
132.07
130.87
125.36
121.92
113.78
113.32
107.25
61.37
56.89
56.68
56.59
49.71
49.54
49.37
49.20
49.03
48.86
48.69
35.88
35.21
210 200 190 180 170 160 150 140 130 120 110 100 90 80 70 60 50 40 30 20 10 ppm
H3CO
H3CO
OCH3
O
OCH3
OCH3
149.37
145.39
130.87
125.35
121.92
113.76
113.30
107.24
210 200 190 180 170 160 150 140 130 120 110 100 90 80 70 60 50 40 30 20 10 ppm
149.37
145.39
130.87
125.35
121.92
113.76
113.30
107.24
61.37
56.88
56.67
56.59
35.88
35.21
210 200 190 180 170 160 150 140 130 120 110 100 90 80 70 60 50 40 30 20 10 ppm

MD9

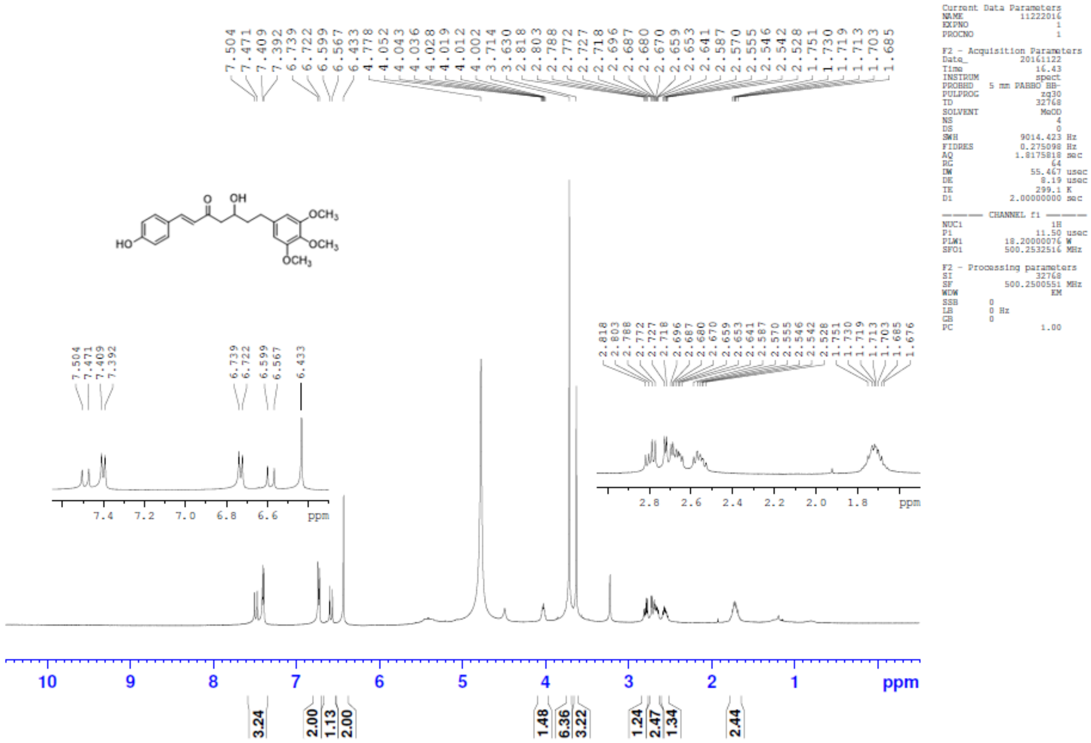
7.504 7.471 7.409 7.392 6.739 6.722 6.599 6.567 6.433 4.778 4.052 4.043 4.036 4.028 4.019 4.012 4.002 3.714 3.630 2.818 2.803 2.788 2.772 2.727 2.718 2.696 2.687 2.680 2.670 2.659 2.653 2.641 2.587 2.570 2.555 2.546 2.542 2.528 1.751 1.730 1.719 1.713 1.703 1.685
HO
O
OH
OCH3
OCH3
OCH3
ppm
3.24
2.00
1.13
2.00
1.48
6.36
3.22
1.24
2.47
1.34
2.44

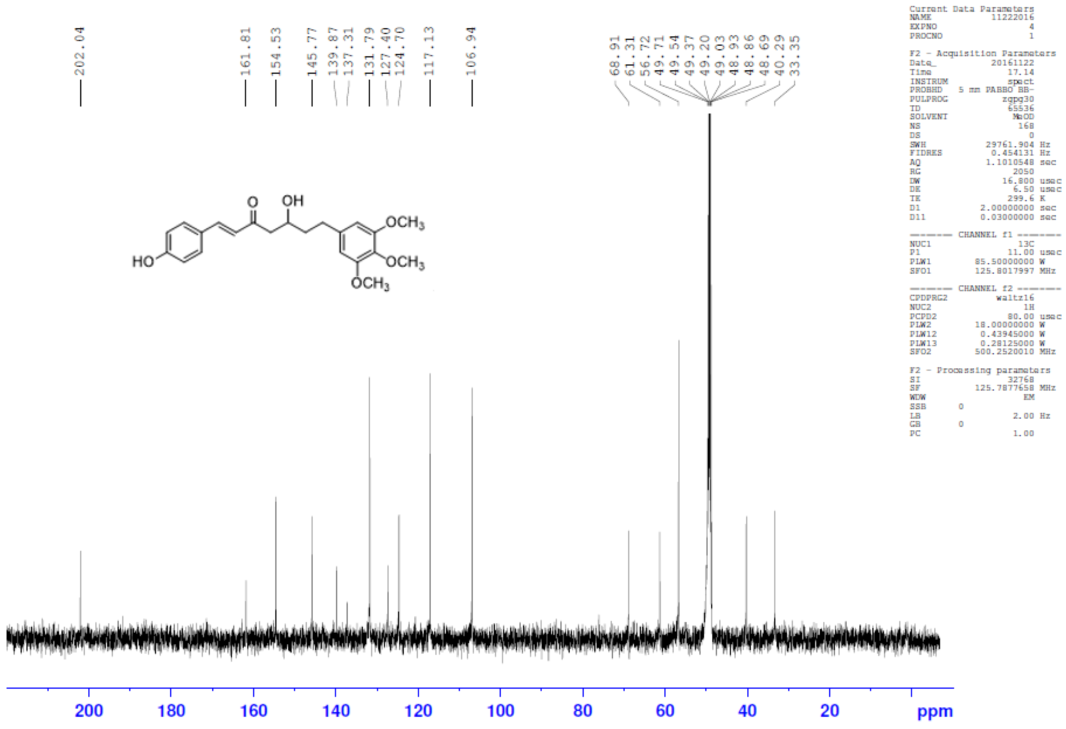
202.04
161.81
154.53
145.77
139.87
137.31
131.79
127.40
124.70
117.13
106.94
68.91
61.31
56.72
49.71
49.54
49.37
49.20
49.03
48.86
48.69
40.29
33.35
HO
O
OH
OCH3
OCH3
OCH3
ppm

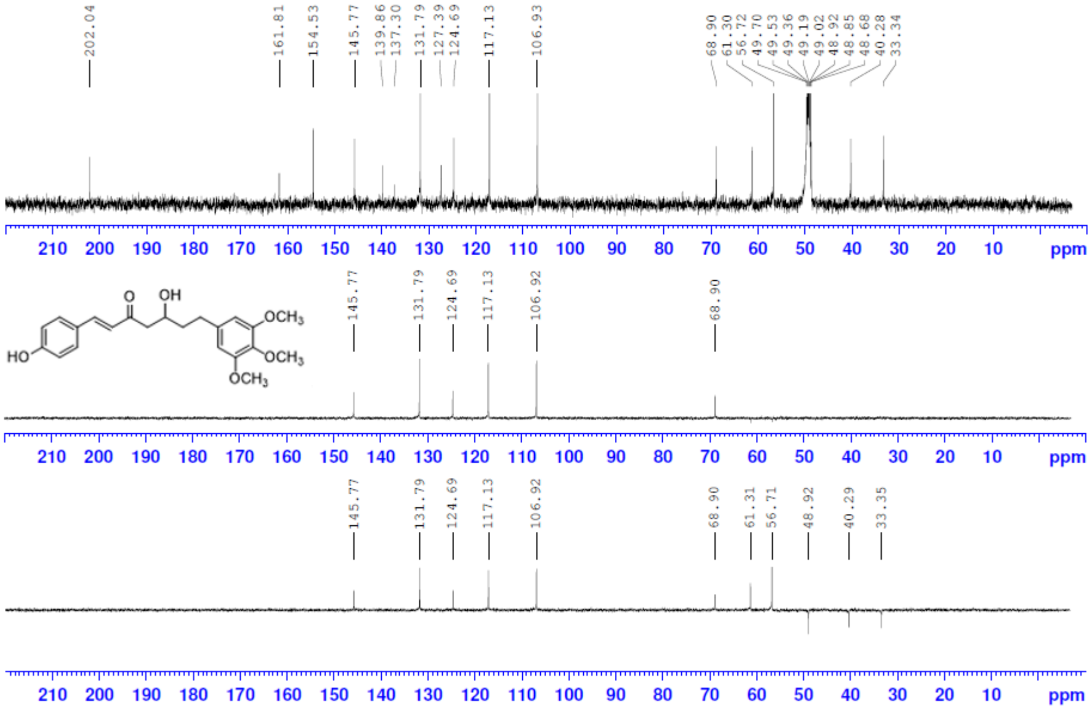

MD9a

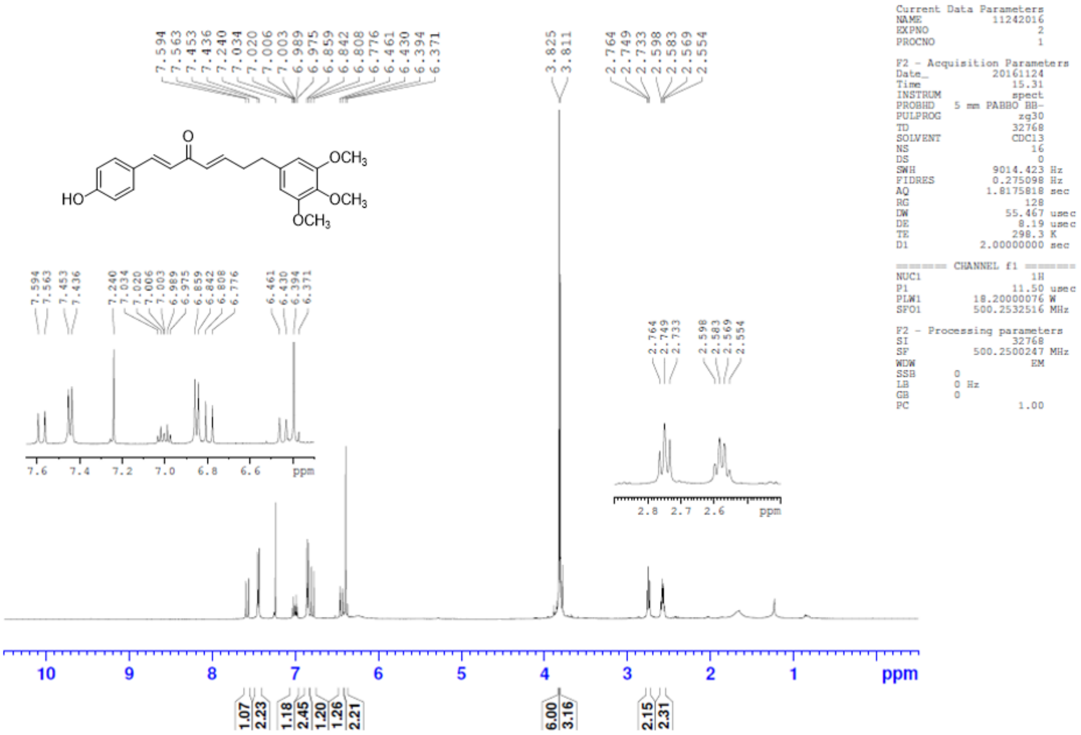

Current Data Parameters
NAME 11242016
EXPNO 2
PROCNO 1
F2 - Acquisition Parameters
Date_ 20161124
Time 15.31
INSTRUM spect
PROBHD 5 mm PABBO BB-
PULPROG zg30
TD 32768
SOLVENT CDCl3
NS 16
DS 0
SWH 9014.423 Hz
FIDRES 0.275098 Hz
AQ 1.8175818 sec
RG 128
DW 55.467 usec
DE 8.19 usec
TE 298.3 K
D1 2.00000000 sec
====== CHANNEL f1 ======
NUC1 1H
P1 11.50 usec
PLW1 18.20000076 W
SFO1 500.2532516 MHz
F2 - Processing parameters
SI 32768
SF 500.2500247 MHz
WDW EM
SSB 0
LB 0 Hz
GB 0
PC 1.00

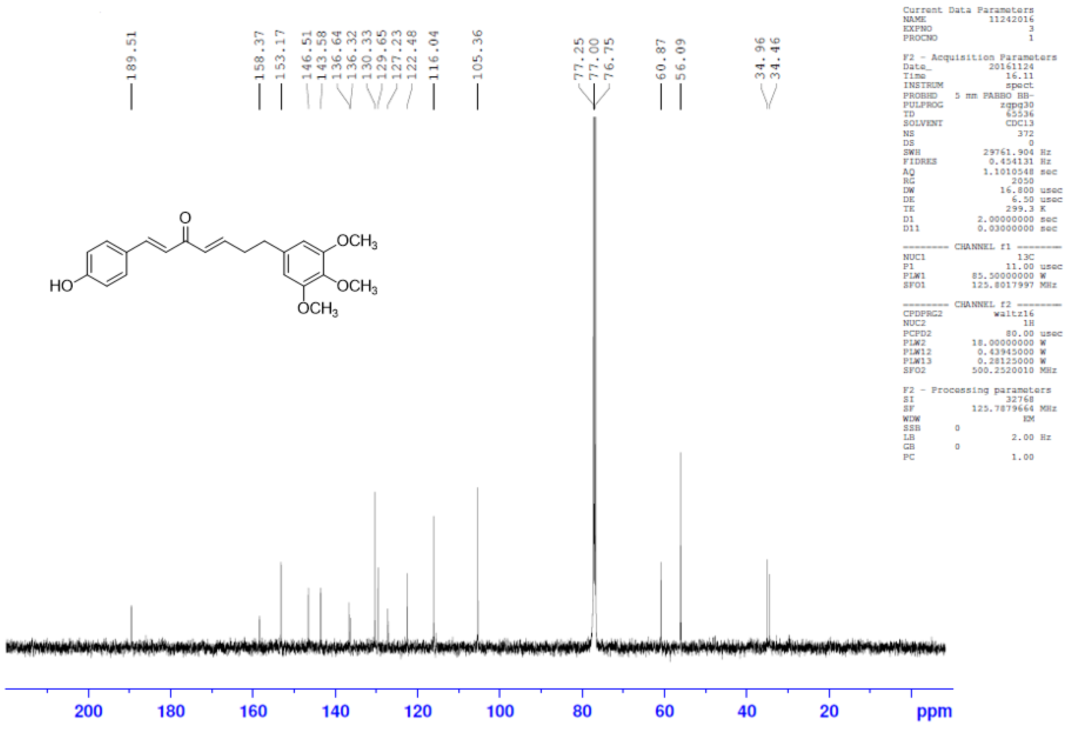
189.51
158.37
153.17
146.51
143.58
136.64
136.32
130.33
129.65
127.23
122.48
116.04
105.36
77.25
77.00
76.75
60.87
56.09
34.96
34.46
O
OCH3
HO
OCH3
OCH3
Current Data Parameters
NAME 11242016
EXPNO 3
PROCNO 1
F2 - Acquisition Parameters
Date_ 20161124
Time 16.11
INSTRUM spect
PROBHD 5 mm PABBO BB-
PULPROG zgpg30
TD 65536
SOLVENT CDCl3
NS 372
DS 0
SWH 29761.904 Hz
FIDRES 0.454131 Hz
AQ 1.1010548 sec
RG 2050
DW 16.800 usec
DE 6.50 usec
TE 299.3 K
D1 2.00000000 sec
D11 0.03000000 sec
======== CHANNEL f1 ========
NUC1 13C
P1 11.00 usec
PLW1 85.50000000 W
SFO1 125.8017997 MHz
======== CHANNEL f2 ========
CPDPRG2 waltz16
NUC2 1H
PCPD2 80.00 usec
PLW2 18.00000000 W
PLW12 0.43945000 W
PLW13 0.28125000 W
SFO2 500.2520010 MHz
F2 - Processing parameters
SI 32768
SF 125.7879664 MHz
WDW EM
SSB 0
LB 2.00 Hz
GB 0
PC 1.00
200 180 160 140 120 100 80 60 40 20 ppm

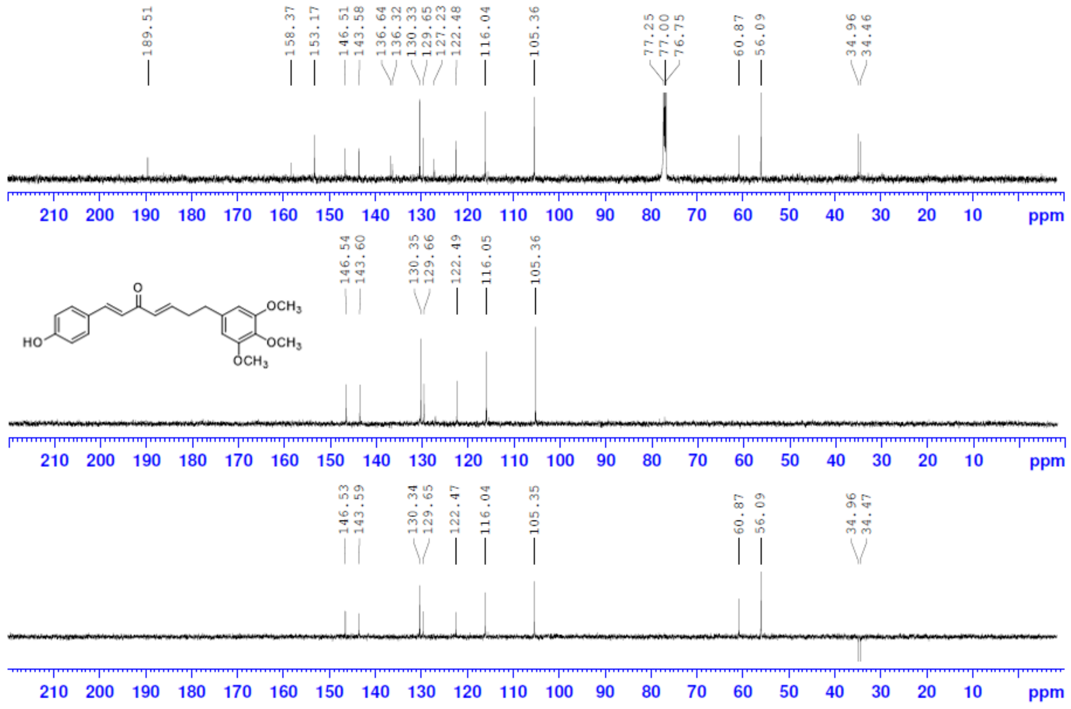
189.51
158.37
153.17
146.51
143.58
136.64
136.32
130.33
129.65
127.23
122.48
116.04
105.36
77.25
77.00
76.75
60.87
56.09
34.96
34.46
210 200 190 180 170 160 150 140 130 120 110 100 90 80 70 60 50 40 30 20 10 ppm
146.54
143.60
130.35
129.66
122.49
116.05
105.36
O
OCH3
HO
OCH3
OCH3
210 200 190 180 170 160 150 140 130 120 110 100 90 80 70 60 50 40 30 20 10 ppm
146.53
143.59
130.34
129.65
122.47
116.04
105.35
60.87
56.09
34.96
34.47
210 200 190 180 170 160 150 140 130 120 110 100 90 80 70 60 50 40 30 20 10 ppm

MD10

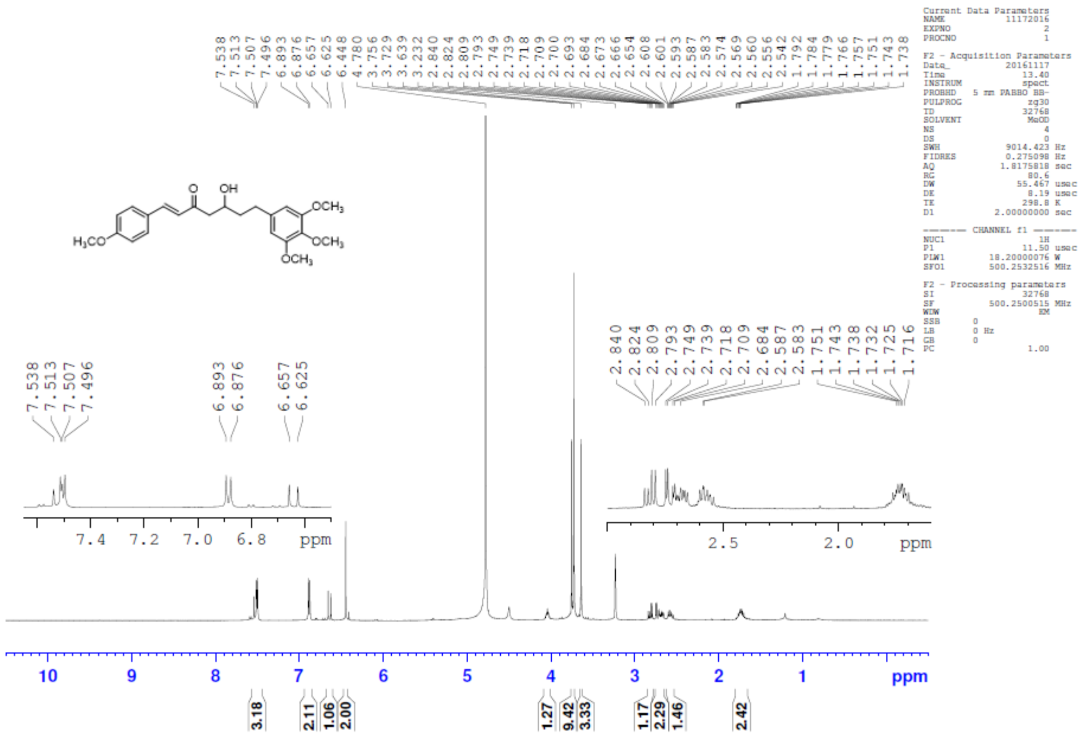
7.538 7.513 7.507 7.496 6.893 6.876 6.657 6.625 6.448 4.780 3.756 3.729 3.639 3.232 2.840 2.824 2.809 2.793 2.749 2.739 2.718 2.709 2.700 2.693 2.684 2.673 2.666 2.654 2.608 2.601 2.593 2.587 2.583 2.574 2.569 2.560 2.556 2.542 1.792 1.784 1.779 1.766 1.757 1.751 1.743 1.738
ppm
3.18 2.11 1.06 2.00 1.27 9.42 3.33 1.17 2.29 1.46 2.42

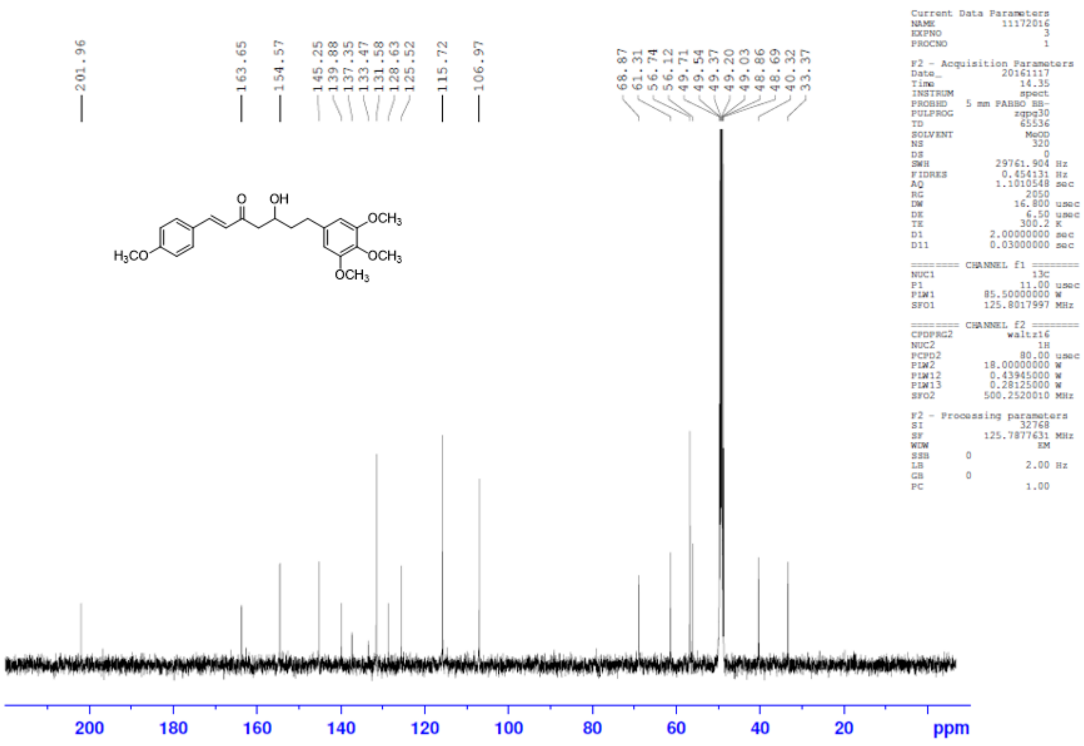
201.96 163.65 154.57 145.25 139.88 137.35 133.47 131.58 128.63 125.52 115.72 106.97 68.87 61.31 56.74 56.12 49.71 49.54 49.37 49.20 49.03 48.86 48.69 40.32 33.37
ppm

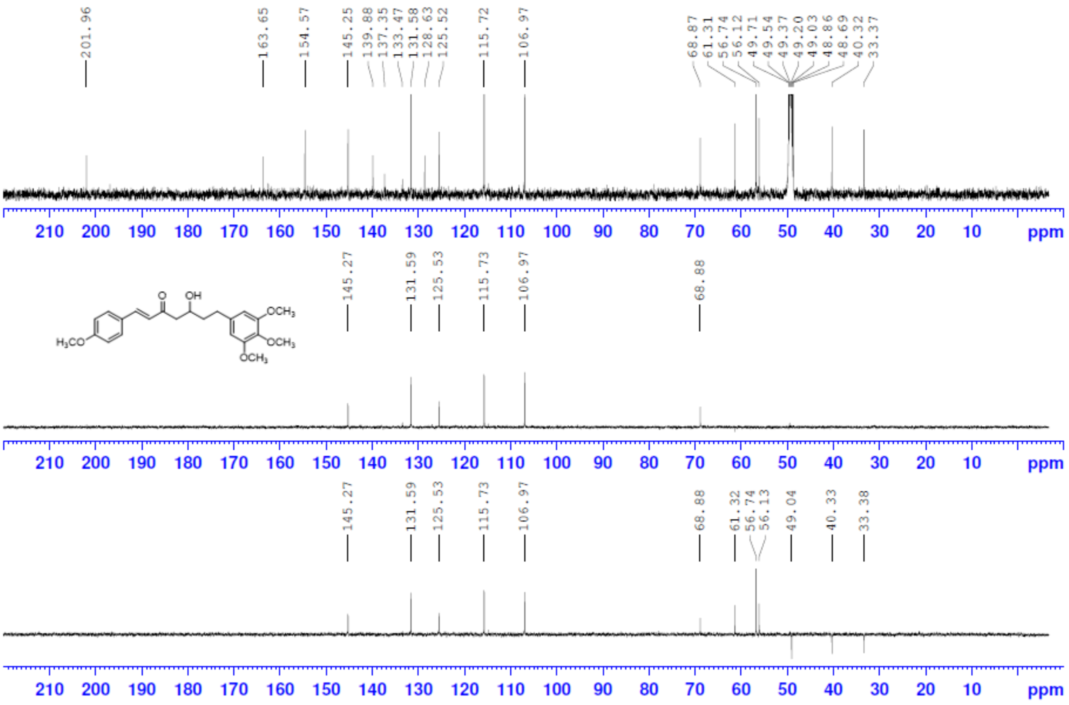
201.96
163.65
154.57
145.25
139.88
137.35
133.47
131.58
128.63
125.52
115.72
106.97
68.87
61.31
56.74
56.12
49.71
49.54
49.37
49.20
49.03
48.86
48.69
40.32
33.37
ppm
145.27
131.59
125.53
115.73
106.97
68.88
OH
O
OCH_3
H_3CO
OCH_3
OCH_3
ppm
145.27
131.59
125.53
115.73
106.97
68.88
61.32
56.74
56.13
49.04
40.33
33.38
ppm

MD10a

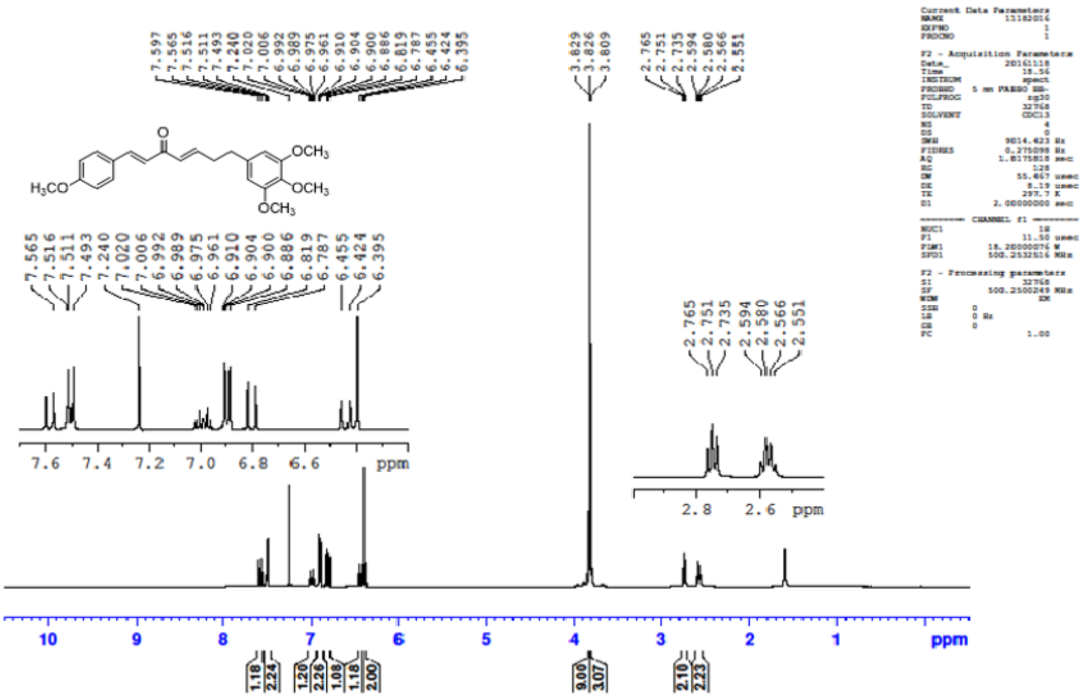
7.597
7.565
7.516
7.511
7.493
7.240
7.020
7.006
6.992
6.989
6.975
6.961
6.910
6.904
6.900
6.886
6.819
6.787
6.455
6.424
6.395
3.829
3.826
3.809
2.765
2.751
2.735
2.594
2.580
2.566
2.551
O
OCH_3
H_3CO
OCH_3
OCH_3
ppm
ppm
ppm
1.18
2.24
1.20
2.26
1.08
1.18
2.00
9.00
3.07
2.10
2.23

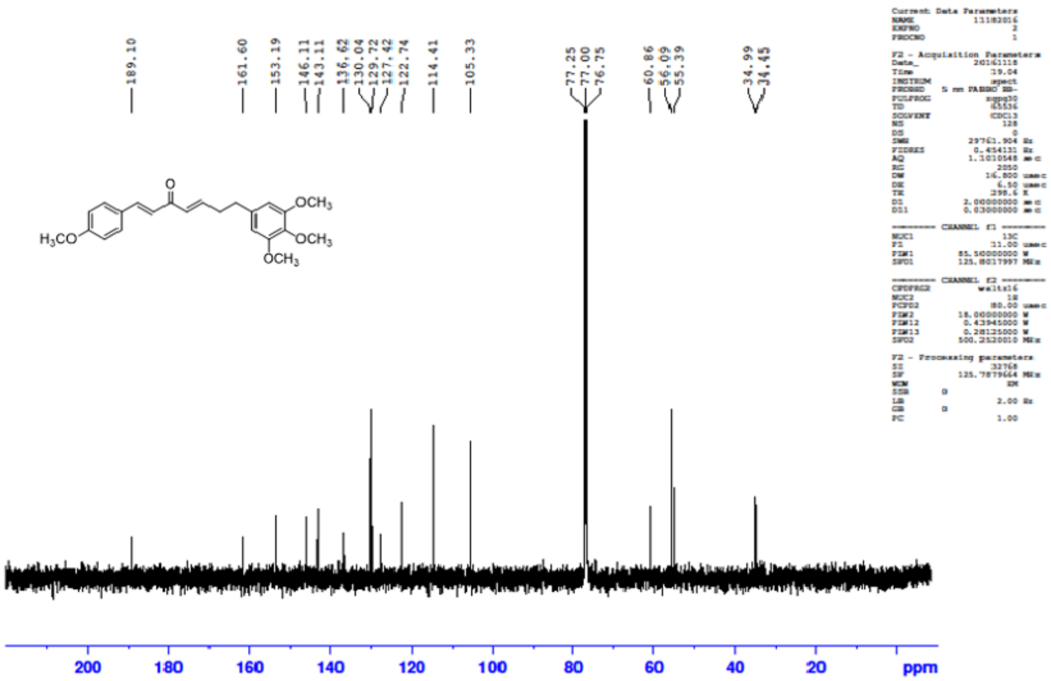
189.10
161.60
153.19
146.11
143.11
136.62
130.04
129.72
127.42
122.74
114.41
105.33
77.25
77.00
76.75
60.86
56.09
55.39
34.99
34.45
OCH3
H3CO
OCH3
OCH3
O
200 180 160 140 120 100 80 60 40 20 ppm

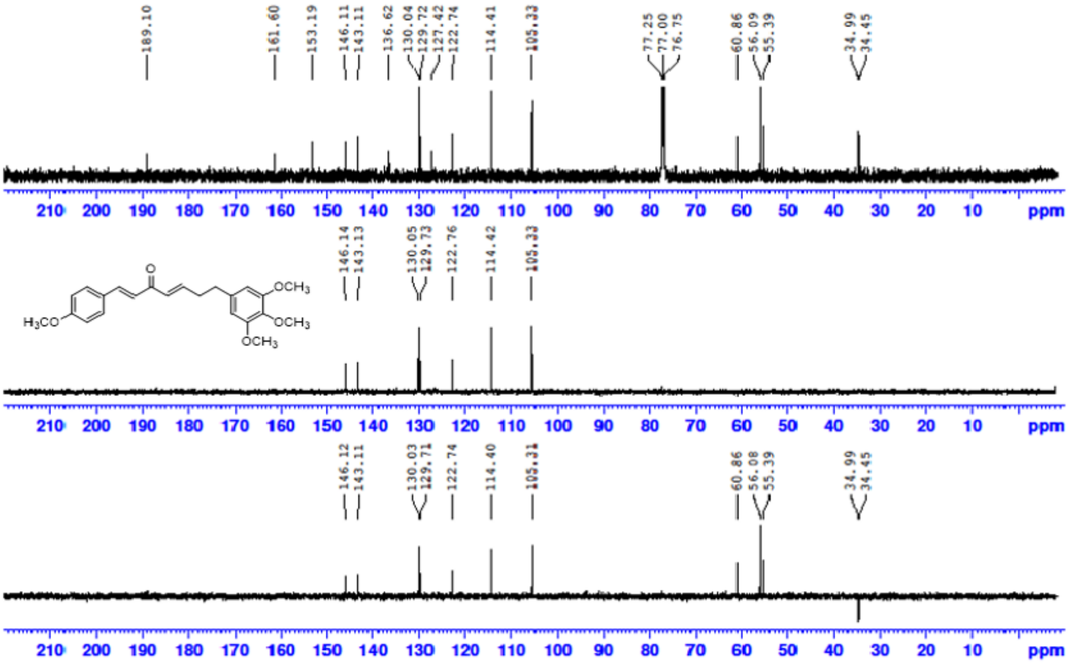
189.10
161.60
153.19
146.11
143.11
136.62
130.04
129.72
127.42
122.74
114.41
105.33
77.25
77.00
76.75
60.86
56.09
55.39
34.99
34.45
210 200 190 180 170 160 150 140 130 120 110 100 90 80 70 60 50 40 30 20 10 ppm
146.14
143.13
130.05
129.73
122.76
114.42
105.33
OCH3
H3CO
OCH3
OCH3
O
210 200 190 180 170 160 150 140 130 120 110 100 90 80 70 60 50 40 30 20 10 ppm
146.12
143.11
130.03
129.71
122.74
114.40
105.31
60.86
56.08
55.39
34.99
34.45
210 200 190 180 170 160 150 140 130 120 110 100 90 80 70 60 50 40 30 20 10 ppm

MD11

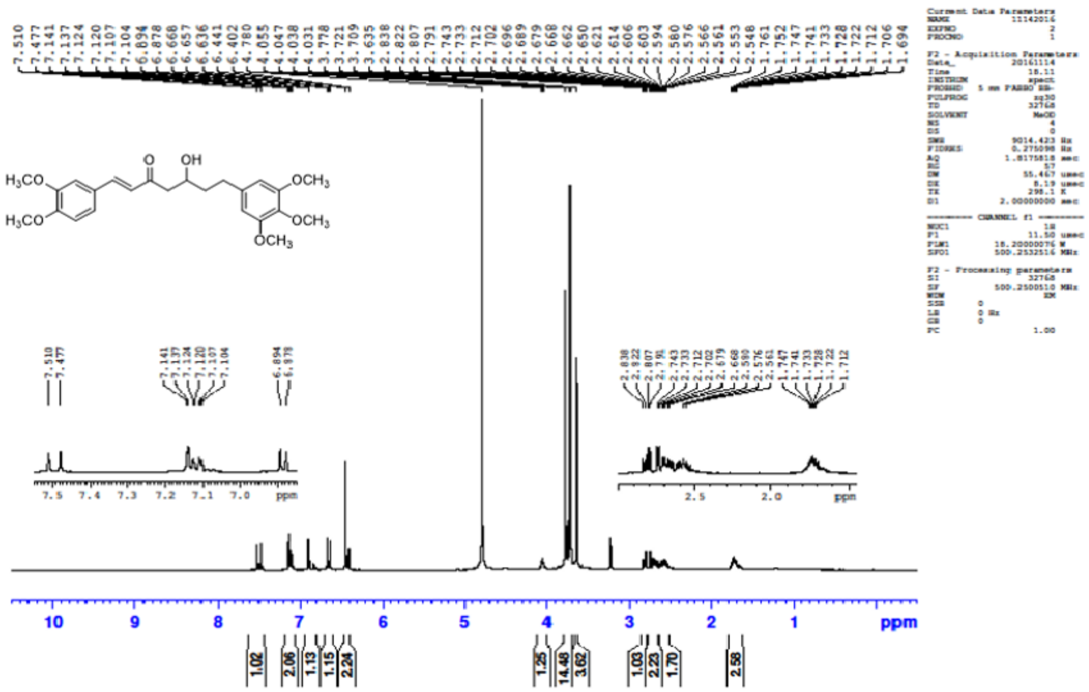

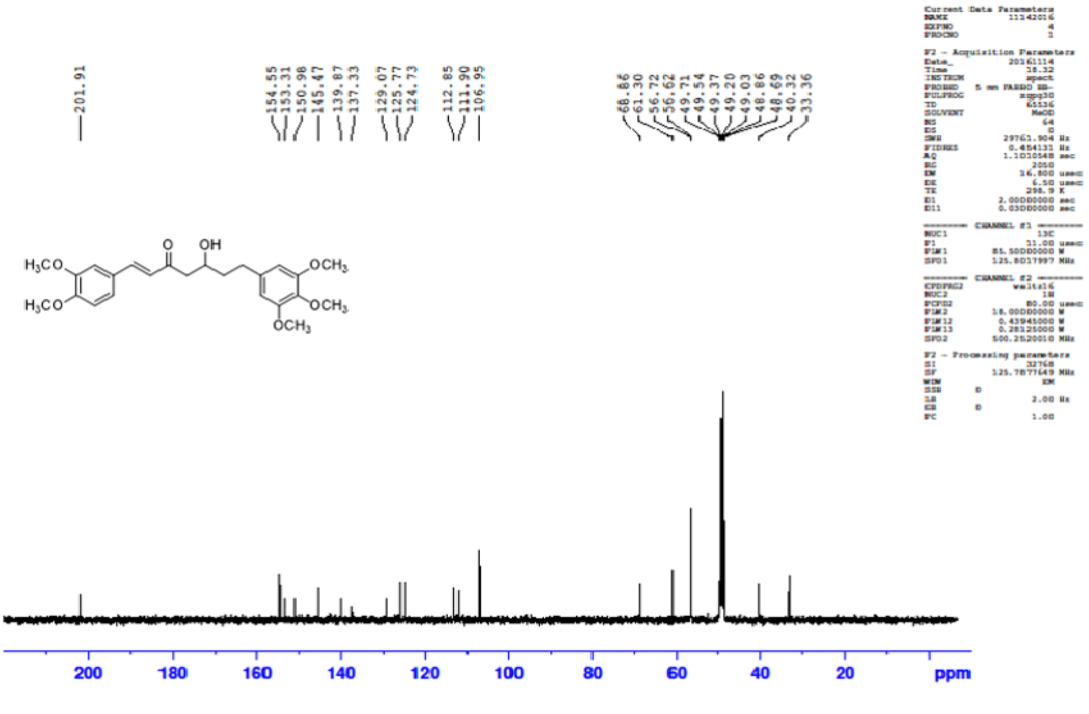

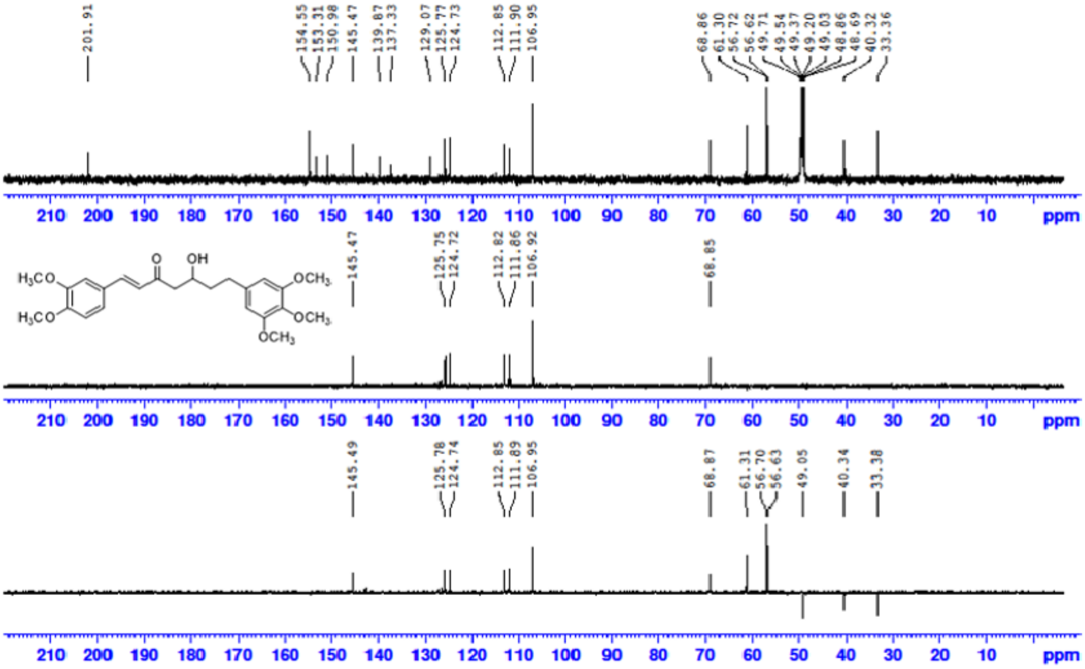
201.91
154.55
153.31
150.98
145.47
139.87
137.33
129.07
125.77
124.73
112.85
111.90
106.95
68.86
61.30
56.72
56.62
49.71
49.54
49.37
49.20
49.03
48.86
48.69
40.32
33.36
210 200 190 180 170 160 150 140 130 120 110 100 90 80 70 60 50 40 30 20 10 ppm
H_3CO
H_3CO
O
OH
OCH_3
OCH_3
OCH_3
145.47
125.75
124.72
112.82
111.86
106.92
68.85
210 200 190 180 170 160 150 140 130 120 110 100 90 80 70 60 50 40 30 20 10 ppm
145.49
125.78
124.74
112.85
111.89
106.95
68.87
61.31
56.70
56.63
49.05
40.34
33.38
210 200 190 180 170 160 150 140 130 120 110 100 90 80 70 60 50 40 30 20 10 ppm

MD11a

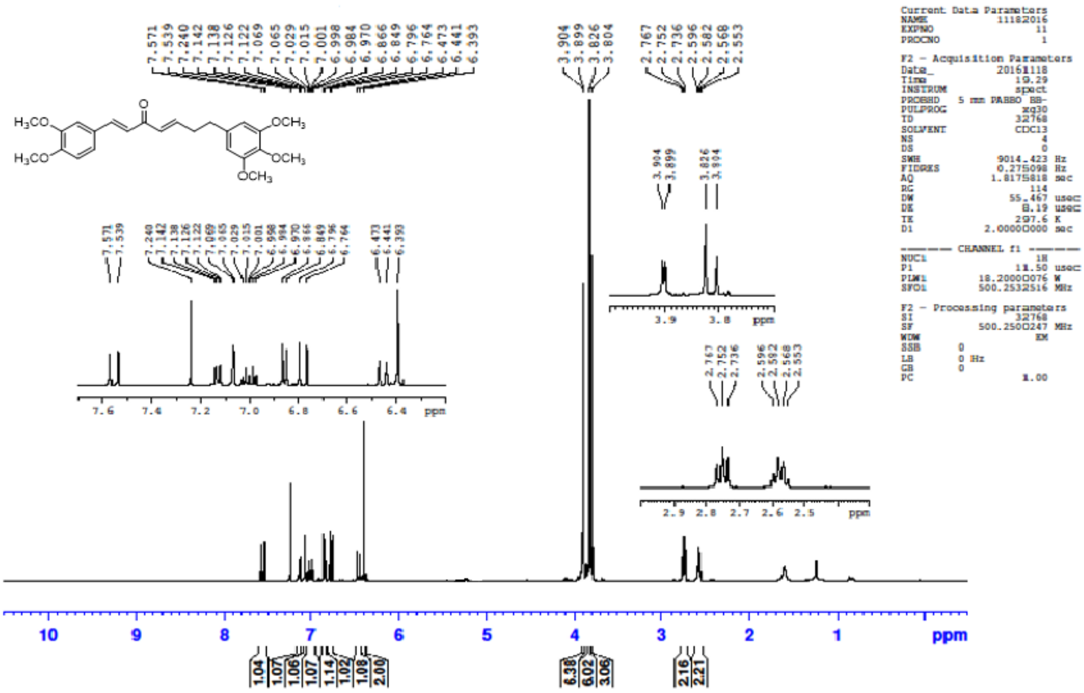
7.571
7.539
7.240
7.142
7.138
7.126
7.122
7.069
7.065
7.029
7.015
7.001
6.998
6.984
6.970
6.866
6.849
6.796
6.764
6.473
6.441
6.393
3.904
3.899
3.826
3.804
2.767
2.752
2.736
2.596
2.582
2.568
2.553
H_3CO
H_3CO
O
OCH_3
OCH_3
OCH_3
7.6 7.4 7.2 7.0 6.8 6.6 6.4 ppm
3.9 3.8 ppm
2.9 2.8 2.7 2.6 2.5 ppm
10 9 8 7 6 5 4 3 2 1 ppm
1.04
1.07
1.06
1.07
1.14
1.02
1.08
2.00
6.38
6.02
3.06
2.16
2.21
Current Data Parameters
NAME
EXPNO 11
PROCNO 1
F2 - Acquisition Parameters
INSTRUM spect
SOLVENT CDCl3
NS 4
DS 0
SWH 9014.423 Hz
RG 114
DW 55.467 usec
TE 297.6 K
D1 2.00000000 sec
CHANNEL f1
NUC1 1H
SFO1 500.2532516 MHz
F2 - Processing parameters
SI 32768
SF 500.2500247 MHz
WDW EM
SSB 0
LB 0 Hz
GB 0

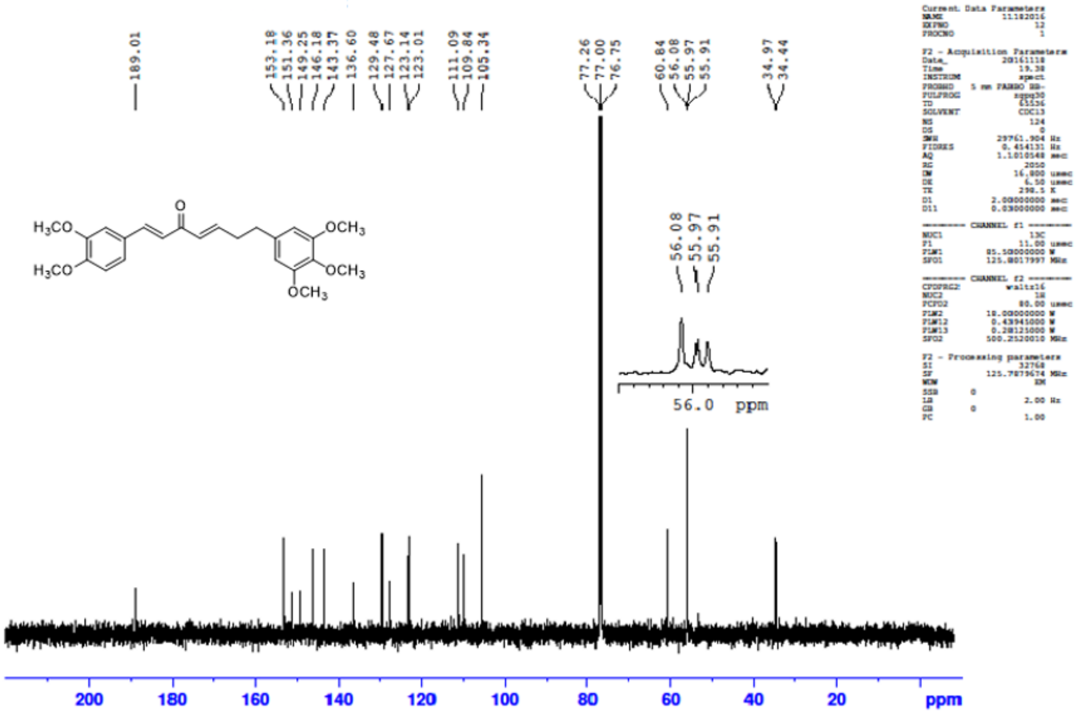
189.01
153.18
151.36
149.25
146.18
143.37
136.60
129.48
127.67
123.14
123.01
111.09
109.84
105.34
77.26
77.00
76.75
60.84
56.08
55.97
55.91
34.97
34.44
56.0 ppm
200
180
160
140
120
100
80
60
40
20
ppm

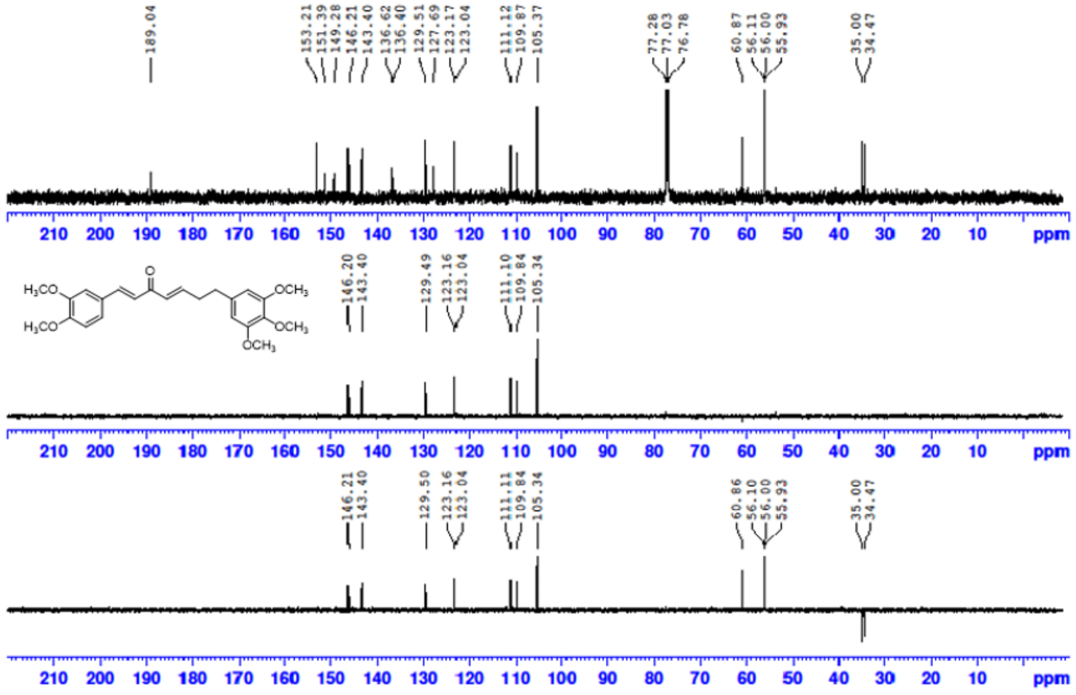
189.04
153.21
151.39
149.28
146.21
143.40
136.62
136.40
129.51
127.69
123.17
123.04
111.12
109.87
105.37
77.28
77.03
76.78
60.87
56.11
56.00
55.93
35.00
34.47
146.20
143.40
129.49
123.16
123.04
111.10
109.84
105.34
146.21
143.40
129.50
123.16
123.04
111.11
109.84
105.34
60.86
56.10
56.00
55.93
35.00
34.47
210 200 190 180 170 160 150 140 130 120 110 100 90 80 70 60 50 40 30 20 10 ppm

MD12

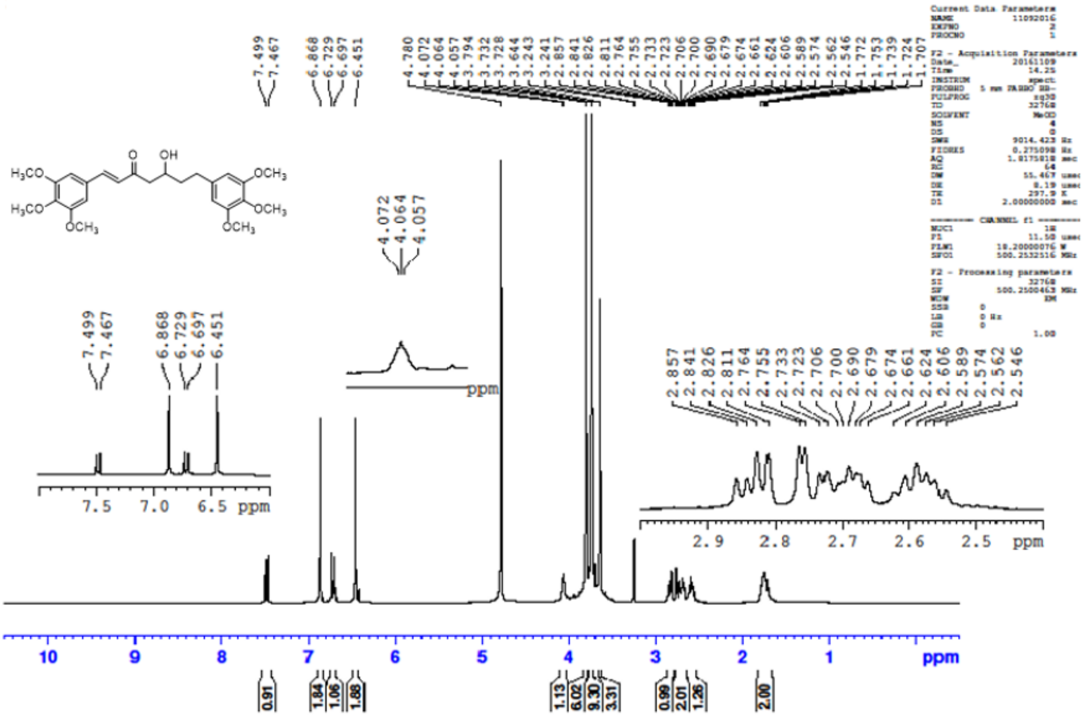
7.499
7.467
6.868
6.729
6.697
6.451
4.780
4.072
4.064
4.057
3.794
3.732
3.728
3.644
3.243
3.241
2.857
2.841
2.826
2.811
2.764
2.755
2.733
2.723
2.706
2.700
2.690
2.679
2.674
2.661
2.624
2.606
2.589
2.574
2.562
2.546
1.772
1.753
1.739
1.724
1.707
ppm
0.91
1.84
1.06
1.88
1.13
6.02
9.30
3.31
0.99
2.01
1.26
2.00

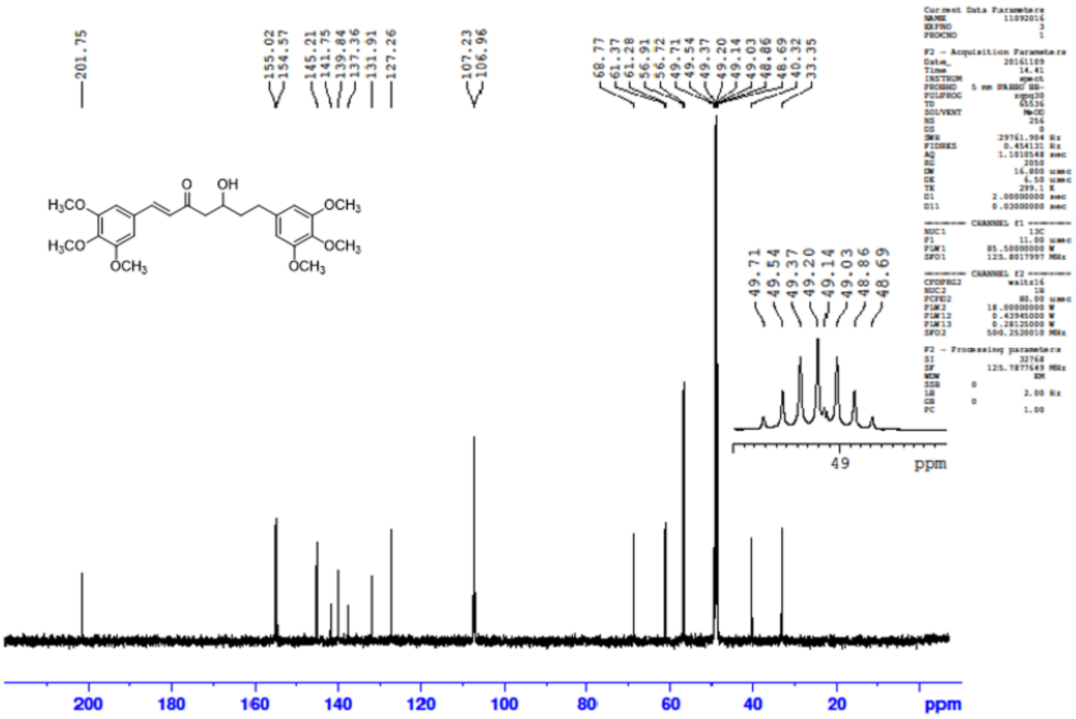
201.75
155.02
154.57
145.21
141.75
139.84
137.36
131.91
127.26
107.23
106.96
68.77
61.37
61.28
56.91
56.72
49.71
49.54
49.37
49.20
49.14
49.03
48.86
48.69
40.32
33.35
ppm

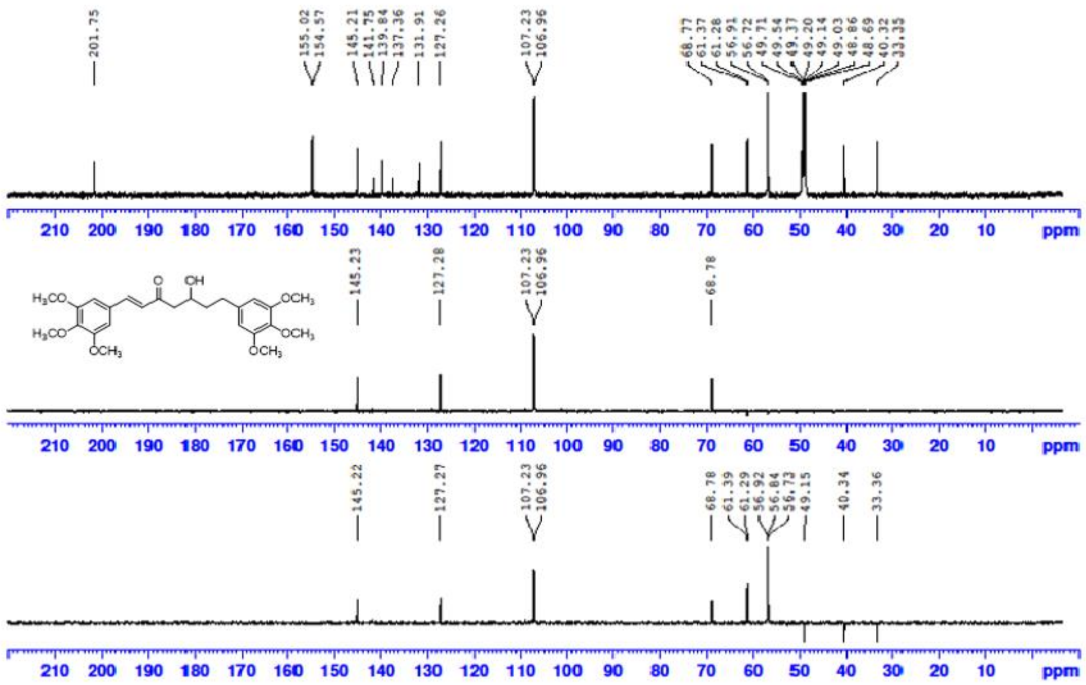
201.75
155.02
154.57
145.21
141.75
139.84
137.36
131.91
127.26
107.23
106.96
68.77
61.37
61.28
56.91
56.72
40.32
33.35
145.23
127.28
107.23
106.96
68.78
145.22
127.27
107.23
106.96
68.78
61.39
61.29
56.92
56.84
56.73
49.15
40.34
33.36
ppm

MD12a

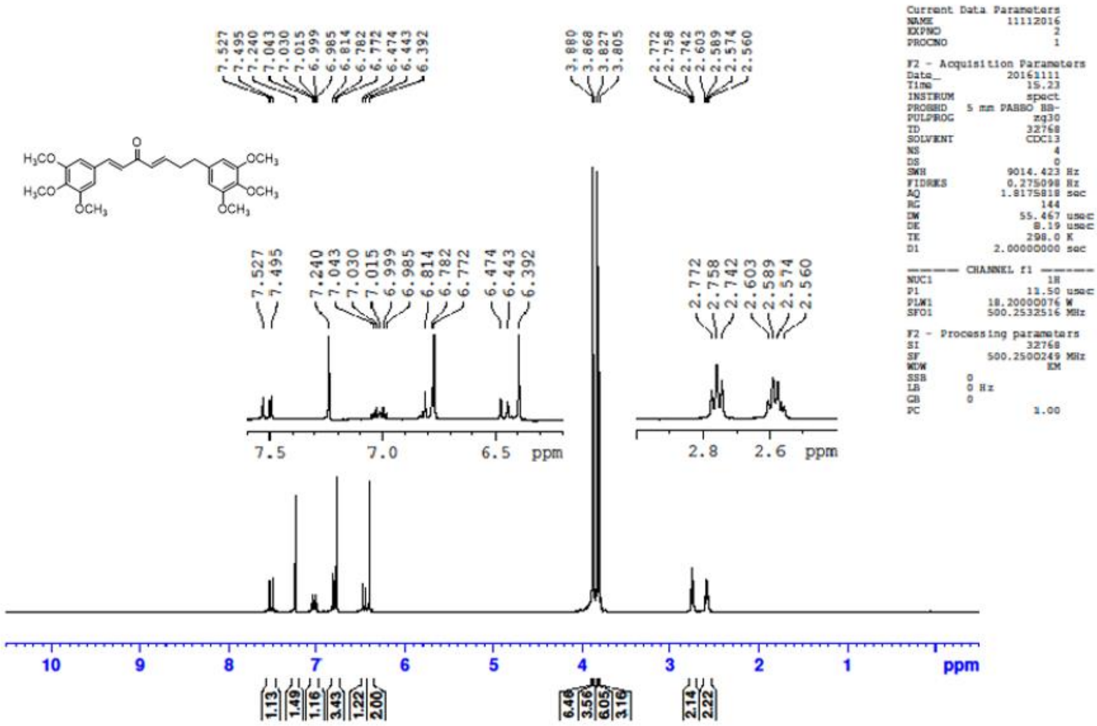
7.527
7.495
7.240
7.043
7.030
7.015
6.999
6.985
6.814
6.782
6.772
6.474
6.443
6.392
3.880
3.868
3.827
3.805
2.772
2.758
2.742
2.603
2.589
2.574
2.560
ppm

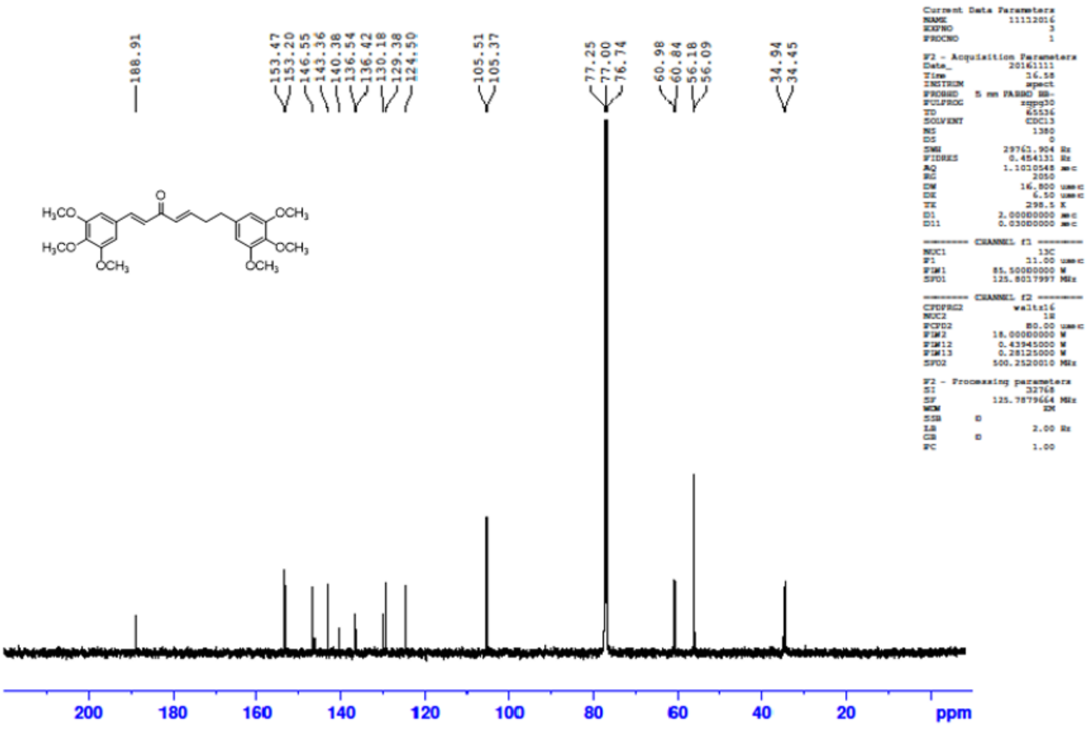
188.91
153.47
153.20
146.55
143.36
140.38
136.54
136.42
130.18
129.38
124.50
105.51
105.37
77.25
77.00
76.74
60.98
60.84
56.18
56.09
34.94
34.45
200 180 160 140 120 100 80 60 40 20 ppm

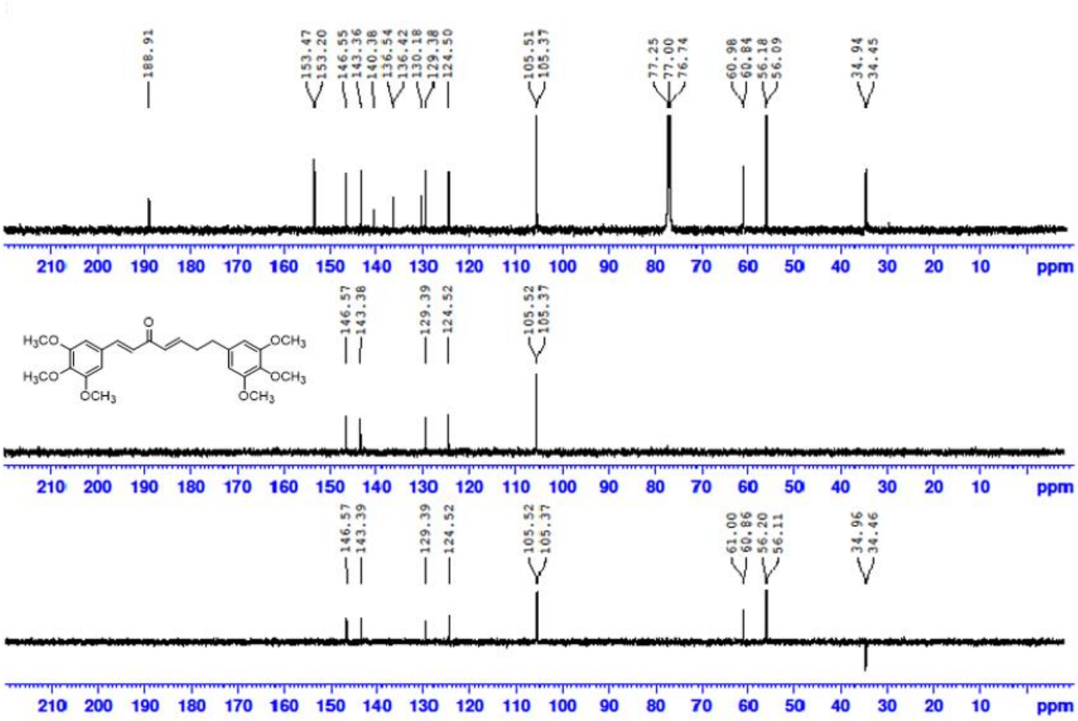
188.91
153.47
153.20
146.55
143.36
140.38
136.54
136.42
130.18
129.38
124.50
105.51
105.37
77.25
77.00
76.74
60.98
60.84
56.18
56.09
34.94
34.45
146.57
143.38
129.39
124.52
105.52
105.37
146.57
143.39
129.39
124.52
105.52
105.37
61.00
60.86
56.20
56.11
34.96
34.46
210 200 190 180 170 160 150 140 130 120 110 100 90 80 70 60 50 40 30 20 10 ppm

MD13

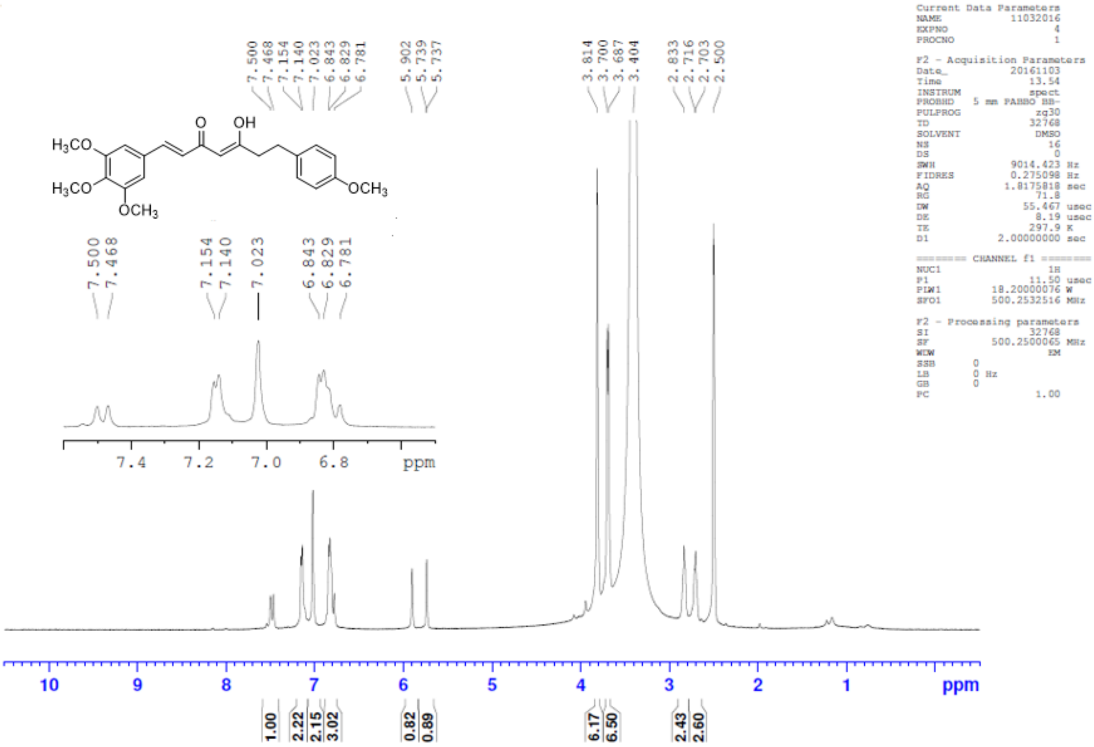

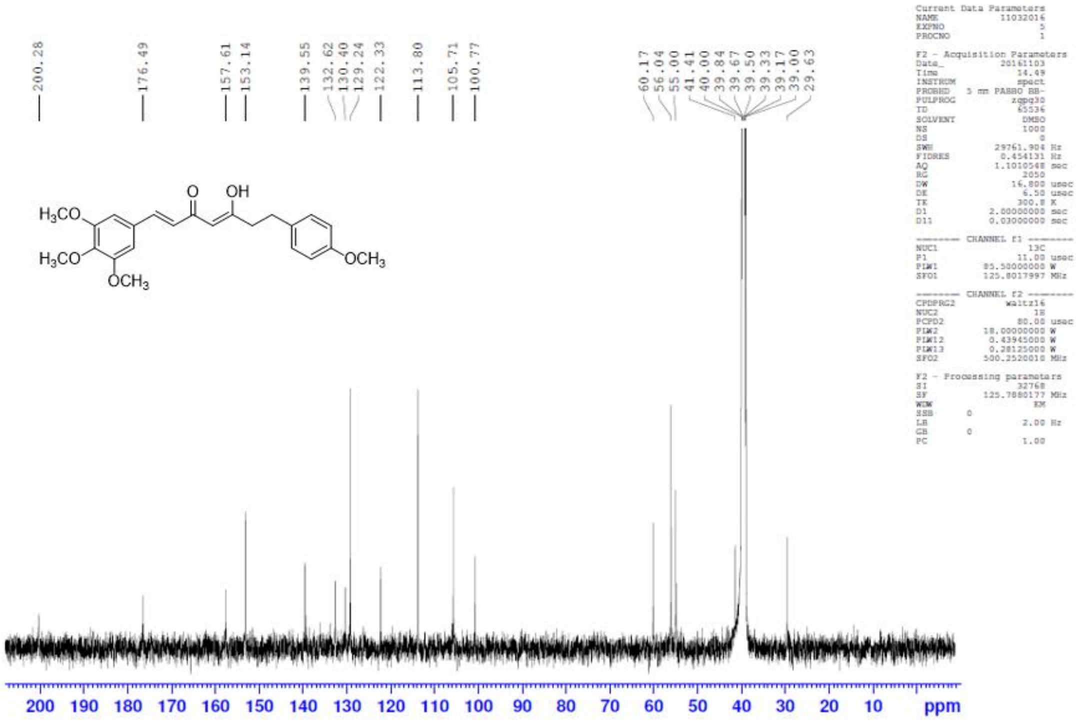

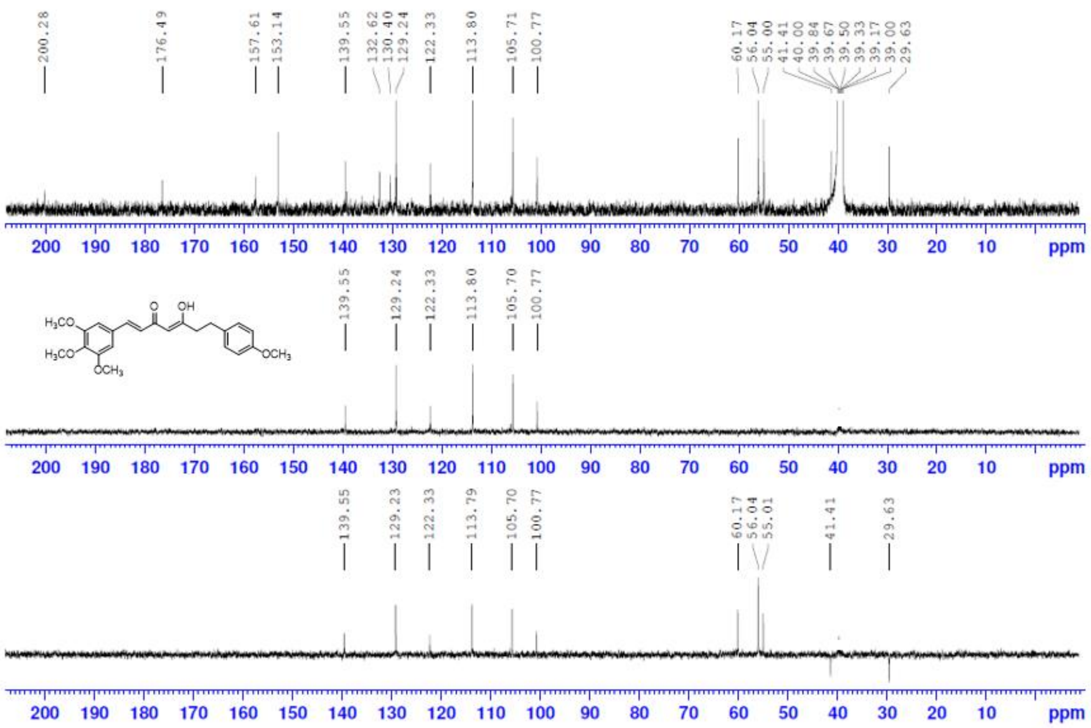
200.28
176.49
157.61
153.14
139.55
132.62
130.40
129.24
122.33
113.80
105.71
100.77
60.17
56.04
55.00
41.41
40.00
39.84
39.67
39.50
39.33
39.17
39.00
29.63
200 190 180 170 160 150 140 130 120 110 100 90 80 70 60 50 40 30 20 10 ppm
139.55
129.24
122.33
113.80
105.70
100.77
H3CO
H3CO
OCH3
O
OH
OCH3
200 190 180 170 160 150 140 130 120 110 100 90 80 70 60 50 40 30 20 10 ppm
139.55
129.23
122.33
113.79
105.70
100.77
60.17
56.04
55.01
41.41
29.63
200 190 180 170 160 150 140 130 120 110 100 90 80 70 60 50 40 30 20 10 ppm
